# Pd(II)-Catalyzed Aminoacetoxylation of Alkenes Via Tether Formation

**DOI:** 10.1021/acs.orglett.2c01838

**Published:** 2022-07-11

**Authors:** Thomas Rossolini, Ashis Das, Stefano Nicolai, Jérôme Waser

**Affiliations:** Laboratory of Catalysis and Organic Synthesis and National Centre of Competence in Research Catalysis, Institute of Chemical Sciences and Engineering, Ecole Polytechnique Federale de Lausanne, EPFL, 1015 Lausanne, Switzerland

## Abstract

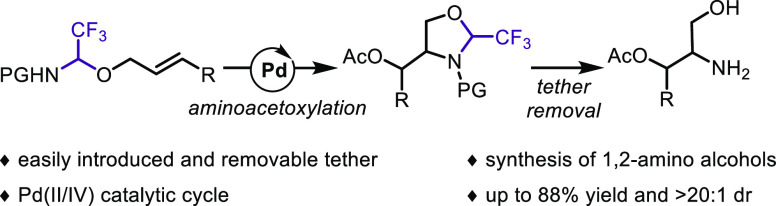

A Pd-catalyzed method based on the use of a molecular tether is described for olefin difunctionalization. Enabled by an easily introduced trifluoroacetaldehyde-derived tether, a simultaneous introduction of oxygen and nitrogen heteroatoms across unsaturated carbon–carbon bonds was achieved under oxidative conditions, most probably via high-valent Pd intermediates. Good yields and high diastereoselectivity were obtained with aryl-substituted alkenes, whereas nonterminal alkyl-substituted olefins gave aza-Heck products. Tether cleavage under mild conditions provided fast access to functionalized β-amino alcohols.

Recent advances in catalytic alkene multifunctionalization have significantly facilitated the generation of molecular complexity from simple precursors due to the broad accessibility and unparalleled reactivity of olefins.^1^ Palladium-catalyzed processes, in particular, have led to important progress in the field of alkene derivatization,^2^ ranging from standard intermolecular cross-coupling reactions to cascade cyclizations in natural product synthesis.^3^ Despite these improvements, reactivity and selectivity challenges frequently encountered in intermolecular transition-metal catalyzed reactions limit the broad application of these transformations. Consequently, recent efforts have been focused on the development of transient intramolecular pathways to gain a better control of both reactivity and selectivity.^4,5^ Early works concentrated on the use of carbamate or imidate tethers, but the reaction precursors had to be isolated prior to the reaction, and harsh conditions were often required for removal of the tethers.^6^ In an effort to solve these issues, our group introduced acetal-based tethers in the context of Pd^0^/Pd^II^ catalysis for selective alkene functionalization.^7^ In 2017, we reported a Pd-catalyzed carboamination reaction of allylic alcohols for the synthesis of amino alcohols exploiting a trifluoroacetaldehyde-derived tethering strategy (Scheme 1a).^8^ Despite broad applicability, this Pd^0^/Pd^II^ methodology was only efficient for C–C bond formation across terminal alkenes. We therefore aimed to develop an alternative olefin vicinal difunctionalization leading to C–X bond formation, ideally also applicable to internal alkenes. In particular, we sought to investigate a novel tethering Pd^II^/Pd^IV^-based manifold toward oxidative alkene difunctionalization to access multiple carbon-heteroatom bonds.^9^

**Scheme 1:**
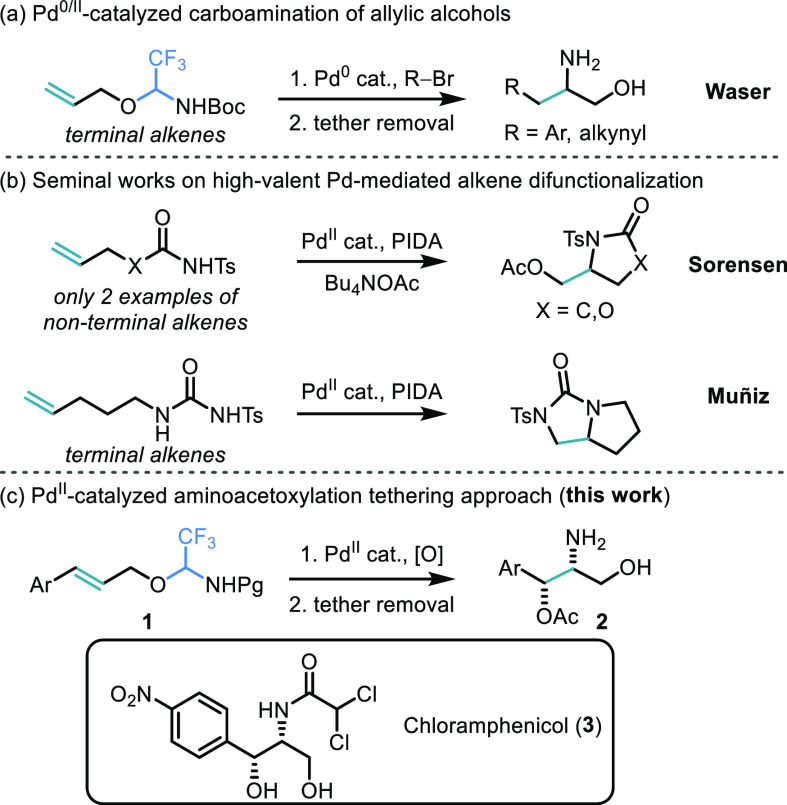
Pd-Catalyzed Intramolecular Olefin Difunctionalization Strategies

In 2005, Sorensen and Muñiz described the first Pd^II^/Pd^IV^-catalyzed intramolecular aminoacetoxylation and diamination processes, respectively (Scheme 1b).^10,11^ Notwithstanding these advances, the reactions were mostly limited to terminal olefins, and cleavage of the obtained carbamate/urea products was difficult. Following these seminal reports, the exploration of Pd^II^ catalysis under oxidative conditions for the simultaneous introduction of two carbon-heteroatom bonds across an unsaturated system has received signification attention.^12^ In view of the efficiency demonstrated by these methods, the exploitation of a Pd^II/IV^ catalytic cycle to further expand our tethering strategy beyond previously established Pd^0/II^ routes was considered. Inspired by the seminal work of Sorensen, we decided to study the aminoacetoxylation of internal olefins in combination with a trifluoroacetaldehyde-derived tether. Herein, we wish to report the successful implementation of this strategy to access substituted vicinal amino alcohols, which represent important building blocks commonly found in ligands and bioactive compounds (Scheme 1c).^13^ In particular, the 1-aryl-2-amino-propan-1,3-diol scaffold accessed in racemic form by our methodology can be found in chloroamphenicol (chloromycetin, **3**), an antibiotic extracted from a soil actinomycete in 1947, which is on the WHO list of essential medicines 2021.^14^

A preliminary evaluation of the oxyamination process was performed with cinnamyl-derived O–N tethered substrate **1a**, Pd(OAc)_2_ as catalyst, and the hypervalent iodine reagent (HIR) (diacetoxyiodo)benzene (PIDA) as oxidant (Table 1). The choice of this model system was based on the successful use of this tether in our previous work^8^ combined with the fact that a benzene ring had been the only alkene β substituent reported by Sorensen.^10^ Substrate **1a** can easily be obtained in one step from the corresponding allylic alcohol.^15^ Following optimization studies, compound **2a** was obtained in 88% NMR yield and 12:1 diastereomeric ratio (dr) employing 10 mol % of Pd(OAc)_2_ and 2 equiv of PIDA as oxidant in MeCN (entry 1). Using 5 mol % of catalyst or other palladium sources led to lower yields (entries 2–4). In contrast to Sorensen’s work,^10^ the addition of tetrabutylammonium acetate (TBAA) was not necessary to obtain a high yield and good diastereoselectivity (entry 5). In fact, it even led to a loss of diastereoselectivity. Addition of acetic acid also gave diminished yield and dr (entry 6). Heating to 50 °C was necessary to ensure high conversion (entry 7). The use of other oxidants was not appropriate for promoting aminoacetoxylation (entry 8). The relative configuration of major product **2a** was confirmed by single-crystal X-ray diffraction (see Scheme 3a), and the stereochemistry of the other compounds was assigned by analogy.

**Table 1:**
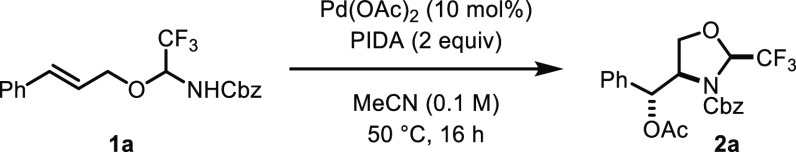
Optimization of the Pd-Catalyzed Oxyamination^a^

entry	deviations from above	**2a** (%), dr
1	none	88, 12:1
2	5 mol % of Pd(OAc)_2_	67, 12:1
3	10 mol % of Pd_2_(dba)_3_	80, 15:1
4	10 mol % of Pd(tfa)_2_	44, 2.5:1
5	1 equiv of TBAA	83, 1:1
6	4 equiv of AcOH	79, 9:1
7	room temperature	20, >20:1
8	AcOBX or PIFA as oxidant	0, –

a All reactions were performed on 0.1 mmol scale. ^1^H NMR yield based on trichloroethylene as internal standard. AcOBX = 1-acetoxy-1,2-benziodoxol-3(1*H*)-one.

With optimized conditions in hand, the scope of the aminoacetoxylation was investigated (Scheme 2). Model Product **2a** was isolated in 87% yield and 15:1 dr on a 0.2 mmol scale. It performed well also on a 1.5 mmol scale, affording product **2a** in 74% isolated yield and 15:1 dr. Different protecting groups on the nitrogen atom such as tosyl and Boc were well tolerated (**2b** and **2c**), although with variable diastereomeric ratios (1.5:1 and >20:1, respectively). Next, the effect of electronic variation on the aromatic ring was examined. Efficient reaction outcomes were obtained with electron-donating and electron-withdrawing functional groups in the *para* position (51–88%, **2d**–**2i**), although no product was observed with an amine substituent (**2j**).^16^ Other substitution patterns on the aromatic ring were investigated: difluoro substitution in the *meta* and *ortho* positions was tolerated with 77% and 56% yield, respectively (**2k** and **2l**). An *ortho*-methoxy functionality delivered product **2m** in 75% yield and greater than 20:1 dr. With a *meta* methyl-substituted substrate, the aminoacetoxylated compound **2n** was obtained in 69% yield and 9:1 dr. In order to explore the stereospecificity of the reaction, the *cis*-isomer of **1a** was subjected to the reaction conditions. A mixture of diastereisomers in 2.5:1 dr was observed by crude NMR analysis, and the major isomer **2a**′ was isolated in 18% yield.^17^ As another diastereoisomer was obtained as the major product, the reaction is indeed stereospecific, but unfortunately less efficient and selective for *cis* alkenes. Finally, a terminal olefin delivered product **2o** in 46% yield and 2:1 dr. To examine the generality of this transformation, the scope beyond cinnamyl-derived substrates was then investigated.^18^ Heteroaromatic substrates decomposed under the reaction conditions (**2p** and **2q**). These results could be attributed to the direct reaction of PIDA with electron-rich aromatics, which has been reported even in the absence of a metal catalyst.^19^ Additionally, poor conversion of the starting material was observed in the case of a pyridyl-substituted compound (**2r**) and trisubstituted alkenes.^18^

**Scheme 2:**
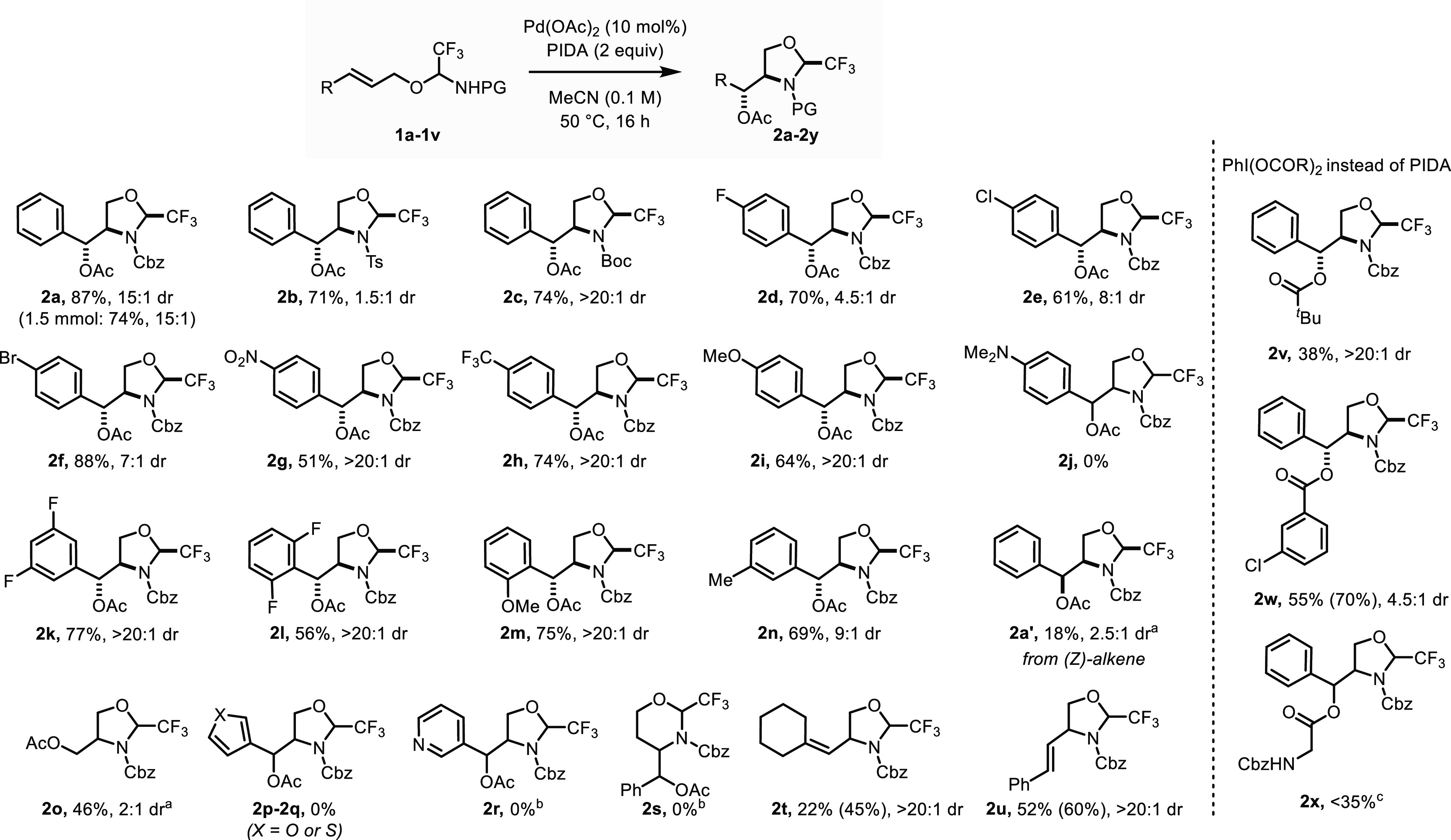
Scope of the Palladium-Catalyzed Tethered Aminoacetoxylation Reaction Combined isolated yields for minor and major diastereoisomers on a 0.2 mmol scale are given unless stated otherwise. ^1^H NMR yield based on trichloroethylene as internal standard is given in parentheses. Only the major isomer was isolated. No conversion of corresponding starting material was observed. NMR yield based on conversion between starting material and expected product **2x**.

In our work, we did not observe 6-*endo* aminoacetoxylated products, although such a process has been observed in the past employing specific ligands.^12e,20^ We decided nevertheless to test our reaction conditions with a homocinnamyl-derived substrate. No six-membered ring product **2s** was obtained, and the starting material was fully recovered. Next, we investigated aliphatic substituents on the alkene. With a cyclohexyl group, the aminoacetoxylated product was not formed. However, compound **2t** was isolated in 22% yield and greater than 20:1 dr. A β-hydride elimination step from an alkyl Pd^II^-intermediate following the aminopalladation process would account for this observation.^12a,12b^ In order to confirm that the β-hydride elimination was favored, the transformation was performed with a substrate similar to model **1a** with an extra methylene group between the alkene and benzene ring. Indeed, exclusive formation of elimination product **2u** was observed (52% yield and >20:1 dr). Even if the oxyamination was not successful, these Heck-like cyclization products also represent valuable building blocks bearing a versatile alkene.^21^

Inspired by a recent work by Beccalli and co-workers,^12g,22^ a series of hypervalent iodine reagents (PhI(OCOR)_2_) other than commercially available PhI(OAc)_2_ was investigated as both oxidant and carboxylate source. A lower reactivity was observed when PIDA was replaced with 2 equiv of bis(*tert*-butylcarbonyloxy)iodobenzene, affording the corresponding pivalate compound **2v** in 38% yield as a single diastereoisomer.^23^ A good conversion was achieved with PhI(*m*cpba)_2_ providing **2w** in 55% isolated yield and 4.5:1 dr. A more elaborate *N*-Cbz-Gly-based reagent showed ∼30% conversion toward the desired product **2x**. The use of other HIRs for the introduction of halides (e.g., F and Cl) was not successful.

To demonstrate the synthetic utility of the present methodology for the generation of functionalized β-amino alcohols, we next turned our attention to tether removal. *N*-Cbz-protected compound **2a** was stable under acidic hydrolysis conditions. Therefore, we decided to remove the Cbz group first (Scheme 3a). Compound **2a** was subjected to hydrogenation conditions followed by cleavage of the trifluoroacetaldehyde-derived tether under mild acidic conditions to afford amino alcohol **4a** in 73% yield over two steps. A short reaction time for the heterogeneous hydrogenation step employing Pearlman’s catalyst was required in order to avoid undesired hydrogenation of the acetate group after full conversion of the starting material. Notably, compound **4a** is an intermediate in the total synthesis of the antibiotic chloroamphenicol (**3**).^24^ The deprotected intermediate **5a**, isolated in 88% yield, could be recrystallized to determine the relative stereochemical configuration.^25^ In an effort to elucidate the structure of the minor diastereoisomer formed in the reaction, compound **2d** was subjected to the same sequential procedure to give the corresponding amino alcohol (Scheme 3b). This substrate was chosen as it could be isolated with a diastereomeric ratio of 4.2:1 on a 0.5 mmol scale. Hydrogenation to remove the Cbz group afforded intermediate **5d** with a comparable 4.3:1 dr. Tether cleavage under acidic conditions delivered product **4d** in 71% yield as an 8:1 inseparable mixture of diastereoisomers. Despite the different diastereomeric ratio, the observation of two products would suggest that the minor diastereoisomer has the same configuration at the center next to the CF_3_ group, as no epimerization had been observed when **2a** was deprotected.

**Scheme 3:**
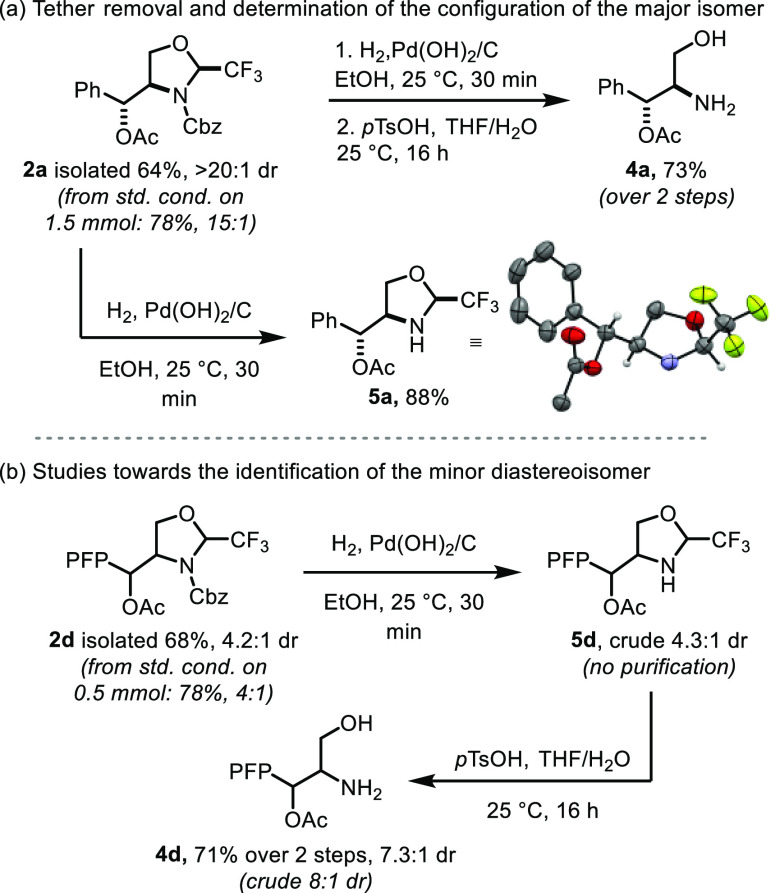
Tether Removal and Structure Determination See the Supporting Information for experimental details. PFP = *p*-fluorophenyl.

From a mechanistic viewpoint, the observed stereochemical outcome could be attributed to a first step involving *cis*-aminopalladation of the alkene followed by PIDA-mediated oxidation of the alkyl-Pd^II^ species generating a Pd^IV^ intermediate, which would give the desired compound through reductive elimination (See Scheme S1 in section G of the Supporting Information for a speculative catalytic cycle). Alternatively, a *trans*-aminopalladation followed by an S_N_2-type displacement of the generated high-valent Pd intermediate by an acetate would also lead to the same outcome.^12a^

In conclusion, a procedure for the generation of synthetically useful 1,2-amino alcohols has been developed. The transformation is based on an approach combining tethering chemistry and high-valent palladium catalysis for the diastereoselective construction of functionalized building blocks via an oxyamination process and subsequent removal of the trifluoroacetaldehyde-derived tether. Our work highlights that the formation of high-valent Pd^IV^ species for the construction of carbon-heteroatom bonds is compatible with aldehyde-based tethering strategies, setting the basis for the future development of highly selective alkene functionalizations.

## Supporting Information Available

Experimental procedures and characterization data for all new compounds; copy of NMR spectra (PDF)

## Accession Codes

CCDC 2173505 contains the supplementary crystallographic data for this paper. These data can be obtained free of charge via www.ccdc.cam.ac.uk/data_request/cif, or by emailing data_request@ccdc.cam.ac.uk, or by contacting The Cambridge Crystallographic Data Centre, 12 Union Road, Cambridge CB2 1EZ, UK; fax: +44 1223 336033.

## Notes

Raw data for NMR, MS, and IR is available free of charge from zenodo.org: DOI: 10.5281/zenodo.6786359.
