# Supplementary material for: Pd(II)-Catalyzed Aminoacetoxylation of Alkenes Via Tether Formation
Source: Org Lett. 2022 Jul 11;24(28):5068–72. doi: 10.1021/acs.orglett.2c01838 (PMC9490825; doi:10.1021/acs.orglett.2c01838)

Supporting Information

for

"Pd(II)-Catalyzed Aminoacetoxylation of Alkenes via Tether Formation"

Thomas Rossolini[†a], Ashis Das[†a], Stefano Nicolai[a], and Jerome Waser*[a]

[a] Laboratory of Catalysis and Organic Synthesis, Ecole Polytechnique Fédérale de Lausanne, EPFL, SB ISIC LCSO, BCH 1402, 1015 Lausanne (Switzerland)

†These authors contributed equally to this work.

*Correspondence to: jerome.waser@epfl.ch

Table of Contents

A. General Information 3

B. Synthesis of the Starting Materials 4

B.1. Synthesis of the Tether Precursors 4

B.2. Synthesis of HIR 7

B.3. Synthesis of tether substrates - General Procedure 1 (GP1) 8

C. Amino oxygenation of alkenes 18

C.1. General Procedure for the Amino oxygenation of alkenes (GP2) 18

C.2. Characterization of Amino oxygenation products 19

D. Additional not Successful Substrates (see starting materials 8a-8e) 26

E. Tether Removal 27

F. X-Ray Crystallographic Data 29

F.1. Single Crystal X-Ray Diffraction for compound 5a 29

G. Proposed Reaction Mechanism 31

H. NMR Spectra 32

A. General Information

The NMR spectra were recorded on a Brucker DPX-400 spectrometer at 400 MHz for ^{1}H, 101 MHz for ^{13}C, 376 MHz for ^{19}F and 162 MHz for ^{31}P. The chemical shift (δ) for ^{1}H and ^{13}C are given in ppm relative to residual signals of the solvents (chloroform-d - 7.26 ppm ^{1}H NMR and 77.16 ppm ^{13}C NMR; methanol-d4 3.31 ppm ^{1}H NMR and 49.0 ppm ^{13}C NMR; dmso-d6 2.50 ppm ^{1}H NMR and 39.52 ppm ^{13}C NMR). Carbon spectra have been measured using broadband {^{1}H} decoupling. Coupling constants are given in Hertz. The following abbreviations are used to indicate the multiplicity: s, singlet; d, doublet; q, quartet; m, multiplet; bs, broad signal; app, apparent. Infrared spectra were recorded on a JASCO FT-IR B4100 spectrophotometer with an ATR PRO410-S and a ZnSe prisma and are reported as cm^{-1} (w = weak, m = medium, s = strong, br = broad). High resolution mass spectrometric measurements were performed by the mass spectrometry service of ISIC at the EPFL on a MICROMASS (ESI) Q-TOF Ultima API. The raw data obtained from the Q-TOF Waters instrument does not take into account the mass of the electron for the ion, the obtained raw data has been therefore corrected by removing the mass of the electron (5 mDa).

The diffraction data for crystal structures were collected by mass spectrometry service of ISIC at the EPFL at low temperature using Cu (323) or Mo (520) K_a radiation on a Rigaku SuperNova dual system in combination with Atlas type CCD detector. The data reduction and correction were carried out by *CrysAlis*^{Pro} (Rigaku Oxford Diffraction, release 1.171.40.68a, **2019**). The solutions and refinements were performed by *SHELXT*[1] and *SHELXL*[2], respectively. The crystal structures were refined using full-matrix least-squares based on F^2 with all non-H atoms defined in anisotropic manner. Hydrogen atoms were placed in calculated positions by means of the "riding" model. Yields of isolated products refer to materials of >95% purity as determined by ^{1}H NMR.

The authors are indebted to the team of the research support service of ISIC at EPFL, particularly to the NMR, X-Ray, and the High-Resolution Mass Spectrometry Units.

General Procedures. All reactions were set up under a nitrogen atmosphere in oven-dried glassware using standard Schlenk techniques, unless otherwise stated. Synthesis grade solvents were used as purchased; anhydrous solvents (THF, Et_2O, Toluene and DCM) were taken from a commercial SPS solvent dispenser (H_2O content < 10 ppm, *Karl-Fischer* titration). Chromatographic purification of products was accomplished using flash chromatography (FC) on SiliaFlash P60 silica gel (230 - 400 mesh) or using Biotage Isolera Spektra One with pre-packaged silica cartridges purchased from Buchi, models: Sepacore or GraceResolve (4 g, 12 g, 25 g, 40 g). For thin layer chromatography (TLC) analysis throughout this work, Pre-coated TLC sheets ALUGRAM® Xtra SIL G/UV_{254} were employed, using UV light as the visualizing agent and basic aqueous potassium permanganate ($KMnO_4$) or *p*-anisaldehyde stain solutions, and heat as developing agents. Organic solutions were concentrated under reduced pressure on a Büchi rotatory evaporator. For reaction requiring heating, heat source is provided by DrySyn heating blocks connected to hotplates with a probe to maintain constant heating.

Determination of ^{1}H NMR yield and diastereomeric ratio: An aliquot from the crude residue was dissolved in chloroform-d or acetonitrile-d3 to determine diastereomeric ratio and NMR yield using trichloroethylene as internal standard. The dr was determined by integrating the ^{1}H NMR signal of the characteristic alpha-proton to the acetate group (usually between 6.20 and 5.80 ppm in acetonitrile-d3).

Materials. Most of the starting materials used in this study are commercial and were purchased in the highest purity available from Sigma-Aldrich, Fluka, Alfa Aesar, Fluorochem, Enamine and used as received, without further purifications. Palladium(II)acetate was purchased from Fluorochem.

B. Synthesis of the Starting Materials

B.1. Synthesis of the Tether Precursors

Synthesis of 1-((*Tert*-butoxycarbonyl)amino)-2,2,2-trifluoroethyl acetate (6a)

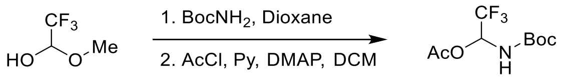

(step 1) Following a slightly modified procedure,[1] a 100 mL pressure tube was charged with tert-butyl carbamate (7.03 g, 60.0 mmol), 2,2,2-trifluoro-1-methoxyethanol (7.69 mL, 66.0 mmol, 1.1 equiv.), 4Å MS (10 g) and dioxane (80 mL). The tube was sealed under nitrogen atmosphere. The resulting mixture was heated at 100 °C for 5 d and then cooled down to rt. The mixture was filtered over Celite, and the cake was washed with ether (3x 20 mL). The volatiles were removed under reduced pressure and the resulting solid was recrystallized in chloroform to afford white crystals 5.10 (8.20 g, 38.1 mmol, 64% for 2 crops).

(step 2) To a solution of pyridine (1.84 mL, 22.8 mmol, 1.4 equiv.) and DMAP (50 mg, 0.41 mmol, 2.5 mol%) in dichloromethane (80 mL) at 0 °C was slowly added acetyl chloride (1.39 mL, 19.5 mmol, 1.2 equiv.). To the resulting mixture was added *tert*-butyl (2,2,2-trifluoro-1- hydroxyethyl) carbamate (3.50 g, 16.3 mmol) portion-wise. Then, the mixture was stirred at 0 °C for 20 min and quenched with water (10 mL). The pH was adjusted to 2 by addition of 0.1 N HCl and the layers were separated. The organic layer was washed with 0.1 N HCl (3x20 mL) and brine (30 mL), dried over $MgSO_4$, filtered, and concentrated under reduced pressure. The crude residue was purified by column chromatography (pentane: EtOAc 10:1) affording the title compound (4.05 g, 15.8 mmol, 97 % yield) as a white solid.

^{1}H NMR (400 MHz, Chloroform-*d*) δ 6.66 (bs, 1H), 5.25 (bs, 1H), 2.08 (s, 3H), 1.41 (s, 9H).

^{13}C{^{1}H} NMR (101 MHz, Chloroform-*d*) δ 168.0, 152.9, 123.1 (q, *J* = 281.3), 82.2, 72.0 (q, *J* = 39.1 Hz), 28.1, 20.5.

Spectral data was consistent with values reported in literature.[1]

Synthesis of 1-(((benzyloxy)carbonyl)amino)-2,2,2-trifluoroethyl acetate (6b)

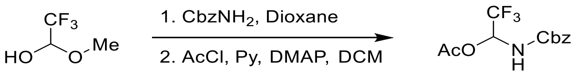

The Cbz-protected tether was prepared by following a similar protocol as for the Boc derivative.

1) Following a slightly modified procedure,[1] a 100 mL pressure tube was charged with benzyl carbamate (2.27 g, 15.0 mmol), 2,2,2-trifluoro-1-methoxyethanol (**5.1**) (1.59 mL, 16.5 mmol, 1.10 equiv.), 4Å MS (3 g) and dioxane (23 mL). The tube was sealed under nitrogen atmosphere. The resulting mixture was heated at 100 °C for 5 d and then cooled down to rt. The mixture was filtered over Celite, and the cake was washed with ether (3x5 mL). The volatiles were removed under reduced pressure and the resulting solid was recrystallized in chloroform to afford white crystals (1.87 g, 7.50 mmol, 50% for 2 crops).

2) To a solution of pyridine (1.14 mL, 14.0 mmol, 1.4 equiv.) and DMAP (30 mg, 0.25 mmol, 2.5 mol%) in dichloromethane (50 mL) at 0 °C was slowly added acetyl chloride (0.93 mL, 12 mmol, 1.2 equiv.). To the resulting mixture was added benzyl (2,2,2-trifluoro-1-hydroxyethyl)carbamate (2.50 g, 10.0 mmol, 1 equiv.) portion-wise. Then the mixture was stirred at 0 °C for 20 min and quenched with water (10 mL). The pH was adjusted to 2 by addition of 0.1 N HCl and the layers were separated. The organic layer was washed with 0.1 N HCl (3x20 mL) and brine (30 mL), dried over $MgSO_4$, filtered, and concentrated under reduced pressure. The crude residue was purified by column chromatography (Pentane: EtOAc 10:1) affording the title compound (2.40 g, 8.32 mmol, 83% yield) as a white solid.

^{1}H NMR (400 MHz, Chloroform-*d*) δ 7.44 – 7.29 (m, 5H), 6.80 (dd, *J* = 11.0, 5.6 Hz, 1H), 5.58 (s, 1H), 5.23 – 5.09 (m, 2H), 2.14 (s, 3H).

---

1 Orcel, U.; Waser, J. *Angew. Chem. Int. Ed*. **2016**, *55*, 12881−12885.

13C{1H} NMR (101 MHz, Chloroform-*d*) δ 168.1, 154.2, 135.3, 128.8 (2), 128.6, 121.7 (q, *J* = 281.0 Hz), 72.3 (q, *J* = 36.9 Hz), 68.3, 20.6.

19F NMR (376 MHz, Chloroform-*d*) δ -80.2.

IR (cm-1) 3031 (w), 1665 (w), 1503 (w), 1451 (w), 1293 (m), 1175 (s), 1153 (s).

HRMS (ESI/QTOF) m/z: $[M + H]^+$ Calculated for $C_{25}H_{23}F_3NO^+$ 410.1726; Found 410.1728.

Synthesis of N-(1-chloro-2,2,2-trifluoroethyl)-4-methylbenzenesulfonamide (6c)

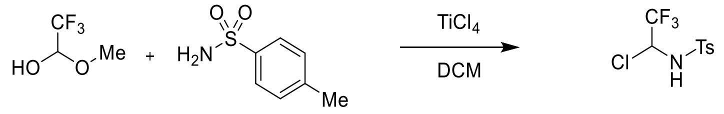

In a 100-mL two-necked round-bottomed flask, *p*-tosyl amide (8.56 g, 50.0 mmol, 1.0 equiv.) was suspended in DCM (dry, 86 mL). 2,2,2-Trifluoro-1-methoxyethanol (4.8 mL, 50 mmol, 1.0 equiv.) was then added at room temperature. Titanium tetrachloride (11.0 mL, 100 mmol, 2.0 equiv.) was slowly added to the suspension, resulting in a clear bright yellow solution. The latter was stirred at room temperature overnight, slowly becoming a yellow suspension. After 20 hours, the reaction was quenched through cautious and slow addition of water (20 mL) at 0 °C (attention: release of gas and fume!). The quenched mixture looked like a milky organic solution separated from a yellowish aqueous layer. The latter was extracted with DCM (3 x 100 mL). The combined organic layers were dried over $MgSO_4$, filtered and concentrated in vacuo to obtain an off-white solid (12.2 g, 42.3 mmol, 85%). The compound was used without further purification in the next step.

1H NMR (400 MHz, Chloroform-*d*) δ 7.81 (d, *J* = 8.0 Hz, 2H), δ 7.36 (d, *J* = 8.0 Hz, 2H), 6.16 (d, *J* = 10.0 Hz, 1H), 5.85 (dq, *J* = 10.0 Hz, 4.0 Hz, 1H), 2.46 (s, 3H).

Spectral data was consistent with the values reported in the literature.[2]

Synthesis of Alcohols:[3]

Procedure A

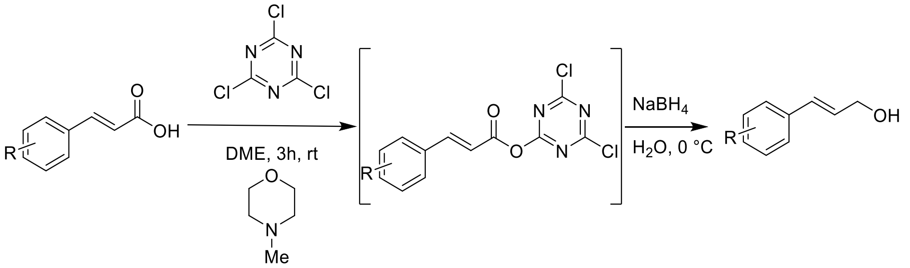

Alcohols

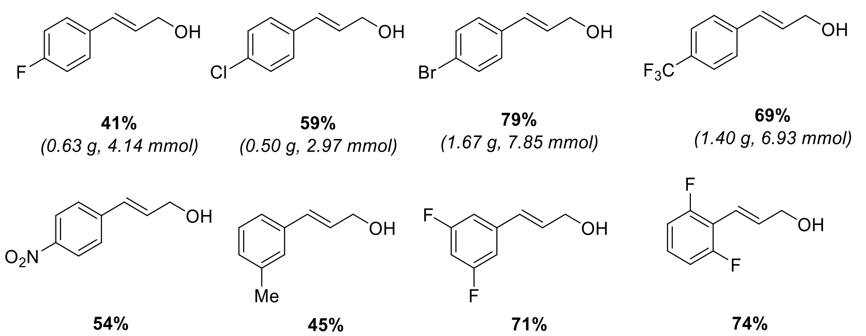

Procedure A: To a solution of cyanuric chloride (1 equiv.) dissolved in DME (3/4th commercial grade), N-methyl morpholine (1 equiv.) was added at room temperature under stirring. A white suspension was formed and to this mixture a solution cinnamic acid (1 equiv.) in 1/4th DME was added. After 3 h at room temperature the mixture

[2] Champalbert, J.; Guillois, A.; Jullien, J. ; Jullien, R. ; Lai, N.-T. ; Pascard, C. ; Prange, T. *Tetrahedron* **2011**, *18*, 3254−3259.

[3] All alcohols were directly submitted for the next step without further purification.

was filtered (Buchner funnel). The flask was cooled to 0 °C and $NaBH_4$ (1.5 equiv.) dissolved in water was added (attention: evolution of gas was observed during the addition). The mixture was stirred for additional 5 min at 0°C and diethyl ether was added. The solution was acidified (HCl 10% or $KHSO_4$ can be used depending on the nature of the substrate). The organic layer was separated and subsequently washed with a solution of Na_2CO_3 10% and brine. After drying over anhydrous Na_2SO_4 the solvent was evaporated to give the pure products.

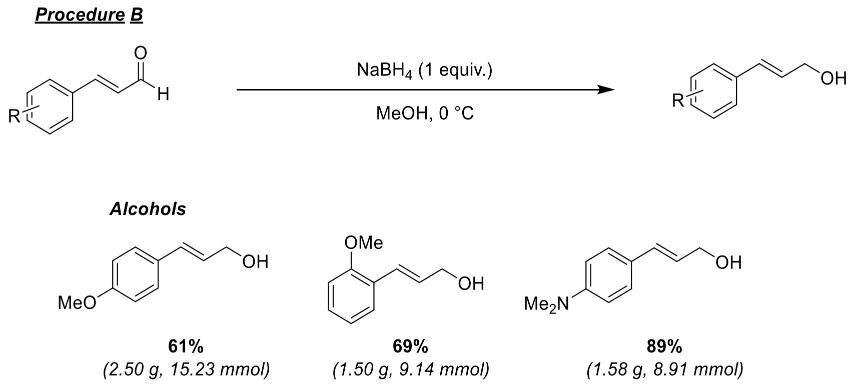

Procedure B: To a solution of 1.0 eq. of aldehyde in MeOH (1.5 mL/mmol) under Ar, 1.0 eq. of $NaBH_4$ was slowly added at 0 °C. The reaction was stirred for 30 min. and let it warmed up slowly to rt until completion (usually 12 hours). The reaction was quenched with saturated NH_4Cl solution (5.0 mL/mmol) and the aqueous layer was extracted with Et_2O (3 x 5.0 mL/mmol). The combined organic layers were washed with brine (5.0 mL/mmol), dried over anhydrous $MgSO_4$ and the solvent was removed under reduced pressure.

Procedure C:

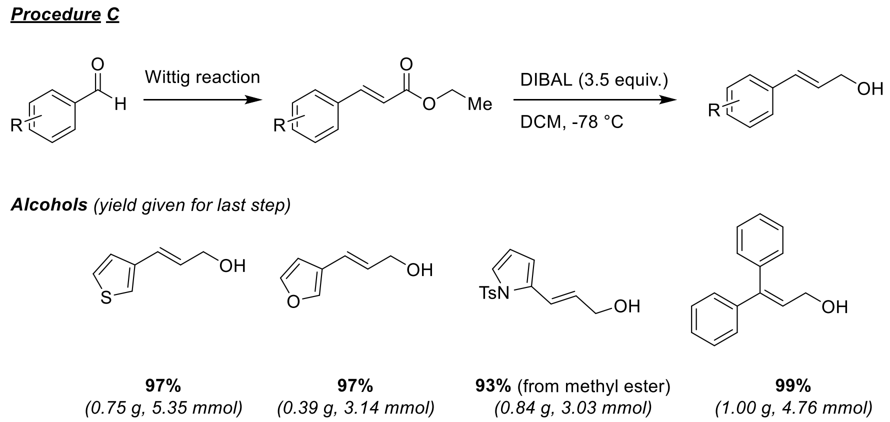

Conversion of carboxaldehydes to α,β-unsaturated esters:

Prepared in the manner of Travas-Sejdic, et al.[4] To a flame-dried 2-neck round-bottom flask with stir bar, condenser, septum, and nitrogen inlet, carboxaldehyde (1 equiv.), THF (0.17 M), and methyl(triphenylphosphoranylidene) acetate (1.5 equiv.) were added and the solution was heated to 50 °C in an oil bath. The reaction progress was monitored by TLC. The solvent was removed in vacuo and the crude material was purified by flash column chromatography.

Conversion of α,β-unsaturated esters to allylic alcohols:

To a flame-dried round-bottomed flask equipped with stir bar, septum, and nitrogen inlet, a solution of α,β-unsaturated esters (1 equiv.) in DCM (0.02 M), was added via syringe. The solution was cooled to -78 °C in a dry ice/acetone bath. Diisobutylaluminum hydride (DIBAL, 1.0 M in toluene unless otherwise specified, 3.5 equiv.) was added dropwise via syringe and the reaction mixture was stirred for 1 h. The reaction was monitored by TLC. Half-saturated sodium potassium tartrate solution was added and the mixture was vigorously stirred for approximately 16 h at rt before adding to a separatory funnel. The organic layer was separated and the aqueous

[4] Peng, H.; Soeller, C.; Travas-Sejdic, J. *Macromolecules* **2007**, *40*, 909−914.

layer was extracted with ether (2x). The combined organic layer was washed with deionized water (1x) and brine (1x), then dried over magnesium sulfate, gravity filtered, and concentrated under reduced pressure. The crude product was purified by silica gel flash column chromatography.

B.2. Synthesis of HIR

Phenyl-λ3-iodanediyl bis(3-chlorobenzoate) (7a)

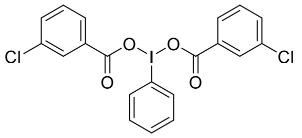

Prepared according to a modified literature procedure.[5] In a round-bottom flask, $PhI(OAc)_2$ (1.55 mmol, 1.0 equiv.) and the corresponding acid (3.11 mmol, 2 equiv.) were dissolved in xylene (mixture, 0.2 M) and the flask was heated to 55 °C under reduced pressure (about 10 mbar) using a diaphragm pump. When xylene was removed, the solids were filtered off (pentane as eluent), and dried under vacuum to give the desired product, which was obtained as white solid (727 mg, 1.41 mmol, 91%) and used in the next step without further purification. Data matched those reported in the literature.[6]

^{1}H NMR (400 MHz, Chloroform-*d*) δ 8.29 – 8.19 (m, 2H), 7.88 (t, *J* = 1.9 Hz, 2H), 7.82 (dt, *J* = 7.8, 1.4 Hz, 2H), 7.71 – 7.62 (m, 1H), 7.63 – 7.52 (m, 2H), 7.47 (ddd, *J* = 8.0, 2.2, 1.1 Hz, 2H), 7.31 (t, *J* = 7.9 Hz, 2H).

Phenyl-λ3-iodanediyl bis(2-(((benzyloxy)carbonyl)amino)acetate) (7b)

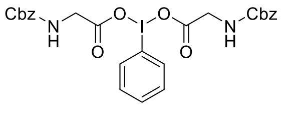

Prepared according to a modified literature procedure.[5] In a round-bottom flask, $PhI(OAc)_2$ (1.55 mmol, 1.0 equiv.) and the corresponding acid (3.11 mmol, 2 equiv.) were dissolved in xylene (mixture, 0.2 M) and the flask was heated to 55 °C under reduced pressure (about 10 mbar) using a diaphragm pump. When xylene was removed, solid were filtered off (pentane as eluent), and dried under vacuum to give the desired product which was obtained as white solid (775 mg, 1.25 mmol, 80%) and used in the next step without further purification.

^{1}H NMR (400 MHz, Chloroform-*d*) δ 8.10 – 8.03 (m, 2H), 7.62 (t, *J* = 7.5 Hz, 1H), 7.50 (t, *J* = 7.7 Hz, 2H), 7.38 – 7.30 (m, 10H), 5.10 (s, 4H), 3.90 (d, *J* = 5.4 Hz, 4H).

HRMS (ESI/QTOF) m/z: $[M + Na]^+$ Calcd for $C_{26}H_{25}IN_2NaO_8^+$ 643.0548; Found 643.0555.

[5] Giofrè, S.; Molteni, L.; Nava, D.; Lo Presti, L. ; Beccalli, E. M. *Angew. Chem. Int. Ed.* **2021**, *60,* 21723−21727.

[6] Koch, V.; Bräse, S. *Eur. J. Org. Chem.* **2021**, 3478−3483.

B.3. Synthesis of tether substrates - General Procedure 1 (GP1)

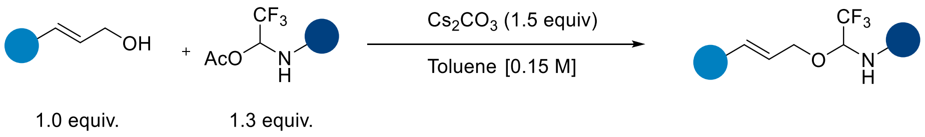

To a stirred solution of allyl alcohol (1.0 equiv.) in toluene [0.15 M] at room temperature was added Cs_2CO_3 (1.5 equiv.). Then, tether precursor (1.3 equiv.) and caesium carbonate (1.5 equiv.) were added and the resulting mixture was stirred for 12 h. After completion of the reaction according to TLC the reaction mixture was filtered through a plug of silica and eluted with ethyl acetate (as an alternative, an aqueous work up could be also performed). The filtrate was then evaporated under reduced pressure to give the title compound.

Benzyl (1-(cinnamyloxy)-2,2,2-trifluoroethyl)carbamate (1a)

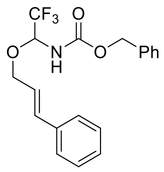

Prepared following GP1 from corresponding allyl alcohol (2.09 g, 15.6 mmol). Purification was performed on a Biotage flash column chromatography system with a 120 g cartridge (SiO_2, 10 – 40% EtOAc in pentane) to afford title compound as white solid (5.2 g, 14 mmol, 91% yield).

R_f value: 0.25 (20% Ethyl acetate in Pentane).

m.p.: 94 – 96 °C.

^{1}H NMR (400 MHz, Chloroform-d) δ 7.43 – 7.23 (m, 10H), 6.66 (d, *J* = 15.9 Hz, 1H), 6.25 (dt, *J* = 15.9, 6.4 Hz, 1H), 5.52 – 5.37 (m, 2H), 5.20 – 5.08 (m, 2H), 4.39 (dd, *J* = 12.6, 6.0 Hz, 1H), 4.31 (dd, *J* = 12.6, 6.7 Hz, 1H).

^{13}C NMR (101 MHz, $CDCl_3$) δ 155.5, 136.3, 135.5, 134.7, 128.8, 128.8, 128.7, 128.4, 128.3, 126.8, 123.6, 122.2 (q, *J* = 278.8 Hz), 78.5 (q, *J* = 35.0 Hz), 70.2, 68.0.

^{19}F NMR (376 MHz, Chloroform-*d*) δ -80.6.

IR (cm^{-1}) 3280 (m), 3034 (w), 1700 (s), 1537 (s), 1335 (m), 1254 (s), 1197 (s), 1157 (s), 1119 (s), 1057 (s), 965 (m), 749 (s), 701 (s).

HRMS (ESI/QTOF) m/z: $[M + Na]^+$ Calcd for $C_{19}H_{18}F_3NNaO_3^+$ 388.1131; Found 388.1124.

N-(1-(Cinnamyloxy)-2,2,2-trifluoroethyl)-4-methylbenzenesulfonamide (1b)

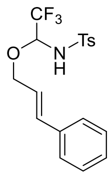

Prepared following GP1 from corresponding allyl alcohol (1.97 g, 14.7 mmol). Purification was performed on a Biotage flash column chromatography system with a 120 g cartridge (SiO_2, 10 – 40% EtOAc in pentane) to afford title compound as a white solid (2.5 g, 11 mmol, 75% yield).

R_f value: 0.26 (20% Ethyl acetate in Pentane).

m.p.: 92 – 93 °C.

^{1}H NMR (400 MHz, Chloroform-*d*) δ 7.79 – 7.73 (m, 2H), 7.43 – 7.23 (m, 7H), 6.63 (dd, *J* = 15.9, 1.5 Hz, 1H), 6.13 (ddd, *J* = 15.9, 7.1, 5.6 Hz, 1H), 5.48 (d, *J* = 10.2 Hz, 1H), 5.09 (dq, *J* = 9.2, 4.6 Hz, 1H), 4.40 (ddd, *J* = 12.5, 5.6, 1.5 Hz, 1H), 4.31 (ddd, *J* = 12.5, 7.1, 1.3 Hz, 1H), 2.41 (s, 3H).

^{13}C NMR (101 MHz, Chloroform-*d*) δ 144.4, 137.7, 136.2, 135.2, 130.0, 128.8, 128.3, 127.0, 126.8, 123.0, 122.0 (q, *J* = 282.7 Hz), 80.2 (q, *J* = 35.3 Hz), 69.8, 21.7.

^{19}F NMR (376 MHz, Chloroform-*d*) δ -80.3.

IR (cm^{-1}) 3258 (w), 2925 (w), 1600 (w), 1451 (w), 1338 (m), 1275 (m), 1189 (s), 1157 (s), 1070 (s), 967 (m), 914 (m), 814 (m), 749 (m), 666 (s).

HRMS (ESI/QTOF) m/z: $[M + Na]^+$ Calcd for $C_{18}H_{18}F_3NNaO_3S^+$ 408.0852; Found 408.0855.

Tert-butyl (1-(cinnamyloxy)-2,2,2-trifluoroethyl)carbamate (1c)

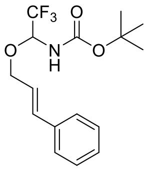

Prepared following GP1 from corresponding allyl alcohol (0.27 g, 2.0 mmol). Purification was performed on a Biotage flash column chromatography system with a 25 g cartridge (SiO_2, 10 – 40% EtOAc in pentane) to afford title compound as white solid (550 mg, 1.66 mmol, 83% yield).

R_f value: 0.38 (20% Ethyl acetate in Pentane).

m.p.: 82 – 84°C.

^{1}H NMR (400 MHz, Chloroform-d) δ 7.44 – 7.37 (m, 2H), 7.36 – 7.29 (m, 2H), 7.28 – 7.22 (m, 1H), 6.66 (dd, *J* = 15.9, 1.5 Hz, 1H), 6.26 (ddd, *J* = 15.9, 6.7, 5.8 Hz, 1H), 5.41 (dq, *J* = 9.9, 4.8 Hz, 1H), 5.20 (d, *J* = 10.9 Hz, 1H), 4.39 (ddd, *J* = 12.9, 5.7, 1.4 Hz, 1H), 4.34 – 4.24 (m, 1H), 1.48 (s, 9H).

^{13}C NMR (101 MHz, $CDCl_3$) δ 154.6, 136.4, 134.4, 128.7, 128.2, 126.8, 123.9, 122.4 (q, *J* = 281.7 Hz), 81.5, 78.1 (d, *J* = 34.8 Hz), 69.9, 28.3.

^{19}F NMR (376 MHz, Chloroform-*d*) δ -80.6.

IR (cm^{-1}) 3326 (w), 2981 (w), 1720 (m), 1503 (m), 1371 (m), 1053 (m), 968 (w), 890 (w), 745 (w), 695 (m).

HRMS (ESI/QTOF) m/z: $[M + Na]^+$ Calcd for $C_{16}H_{20}F_3NNaO_3^+$ 354.1287; Found 354.1286.

Benzyl (E)-(2,2,2-trifluoro-1-((3-(4-fluorophenyl)allyl)oxy)ethyl)carbamate (1d)

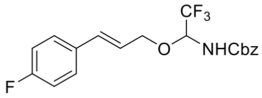

Prepared following GP1 from corresponding allyl alcohol (0.46 g, 3.0 mmol). Purification was performed on a Biotage flash column chromatography system with a 25 g cartridge (SiO_2, 10 – 40% EtOAc in pentane) to afford title compound as white solid (960 mg, 2.50 mmol, 83% yield).

R_f value: 0.39 (20% Ethyl acetate in Pentane).

m.p.: 94 – 96 °C.

^{1}H NMR (400 MHz, Chloroform-*d*) δ 7.43 – 7.31 (m, 7H), 7.09 – 6.97 (m, 2H), 6.61 (d, *J* = 15.9 Hz, 1H), 6.16 (dt, *J* = 15.9, 6.3 Hz, 1H), 5.57 – 5.38 (m, 2H), 5.14 (d, *J* = 2.7 Hz, 2H), 4.47 – 4.24 (m, 2H).

^{13}C NMR (101 MHz, Chloroform-*d*) δ 162.8 (d, *J* = 247.5 Hz), 155.5, 135.5, 133.6, 132.5 (d, *J* = 2.9 Hz), 128.8, 128.7, 128.4, 128.4 (d, *J* = 7.8 Hz), 123.3, 122.2 (q, *J* = 281.7 Hz), 115.7 (d, *J* = 21.6 Hz), 78.5 (q, *J* = 35.0 Hz), 70.1, 68.0.

^{19}F NMR (376 MHz, Chloroform-*d*) δ -80.6 (d, *J* = 4.6 Hz, 3F, C*F*$_3$), -113.6 (dd, *J* = 11.7, 6.4 Hz, 1F, Ar*F*).

IR (cm^{-1}) 3429 (w), 2256 (w), 1728 (w), 1512 (w), 1196 (w), 1049 (w), 906 (s), 729 (s).

HRMS (ESI/QTOF) m/z: $[M + Na]^+$ Calcd for $C_{19}H_{17}F_4NNaO_3^+$ 406.1037; Found 406.1035.

Benzyl (E)-(1-((3-(4-chlorophenyl)allyl)oxy)-2,2,2-trifluoroethyl)carbamate (1e)

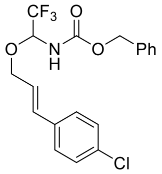

Prepared following GP1 from corresponding allyl alcohol (0.50 g, 3.0 mmol). Purification was performed on a Biotage flash column chromatography system with a 25 g cartridge (SiO_2, 10–40% EtOAc in pentane) to afford title compound as off-white solid (860 mg, 2.15 mmol, 72% yield).

R_f value: 0.35 (20% Ethyl acetate in Pentane).

m.p.: 129 – 131 °C.

^{1}H NMR (400 MHz, Chloroform-d) δ 7.43 – 7.25 (m, 9H), 6.60 (d, *J* = 15.9 Hz, 1H), 6.22 (dt, *J* = 15.9, 6.3 Hz, 1H), 5.46 (m, 2H), 5.24 – 5.08 (m, 2H), 4.46 – 4.34 (dd, *J* = 12.8, 1H), 4.30 (dd, *J* = 12.8, 6.6 Hz, 1H).

^{13}C NMR (101 MHz, $CDCl_3$) δ 155.5, 135.5, 134.8, 133.9, 133.3, 128.9, 128.8, 128.7, 128.4, 128.0, 124.3, 122.2 (q, *J* = 281.9 Hz), 78.6 (q, *J* = 35.1 Hz), 70.0, 68.0.

^{19}F NMR (376 MHz, Chloroform-*d*) δ -80.6.

IR (cm^{-1}) 3293 (m), 3036 (w), 1701 (s), 1534 (m), 1339 (m), 1279 (s), 1192 (s), 1158 (s), 1051 (s), 968 (m), 745 (m), 698 (s).

HRMS (ESI/QTOF) m/z: $[M + Na]^+$ Calcd for $C_{19}H_{17}ClF_3NNaO_3^+$ 422.0741; Found 422.0736.

Benzyl (E)-(1-((3-(4-bromophenyl)allyl)oxy)-2,2,2-trifluoroethyl)carbamate (1f)

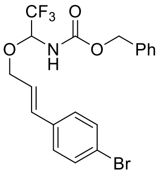

Prepared following GP1 from corresponding allyl alcohol (0.64 g, 3.0 mmol). Purification was performed on a Biotage flash column chromatography system with a 25 g cartridge (SiO_2, 10–40% EtOAc in pentane) to afford title compound as white solid (1.23 g, 2.78 mmol, 93% yield).

R_f value: 0.38 (20% Ethyl acetate in Pentane).

m.p.: 146 – 147 °C.

^{1}H NMR (400 MHz, Chloroform-d) δ 7.50 – 7.42 (m, 2H), 7.36 (qdt, *J* = 7.3, 4.8, 2.4 Hz, 5H), 7.25 (d, *J* = 8.4 Hz, 2H), 6.59 (d, *J* = 15.9 Hz, 1H), 6.23 (dt, *J* = 15.8, 6.3 Hz, 1H), 5.50 – 5.37 (m, 2H), 5.19 – 5.08 (m, 2H), 4.37 (dd, *J* = 12.7, 5.9 Hz, 1H), 4.29 (dd, *J* = 12.7, 6.6 Hz, 1H).

^{13}C NMR (101 MHz, $CDCl_3$) δ 155.5, 135.5, 135.2, 133.2, 131.9, 128.8, 128.7, 128.4, 128.3, 124.5, 122.2 (q, *J* = 281.5 Hz), 122.1, 78.6 (q, *J* = 35.1 Hz), 70.0, 68.0.

^{19}F NMR (376 MHz, Chloroform-*d*) δ -80.6.

IR (cm^{-1}) 3295 (m), 1701 (s), 1536 (s), 1371 (w), 1279 (s), 1194 (s), 1161 (s), 1123 (s), 1055 (s), 969 (m), 743 (m), 701 (m).

HRMS (ESI/QTOF) m/z: $[M + Na]^+$ Calcd for $C_{19}H_{17}BrF_3NNaO_3^+$ 466.0236; Found 466.0234.

Benzyl (E)-(2,2,2-trifluoro-1-((3-(4-nitrophenyl)allyl)oxy)ethyl)carbamate (1g)

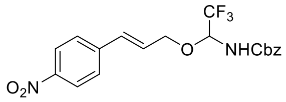

Prepared following GP1 from corresponding allyl alcohol (0.90 g, 5.0 mmol). Purification was performed on a Biotage flash column chromatography system (SiO_2, 10 – 40% EtOAc in pentane) to afford title compound as white solid (1.45 g, 3.53 mmol, 71% yield).

R_f value: 0.24 (20% Ethyl acetate in Pentane)

m.p.: 116 – 117 °C.

^{1}H NMR (400 MHz, Chloroform-*d*) δ 8.18 (d, *J* = 8.8 Hz, 2H), 7.51 (d, *J* = 8.5 Hz, 2H), 7.41 – 7.31 (m, 5H), 6.71 (d, *J* = 15.9 Hz, 1H), 6.41 (dt, *J* = 16.0, 5.8 Hz, 1H), 5.57 – 5.37 (m, 2H), 5.16 (d, *J* = 6.1 Hz, 2H), 4.52 – 4.39 (m, 1H), 4.39 – 4.29 (m, 1H).

^{13}C NMR (101 MHz, Chloroform-*d*) δ 155.6, 147.4, 142.7, 135.4, 135.3, 131.6, 128.8, 128.8, 128.3, 127.3, 124.2, 122.1 (q, *J* = 281.8 Hz), 78.8 (q, *J* = 34.5 Hz), 69.4, 68.1.

^{19}F NMR (376 MHz, Chloroform-*d*) δ -80.5 (d, *J* = 3.8 Hz).

IR (cm^{-1}) 3305 (w), 3039 (w), 2947 (w), 1724 (s), 1520 (s), 1342 (s), 1192 (s), 1157 (s), 1045 (s).

HRMS not found.

Benzyl (E)-(2,2,2-trifluoro-1-((3-(4-(trifluoromethyl)phenyl)allyl)oxy)ethyl)carbamate (1h)

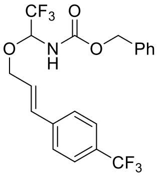

Prepared following GP1 from corresponding allyl alcohol (0.61 g, 3.0 mmol). Purification was performed on a Biotage flash column chromatography system with a 25 g cartridge (SiO_2, 10 – 40% EtOAc in pentane) to afford title compound as white solid (728 mg, 1.68 mmol, 56% yield).

R_f value: 0.34 (20% Ethyl acetate in Pentane).

m.p.: 112 – 114 °C.

^{1}H NMR (400 MHz, Chloroform-d) δ 7.57 (d, *J* = 8.1 Hz, 2H), 7.48 (d, *J* = 8.1 Hz, 2H), 7.42 – 7.30 (m, 5H), 6.68 (d, *J* = 16.0 Hz, 1H), 6.33 (dt, *J* = 15.9, 6.1 Hz, 1H), 5.51 – 5.40 (m, 2H), 5.20 – 5.09 (m, 2H), 4.41 (dd, *J* = 13.1, 5.8 Hz, 1H), 4.33 (dd, *J* = 13.1, 6.4 Hz, 1H).

^{13}C NMR (101 MHz, $CDCl_3$) δ 155.5z, 139.8, 135.4, 132.7, 130.0 (d, *J* = 32.6 Hz), 128.8, 128.8, 128.4, 127.0, 126.5, 125.7 (q, *J* = 3.8 Hz), 124.2 (q, *J* = 271.9 Hz), 122.1 (q, *J* = 281.6 Hz), 78.7 (q, *J* = 35.4 Hz), 69.7, 68.0.

^{19}F NMR (376 MHz, Chloroform-*d*) δ -62.6, -80.6.

IR (cm^{-1}) 3294 (w), 3040 (w), 1702 (m), 1533 (m), 1326 (s), 1190 (s), 1159 (s), 1119 (s), 1065 (s), 969 (m), 753 (m), 702 (m).

HRMS (ESI/QTOF) m/z: $[M + Na]^+$ Calcd for $C_{20}H_{17}F_6NNaO_3^+$ 456.1005; Found 456.1002.

Benzyl (E)-(2,2,2-trifluoro-1-((3-(4-methoxyphenyl)allyl)oxy)ethyl)carbamate (1i)

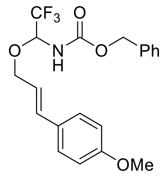

Prepared following GP1 from corresponding allyl alcohol (0.49 g, 3.0 mmol). Purification was performed on a Biotage flash column chromatography system with a 25 g cartridge (SiO_2, 10 – 40% EtOAc in pentane) to afford title compound as amorphous solid (850 mg, 2.15 mmol, 72% yield).

R_f value: 0.38 (20% Ethyl acetate in Pentane).

m.p.: 89 – 90 °C.

^{1}H NMR (400 MHz, Chloroform-d) δ 7.35 (tq, *J* = 5.9, 3.6 Hz, 7H), 6.90 – 6.82 (m, 2H), 6.60 (d, *J* = 15.8 Hz, 1H), 6.11 (dt, *J* = 15.8, 6.5 Hz, 1H), 5.52 – 5.38 (m, 2H), 5.14 (d, *J* = 5.0 Hz, 2H), 4.37 (dd, *J* = 12.3, 6.2 Hz, 1H), 4.28 (dd, *J* = 12.4, 6.9 Hz, 1H), 3.81 (s, 3H).

^{13}C NMR (101 MHz, $CDCl_3$) δ 159.8, 155.5, 135.5, 134.5, 129.0, 128.8, 128.7, 128.4, 128.1, 122.2 (q, *J* = 281.5 Hz), 121.2, 114.1, 78.4 (q, *J* = 35.0 Hz), 70.4, 67.9, 55.4.

^{19}F NMR (376 MHz, Chloroform-*d*) δ -80.6.

IR (cm^{-1}) 3312 (w), 2956 (w), 1717 (s), 1608 (m), 1512 (s), 1246 (s), 1189 (s), 1158 (s), 1041 (s), 969 (m), 753 (m), 698 (s).

HRMS (ESI/QTOF) m/z: $[M + Na]^+$ Calcd for $C_{20}H_{20}F_3NNaO_4^+$ 418.1237; Found 418.1239.

Benzyl (E)-(1-((3-(4-(dimethylamino)phenyl)allyl)oxy)-2,2,2-trifluoroethyl)carbamate (1j)

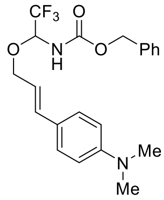

Prepared following GP1 from corresponding allyl alcohol (0.53 g, 3.0 mmol). Purification was performed on a Biotage flash column chromatography system with a 25 g cartridge (SiO_2, 10 – 40% EtOAc in pentane) to afford title compound as amorphous solid (960 mg, 2.35 mmol, 78% yield).

R_f value: 0.34 (20% Ethyl acetate in Pentane).

m.p.: 134 – 136 °C.

^{1}H NMR (400 MHz, Chloroform-d) δ 7.42 – 7.26 (m, 7H), 6.71 – 6.63 (m, 2H), 6.57 (d, *J* = 15.8 Hz, 1H), 6.04 (dt, *J* = 15.8, 6.8 Hz, 1H), 5.48 (dd, *J* = 10.2, 5.0 Hz, 1H), 5.38 (d, *J* = 10.4 Hz, 1H), 5.21 – 5.07 (m, 2H), 4.36 (dd, *J* = 12.1, 6.3 Hz, 1H), 4.27 (dd, *J* = 12.1, 7.1 Hz, 1H), 2.97 (s, 6H).

^{13}C NMR (101 MHz, $CDCl_3$) δ 155.5, 150.6, 135.6, 135.5, 128.8, 128.6, 128.4, 127.9, 124.6, 122.3 (q, *J* = 281.4 Hz), 118.8, 112.4, 78.2 (q, *J* = 35.3 Hz), 70.9, 67.9, 40.6.

^{19}F NMR (376 MHz, Chloroform-*d*) δ -80.7.

IR (cm^{-1}) 3328 (w), 2926 (w), 1721 (s), 1610 (s), 1522 (s), 1240 (s), 1185 (s), 1154 (s), 1043 (s), 741 (m), 697 (m).

HRMS (ESI/QTOF) m/z: $[M + H]^+$ Calcd for $C_{21}H_{24}F_3N_2O_3^+$ 409.1734; Found 409.1732.

Benzyl (E)-(1-((3-(3,5-difluorophenyl)allyl)oxy)-2,2,2-trifluoroethyl)carbamate (1k)

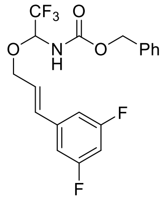

Prepared following GP1 from corresponding allyl alcohol (0.51 g, 3.0 mmol). Purification was performed on a Biotage flash column chromatography system with a 25 g cartridge (SiO_2, 10 – 40% EtOAc in pentane) to afford title compound as white solid (960 mg, 2.39 mmol, 80% yield).

R_f value: 0.36 (20% Ethyl acetate in Pentane).

m.p.: 92 – 94 °C.

^{1}H NMR (400 MHz, Chloroform-d) δ 7.43 – 7.29 (m, 5H), 6.89 (d, *J* = 7.4 Hz, 2H), 6.71 (tt, *J* = 8.8, 2.3 Hz, 1H), 6.56 (d, *J* = 15.9 Hz, 1H), 6.25 (dt, *J* = 15.9, 6.0 Hz, 1H), 5.44 (d, *J* = 5.0 Hz, 2H), 5.15 (d, *J* = 2.2 Hz, 2H), 4.39 (dd, *J* = 13.3, 5.6 Hz, 1H), 4.30 (dd, *J* = 13.2, 6.4 Hz, 1H).

^{13}C NMR (101 MHz, $CDCl_3$) δ 163.3 (dd, *J* = 247.9, 13.0 Hz), 155.5, 139.7 (t, *J* = 9.5 Hz), 135.4, 132.0, 128.8, 128.8, 128.4, 126.6, 122.1 (q, *J* = 281.6 Hz), 110.4 – 108.7 (m), 103.4 (t, *J* = 25.5 Hz), 78.7 (q, *J* = 35.1 Hz), 69.4, 68.1.

^{19}F NMR (376 MHz, Chloroform-*d*) δ -80.6, -110.0.

IR (cm^{-1}) 3318 (w), 2925 (w), 1720 (s), 1591 (m), 1516 (m), 1453 (m), 1333 (m), 1279 (s), 1236 (s), 1193 (s), 1160 (s), 1117 (s), 1044 (s), 985 (s), 674 (w).

HRMS (ESI/QTOF) m/z: $[M + Na]^+$ Calcd for $C_{19}H_{16}F_5NNaO_3^+$ 424.0943; Found 424.0938.

Benzyl (E)-(1-((3-(2,6-difluorophenyl)allyl)oxy)-2,2,2-trifluoroethyl)carbamate (1l)

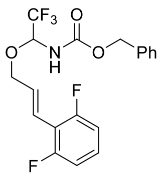

Prepared following GP1 from corresponding allyl alcohol (0.51 g, 3.0 mmol). Purification was performed on a Biotage flash column chromatography system with a 25 g cartridge (SiO_2, 10 – 40% EtOAc in pentane) to afford title compound as amorphous solid (640 mg, 1.59 mmol, 53% yield).

R_f value: 0.38 (20% Ethyl acetate in Pentane).

m.p.: 120 – 122 °C.

^{1}H NMR (400 MHz, Chloroform-d) δ 7.35 (h, *J* = 5.2 Hz, 5H), 7.17 (tt, *J* = 8.4, 6.2 Hz, 1H), 6.94 – 6.80 (m, 2H), 6.69 (d, *J* = 16.4 Hz, 1H), 6.57 (dt, *J* = 16.4, 5.8 Hz, 1H), 5.54 – 5.39 (m, 2H), 5.16 (d, *J* = 2.2 Hz, 2H), 4.42 (dd, *J* = 13.0, 5.5 Hz, 1H), 4.33 (dd, *J* = 13.0, 6.1 Hz, 1H).

^{13}C NMR (101 MHz, $CDCl_3$) δ 161.1 (dd, *J* = 252.0, 7.5 Hz), 155.5, 135.5, 130.8 (t, *J* = 7.8 Hz), 128.9, 128.8, 128.7, 128.4, 122.2 (q, *J* = 281.5 Hz), 120.3, 113.7 (t, *J* = 15.2 Hz), 112.07 – 111.2 (m), 78.8 (q, *J* = 35.2 Hz), 70.7, 68.0.

^{19}F NMR (376 MHz, Chloroform-*d*) δ -80.7, -113.0.

IR (cm^{-1}) 3269 (m), 3038 (w), 1699 (s), 1539 (m), 1465 (m), 1254 (s), 1197 (s), 1153 (s), 1124 (m), 1059 (s), 987 (s), 781 (m), 701 (s).

HRMS (ESI/QTOF) m/z: $[M + Na]^+$ Calcd for $C_{19}H_{16}F_5NNaO_3^+$ 424.0943; Found 424.0947.

Benzyl (E)-(2,2,2-trifluoro-1-((3-(2-methoxyphenyl)allyl)oxy)ethyl)carbamate (1m)

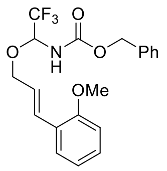

Prepared following GP1 from corresponding allyl alcohol (0.49 g, 3.0 mmol). Purification was performed on a Biotage flash column chromatography system with a 25 g cartridge (SiO_2, 10 – 40% EtOAc in pentane) to afford title compound as white solid (760 mg, 1.92 mmol, 64% yield).

R_f value: 0.39 (20% Ethyl acetate in Pentane).

m.p.: 92 – 94 °C.

^{1}H NMR (400 MHz, Chloroform-d) δ 7.43 (d, *J* = 7.6 Hz, 1H), 7.36 (q, *J* = 6.4 Hz, 5H), 7.30 – 7.21 (m, 1H), 7.02 – 6.90 (m, 2H), 6.88 (dd, *J* = 8.3, 1.1 Hz, 1H), 6.29 (dt, *J* = 16.0, 6.4 Hz, 1H), 5.49 (dq, *J* = 9.6, 4.7 Hz, 1H), 5.41 (d, *J* = 10.5 Hz, 1H), 5.21 – 5.09 (m, 2H), 4.40 (dd, *J* = 12.3, 6.2 Hz, 1H), 4.32 (dd, *J* = 12.4, 6.7 Hz, 1H), 3.85 (s, 3H).

^{13}C NMR (101 MHz, $CDCl_3$) δ 157.1, 155.5, 135.6, 129.7, 129.3, 128.8, 128.6, 128.4, 127.4, 125.3, 124.3, 122.3 (q, *J* = 281.4 Hz), 120.8, 111.0, 78.6 (q, *J* = 35.1 Hz), 70.9, 67.9, 55.6.

^{19}F NMR (376 MHz, Chloroform-*d*) δ -80.7.

IR (cm^{-1}) 3310 (w), 2944 (w), 1716 (s), 1526 (m), 1281 (s), 1191 (s), 1160 (s), 1048 (s), 976 (m), 752 (s), 700 (m).

HRMS (ESI/QTOF) m/z: $[M + Na]^+$ Calcd for $C_{20}H_{20}F_3NNaO_4^+$ 418.1237; Found 418.1239.

Benzyl (E)-(2,2,2-trifluoro-1-((3-(m-tolyl)allyl)oxy)ethyl)carbamate (1n)

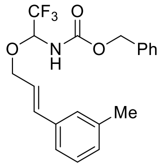

Prepared following GP1 from corresponding allyl alcohol (0.45 g, 3.0 mmol). Purification was performed on a Biotage flash column chromatography system with a 25 g cartridge (SiO_2, 10 – 40% EtOAc in pentane) to afford title compound as amorphous solid (560 mg, 1.48 mmol, 49% yield).

R_f value: 0.39 (20% Ethyl acetate in Pentane).

m.p.: 91 – 92 °C.

^{1}H NMR (400 MHz, Chloroform-d) δ 7.35 (m, 5H), 7.26 – 7.15 (m, 3H), 7.13 – 7.05 (m, 1H), 6.63 (d, *J* = 15.9 Hz, 1H), 6.24 (dt, *J* = 15.8, 6.4 Hz, 1H), 5.48 (dq, *J* = 9.4, 4.6 Hz, 1H), 5.40 (d, *J* = 10.6 Hz, 1H), 5.20 – 5.08 (m, 2H), 4.38 (dd, *J* = 12.5, 6.0 Hz, 1H), 4.30 (dd, *J* = 12.6, 6.7 Hz, 1H), 2.35 (s, 3H).

^{13}C NMR (101 MHz, $CDCl_3$) δ 155.5, 138.3, 136.2, 135.5, 134.9, 129.1, 128.8, 128.7, 128.6, 128.4, 127.6, 124.0, 123.4, 122.2 (q, *J* = 281.3 Hz), 78.5 (q, *J* = 35.1 Hz), 70.2, 68.0, 21.5.

^{19}F NMR (376 MHz, Chloroform-*d*) δ -80.6.

IR (cm^{-1}) 3319 (w), 3035 (w), 1715 (s), 1514 (m), 1279 (m), 1234 (s), 1190 (s), 1158 (s), 1044 (s), 969 (m), 775 (m), 697 (s).

HRMS (ESI/QTOF) m/z: $[M + Na]^+$ Calcd for $C_{20}H_{20}F_3NNaO_3^+$ 402.1287; Found 402.1284.

Benzyl (Z)-(2,2,2-trifluoro-1-((3-phenylallyl)oxy)ethyl)carbamate (1a')

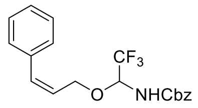

Prepared following GP1 from corresponding allyl alcohol (2.7 g, 20 mmol). Purification was performed on a Biotage flash column chromatography system (SiO_2, 10 – 40% EtOAc in pentane) to afford title compound as white solid (7.34 g, 20.1 mmol, quant.).

R_f value: 0.29 (20% Ethyl acetate in Pentane).

m.p.: 73 – 75 °C.

^{1}H NMR (400 MHz, Chloroform-*d*) δ 7.42 – 7.26 (m, 8H), 7.23 – 7.17 (m, 2H), 6.69 (d, *J* = 11.7 Hz, 1H), 5.82 (dt, *J* = 12.2, 6.5 Hz, 1H), 5.48 – 5.32 (m, 2H), 5.12 (s, 2H), 4.47 (dd, *J* = 12.3, 6.5, 1.6 Hz, 2H).

^{13}C NMR (101 MHz, Chloroform-*d*) δ 155.4, 136.2, 135.5, 133.6, 128.9, 128.8, 128.7, 128.5, 128.4, 127.7, 126.4, 122.2 (q, *J* = 281.6 Hz), 79.0 (q, *J* = 34.9 Hz), 67.9, 66.2.

^{19}F NMR (376 MHz, Chloroform-*d*) δ -80.7 (d, *J* = 5.6 Hz).

IR (cm^{-1}) 3313 (w), 3032 (w), 2951 (w), 1720 (s), 1523 (m), 1277 (m), 1234 (s), 1188 (s), 1157 (s).

HRMS (ESI/QTOF) m/z: [M + Na]$^+$ Calcd for $C_{19}H_{18}F_3NNaO_3^+$ 388.1131; Found 388.1142.

Benzyl (1-(allyloxy)-2,2,2-trifluoroethyl)carbamate (1o)

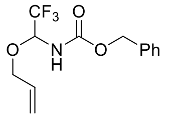

Prepared following GP1 from corresponding allyl alcohol (175 mg, 3.01 mmol). Purification was performed on a Biotage flash column chromatography system with a 25 g cartridge (SiO_2, 10 – 40% EtOAc in pentane) to afford title compound as white solid (507 mg, 1.75 mmol, 58% yield).

R_f value: 0.34 (20% Ethyl acetate in Pentane).

m.p.: 70 – 71 °C.

^{1}H NMR (400 MHz, Chloroform-d) δ 7.44 – 7.30 (m, 5H), 5.88 (ddt, *J* = 16.5, 11.0, 5.8 Hz, 1H), 5.47 – 5.29 (m, 3H), 5.29 – 5.22 (m, 1H), 5.17 (s, 2H), 4.23 (dd, *J* = 12.8, 5.3 Hz, 1H), 4.12 (dd, *J* = 12.8, 6.3 Hz, 1H).

^{13}C NMR (101 MHz, $CDCl_3$) δ 155.5, 135.6, 132.6, 128.8, 128.7, 128.4, 122.2 (q, *J* = 281.5 Hz), 119.3, 78.4 (q, *J* = 35.1 Hz), 70.2, 68.0.

^{19}F NMR (376 MHz, Chloroform-*d*) δ -80.8.

IR (cm^{-1}) 3307 (w), 3036 (w), 1714 (s), 1531 (s), 1336 (m), 1280 (s), 1237 (s), 1193 (s), 1159 (s), 1048 (s), 699 (s).

HRMS (ESI/QTOF) m/z: [M + Na]$^+$ Calcd for $C_{13}H_{14}F_3NNaO_3^+$ 312.0818; Found 312.0823.

Benzyl (E)-(2,2,2-trifluoro-1-((3-(furan-3-yl)allyl)oxy)ethyl)carbamate (1p)

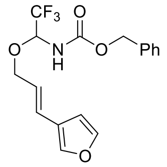

Prepared following GP1 from corresponding allyl alcohol (161 mg, 1.296 mmol). Purification was performed on a Biotage flash column chromatography system with a 25 g cartridge (SiO_2, 10 – 40% EtOAc in pentane) to afford title compound as white solid (343 mg, 0.674 mmol, 52% yield).

R_f value: 0.34 (20% Ethyl acetate in Pentane).

m.p.: 91 – 93 °C.

^{1}H NMR (400 MHz, Chloroform-d) δ 7.44 (s, 1H), 7.42 – 7.29 (m, 6H), 6.63 – 6.39 (m, 2H), 5.97 (dt, *J* = 14.8, 6.5 Hz, 1H), 5.53 – 5.31 (m, 2H), 5.15 (br s, 2H), 4.33 (dd, *J* = 12.5, 6.0 Hz, 1H), 4.24 (dd, *J* = 12.5, 7.0 Hz, 1H).

^{13}C NMR (101 MHz, $CDCl_3$) δ 155.6, 143.8, 141.3, 135.5, 128.8, 128.7, 128.4, 124.8, 123.4, 123.1, 122.2 (q, *J* = 281.5 Hz), 107.7, 78.4 (q, *J* = 35.1 Hz), 70.0, 68.0.

^{19}F NMR (376 MHz, Chloroform-*d*) δ -80.6.

IR (cm^{-1}) 3334 (w), 3035 (w), 1729 (m), 1522 (m), 1368 (m), 1154 (s), 1046 (s), 911 (m), 813 (w), 732 (s), 671 (s).

HRMS (ESI/QTOF) m/z: [M + Na]$^+$ Calcd for $C_{17}H_{16}F_3NNaO_4^+$ 378.0924; Found 378.0932.

Benzyl (E)-(2,2,2-trifluoro-1-((3-(thiophen-3-yl)allyl)oxy)ethyl)carbamate (1q)

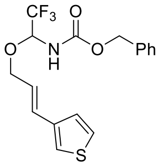

Prepared following GP1 from corresponding allyl alcohol (184 mg, 1.313 mmol). Purification was performed on a Biotage flash column chromatography system with a 25 g cartridge (SiO_2, 10 – 40% EtOAc in pentane) to afford title compound as amorphous solid (350 mg, 0.985 mmol, 75% yield).

R_f value: 0.23 (20% Ethyl acetate in Pentane).

^{1}H NMR (400 MHz, Chloroform-d) δ 7.35 (qd, *J* = 5.9, 2.8 Hz, 5H), 7.28 (ddd, *J* = 5.1, 2.9, 0.6 Hz, 1H), 7.21 (d, *J* = 5.7 Hz, 2H), 6.66 (d, *J* = 15.8 Hz, 1H), 6.09 (dt, *J* = 15.8, 6.5 Hz, 1H), 5.46 (dd, *J* = 10.2, 4.9 Hz, 1H), 5.37 (d, *J* = 10.4 Hz, 1H), 5.20 – 5.08 (m, 2H), 4.35 (dd, *J* = 12.5, 6.0 Hz, 1H), 4.27 (dd, *J* = 12.4, 6.9 Hz, 1H).

^{13}C NMR (101 MHz, $CDCl_3$) δ 155.5, 139.0, 135.5, 129.0, 128.8, 128.7, 128.4, 126.3, 125.2, 123.4, 123.3, 122.2 (q, *J* = 281.3 Hz), 78.4 (q, *J* = 35.1 Hz), 70.1, 68.0.

^{19}F NMR (376 MHz, Chloroform-*d*) δ -80.7.

IR (cm^{-1}) 3299 (w), 3035 (w), 1718 (s), 1533 (m), 1337 (m), 1280 (s), 1237 (s), 1194 (s), 1161 (s), 1050 (s), 968 (m), 773 (m), 698 (m).

HRMS (ESI/QTOF) m/z: $[M + Na]^+$ Calcd for $C_{17}H_{16}F_3NNaO_3S^+$ 394.0695; Found 394.0696.

Benzyl (E)-(2,2,2-trifluoro-1-((3-(pyridin-3-yl)allyl)oxy)ethyl)carbamate (1r)

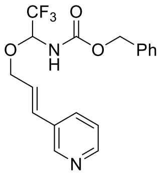

Prepared following GP1 from corresponding allyl alcohol (177 mg, 1.308 mmol). Purification was performed on a Biotage flash column chromatography system with a 25 g cartridge (SiO_2, 10 – 40% EtOAc in pentane) to afford title compound as solid (350 mg, 0.955 mmol, 73% yield).

R_f value: 0.36 (20% Ethyl acetate in Pentane).

m.p.: 77 – 79 °C.

^{1}H NMR (400 MHz, Chloroform-d) δ 8.60 (s, 1H), 8.49 (dd, *J* = 4.8, 1.6 Hz, 1H), 7.69 (dt, *J* = 8.0, 2.0 Hz, 1H), 7.42 – 7.29 (m, 5H), 7.27 – 7.21 (m, 1H), 6.63 (d, *J* = 16.0 Hz, 1H), 6.30 (dt, *J* = 16.0, 6.0 Hz, 1H), 5.73 (d, *J* = 10.4 Hz, 1H), 5.47 (dq, *J* = 9.9, 4.8 Hz, 1H), 5.21 – 5.09 (m, 2H), 4.46 – 4.36 (m, 1H), 4.36 – 4.26 (m, 1H).

^{13}C NMR (101 MHz, $CDCl_3$) δ 155.6, 149.2, 148.6, 135.5, 133.3, 132.0, 130.6, 128.8, 128.7, 128.4, 126.1, 123.6, 122.2 (q, *J* = 281.3 Hz), 78.7 (q, *J* = 35.1 Hz), 69.7, 68.0.

^{19}F NMR (376 MHz, Chloroform-*d*) δ -80.5.

IR (cm^{-1}) 3178 (w), 2925 (w), 2338 (w), 1725 (s), 1556 (m), 1281 (s), 1244 (s), 1187 (s), 1159 (s), 1044 (s), 970 (m), 740 (m), 702 (s).

HRMS (ESI/QTOF) m/z: $[M + H]^+$ Calcd for $C_{18}H_{18}F_3N_2O_3^+$ 367.1264; Found 367.1264.

Benzyl (E)-(2,2,2-trifluoro-1-((4-phenylbut-3-en-1-yl)oxy)ethyl)carbamate (1s)

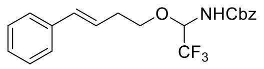

Prepared following GP1 from corresponding alcohol (2.98 g, 20.1 mmol). Purification was performed on a Biotage flash column chromatography system with a 250 g cartridge (SiO_2, 10 – 40% EtOAc in pentane) to afford title compound as white solid (7.92 g, 20.1 mmol, quant.).

R_f value: 0.36 (20% Ethyl acetate in Pentane).

m.p.: 95 – 97 °C.

^{1}H NMR (400 MHz, Chloroform-*d*) δ 7.42 – 7.28 (m, 9H), 7.26 – 7.20 (m, 1H), 6.47 (d, *J* = 15.8 Hz, 1H), 6.18 (dt, *J* = 15.8, 6.9 Hz, 1H), 5.65 – 5.31 (m, 2H), 5.17 (s, 2H), 3.93 – 3.65 (m, 2H), 2.61 – 2.41 (m, 2H).

^{13}C NMR (101 MHz, Chloroform-*d*) δ 155.7, 137.4, 135.5, 132.5, 128.8, 128.7, 128.7, 128.4, 127.4, 126.2, 125.7, 122.2 (q, *J* = 281.7 Hz), 79.5 (q, *J* = 35.1 Hz), 69.4, 67.9, 33.0.

^{19}F NMR (376 MHz, Chloroform-*d*) δ -80.7 (d, *J* = 4.4 Hz).

IR (cm^{-1}) 3311 (w), 3027 (w), 2950 (w), 1722 (s), 1530 (m), 1280 (s), 1240 (s), 1191 (s), 1161 (s).

HRMS (ESI/QTOF) m/z: $[M + Na]^+$ Calcd for $C_{20}H_{20}F_3NNaO_3^+$ 402.1287; Found 402.1283.

Benzyl (E)-(1-((3-cyclohexylallyl)oxy)-2,2,2-trifluoroethyl)carbamate (1t)

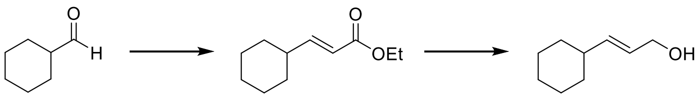

I II

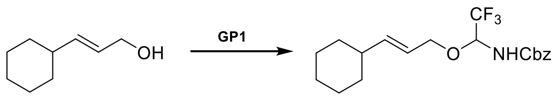

II 8t

Ester **I** and alcohol **II** were prepared according to adapted literature procedures.[7] NMR data of intermediate I matched those from reported literature, whereas alcohol intermediate **II** was used in the next step without further purification. For ester **I**: a 50 mL RBF under N_2 atmosphere was charged with DME (21 mL), cooled to 0 °C and NaH (60% mineral oil, 268 mg, 6.69 mmol, 1.5 eq.) was added. Triethyl phosphonoacetate (1.33 mL, 6.69 mmol, 1.5 eq.) was added dropwise to the suspension at 0°C and the resulting mixture was stirred at the same temperature for 30 min. Then, cyclohexanecarboxaldehyde (0.54 mL, 4.46 mmol, 1 eq.) was added to the reaction mixture at 0 °C. After complete addition, the reaction mixture was stirred at room temperature for 3 h. The reaction mixture was then diluted with sat. aq. NH_4Cl (75 mL), extracted with EtOAc (3 x 50 mL), washed with brine, dried over $MgSO_4$, filtered, and concentrated under reduced pressure. Purification by FC (pentane/EtOAc 100:0 to 95:5) afforded title compound (660 mg, 3.62 mmol, 81%) as a colorless oil. NMR data matched those from reported literature.[7a] For alcohol **II**: a solution of intermediate **I** (320 mg, 1.76 mmol, 1 eq.) in DCM (9 mL) under N_2 atmosphere was treated by dropwise addition of DIBAL-H (1.0 M in toluene, 4.39 mL, 4. 39 mmol, 2.5 eq.) at -78 °C and the resulting solution was stirred at the same temperature for 1 h. The reaction mixture was then diluted with Et_2O and cooled to 0 °C, quenched with 0.1 mL of water (slow addition), then 0.1 mL of a sat. aq. NaOH solution, and again 0.2 mL water. The mixture was then warmed to room temperature and stirred for 15 min, after which $MgSO_4$ was added and stirred for another 15 min before filtration and concentration under reduced pressure to give a colorless oily residue which was used in the next step without further purification. For crude intermediate **II**: ^{1}H NMR (400 MHz, Chloroform-*d*) δ 5.65 – 5.54 (m, 2H), 4.29 – 3.90 (m, 2H), 2.06 – 1.88 (m, 1H), 1.83 – 1.57 (m, 4H), 1.38 – 0.97 (m, 6H).

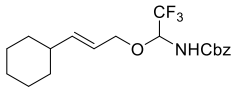

Prepared following GP1 from corresponding allyl alcohol (120 mg, 0.856 mmol). Purification was performed on a Biotage flash column chromatography system with a 12 g cartridge (SiO_2, 10 – 40% EtOAc in pentane) to afford title compound as white solid (195 mg, 0.530 mmol, 61% yield).

R_f = 0.51 in 95:5 pentane/EtOAc.

m.p.: 78 – 80 °C.

^{1}H NMR (400 MHz, Chloroform-*d*) δ 7.45 – 7.31 (m, 5H), 5.72 (dd, *J* = 15.6, 6.5 Hz, 1H), 5.56 – 5.30 (m, 3H), 5.16 (s, 2H), 4.18 (dd, *J* = 11.9, 5.9 Hz, 1H), 4.05 (dd, *J* = 11.9, 7.1 Hz, 1H), 2.08 – 1.90 (m, 1H), 1.77 – 1.61 (m, 5H), 1.37 – 0.98 (m, 5H).

^{13}C NMR (101 MHz, Chloroform-*d*) δ 155.5, 143.4, 135.6, 128.8, 128.7, 128.4, 122.3 (q, *J* = 281.3 Hz), 121.5, 77.9 (q, *J* = 35.1 Hz), 70.4, 67.8, 40.5, 32.7, 32.6, 26.2, 26.1.

^{19}F NMR (376 MHz, Chloroform-*d*) δ -80.8 (d, *J* = 5.9 Hz).

IR (cm^{-1}) 3339 (w), 2926 (m), 2853 (m), 1715 (s), 1526 (m), 1281 (m), 1235 (s), 1189 (s), 1156 (s), 1045 (s), 972 (m).

HRMS (nanochip-ESI/LTQ-Orbitrap) m/z: $[M + Na]^+$ Calcd for $C_{19}H_{24}F_3NNaO_3^+$ 394.1600; Found 394.1594.

[7] (a) Alhamadsheh, M. M.; Palaniappan, N.; DasChouduri, S.; Reynolds, K. A. *J. Am. Chem. Soc.* **2007**, *129*, 1910−1911; (b) Stiller, J.; Marqués-Lopez, E.; Herrera, R. P.; Frölich, R.; Strohmann, C.; Christmann, M. *Org. Lett.* **2011**, *13*, 70−73, respectively.

Benzyl (E)-(2,2,2-trifluoro-1-((4-phenylbut-2-en-1-yl)oxy)ethyl)carbamate (1u)

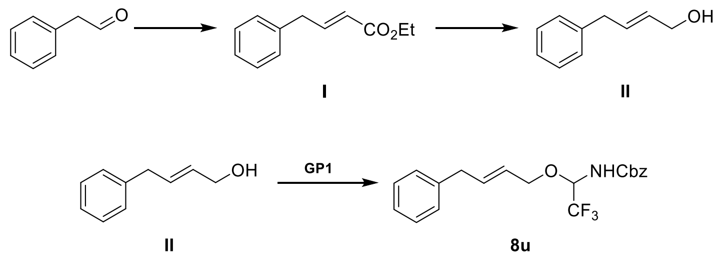

Ester **I** and alcohol **II** were prepared according to adapted literature procedures. [7b,8] NMR data of intermediate I matched those from reported literature,[9] whereas alcohol intermediate **II** was used in the next step without further purification. For ester **I**: a 100 mL RBF under N_2 atmosphere was charged with THF (40 mL), cooled to 0 °C and NaH (60% mineral oil, 509 mg, 12.7 mmol, 1.5 eq.) was added. Triethyl phosphonoacetate (2.50 mL, 12.7 mmol, 1.5 eq.) was added dropwise to the suspension at 0°C and the resulting mixture was stirred at the same temperature for 30 min. Then, phenylacetaldehyde (0.99 mL, 8.5 mmol, 1 eq.) was added dropwise to the reaction mixture at 0 °C. After complete addition, the reaction mixture was stirred at room temperature for 4 h. The reaction mixture was then diluted with sat. aq. NH_4Cl (150 mL), extracted with EtOAc (3 x 100 mL), washed with brine, dried over $MgSO_4$, filtered, and concentrated under reduced pressure. Purification by FC (biotage gradient: pentane/EtOAc 100:0 to 80:20) afforded title compound (780 mg, 4.10 mmol, 48%) as colorless oil. NMR data matched those from reported literature. For alcohol **II**: a solution of intermediate **I** (770 mg, 4.05 mmol, 1 eq.) in DCM (13.5 mL) under N_2 atmosphere was treated by dropwise addition of DIBAL-H (1.0 M in toluene, 8.10 mL, 8.10, 2 eq.) at -78 °C and the resulting solution was stirred at the same temperature for 2 h. The reaction mixture was then diluted with ether and cooled to 0 °C, quenched with 0.32 mL of water (slow addition), then 0.32 mL of a sat. aq. NaOH solution, and again 0.81 mL water. The mixture was then warmed to room temperature and stirred for 15 min, after which $MgSO_4$ was added and stirred for another 15 min before filtration and concentration under reduced pressure to give a yellow oil residue which was used in the next step without further purification. For crude intermediate **II**: ^{1}H NMR (400 MHz, Chloroform-*d*) δ 7.40 – 7.26 (m, 2H), 7.24 – 7.12 (m, 3H), 5.87 (dtt, *J* = 14.8, 6.6, 1.4 Hz, 1H), 5.71 (dtt, *J* = 15.2, 5.8, 1.5 Hz, 1H), 4.13 (t, *J* = 5.6 Hz, 2H), 3.39 (d, *J* = 6.7 Hz, 2H).

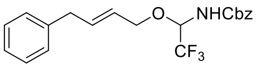

Prepared following GP1 from corresponding allyl alcohol (400 mg, 2.699 mmol). Purification was performed on a Biotage flash column chromatography system with a 40 g cartridge (SiO_2, 10 – 40% EtOAc in pentane) to afford title compound as white solid (618 mg, 1.63 mmol, 60% yield).

R_f = 0.53 in 9:1 pentane/EtOAc.

m.p.: 59 – 61 °C.

^{1}H NMR (400 MHz, Chloroform-*d*) δ 7.44 – 7.13 (m, 10H), 6.04 – 5.87 (m, 1H), 5.72 – 5.50 (m, 1H), 5.47 – 5.34 (m, 2H), 5.23 – 5.07 (m, 2H), 4.22 (dd, *J* = 12.1, 5.8 Hz, 1H), 4.16 – 4.03 (m, 1H), 3.41 (d, *J* = 6.7 Hz, 2H).

^{13}C NMR (101 MHz, Chloroform-*d*) δ 155.5, 139.6, 135.6, 135.5, 128.8, 128.7, 128.6, 128.4, 126.4, 126.2, 125.6, 122.2 (d, *J* = 281.5 Hz), 78.2 (q, *J* = 35.2 Hz), 69.9, 67.9, 38.8.

^{19}F NMR (376 MHz, Chloroform-*d*) δ -80.7 (d, *J* = 4.2 Hz).

IR (cm^{-1}) 3312 (w), 3032 (w), 1735 (s), 1532 (m), 1281 (s), 1236 (s), 1192 (s), 1046 (s), 973 (m).

HRMS (ESI/QTOF) m/z: $[M + Na]^+$ Calcd for $C_{20}H_{20}F_3NNaO_3^+$ 402.1287; Found 402.1280.

[8] Corbett, M. T.; Johnson, J. S. *Angew. Chem. Int. Ed.* **2014**, *53*, 255−259.
[9] Jung, H.; Schrader, M.; Kim, D.; Baik, M.-H.; Park, Y.; Chang, S. *J. Am. Chem. Soc.* **2019**, *141*, 15356−15366.

4-Methyl-N-(2,2,2-trifluoro-1-((2-methylallyl)oxy)ethyl)benzenesulfonamide (8a)

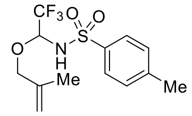

Prepared following GP1 from corresponding allyl alcohol (724 mg, 10.041 mmol). Purification was performed on a Biotage flash column chromatography system with a 25 g cartridge (SiO_2, 10 – 40% EtOAc in pentane) to afford title compound as amorphous solid (1.56 g, 4.82 mmol, 48% yield).

R_f value: 0.29 (20% Ethyl acetate in Pentane).

^{1}H NMR (400 MHz, Chloroform-d) δ 7.81 – 7.71 (m, 2H), 7.36 – 7.27 (m, 2H), 5.28 (d, *J* = 10.2 Hz, 1H), 5.06 – 4.94 (m, 3H), 4.08 (q, *J* = 12.4 Hz, 2H), 2.43 (s, 3H), 1.71 (s, 3H).

^{13}C NMR (101 MHz, $CDCl_3$) δ 144.4, 139.8, 137.7, 129.9, 127.0, 121.9 (q, *J* = 282.4 Hz), 114.8, 80.2 (q, *J* = 35.5 Hz), 72.7, 21.7, 19.4.

^{19}F NMR (376 MHz, Chloroform-*d*) δ -80.5.

IR (cm^{-1}) 3260 (w), 2930 (w), 1454 (w), 1341 (m), 1276 (m), 1193 (s), 1162 (s), 1076 (m), 917 (m), 816 (w), 668 (m).

HRMS (APCI/QTOF) m/z: $[M + H]^+$ Calcd for $C_{13}H_{17}F_3NO_3S^+$ 324.0876; Found 324.0869.

Benzyl (1-((3,3-diphenylallyl)oxy)-2,2,2-trifluoroethyl)carbamate (8b)

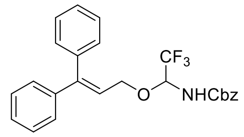

Prepared following GP1 from corresponding allyl alcohol (1.05 g, 5.00 mmol). Purification was performed on a Biotage flash column chromatography system (SiO_2, 10 – 40% EtOAc in pentane) to afford title compound as white solid (2.21 g, 5.00 mmol, quant.).

R_f value: 0.38 (20% Ethyl acetate in Pentane).

m.p.: 131 – 133 °C.

^{1}H NMR (400 MHz, Chloroform-*d*) δ 7.42 – 7.24 (m, 13H), 7.23 – 7.13 (m, 2H), 6.19 (t, *J* = 6.9 Hz, 1H), 5.51 – 5.24 (m, 2H), 5.19 – 5.00 (m, 2H), 4.30 (dd, *J* = 11.9, 6.8 Hz, 1H), 4.22 (dd, *J* = 11.9, 7.1 Hz, 1H).

^{13}C NMR (101 MHz, Chloroform-*d*) δ 155.3, 147.0, 141.5, 138.8, 135.5, 129.8, 128.8, 128.7, 128.4, 128.3, 128.3, 128.0, 127.9, 127.8, 122.9, 122.2 (q, *J* = 281.9 Hz), 79.0 (q, *J* = 35.3 Hz), 67.9, 67.4.

^{19}F NMR (376 MHz, Chloroform-*d*) δ -80.80 (d, *J* = 4.6 Hz).

IR (cm^{-1}) 3306 (m), 3036 (w), 1706 (s), 1531 (s), 1253 (s), 1197 (s), 1158 (s), 1055 (s), 701 (s).

HRMS (ESI/QTOF) m/z: $[M + Na]^+$ Calcd for $C_{25}H_{22}F_3NNaO_3^+$ 464.1444; Found 464.1432.

Benzyl (E)-(2,2,2-trifluoro-1-((2-methyl-3-phenylallyl)oxy)ethyl)carbamate (8c)

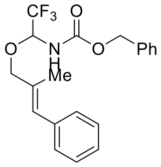

Prepared following GP1 from corresponding allyl alcohol (0.46 g, 3.12 mmol). Purification was performed on a Biotage flash column chromatography system with a 40 g cartridge (SiO_2, 10 – 40% EtOAc in pentane) to afford title compound as white solid (0.82 g, 2.15 mmol, 69% yield).

R_f value: 0.41 (20% Ethyl acetate in Pentane).

m.p.: 74 – 76 °C.

^{1}H NMR (400 MHz, Chloroform-d) δ 7.43 – 7.20 (m, 10H), 6.54 (s, 1H), 5.47 (dq, *J* = 9.4, 4.7 Hz, 1H), 5.40 (d, *J* = 10.5 Hz, 1H), 5.16 (s, 2H), 4.26 (d, *J* = 12.0 Hz, 1H), 4.18 (d, *J* = 12.0 Hz, 1H), 1.90 (d, *J* = 1.5 Hz, 3H).

^{13}C NMR (101 MHz, $CDCl_3$) δ 155.5, 137.1, 135.6, 132.9, 129.6, 129.1, 128.8, 128.7, 128.4, 128.3, 127.0, 122.3 (q, *J* = 281.3 Hz), 78.3 (q, *J* = 35.0 Hz), 75.7, 67.9, 15.4.

^{19}F NMR (376 MHz, Chloroform-*d*) δ -80.7.

IR (cm^{-1}) 3320 (w), 3032 (w), 1717 (s), 1516 (m), 1281 (s), 1235 (s), 1191 (s), 1160 (s), 1044 (s), 991 (m), 747 (m), 699 (s).

HRMS (ESI/QTOF) m/z: $[M + Na]^+$ Calcd for $C_{20}H_{20}F_3NNaO_3^+$ 402.1287; Found 402.1288.

Benzyl (E)-(2,2,2-trifluoro-1-((3-(1-methyl-1H-indol-3-yl)allyl)oxy)ethyl)carbamate (8d)

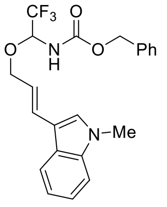

Prepared following GP1 from corresponding allyl alcohol (0.25 g, 1.31 mmol). Purification was performed on a Biotage flash column chromatography system (SiO_2, 10 – 40% EtOAc in pentane) to afford title compound as amorphous solid (350 mg, 0.836 mmol, 64% yield).

R_f value: 0.26 (20% Ethyl acetate in Pentane).

^{1}H NMR (400 MHz, Chloroform-d) δ 7.86 (d, *J* = 7.9 Hz, 1H), 7.40 – 7.29 (m, 5H), 7.29 – 7.24 (m, 2H), 7.19 (ddd, *J* = 8.1, 6.8, 1.4 Hz, 1H), 7.14 (s, 1H), 6.82 (d, *J* = 16.0 Hz, 1H), 6.21 (dt, *J* = 14.9, 6.8 Hz, 1H), 5.53 (dq, *J* = 9.8, 4.8 Hz, 1H), 5.39 (d, *J* = 10.5 Hz, 1H), 5.21 – 5.07 (m, 2H), 4.47 – 4.37 (m, 1H), 4.32 (dd, *J* = 12.0, 7.3 Hz, 1H), 3.76 (s, 3H).

^{13}C NMR (101 MHz, $CDCl_3$) δ 155.5, 137.7, 135.6, 129.2, 128.8, 128.8, 128.6, 128.6, 128.3, 126.2, 122.4, 122.4 (q, *J* = 281.7 Hz),120.3, 119.0, 112.7, 109.7, 78.0 (q, *J* = 35.0 Hz), 71.4, 67.9, 33.0.

^{19}F NMR (376 MHz, Chloroform-*d*) δ -80.7.

IR (cm^{-1}) 3313 (w), 2935 (w), 1715 (s), 1533 (m), 1335 (m), 1280 (s), 1238 (s), 1192 (s), 1160 (s), 1048 (s), 911 (m), 741 (s), 699 (m).

HRMS (ESI/QTOF) m/z: [M + Na]$^+$ Calcd for $C_{22}H_{21}F_3N_2NaO_3^+$ 441.1396; Found 441.1391.

Benzyl (E)-(2,2,2-trifluoro-1-((3-(1-tosyl-1H-pyrrol-2-yl)allyl)oxy)ethyl)carbamate (8e)

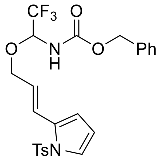

Prepared following GP1 from corresponding allyl alcohol (0.36 g, 1.30 mmol). Purification was performed on a Biotage flash column chromatography system with a 25 g cartridge (SiO_2, 10 – 40% EtOAc in pentane) to afford benzyl (E)-(2,2,2-trifluoro-1-((3-(1-tosyl-1H-pyrrol-2-yl)allyl)oxy)ethyl)carbamate as amorphous solid (343 mg, 0.674 mmol, 52% yield).

R_f value: 0.24 (20% Ethyl acetate in Pentane).

^{1}H NMR (400 MHz, Chloroform-d) δ 7.71 (d, *J* = 8.0 Hz, 2H), 7.42 – 7.28 (m, 7H), 7.25 (d, *J* = 7.0 Hz, 1H), 7.08 (d, *J* = 15.8 Hz, 1H), 6.44 – 6.38 (m, 1H), 6.23 (t, *J* = 3.4 Hz, 1H), 5.95 (dt, *J* = 15.8, 6.3 Hz, 1H), 5.42 (d, *J* = 4.0 Hz, 2H), 5.18 (s, 2H), 4.33 (dd, *J* = 13.1, 5.7 Hz, 1H), 4.22 (dd, *J* = 12.9, 6.8 Hz, 1H), 2.38 (s, 3H).

^{13}C NMR (101 MHz, $CDCl_3$) δ 155.6, 145.2, 136.0, 135.5, 132.4, 130.1, 128.8, 128.7, 128.4, 127.2, 124.9, 123.6, 122.9, 121.8 (q, *J* = 281.3 Hz), 112.6, 112.4, 78.4 (q, *J* = 35.0 Hz), 69.6, 68.0, 21.7.

^{19}F NMR (376 MHz, Chloroform-*d*) δ -80.6.

IR (cm^{-1}) 3308 (w), 2926 (w), 1715 (s), 1525 (m), 1279 (m), 1236 (s), 1192 (s), 1159 (s), 1046 (s), 968 (m), 780 (m), 740 (m), 699 (m).

HRMS (ESI/QTOF) m/z: [M + Na]$^+$ Calcd for $C_{24}H_{23}F_3N_2NaO_5S^+$ 531.1172; Found 531.1177.

C. Amino oxygenation of alkenes

C.1. General Procedure for the Amino oxygenation of alkenes (GP2)

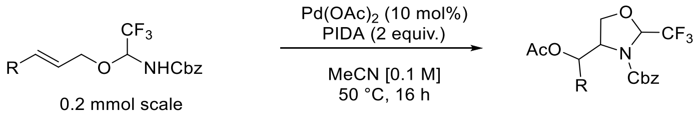

An oven-dried 8 mL microwave vial equipped with a Teflon coated stirring bar was charged with $Pd(OAc)_2$ (4.5 mg, 20 μmol, 10 mol%), PIDA (129 mg, 0.400 mmol, 2.00 equiv.) and tethered starting material (0.20 mmol, 1.0 equiv.). The vial was then sealed, purged with N_2 and placed in a heating metal block. 2.0 mL of MeCN were added and the suspension was stirred at 50 °C for 16 hours. Next, the reaction mixture was filtered through a plug of silica gel eluting with 15 mL of EtOAc and concentrated under reduced pressure. The crude material was purified by flash column chromatography on silica gel to afford the corresponding product.

C.2. Characterization of Amino oxygenation products

(Benzyl 4-(acetoxy(phenyl)methyl)-2-(trifluoromethyl)oxazolidine-3-carboxylate (2a)

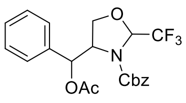

2a

Prepared according to the general procedure **GP2** using benzyl (1-(cinnamyloxy)-2,2,2-trifluoroethyl)carbamate (73 mg, 0.20 mmol, 1.0 equiv.). Crude dr 15:1. The crude material was purified by flash column chromatography (pentane/EtOAc gradient 100:0 to 80:20) to give **2a** (major, 74 mg, 0.17 mmol, 87% yield) as colorless oil.

For scale-up on 1.5 mmol scale: A round-bottom flask equipped with stirring bar was charged with $Pd(OAc)_2$ (34 mg, 0.15 mmol), PIDA (970 mg, 3.01 mmol), and benzyl (1-(cinnamyloxy)-2,2,2-trifluoroethyl)carbamate (550 mg, 1.50 mmol). The mixture was purged with nitrogen, subjected to vacuum and backfilled with nitrogen (3x cycles), diluted with dry acetonitrile (15 mL), heated to 50 °C and stirred at this temperature for 16 h. Crude dr 15:1. The crude material was purified by flash column chromatography (pentane/EtOAc gradient 98:2 to 80:20) to give **2a** (major, 469 mg, 1.11 mmol, 74% yield) as colorless oil.

R_f = 0.20 (10% EA/Pentane).

^{1}H NMR (400 MHz, Acetonitrile-*d3*) δ 7.48 – 7.29 (m, 10H), 5.83 (d, *J* = 8.8 Hz, 1H), 5.64 (q, *J* = 5.0 Hz, 1H), 5.22 ((d, *J* = 4.8 Hz, 2H), 4.72 – 4.59 (m, 1H), 4.04 (ddt, *J* = 9.0, 7.1, 0.9 Hz, 1H), 3.90 (ddd, *J* = 9.0, 4.8, 0.8 Hz, 1H), 1.89 (s, 3H).

^{13}C NMR (101 MHz, Acetonitrile-*d3)* δ 170.6, 155.9, 137.8, 137.2, 129.7, 129.6, 129.5, 129.2, 128.9, 128.6, 123.7 (q, *J* = 284.4 Hz), 87.2 (q, *J* = 35.5 Hz), 76.0, 69.9, 68.9, 61.1, 21.0.

^{19}F NMR (376 MHz, Acetonitrile-*d3*) δ -79.4.

IR (cm^{-1}) 2923 (w), 1725 (s), 1395 (m), 1348 (m), 1290 (s), 1231 (s), 1032 (m), 971 (m), 854 (w), 757 (m), 700 (s).

HRMS (ESI/QTOF) m/z: [M + Na]$^+$ Calcd for $C_{21}H_{20}F_3NNaO_5^+$ 446.1186; Found 446.1181.

Phenyl(3-tosyl-2-(trifluoromethyl)oxazolidin-4-yl)methyl acetate (2b)

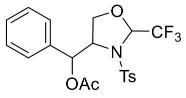

2b

Prepared according to the general procedure **GP2** using N-(1-(cinnamyloxy)-2,2,2-trifluoroethyl)-4-methylbenzenesulfonamide (77 mg, 0.20 mmol, 1.0 equiv.). Crude dr 3:2. The crude material was purified by flash column chromatography (pentane/EtOAc gradient 100:0 to 80:20) to give product **2b** as an inseparable mixture of diastereoisomers (major and minor, 63 mg, 0.14 mmol, 71% yield) as a viscous liquid.

R_f = 0.45 (20% EA/Pentane).

^{1}H NMR (400 MHz, Chloroform-*d*, ca. 1:0.7 mixture of diastereomers) 7.88 – 7.79 (m, 2H, major), 7.60 – 7.54 (m, 2H, minor), 7.43 – 7.22 (m, 14H, major and minor), 6.11 (d, *J* = 4.5 Hz, 1H, minor), 5.85 (d, *J* = 9.3 Hz, 1H, major), 5.63 (q, *J* = 5.1 Hz, 1H, major), 5.51 (q, *J* = 5.2 Hz, 1H, minor), 4.44 – 4.31 (m, 3H, major and minor), 3.92 – 3.82 (m, 1H, minor), 3.78 (dd, *J* = 9.2, 4.8 Hz, 1H, major), 3.64 – 3.55 (m, 1H), 2.46 (s, 3H, major), 2.43 (s, 3H, minor), 2.08 (s, 3H, minor), 1.85 (s, 3H, major).

^{13}C NMR (101 MHz, Chloroform-*d*, ca. 1:0.7 mixture of diastereomers) δ 169.6, 169.3, 145.3 (2C), 136.9, 136.2, 134.7, 134.0, 130.3, 130.2, 129.2, 129.0, 128.9, 128.7, 128.2 (2C), 127.9, 126.4, 122.4 (q, *J* = 285.2 Hz), 122.2 (q, *J* = 284.5 Hz), 87.9 (2q, *J* = 36.6 Hz, two quartets are merging for two diastereomer), 74.9, 73.0, 69.5, 68.9, 63.6, 62.7, 21.8 (2C), 20.9 (2C).

^{19}F NMR (376 MHz, Chloroform-*d*, ca. 1:0.69 mixture of diastereomers) δ -78.2 (minor), -78.8 (major).

IR (cm^{-1}) 2924 (w), 1746 (m), 1369 (m), 1229 (s), 1187 (s), 1165 (s), 1132 (s), 1026 (m), 760 (m), 703 (m), 670 (s).

HRMS (ESI/QTOF) m/z: [M + Na]$^+$ Calcd for $C_{20}H_{20}F_3NNaO_5S^+$ 466.0906; Found 466.0907.

Tert-butyl 4-(acetoxy(phenyl)methyl)-2-(trifluoromethyl)oxazolidine-3-carboxylate (2c)

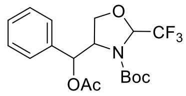

2c

Prepared according to the general procedure **GP2** using _tert_-butyl (1-(cinnamyloxy)-2,2,2-trifluoroethyl)carbamate (67 mg, 0.20 mmol, 1.0 equiv.). Crude dr >20:1. The crude material was purified by flash column chromatography (pentane/EtOAc gradient 100:0 to 80:20) to give the corresponding product **2c** (58 mg, 0.15 mmol, 74% yield, single diastereoisomer) as a viscous liquid.

R_f = 0.35 in 9:1 pentane/EtOAc.

^{1}H NMR (400 MHz, Chloroform-_d_) δ 7.40 – 7.27 (m, 5H), 5.91 (d, _J_ = 8.8 Hz, 1H), 5.50 (q, _J_ = 5.0 Hz, 1H), 4.60 (td, _J_ = 8.1, 5.6 Hz, 1H), 4.00 – 3.86 (m, 2H), 2.06 (s, 3H), 1.53 (s, 9H).

^{13}C{^{1}H} NMR (101 MHz, Chloroform-_d_) δ 169.7, 153.9, 136.6, 129.0, 128.8, 127.8, 122.6 (q, _J_ = 285.3 Hz), 86.4 (q, _J_ = 35.9 Hz), 82.6, 75.6, 69.2, 59.9, 28.2, 21.1.

^{19}F NMR (376 MHz, Chloroform-_d_) δ -79.5.

IR (cm^{-1}) 2922 (w), 1745 (m), 1719 (s), 1369 (s), 1234 (s), 1159 (s), 1038 (m), 963 (w), 853 (w), 703 (m).

HRMS (ESI/QTOF) m/z: [M + Na]$^+$ Calcd for $C_{18}H_{22}F_3NNaO_5^+$ 412.1342; Found 412.1342.

Benzyl 4-(acetoxy(4-fluorophenyl)methyl)-2-(trifluoromethyl)oxazolidine-3-carboxylate (2d)

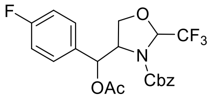

2d

Prepared following general procedure **GP2** using benzyl (_E_)-(2,2,2-trifluoro-1-((3-(4-fluorophenyl)allyl)oxy)ethyl)carbamate (77 mg, 0.20 mmol, 1.0 equiv.). Crude dr 4.5:1. Purification by flash column chromatography (SiO_2; pentane/EtOAc 98:2 to 90:10) gave product **2d** as an inseparable mixture of diastereoisomers (major and minor, 62 mg, 0.14 mmol, 70% yield) as colorless oil.

R_f = 0.30 in 9:1 pentane/EtOAc.

^{1}H NMR (400 MHz, Chloroform-_d_, ca. 5:1 mixture of diastereoisomers) δ 7.44 – 7.35 (m, 10H, major and minor), 7.34 – 7.27 (m, 4H, major and minor), 7.09 – 6.99 (m, 2H, major), 6.99 – 6.90 (m, 2H, minor), 6.21 (br, 1H, minor), 5.90 (d, _J_ = 8.8 Hz, 1H, major), 5.68 – 5.51 (m, 2H, major and minor; for major: 5.59 (q, _J_ = 5.0 Hz)), 5.25 (s, 2H, major), 5.15 (d, _J_ = 12.0 Hz, 1H, minor), 5.06 (br, 1H, minor), 4.63 (td, _J_ = 8.2, 5.6 Hz, 1H, major), 4.43 – 4.28 (m, 2H, minor), 4.11 – 4.01 (m, 1H, minor), 4.02 – 3.93 (m, 1H, major), 3.89 (dd, _J_ = 9.2, 5.4 Hz, 1H, major), 2.11 (s, 3H, minor), 1.93 (s, 3H, major).

^{13}C NMR (101 MHz, Chloroform-_d_, mixture of diastereoisomers, signals not fully resolved) δ 169.6, 169.5, 162.9 (d, _J_ = 248.1 Hz), 154.8, 154.6, 135.4, 135.1, 132.7, 132.2 (d, _J_ = 3.2 Hz), 129.5 (d, _J_ = 8.3 Hz), 128.7, 128.6, 128.2, 127.9 (d, _J_ = 8.2 Hz), 122.3 (q, _J_ = 285.0 Hz), 115.8 (d, _J_ = 21.5 Hz), 115.5, 86.3 (q, _J_ = 36.5 Hz), 74.6, 72.0, 69.0, 68.7, 68.6, 61.5, 60.1, 20.8.

^{19}F NMR (376 MHz, Chloroform-_d_, mixture of diastereoisomers) δ -78.5 – -78.9 (m, C_F_$_3$, minor), -79.4 (d, _J_ = 6.5 Hz, C_F_$_3$, major), -112.4 (ddd, _J_ = 14.2, 8.9, 5.3 Hz, Ar_F_, major), -113.3 (br, Ar_F_, minor).

IR (cm^{-1}) 3039 (w), 2958 (w), 2920 (w), 1728 (s), 1608 (w), 1512 (m), 1392 (m), 1296 (s), 1157 (s).

HRMS (ESI/QTOF) m/z: [M + Na]$^+$ Calcd for $C_{21}H_{19}F_4NNaO_5^+$ 464.1092; Found 464.1100.

Benzyl 4-(acetoxy(4-chlorophenyl)methyl)-2-(trifluoromethyl)oxazolidine-3-carboxylate (2e)

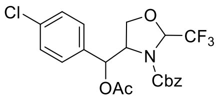

2e

Prepared following general procedure **GP2** using benzyl (*E*)-(2,2,2-trifluoro-1-((3-(4-chlorophenyl)allyl)oxy)ethyl)carbamate (80 mg, 0.20 mmol, 1.0 equiv.). Crude dr 8:1. Purification by flash column chromatography (SiO_2; pentane/EtOAc 98:2 to 90:10) gave product **2e** as an inseparable mixture of diastereoisomers (major and minor, 56 mg, 0.12 mmol, 61% yield) as yellow oil.

R_f = 0.29 in 9:1 pentane/EtOAc.

^{1}H NMR (400 MHz, Chloroform-*d*, ca. 11:1 mixture of diastereoisomers) δ 7.49 – 7.35 (m, 10H, major and minor), 7.34 – 7.29 (m, 4H, major and minor), 7.28 – 7.22 (m, 4H, major and minor), 6.19 (br, 1H, minor), 5.87 (d, *J* = 8.8 Hz, 1H, major), 5.59 (br q, *J* = 5.0 Hz, 2H, major and minor), 5.24 (s, 2H, major), 5.15 (d, *J* = 11.9 Hz, 2H, minor), 4.62 (td, *J* = 8.0, 5.4 Hz, 1H, major), 4.40 – 4.29 (m, 2H, minor), 4.09 – 4.01 (m, 1H, minor), 3.98 (t, *J* = 8.3 Hz, 1H, major), 3.89 (dd, *J* = 9.2, 5.4 Hz, 1H, major), 2.11 (s, 3H, minor), 1.93 (s, 3H, major).

^{13}C NMR (101 MHz, Chloroform-*d*, mixture of diastereoisomers, signals not fully resolved) δ 169.6, 154.9, 135.6, 135.5, 135.0, 134.9, 129.2, 129.1, 128.8, 128.7, 128.3, 127.6, 122.4 (q, *J* = 285.0 Hz), 86.4 (q, *J* = 36.2 Hz), 74.7, 72.1, 69.1, 68.8, 61.6, 60.1, 20.9.

^{19}F NMR (376 MHz, Chloroform-*d*, mixture of diastereoisomers) δ -78.7 (br, minor), -79.3 (d, *J* = 6.2 Hz, major).

IR (cm^{-1}) 3035 (w), 2958 (w), 2924 (w), 1728 (s), 1493 (w), 1392 (m), 1292 (s), 1227 (s), 1157 (s).

HRMS (ESI/QTOF) m/z: $[M + Na]^+$ Calcd for $C_{21}H_{19}ClF_3NNaO_5^+$ 480.0796; Found 480.0798.

Benzyl 4-(acetoxy(4-bromophenyl)methyl)-2-(trifluoromethyl)oxazolidine-3-carboxylate (2f)

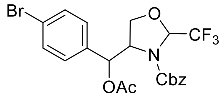

2f

Prepared according to the general procedure **GP2** using benzyl (*E*)-(1-((3-(4-bromophenyl)allyl)oxy)-2,2,2-trifluoroethyl)carbamate (89 mg, 0.20 mmol, 1.0 equiv.). Crude dr 7:1. The crude material was purified by flash column chromatography (pentane/EtOAc gradient 100:0 to 80:20) to give the corresponding product **2f** as an inseparable mixture of diastereoisomers (major and minor, 88 mg, 0.17 mmol, 88% yield) as a viscous liquid.

R_f = 0.24 in 9:1 Pentane/EtOAc.

^{1}H NMR (400 MHz, Chloroform-*d, ca. 11:1 mixture of diastereomers, assigned major diastereomer*) δ 7.52 – 7.44 (m, 2H), 7.45 – 7.33 (m, 5H), 7.26 – 7.16 (m, 2H), 5.85 (d, *J* = 8.7 Hz, 1H), 5.58 (q, *J* = 5.0 Hz, 1H), 5.24 (s, 2H), 4.61 (td, *J* = 8.0, 5.4 Hz, 1H), 3.98 (t, *J* = 8.3 Hz, 1H), 3.89 (dd, *J* = 9.2, 5.4 Hz, 1H), 1.93 (s, 3H).

^{13}C NMR (101 MHz, Chloroform-*d, ca. 11:1 mixture of diastereomers, assigned major diastereomer*) δ 169.6, 154.9, 135.5, 135.4, 132.1, 129.4, 128.8, 128.7, 128.3, 123.2, 122.4 (q, *J* = 285.0 Hz), 86.4 (q, *J* = 36.2 Hz), 74.7, 69.0, 68.7, 60.0, 20.8.

^{19}F NMR (376 MHz, Chloroform-*d, ca. 11:1 mixture of diastereomers, assigned major diastereomer*) δ -79.3.

IR (cm^{-1}) 2923 (w), 1725 (s), 1395 (s), 1291 (s), 1228 (s), 1157 (s), 853 (m), 738 (m), 697 (m).

HRMS (ESI/QTOF) m/z: $[M + Na]^+$ Calcd for $C_{21}H_{19}BrF_3NNaO_5^+$ 524.0291; Found 524.0295.

Benzyl 4-(acetoxy(4-nitrophenyl)methyl)-2-(trifluoromethyl)oxazolidine-3-carboxylate (2g)

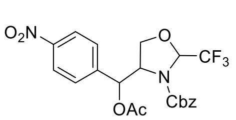

2g

Prepared following general procedure **GP2** using benzyl (*E*)-(2,2,2-trifluoro-1-((3-(4-nitrophenyl)allyl)oxy)ethyl)carbamate (82 mg, 0.20 mmol, 1.0 equiv.). Crude dr >20:1. Purification by flash column chromatography (SiO_2; pentane/EtOAc 98:2 to 80:20) gave compound **2g** (48 mg, 0.10 mmol, 51% yield, single diastereoisomer) as a yellow oil.

R_f = 0.27 in 4:1 pentane/EtOAc.

^{1}H NMR (400 MHz, Chloroform-*d*) δ 8.33 – 8.01 (m, 2H), 7.44 (d, *J* = 8.7 Hz, 2H), 7.42 – 7.29 (m, 5H), 6.08 (d, *J* = 7.6 Hz, 1H), 5.55 (q, *J* = 5.0 Hz, 1H), 5.23 (s, 2H), 4.61 (q, *J* = 7.0 Hz, 1H), 4.20 – 4.02 (m, 1H), 3.96 (dd, *J* = 9.2, 6.1 Hz, 1H), 2.02 (s, 3H).

^{13}C NMR (101 MHz, Chloroform-*d*) δ 169.5, 154.8, 148.2, 143.2, 135.3, 128.9, 128.9, 128.5, 128.5, 123.9, 122.3 (q, *J* = 281.7 Hz), 86.4 (q, *J* = 36.0 Hz), 73.9, 68.9, 68.8, 59.9, 20.8.

^{19}F NMR (376 MHz, Chloroform-*d*) δ -79.2 (d, *J* = 4.9 Hz).

IR (cm^{-1}) 3074 (w), 2958 (w), 1724 (s), 1523 (m), 1392 (m), 1346 (s), 1292 (s), 1227 (s), 1157 (s).

HRMS (ESI/QTOF) m/z: $[M + Na]^+$ Calcd for $C_{21}H_{19}F_3N_2NaO_7^+$ 491.1037; Found 491.1037.

Benzyl 4-(acetoxy(4-(trifluoromethyl)phenyl)methyl)-2-(trifluoromethyl)oxazolidine-3-carboxylate (2h)

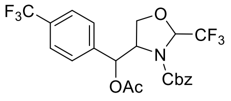

2h

Prepared according to the general procedure **GP2** using benzyl (*E*)-(2,2,2-trifluoro-1-((3-(4-(trifluoromethyl)phenyl)allyl)oxy)ethyl)carbamate (87 mg, 0.20 mmol, 1.0 equiv.). Crude dr >20:1. The crude material was purified by flash column chromatography (pentane/EtOAc gradient 100:0 to 80:20) to give product **2h** (73 mg, 0.15 mmol, 74% yield, single diastereomer) as yellow oil.

R_f = 0.35 in 9:1 Pentane/EtOAc.

^{1}H NMR (400 MHz, Chloroform-*d*) δ 7.63 – 7.56 (m, 2H), 7.46 – 7.32 (m, 7H), 5.98 (d, *J* = 8.4 Hz, 1H), 5.58 (q, *J* = 4.9 Hz, 1H), 5.25 (d, *J* = 2.4 Hz, 2H), 4.63 (td, *J* = 7.8, 5.5 Hz, 1H), 4.08 – 3.97 (m, 1H), 3.92 (dd, *J* = 9.2, 5.6 Hz, 1H), 1.97 (s, 3H).

^{13}C NMR (101 MHz, Chloroform-*d*) δ 169.6, 154.9, 140.3, 135.4, 131.2 (q, *J* = 32.6 Hz), 128.8, 128.8, 128.4, 128.1, 125.9 (q, *J* = 3.8 Hz), 123.9 (q, *J* = 272.2 Hz), 122.3 (q, *J* = 285.2 Hz), 86.4 (q, *J* = 36.2 Hz), 74.6, 69.0, 68.8, 60.0, 20.8.

^{19}F NMR (376 MHz, Chloroform-*d*) δ -62.8, -79.4.

IR (cm^{-1}) 2923 (w), 1727 (s), 1395 (m), 1326 (s), 1229 (s), 1162 (s), 1129 (s), 1068 (m), 851 (m), 757 (m), 698 (m).

HRMS (nanochip-ESI/LTQ-Orbitrap) m/z: [M + H]$^+$ Calcd for $C_{22}H_{20}F_6NO_5^+$ 492.1240; Found 492.1246.

Benzyl 4-(acetoxy(4-methoxyphenyl)methyl)-2-(trifluoromethyl)oxazolidine-3-carboxylate (2i)

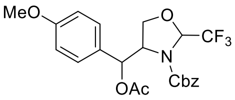

2i

Prepared following general procedure **GP2** using Benzyl (*E*)-(2,2,2-trifluoro-1-((3-(4-methoxyphenyl)allyl)oxy)ethyl)carbamate (79 mg, 0.20 mmol, 1.0 equiv.). Crude dr >20:1. Purification by flash column chromatography (SiO_2; pentane/EtOAc 98:2 to 90:10) gave compound **2i** (58 mg, 0.13 mmol, 64% yield, single diastereoisomer) as yellow oil.

R_f = 0.30 in 9:1 pentane/EtOAc.

^{1}H NMR (400 MHz, Chloroform-*d*) δ 7.43 – 7.30 (m, 5H), 7.15 (s, 2H), 6.81 (br d, *J* = 8.2 Hz, 2H), 6.27 (br d, *J* = 4.3 Hz, 1H), 5.59 – 5.49 (m, 1H), 5.19 (d, *J* = 12.0 Hz, 1H), 5.13 – 5.02 (br m, 1H), 4.41 (t, *J* = 7.9 Hz, 1H), 4.37 – 4.26 (br m, 1H), 4.13 – 3.98 (m, 1H), 3.78 (s, 3H), 2.11 (s, 3H).

^{13}C NMR (101 MHz, Chloroform-*d*) δ 169.8, 159.7, 154.9, 135.3, 129.1, 128.8, 128.7, 128.4, 127.4, 122.8 (d, *J* = 286.0 Hz), 114.2, 86.30 (d, *J* = 35.7 Hz), 72.0, 68.8, 68.8, 61.9, 55.4, 21.0.

^{19}F NMR (376 MHz, Chloroform-*d*) δ -78.7 (br s).

IR (cm^{-1}) 2958 (w), 2843 (w), 1728 (s), 1616 (w), 1516 (m), 1396 (m), 1300 (s), 1234 (s), 1169 (s).

HRMS (ESI/QTOF) m/z: [M + Na]$^+$ Calcd for $C_{22}H_{22}F_3NNaO_6^+$ 476.1291; Found 476.1297.

Benzyl 4-(acetoxy(3,5-difluorophenyl)methyl)-2-(trifluoromethyl)oxazolidine-3-carboxylate (2k)

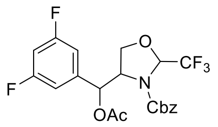

2k

Prepared according to the general procedure **GP2** using benzyl (*E*)-(1-((3-(3,5-difluorophenyl)allyl)oxy)-2,2,2-trifluoroethyl)carbamate (80 mg, 0.20 mmol, 1.0 equiv.). Crude dr >20:1. The crude material was purified by flash column chromatography (pentane/EtOAc gradient 100:0 to 80:20) to give product **2k** (71 mg, 0.15 mmol, 77% yield, single diastereomer) as colourless oil.

R_f = 0.36 in 9:1 Pentane/EtOAc.

^{1}H NMR (400 MHz, Chloroform-*d)* δ 7.46 – 7.30 (m, 5H), 6.94 – 6.82 (m, 2H), 6.76 (tt, *J* = 8.8, 2.3 Hz, 1H), 5.91 (d, *J* = 8.2 Hz, 1H), 5.57 (q, *J* = 5.0 Hz, 1H), 5.25 (s, 2H), 4.56 (td, *J* = 7.8, 5.8 Hz, 1H), 4.10 – 4.01 (m, 1H), 3.96 (dd, *J* = 9.2, 5.7 Hz, 1H), 1.98 (s, 3H).

^{13}C{^{1}H} NMR (101 MHz, Chloroform-*d*) δ 169.4, 163.2 (dd, *J* = 250.3, 12.7 Hz), 154.8, 140.0 (t, *J* = 8.8 Hz), 135.4, 128.9, 128.8, 128.3, 122.3 (q, *J* = 285.2 Hz), 111.0 – 110.4 (m), 104.5 (t, *J* = 25.1 Hz), 86.3 (q, *J* = 36.3 Hz), 74.0, 68.9, 68.9, 59.9, 20.8.

^{19}F NMR (376 MHz, Chloroform-*d*) δ -79.4, -108.3.

IR (cm^{-1}) 2922 (w), 1747 (s), 1602 (m), 1460 (m), 1293 (s), 1230 (s), 1185 (s), 1162 (s), 1127 (s), 966 (m), 854 (m), 702 (m).

HRMS (ESI/QTOF) m/z: [M + Na]$^+$ Calcd for $C_{21}H_{18}F_5NNaO_5^+$ 482.0997; Found 482.1006.

Benzyl 4-(acetoxy(2,6-difluorophenyl)methyl)-2-(trifluoromethyl)oxazolidine-3-carboxylate (2l)

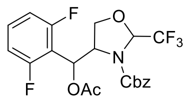

2l

Prepared according to the general procedure **GP2** using benzyl (*E*)-(1-((3-(2,6-difluorophenyl)allyl)oxy)-2,2,2-trifluoroethyl)carbamate (80 mg, 0.20 mmol, 1.0 equiv.). Crude dr >20:1. The crude material was purified by flash column chromatography (pentane/EtOAc gradient 100:0 to 80:20) to give product **2l** (51 mg, 0.11 mmol, 56% yield, single diastereoisomer) as colourless oil.

R_f = 0.34 in 9:1 Pentane/EtOAc.

^{1}H NMR (400 MHz, Chloroform-*d*) δ 7.44 – 7.23 (m, 6H), 6.90 (t, *J* = 8.4 Hz, 2H), 6.28 (d, *J* = 9.2 Hz, 1H), 5.65 (q, *J* = 5.1 Hz, 1H), 5.26 (d, *J* = 1.4 Hz, 2H), 5.01 (td, *J* = 8.4, 5.5 Hz, 1H), 4.08 (ddd, *J* = 9.1, 7.7, 1.1 Hz, 1H), 3.88 (dd, *J* = 9.2, 5.2 Hz, 1H), 1.90 (s, 3H).

^{13}C NMR (101 MHz, Chloroform-*d*) δ 169.8, 161.4 (dd, *J* = 251.9, 7.6 Hz), 155.0, 135.5, 131.3 (t, *J* = 10.5 Hz), 128.8, 128.6, 128.1, 122.5 (q, *J* = 285.0 Hz), 112.6 (t, *J* = 16.9 Hz), 112.2 (d, *J* = 25.1 Hz), 86.5 (q, *J* = 36.2 Hz), 69.3, 68.7, 67.4, 58.7, 20.6.

^{19}F NMR (376 MHz, Chloroform-*d*) δ -79.6, -111.4.

IR (cm^{-1}) 2922 (w), 1724 (s), 1627 (m), 1471 (m), 1394 (m), 1286 (s), 1229 (s), 1157 (s), 968 (m), 791 (m), 736 (m), 696 (m).

HRMS (ESI/QTOF) m/z: $[M + Na]^+$ Calcd for $C_{21}H_{18}F_5NNaO_5^+$ 482.0997; Found 482.0983.

Benzyl 4-(acetoxy(2-methoxyphenyl)methyl)-2-(trifluoromethyl)oxazolidine-3-carboxylate (2m)

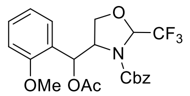

2m

Prepared according to the general procedure **GP2** using benzyl (E)-(2,2,2-trifluoro-1-((3-(2-methoxyphenyl)allyl)oxy)ethyl)carbamate (79 mg, 0.20 mmol, 1.0 equiv.). Crude dr >20:1. The crude material was purified by flash column chromatography (pentane/EtOAc gradient 100:0 to 80:20) to give product **2m** (68 mg, 0.15 mmol, 75% yield, single diastereomer) as colourless oil.

R_f = 0.32 in 9:1 Pentane/EtOAc.

^{1}H NMR (400 MHz, Chloroform-*d*) δ 7.42 – 7.28 (m, 6H), 7.28 – 7.24 (m, 1H), 6.94 (td, *J* = 7.5, 1.1 Hz, 1H), 6.84 (d, *J* = 8.3 Hz, 1H), 6.37 (d, *J* = 8.1 Hz, 1H), 5.59 (q, *J* = 5.0 Hz, 1H), 5.22 (d, *J* = 12.3 Hz, 1H), 5.12 (d, *J* = 12.3 Hz, 1H), 4.72 (td, *J* = 7.8, 5.2 Hz, 1H), 4.08 – 3.94 (m, 2H), 3.77 (s, 3H), 1.94 (s, 3H).

^{13}C{^{1}H} NMR (101 MHz, Chloroform-*d*) δ 169.8, 156.9, 155.1, 135.7, 129.9, 128.7, 128.5, 128.2 (2C), 125.5, 122.6 (q, *J* = 285.2 Hz), 121.0, 111.1, 86.6 (q, *J* = 36.5 Hz), 69.9, 69.3, 68.4, 60.3, 55.7, 21.0.

^{19}F NMR (376 MHz, Chloroform-*d*) δ -79.1.

IR (cm^{-1}) 2929 (w), 1726 (s), 1496 (m), 1396 (m), 1293 (s), 1233 (s), 1182 (s), 1158 (s), 1031 (m), 757 (m), 738 (m), 697 (m), 607 (w).

HRMS (nanochip-ESI/LTQ-Orbitrap) m/z: $[M + Na]^+$ Calcd for $C_{22}H_{22}F_3NNaO_6^+$ 476.1291; Found 476.1269.

Benzyl 4-(acetoxy(m-tolyl)methyl)-2-(trifluoromethyl)oxazolidine-3-carboxylate (2n)

Prepared according to the general procedure **GP2** using benzyl (*E*)-(2,2,2-trifluoro-1-((3-(m-tolyl)allyl)oxy)ethyl)carbamate (76 mg, 0.20 mmol, 1.0 equiv.). Crude dr 9:1. The crude material was purified by flash column chromatography (pentane/EtOAc gradient 100:0 to 80:20) to give product **2n** (major, 60 mg, 0.14 mmol, 69% yield, minor not observed) as colourless oil.

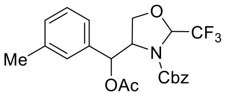

2n

R_f = 0.34 in 9:1 pentane/EtOAc.

^{1}H NMR (400 MHz, Chloroform-*d*) δ 7.51 – 7.30 (m, 5H), 7.28 – 7.19 (m, 1H), 7.14 (d, *J* = 8.5 Hz, 3H), 5.83 (d, *J* = 9.3 Hz, 1H), 5.61 (q, *J* = 5.0 Hz, 1H), 5.26 (s, 2H), 4.73 – 4.63 (m, 1H), 3.98 – 3.86 (m, 2H), 2.33 (s, 3H), 1.91 (s, 3H).

^{13}C NMR (101 MHz, Chloroform-*d*) δ 169.8, 155.0, 138.7, 136.5, 135.6, 129.9, 128.8, 128.8, 128.6, 128.5, 128.2, 124.8, 122.5 (q, *J* = 285.0 Hz), 86.4 (q, *J* = 36.3 Hz), 75.7, 69.3, 68.6, 60.3, 21.5, 21.0.

^{19}F NMR (376 MHz, Chloroform-*d*) δ -79.4.

IR (cm^{-1}) 2923 (w), 1726 (s), 1396 (s), 1348 (m), 1293 (s), 1233 (s), 1158 (s), 1038 (m), 974 (m), 757 (m), 699 (m).

HRMS (ESI/QTOF) m/z: $[M + Na]^+$ Calcd for $C_{22}H_{22}F_3NNaO_5^+$ 460.1342; Found 460.1337.

Benzyl 4-(acetoxy(phenyl)methyl)-2-(trifluoromethyl)oxazolidine-3-carboxylate (2a')

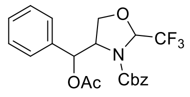

2a'

Prepared following general procedure **GP2** using benzyl (*Z*)-(2,2,2-trifluoro-1-((3-phenylallyl)oxy)ethyl)carbamate (91 mg, 0.20 mmol, 1.0 equiv.). Crude dr 2:5 (inverse compared to **2a**). Purification by flash column chromatography (SiO_2; pentane/EtOAc 98:2 to 90:10) gave compound **2a'** (major, 15 mg, 0.035 mmol, 18% yield, minor not observed) as yellow oil.

R_f = 0.32 in 9:1 pentane/EtOAc.

^{1}H NMR (400 MHz, Acetonitrile-*d3*) δ 7.42 – 7.21 (m, 10H), 6.07 (d, *J* = 5.5 Hz, 1H), 5.61 (q, *J* = 5.1 Hz, 1H), 5.11 (d, *J* = 12.4 Hz, 1H), 4.98 (d, *J* = 12.3 Hz, 1H), 4.51 – 4.42 (m, 1H), 4.37 (ddd, *J* = 8.8, 6.3, 0.9 Hz, 1H), 4.06 (ddd, *J* = 8.5, 6.6, 1.1 Hz, 1H), 2.07 (s, 3H).

^{13}C NMR (101 MHz, Acetonitrile-*d3*, *one carbon signal not resolved*) δ 170.8, 155.8, 138.4, 136.9, 129.5, 129.5, 129.3, 129.0, 127.1, 124.0 (d, *J* = 285.1 Hz), 87.0 (d, *J* = 35.5 Hz), 73.5, 69.6, 69.0, 62.5, 21.0.

^{19}F NMR (376 MHz, Acetonitrile-*d3*) δ -78.5 (br s).

IR (cm^{-1}) 3552 (w), 3035 (w), 2958 (w), 2264 (w), 1732 (s), 1400 (m), 1300 (s), 1230 (s), 1161 (s).

HRMS (nanochip-ESI/LTQ-Orbitrap) m/z: [M + Na]$^+$ Calcd for $C_{21}H_{20}F_3NNaO_5^+$ 446.1186; Found 446.1183.

Benzyl 4-(acetoxymethyl)-2-(trifluoromethyl)oxazolidine-3-carboxylate (2o)

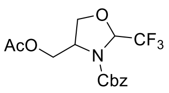

2o

Prepared following general procedure **GP2** using benzyl (1-(allyloxy)-2,2,2-trifluoroethyl)carbamate (28 mg, 0.20 mmol, 1.0 equiv.). Crude dr 2:1. Purification by FC (SiO_2; pentane/EtOAc 98:2 to 80:20) gave compound **2o** (isolated as major isomer >20:1 dr with traces of minor, 32 mg, 0.09 mmol, 46% yield) as yellow oil.

R_f = 0.26 in 9:1 pentane/EtOAc.

^{1}H NMR (400 MHz, Acetonitrile-*d3,* major) δ 7.45 – 7.33 (m, 5H), 5.75 (q, *J* = 5.3 Hz, 1H), 5.19 (d, *J* = 4.3 Hz, 2H), 5.02 – 4.88 (m, 1H), 4.45 (dd, *J* = 13.8, 7.7 Hz, 1H), 3.97 – 3.85 (m, 2H), 3.07 (dd, *J* = 13.8, 9.4 Hz, 1H), 2.01 (s, 3H).

^{13}C NMR (101 MHz, Acetonitrile-*d3,* major) δ 171.1, 155.4, 137.1, 129.6, 129.3, 128.9, 124.0 (q, *J* = 285.1 Hz), 80.1 (q, *J* = 34.8 Hz), 69.0, 67.4, 67.0, 41.1, 21.0.

^{19}F NMR (376 MHz, Acetonitrile-*d3,* major) δ -80.77 (d, *J* = 6.4 Hz).

IR (cm^{-1}) 3324 (w), 3036 (w), 2957 (w), 1718 (s), 1418 (m), 1227 (s), 1177 (s), 1155 (s), 1054 (m), 965 (m).

HRMS (ESI/QTOF) m/z: [M + Na]$^+$ Calcd for $C_{15}H_{16}F_3NNaO_5^+$ 370.0873; Found 370.0870.

Benzyl 4-(cyclohexylidenemethyl)-2-(trifluoromethyl)oxazolidine-3-carboxylate (2t)

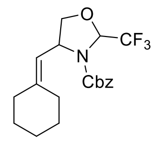

2t

Prepared following general procedure **GP2** using benzyl (E)-(1-((3-cyclohexylallyl)oxy)-2,2,2-trifluoroethyl)carbamate (74 mg, 0.20 mmol, 1.0 equiv.). Crude dr >20:1. Purification on a Biotage flash column chromatography system (SiO_2; pentane/EtOAc 100:0 to 95:5) gave compound **2t** as an inseparable mixture of diastereoisomers (major and minor[10], 16 mg, 0.043 mmol, 22% yield) as colourless oil.

R_f = 0.42 in 98:2 pentane/EtOAc.

^{1}H NMR (400 MHz, Acetonitrile-*d3,* major) δ 7.43 – 7.31 (m, 5H), 5.64 (q, *J* = 5.5 Hz, 1H), 5.13 (d, *J* = 2.0 Hz, 2H), 5.04 (d, *J* = 9.3 Hz, 1H), 4.92 (q, *J* = 8.1 Hz, 1H), 4.34 (ddt, *J* = 10.3, 7.9, 1.6 Hz, 1H), 3.78 (ddq, *J* = 9.2, 7.9, 1.4 Hz, 1H), 2.12 – 2.01 (m, 4H), 1.53 – 1.34 (m, 4H), 1.33 – 1.10 (m, 2H).

^{13}C NMR (101 MHz, Acetonitrile-*d3,* major) δ 155.8, 146.2, 137.3, 129.5, 129.2, 128.9, 124.4 (d, *J* = 286.1 Hz), 119.8, 86.2 (q, *J* = 34.9 Hz), 74.0, 68.5, 55.5, 37.6, 29.7, 29.2, 28.4, 27.2.

^{19}F NMR (376 MHz, Acetonitrile-*d3,* major) δ -80.1 (d, *J* = 7.4 Hz).

IR (cm^{-1}) 3609 (m), 3092 (w), 2604 (w), 2262 (s), 1631 (m), 1400 (w), 1035 (m), 833 (s).

HRMS (ESI/QTOF) m/z: [M + Na]$^+$ Calcd for $C_{19}H_{22}F_3NNaO_3^+$ 392.1444; Found 392.1445.

[10] Traces of minor diastereoisomer visible by NMR but not fully resolved. NMR data given for major diastereoisomer.

Benzyl (*E*)-4-styryl-2-(trifluoromethyl)oxazolidine-3-carboxylate (2u)

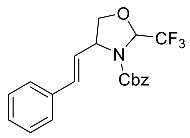

2u

Prepared following general procedure **GP2** using benzyl (*E*)-(2,2,2-trifluoro-1-((4-phenylbut-2-en-1-yl)oxy)ethyl)carbamate (85% purity, 89 mg, 0.20 mmol, 1.0 equiv.). Crude dr >20:1. Purification on a Biotage flash column chromatography system (SiO_2; pentane/EtOAc 100:0 to 85:15) gave compound **2u** (single diastereoisomer, 39 mg, 0.10 mmol, 52% yield) as yellow oil.

R_f = 0.24 in 95:5 pentane/EtOAc.

^{1}H NMR (400 MHz, Chloroform-*d*) δ 7.48 – 7.26 (m, 10H), 6.60 (d, *J* = 15.8 Hz, 1H), 6.08 (dd, *J* = 15.8, 8.3 Hz, 1H), 5.65 (q, *J* = 5.4 Hz, 1H), 5.19 (d, *J* = 3.8 Hz, 2H), 4.73 (p, *J* = 7.3 Hz, 1H), 4.59 – 4.33 (m, 1H), 4.07 (t, *J* = 8.5 Hz, 1H).

^{13}C NMR (101 MHz, Chloroform-*d*) δ 154.6, 136.0, 135.6, 135.0, 128.7, 128.5, 128.4, 128.2, 127.4, 126.8, 125.6, 123.1 (q, *J* = 286.8 Hz), 85.5 (q, *J* = 35.4 Hz), 72.8, 68.4, 59.9.

^{19}F NMR (376 MHz, Chloroform-*d*) δ -79.3 (d, *J* = 5.2 Hz).

IR (cm^{-1}) 3308 (w), 3033 (w), 2905 (w), 1719 (s), 1398 (s), 1344 (s), 1286 (s), 1154 (s), 1134 (s), 962 (s).

HRMS (ESI/QTOF) m/z: $[M + Na]^+$ Calcd for $C_{20}H_{18}F_3NNaO_3^+$ 400.1131; Found 400.1130.

Benzyl 4-(phenyl(pivaloyloxy)methyl)-2-(trifluoromethyl)oxazolidine-3-carboxylate (2v)

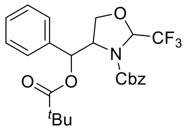

2v

Prepared according to the general procedure **GP2** using benzyl (1-(cinnamyloxy)-2,2,2-trifluoroethyl)carbamate (73 mg, 0.20 mmol, 1.0 equiv.) phenyl-λ3-iodanediyl bis(2,2-dimethylpropanoate) (0.16 mg, 0.40 mmol, 2.00 equiv.). Crude dr >20:1. The crude material was purified by flash column chromatography (pentane/EtOAc gradient 100:0 to 80:20) to give product **2v** (35 mg, 0.075 mmol, 38% yield, single diastereomer) as colourless oil.

R_f = 0.36 in 95:5 pentane/EtOAc.

^{1}H NMR (400 MHz, Acetonitrile-*d3*) δ 7.50 – 7.29 (m, 10H), 5.69 – 5.58 (m, 2H), 5.29 (d, *J* = 12.3 Hz, 1H), 5.17 (d, *J* = 12.4 Hz, 1H), 4.69 (ddd, *J* = 9.9, 6.6, 3.5 Hz, 1H), 4.04 – 3.90 (m, 2H), 1.05 (s, 9H).

^{13}C NMR (101 MHz, Acetonitrile-*d3*) δ 177.8, 155.7, 138.6, 137.0, 129.7 (2C), 129.5, 129.3, 129.1, 128.5, 123.6 (q, *J* = 283.6 Hz), 87.0 (q, *J* = 35.5 Hz), 75.8, 69.8, 69.0, 61.4, 39.3, 27.0.

^{19}F NMR (376 MHz, Acetonitrile-*d3)* δ -80.4.

IR (cm^{-1}) 2965 (w), 1731 (s), 1396 (m), 1349 (m), 1285 (s), 1157 (s), 974 (m), 912 (m), 737 (m), 700 (m).

HRMS (nanochip-ESI/LTQ-Orbitrap) m/z: $[M + Na]^+$ Calcd for $C_{24}H_{26}F_3NNaO_5^+$ 488.1655; Found 488.1631.

Benzyl 4-(((3-chlorobenzoyl)oxy)(phenyl)methyl)-2-(trifluoromethyl)oxazolidine-3-carboxylate (2w)

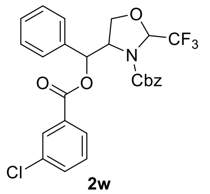

2w

Prepared following general procedure **GP2** using Benzyl (1-(cinnamyloxy)-2,2,2-trifluoroethyl)carbamate (73 mg, 0.20 mmol, 1.0 equiv.) and phenyl- λ3-iodanediyl bis(3-chlorobenzoate) **7a** (0.21 g, 0.40 mmol, 2.0 equiv.). Crude dr 9:2. Purification by flash column chromatography (SiO_2; pentane/EtOAc 98:2 to 90:10) gave product **2w** as an inseparable mixture of diastereoisomers (major and minor, 57 mg, 0.11 mmol, 55% yield) as a yellow oil.

R_f = 0.32 in 9:1 pentane/EtOAc.

^{1}H NMR (400 MHz, Acetonitrile-*d3*, ca. 4:1 mixture of diastereoisomers) δ 8.09 (t, *J* = 1.9 Hz, 1H, minor), 8.03 (t, *J* = 1.8 Hz, 1H, major), 8.00 (t, *J* = 1.4 Hz, 1H, minor), 7.88 (dt, *J* = 7.9, 1.3 Hz, 1H, major), 7.65 (ddd, *J* = 8.1, 2.2, 1.1 Hz, 1H, minor), 7.63 – 7.58 (m, 1H, major), 7.51 – 7.44 (m, 3H, major and minor), 7.43 – 7.25 (m, 19H, major and minor), 6.28 (d, *J* = 6.0 Hz, 1H, minor), 5.95 (d, *J* = 9.6 Hz, 1H, major), 5.70 (q, *J* = 4.8 Hz, 1H, major), 5.62 (q, *J* = 5.0 Hz, 1H, minor), 5.33 (d, *J* = 12.4 Hz, 1H, major), 5.17 – 5.10 (m, 2H, major and minor), 4.97 (d, *J* = 12.4 Hz, 1H, minor), 4.90 (ddd, *J* = 10.2, 7.1, 3.9 Hz, 1H, major), 4.65 (q, *J* = 6.2 Hz, 1H, minor), 4.50 (dd, *J* = 8.9, 5.7 Hz, 1H, minor), 4.22 – 4.12 (m, 1H, minor), 4.12 – 4.03 (m, 1H, major), 3.97 (dd, *J* = 9.1, 3.9 Hz, 1H, major).

^{13}C NMR (101 MHz, Acetonitrile-*d3*, mixture of diastereoisomers, signals not fully resolved) δ 165.0, 164.9, 155.9, 155.8, 138.2, 137.7, 137.0, 136.8, 135.1, 134.4, 134.2, 132.6, 131.3, 130.3, 130.1, 129.8, 129.6, 129.5, 129.5, 129.2, 128.9, 128.9, 128.8, 127.4, 123.7 (q, *J* = 283.9 Hz), 87.1 (q, *J* = 35.4 Hz), 77.7, 75.1, 69.9, 69.1, 69.0, 62.6, 61.4.

^{19}F NMR (376 MHz, Acetonitrile-*d3*, mixture of diastereoisomers) δ -79.3 – -79.6 (br m, minor), -80.2 (d, *J* = 4.7 Hz, major).

IR (cm^{-1}) 3323 (w), 3068 (w), 2919 (w), 1725 (s), 1575 (w), 1395 (m), 1288 (s), 1253 (s), 1157 (s).

HRMS (ESI/QTOF) m/z: $[M + Na]^+$ Calcd for $C_{26}H_{21}ClF_3NNaO_5^+$ 542.0953; Found 542.0957.

D. Additional not Successful Substrates (see starting materials 8a-8e)

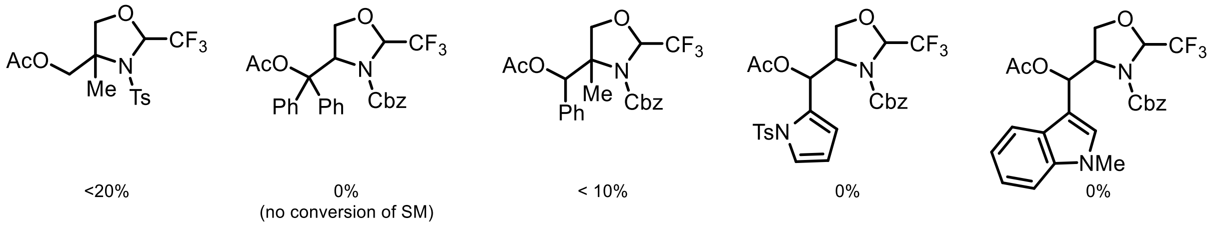

E. Tether Removal

Phenyl(2-(trifluoromethyl)oxazolidin-4-yl)methyl acetate (5a)

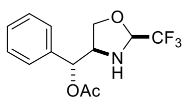

5a

To a solution of major isomer **2a** (450 mg, 1.06 mmol, 1 equiv.) in EtOH (36 mL) was added 20 wt% $Pd(OH)_2/C$ (10 mol%) at room temperature. The reaction mixture was purged with H_2 gas and stirred at room temperature for 30 min. The reaction mixture was then filtered through a pad of Celite and rinsed with DCM. The filtrate was concentrated under reduced pressure and purified by FC (Biotage gradient: pentane/EtOAc 95:5 to 60:40) affording desired compound **5a** (270 mg, 0.933 mmol, 88% yield) as white solid.

R_f = 0.14 in 9:1 pentane/EtOAc.

m.p.: 76 – 78 °C.

^{1}H NMR (400 MHz, Chloroform-*d*) δ 7.38 – 7.30 (m, 5H), 5.60 (d, *J* = 8.4 Hz, 1H), 4.98 (q, *J* = 5.2 Hz, 1H), 3.95 (q, *J* = 8.1 Hz, 1H), 3.71 (tq, *J* = 7.1, 1.4 Hz, 1H), 3.62 (td, *J* = 8.5, 1.0 Hz, 1H), 2.08 (s, 3H).

^{13}C NMR (101 MHz, Chloroform-*d*) δ 170.0, 137.7, 128.9, 128.9, 127.2, 123.4 (q, *J* = 283.1 Hz), 87.6 (q, *J* = 34.3 Hz), 77.6, 68.9, 61.6, 21.3.

^{19}F NMR (376 MHz, Chloroform-*d*) δ -81.6 (d, *J* = 5.3 Hz).

IR (cm^{-1}) 3365 (w), 2903 (w), 1739 (s), 1499 (m), 1373 (m), 1291 (m), 1237 (s), 1153 (s), 1026 (m).

HRMS (ESI/QTOF) m/z: $[M + Na]^+$ Calcd for $C_{13}H_{14}F_3NNaO_3^+$ 312.0818; Found 312.0823.

2-Amino-3-hydroxy-1-phenylpropyl acetate (4a)

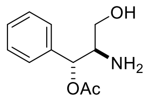

4a

To a solution of major isomer **2a** (400 mg, 0.945 mmol, 1 equiv.) in EtOH (32 mL) was added 20 wt% $Pd(OH)_2/C$ (10 mol%) at room temperature. The reaction mixture was purged with H_2 gas and stirred at room temperature for 30 min. The reaction mixture was then filtered through a pad of Celite and rinsed with DCM. The filtrate was concentrated under reduced pressure to give crude intermediate **5a** as an off-white solid, which was used in the next step without further purification. The latter was dissolved in a mixture of THF (16.1 mL) and H_2O (1.8 mL) to which was added PTSA (7 equiv) and the reaction was allowed to stir at room temperature overnight (full conversion of starting material observed by TLC). The reaction was dissolved in DCM and quenched by the addition of 1 M NaOH (18 mL). The organic layers were separated and the aqueous layer extracted with a 3:1 mixture of $CHCl_3$:isopropanol (4x), dried over Na_2SO_4, filtered, and concentrated under reduced pressure. Purification by FC (Biotage gradient: DCM/MeOH 95:5 to 60:40) afforded desired compound **5a** (144 mg, 0.688 mmol, 73% over two steps from **2a**) as white solid.

R_f = 0.59 in DCM/MeOH 4:1.

m.p.: 131 – 133 °C.

^{1}H NMR (400 MHz, Methanol-*d*) δ 7.38 (dtd, *J* = 7.5, 1.7, 1.0 Hz, 2H), 7.31 (ddd, *J* = 7.6, 6.7, 1.2 Hz, 2H), 7.27 – 7.18 (m, 1H), 4.92 (d, *J* = 4.4 Hz, 1H), 4.09 (td, *J* = 6.1, 4.4 Hz, 1H), 3.68 (dd, *J* = 11.0, 6.1 Hz, 1H), 3.46 (dd, *J* = 11.0, 5.9 Hz, 1H), 1.90 (s, 3H).

^{13}C NMR (101 MHz, Methanol-*d*) δ 173.5, 143.8, 129.1, 128.4, 127.4, 72.9, 62.5, 58.3, 22.6.

IR (cm^{-1}) 3370 (w), 2476 (m), 2235 (w), 2071 (m), 1629 (w), 1122 (s), 975 (s).

HRMS (ESI/QTOF) m/z: $[M + Na]^+$ Calcd for $C_{11}H_{15}NNaO_3^+$ 232.0944; Found 232.0940.

2-Amino-1-(4-fluorophenyl)-3-hydroxypropyl acetate (4d)

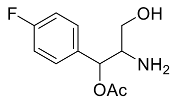

4d

To a solution of **2d** (mixture of diastereoisomers 4.2:1 dr, 115 mg, 0.261 mmol, 1 equiv.) in EtOH (9 mL) was added 20 wt% $Pd(OH)_2/C$ (10 mol%) at room temperature. The reaction mixture was purged with H_2 gas and stirred at room temperature for 30 min (full conversion of starting material by TLC: R_f = 0.27 in pentane/EtOAc 4:1). The reaction mixture was then filtered through a pad of Celite and rinsed with DCM. The filtrate was concentrated under reduced pressure to give crude intermediate as a colorless oil, which was used in the next step without further purification (mixture of diastereoisomers by crude ^{1}H NMR 4.3:1 dr). The crude residue was dissolved in a mixture of THF (4.7 mL) and H_2O (0.52 mL) to which was added PTSA (7 equiv) and the reaction was allowed to stir at room temperature overnight (crude ^{1}H NMR showed ca. 8:1 dr). The reaction was dissolved in DCM and quenched by the addition of 1 M NaOH (5 mL). The organic layers were separated and the aqueous layer extracted with a 3:1 mixture of $CHCl_3$:isopropanol (4x), dried over Na_2SO_4, filtered, and concentrated under reduced pressure. The crude was then passed through a short silica plug (DCM/MeOH 4:1) to give desired compound **4d** as amorphous solid (42 mg, 71% yield over two steps from **2d**, isolated as 7.3:1 dr).

R_f = 0.50 in DCM/MeOH 4:1.

^{1}H NMR (400 MHz, Methanol-*d*, ca. 7.3:1 mixture of diastereoisomers) δ 7.47 – 7.31 (m, 4H, major and minor), 7.09 – 6.98 (m, 4H, major and minor), 4.93 (d, *J* = 4.1 Hz, 1H, major), 4.72 (d, *J* = 7.1 Hz, 1H, minor), 4.06 (td, *J* = 6.1, 4.2 Hz, 2H, major and minor), 3.76 (dd, *J* = 11.3, 6.1 Hz, 1H, minor), 3.68 (dd, *J* = 10.9, 6.3 Hz, 2H, major and minor), 3.47 (dd, *J* = 10.9, 5.9 Hz, 1H, major), 1.89 (s, 3H, major), 1.83 (s, 3H, minor).

^{13}C NMR (101 MHz, Methanol-*d*, ca. 7.3:1 mixture of diastereoisomers, signals not fully resolved) δ 173.5, 173.1, 163.5 (q, *J* = 243.4 Hz), 139.9 (d, *J* = 3.1 Hz), 139.6 (d, *J* = 3.2 Hz), 129.6 (d, *J* = 8.0 Hz), 129.2 (d, *J* = 8.1 Hz), 115.7 (d, *J* = 21.5 Hz), 74.0, 72.1, 62.5, 61.8, 58.2, 57.9, 22.5.

^{19}F NMR (376 MHz, Methanol-*d*, ca. 7.3:1 mixture of diastereoisomers) δ -117.6 (ddd, *J* = 14.9, 9.6, 5.8 Hz, minor), -117.9 (ddd, *J* = 15.0, 9.3, 5.6 Hz, major).

IR (cm^{-1}) 3364 (w), 2469 (m), 2243 (w), 2215 (w), 2072 (m), 1634 (w), 1505 (w), 1225 (w), 1119 (m), 989 (m).

HRMS (ESI/QTOF) m/z: $[M + Na]^+$ Calcd for $C_{11}H_{14}FNNaO_3^+$ 250.0850; Found 250.0855.

F. X-Ray Crystallographic Data

F.1. Single Crystal X-Ray Diffraction for compound 5a (CCDC Number: 2173505) – ellipsoid plot (probability level 50%)

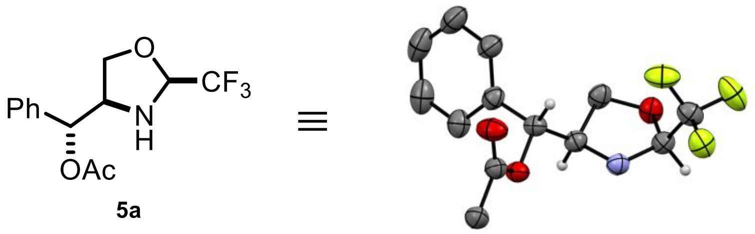

5a

Experimental. Crystals of compound **5a** were obtained by slow evaporation of a pentane/DCM solution. Single clear pale colourless irregular-shaped crystals of compound **5a** were used as supplied. A suitable crystal with dimensions 0.45 × 0.14 × 0.10 mm^3 was selected and mounted on a SuperNova, Dual, Cu at home/near, AtlasS2 diffractometer. The crystal was kept at a steady T = 140.00(10) K during data collection. The structure was solved with the **ShelXT** (Sheldrick, 2015) solution program using dual methods and by using **Olex2** 1.5 (Dolomanov et al., 2009) as the graphical interface. The model was refined with **ShelXL** 2018/3 (Sheldrick, 2015) using full matrix least squares minimisation on $\boldsymbol{F}^2$.

Crystal Data. $C_{13}H_{14}F_3NO_3$, M_r = 289.25, orthorhombic, $P2_12_12_1$ (No. 19), a = 6.71704(16) Å, b = 11.8971(3) Å, c = 17.2416(4) Å, $\alpha = \beta = \gamma = 90^\circ$, V = 1377.83(6) Å^3, T = 140.00(10) K, Z = 4, Z' = 1, μ (Cu K$_\alpha$) = 1.091, 15019 reflections measured, 2864 unique (R_{int} = 0.0583) which were used in all calculations. The final wR_2 was 0.1132 (all data) and R_1 was 0.0419 (I≥2 σ(I)).

**Compound**	**5a**
Formula	$C_{13}H_{14}F_3NO_3$
$D_{calc.}$/ g cm^{-3}	1.394
μ/mm^{-1}	1.091
Formula Weight	289.25
Colour	clear pale colourless
Shape	irregular-shaped
Size/mm^3	0.45×0.14×0.10
T/K	140.00(10)
Crystal System	orthorhombic
Flack Parameter	0.03(19)
Hooft Parameter	-0.01(9)
Space Group	$P2_12_12_1$
a/Å	6.71704(16)
b/Å	11.8971(3)
c/Å	17.2416(4)
α/°	90
β/°	90
γ/°	90
V/Å^3	1377.83(6)
Z	4
Z'	1
Wavelength/Å	1.54184
Radiation type	Cu K$_\alpha$
Θ_{min}/°	4.515
Θ_{max}/°	76.231
Measured Refl's.	15019
Indep't Refl's	2864
Refl's I≥2 σ(I)	2639
R_{int}	0.0583
Parameters	187
Restraints	0
Largest Peak	0.265
Deepest Hole	-0.219
GooF	1.078
wR_2 (all data)	0.1132
wR_2	0.1100
R_1 (all data)	0.0451
R_1	0.0419

G. Proposed Reaction Mechanism

Two reaction pathways are considered as plausible reaction mechanisms (Pathway **A** and **B**). In both cases, the catalytic cycle is proposed to start by an aminopalladation step which leads to intermediate **II** or **III** depending on protonation state of the carbamate group. Here we depict aminopalladation (**II**) followed by deprotonation (**III**). The Pd^{II}-mediated reversible aminopalladation step of alkene **1** can occur via an *anti* (pathway **A**) or a *syn* pathway (pathway **B**) generating protonated Pd^{II} intermediate **II**. In both cases, the observed configuration at the carbon next to the CF_3 group can be rationalized by assuming a pseudo-axial position to avoid $A^{1,3}$ interactions with the carbamate group. Following an irreversible deprotonation step, intermediate **III** is believed to undergo oxidation in the presence of PIDA giving a high-valent alkyl-Pd^{IV} intermediate **IV.** Finally, S_N2-type displacement by an acetate group (pathway **A**) or reductive elimination (pathway **B**) from the Pd^{IV} center would afford the observed aminoacetoxylated product **2** and regenerate the catalyst. Additionally, different reaction outcomes (**2u** and **2t**) observed with non-aromatic derived substrates could be explained by a competing β-hydride elimination process from intermediate **III** or **IV**.

Scheme S1 Tentative mechanism proposal.

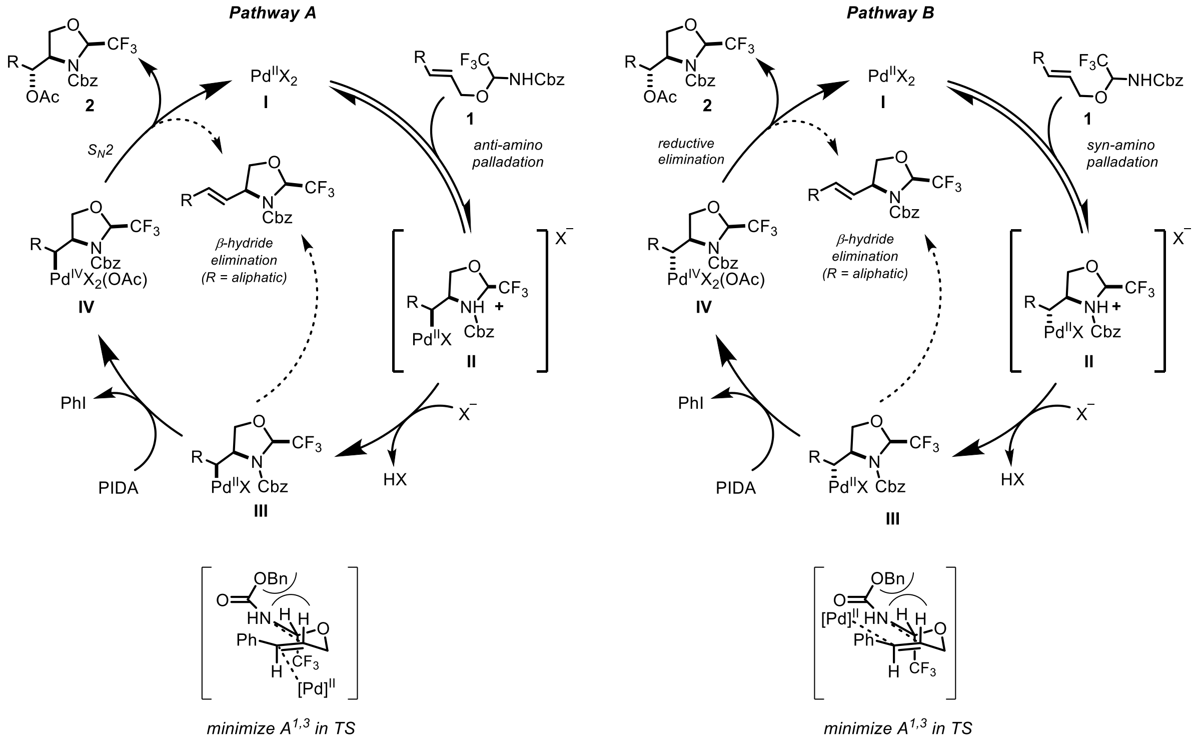

H. NMR Spectra

^{1}H NMR (400 MHz, $CDCl_3$)

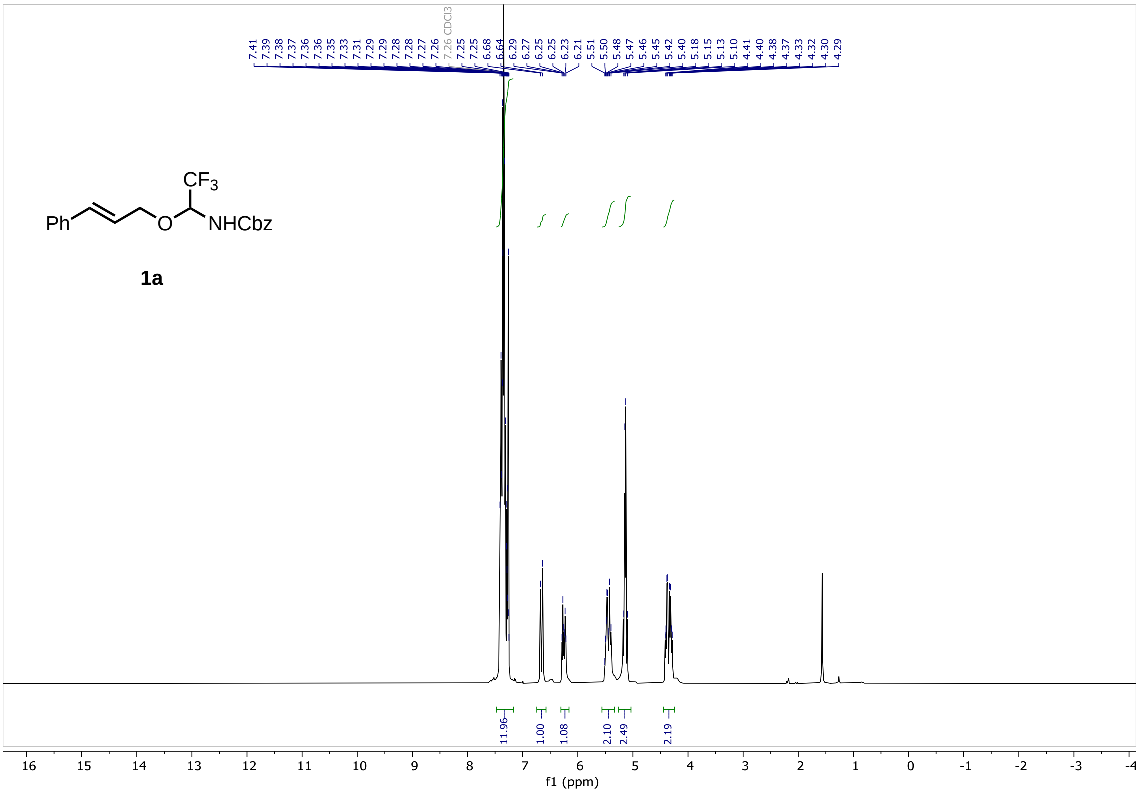

^{13}C NMR (101 MHz, $CDCl_3$)

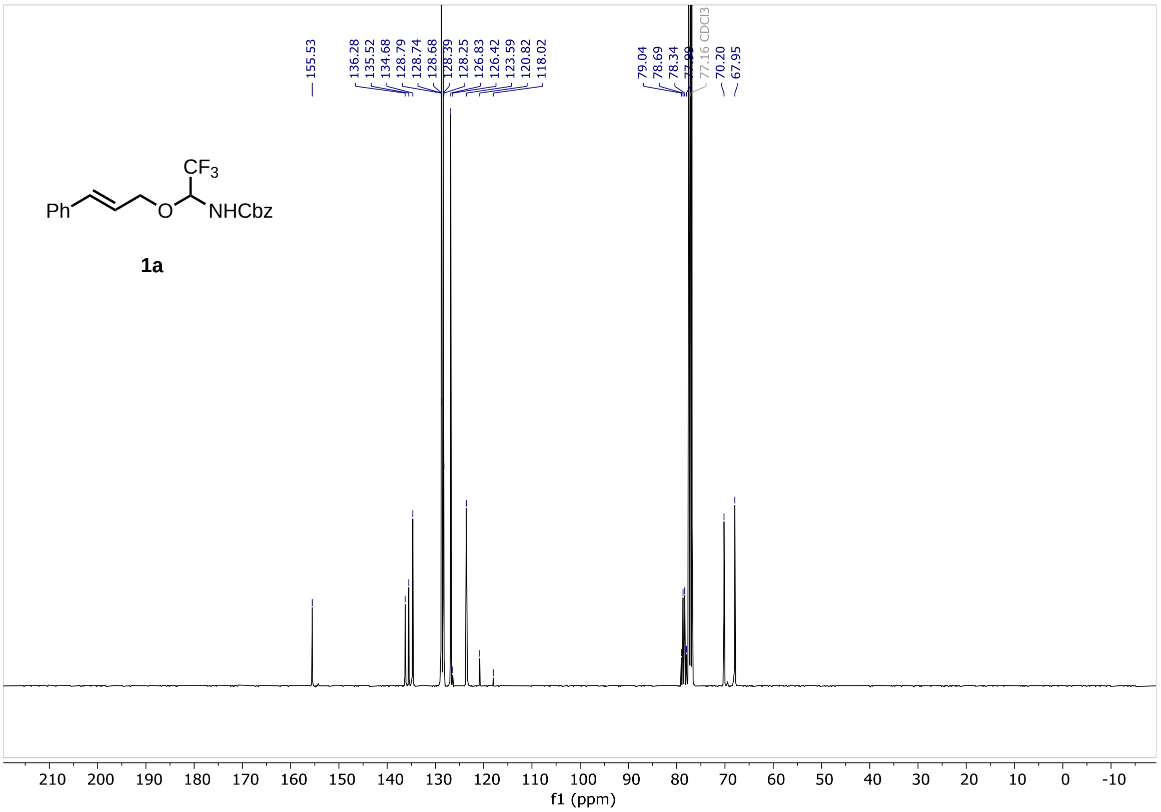

^{19}F NMR (376 MHz, $CDCl_3$)

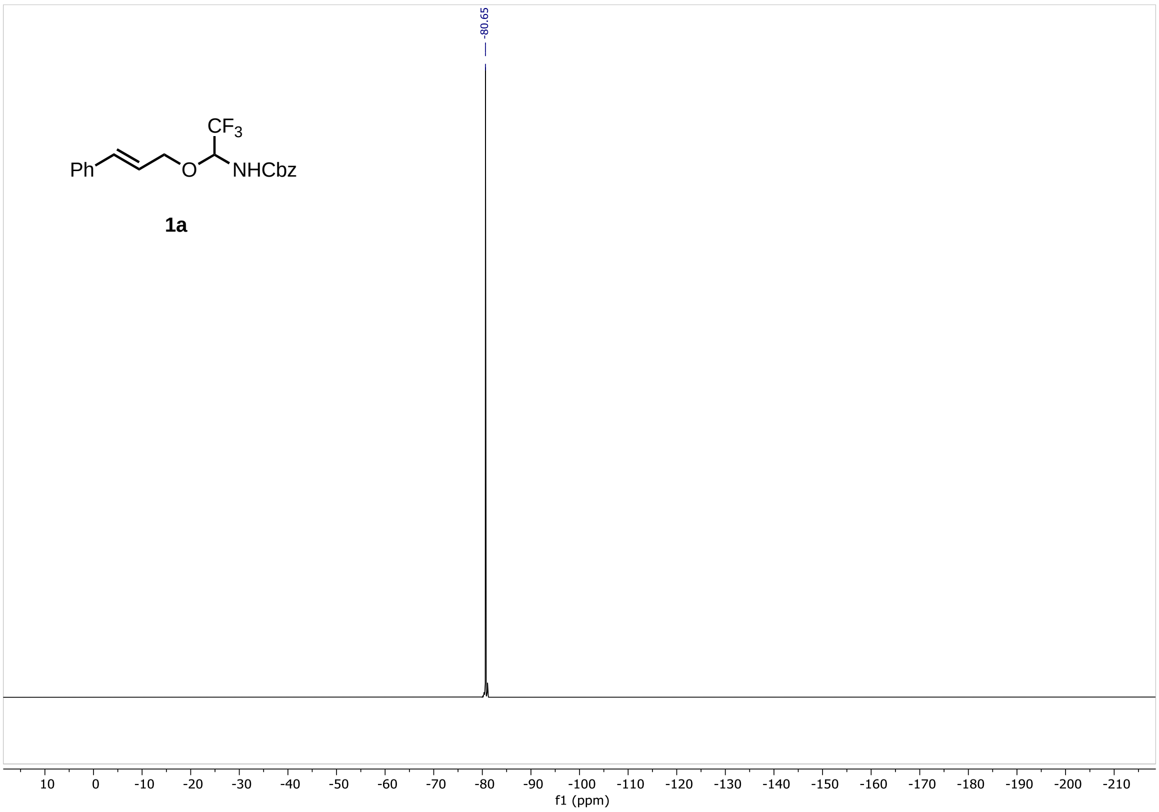

^{1}H NMR (400 MHz, $CDCl_3$)

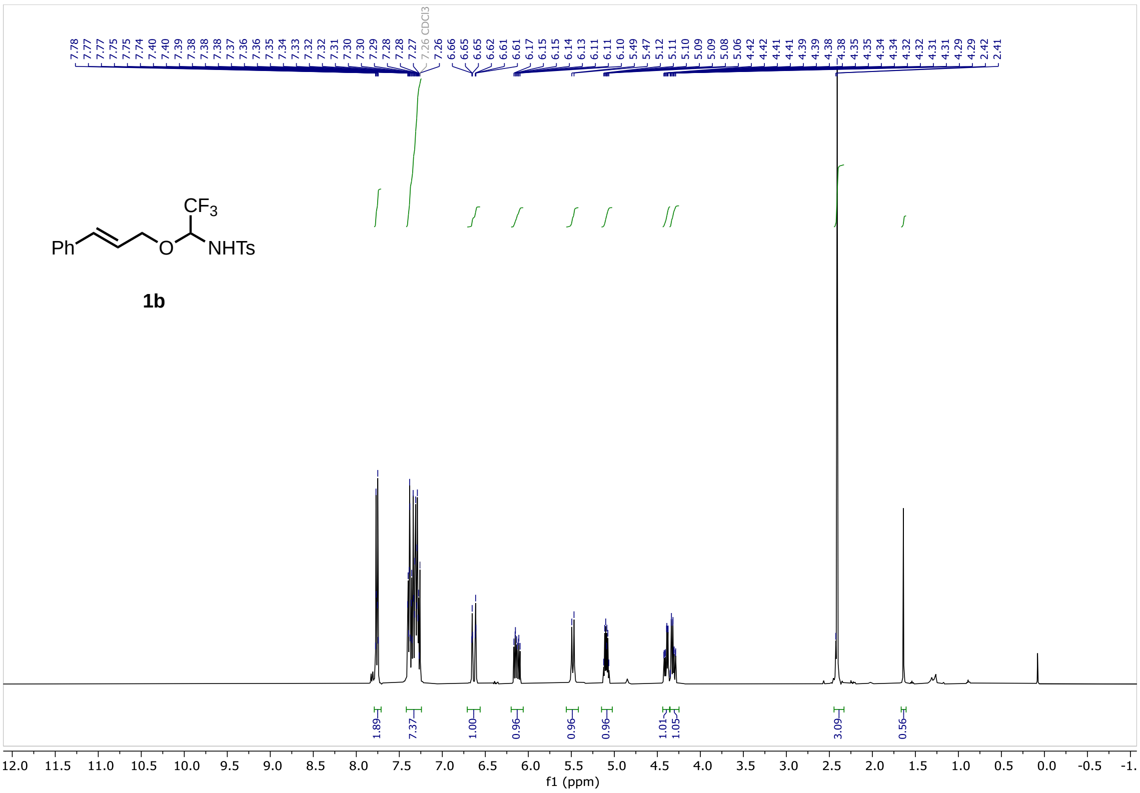

^{13}C NMR (101 MHz, $CDCl_3$)

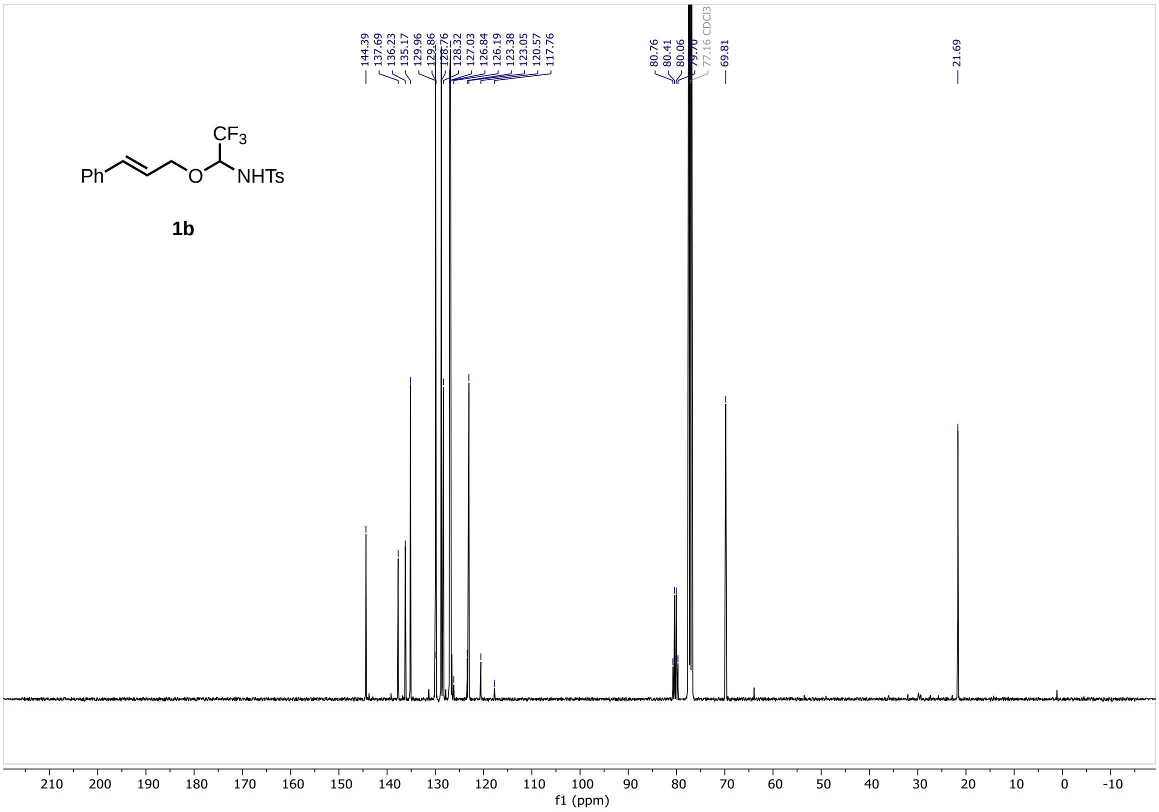

^{19}F NMR (376 MHz, $CDCl_3$)

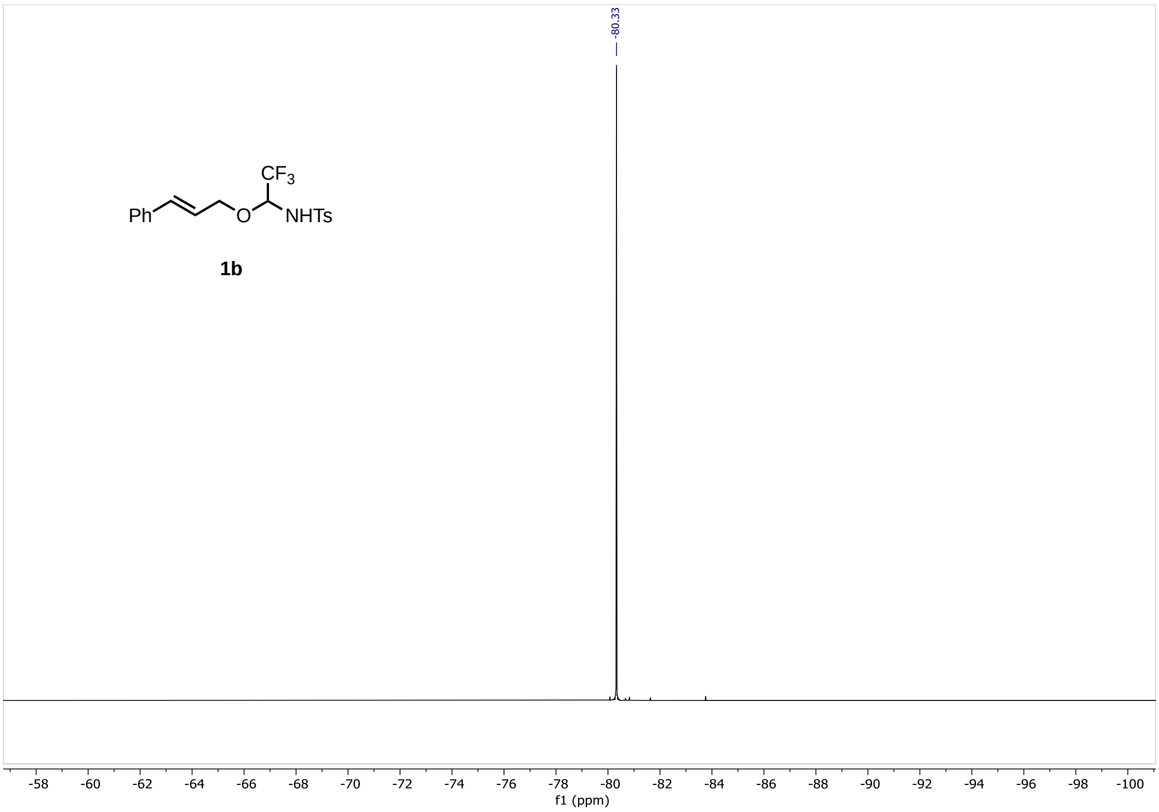

^{1}H NMR (400 MHz, $CDCl_3$)

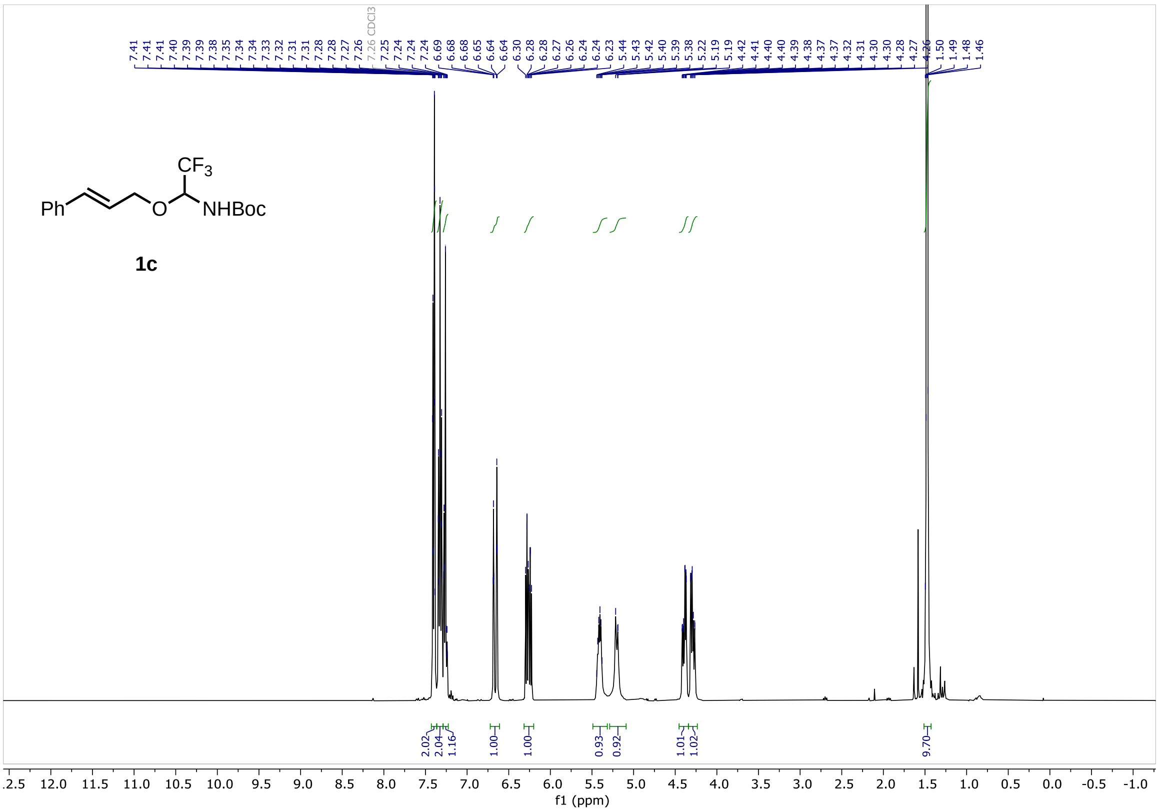

^{13}C NMR (101 MHz, $CDCl_3$)

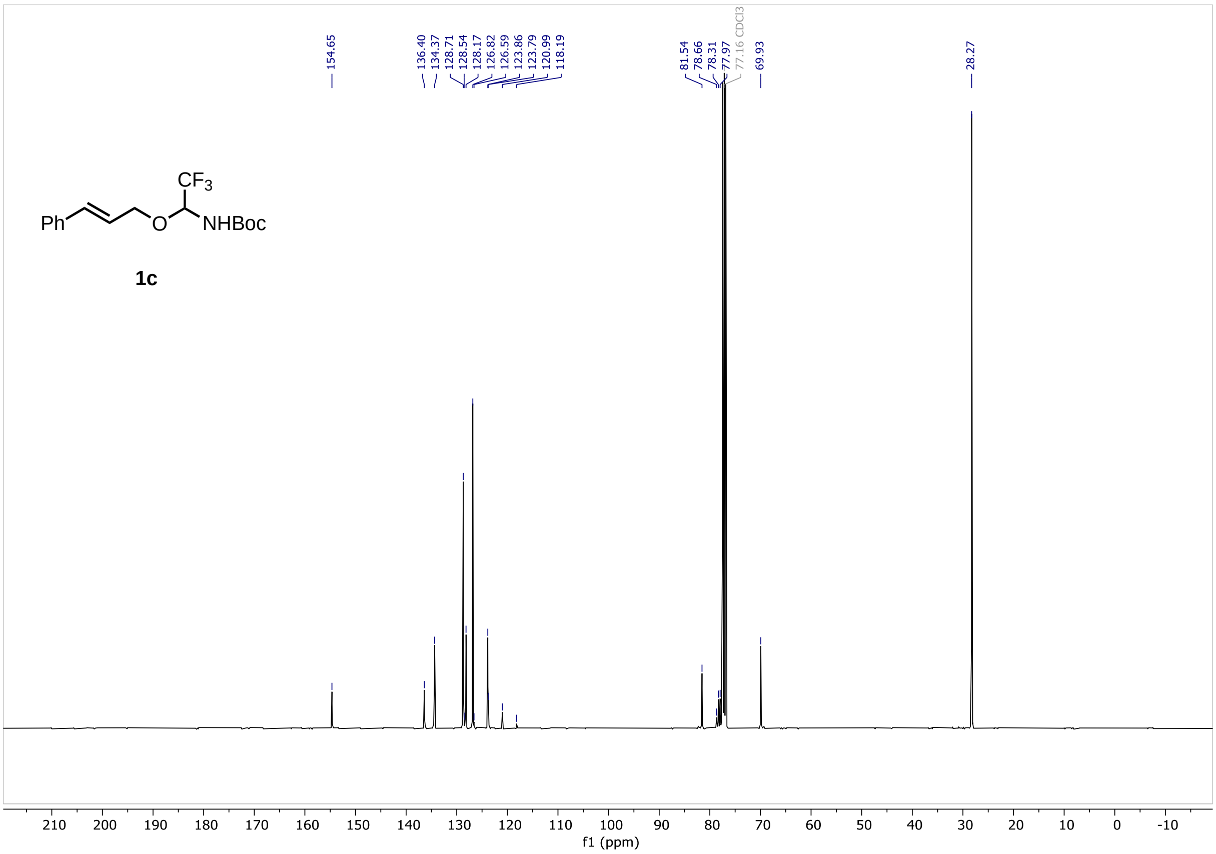

^{19}F NMR (376 MHz, $CDCl_3$)

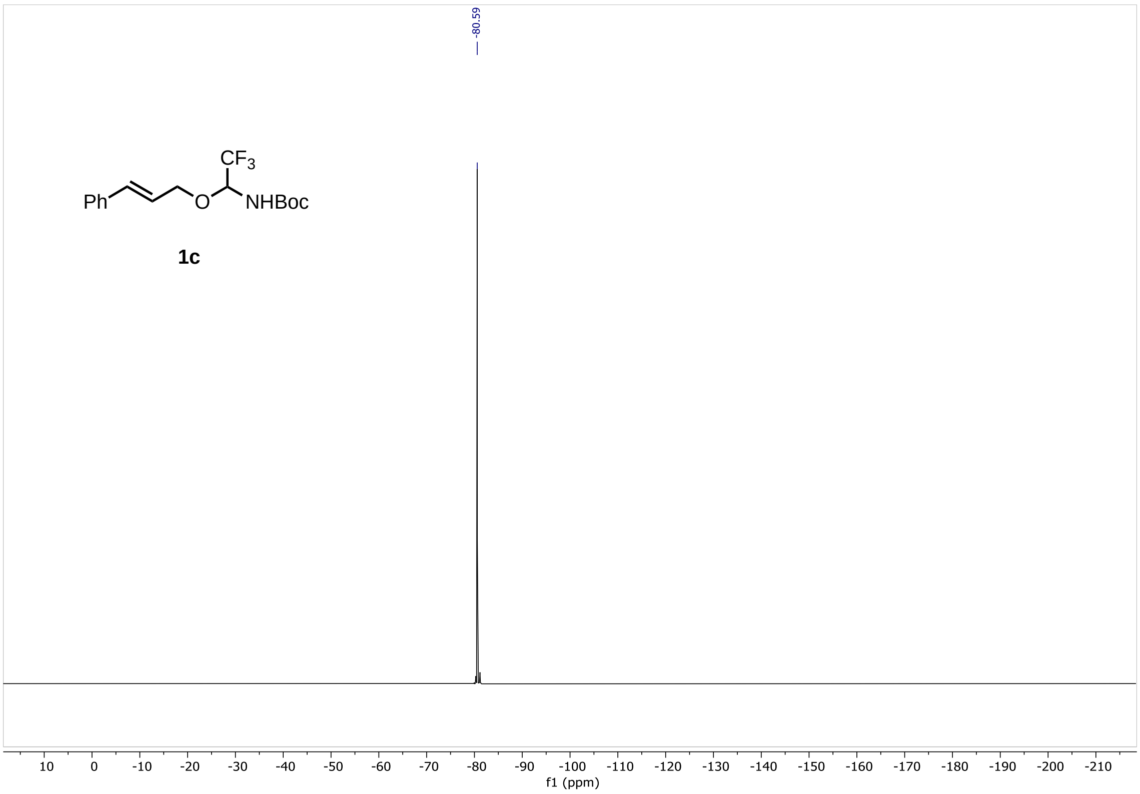

^{1}H NMR (400 MHz, $CDCl_3$)

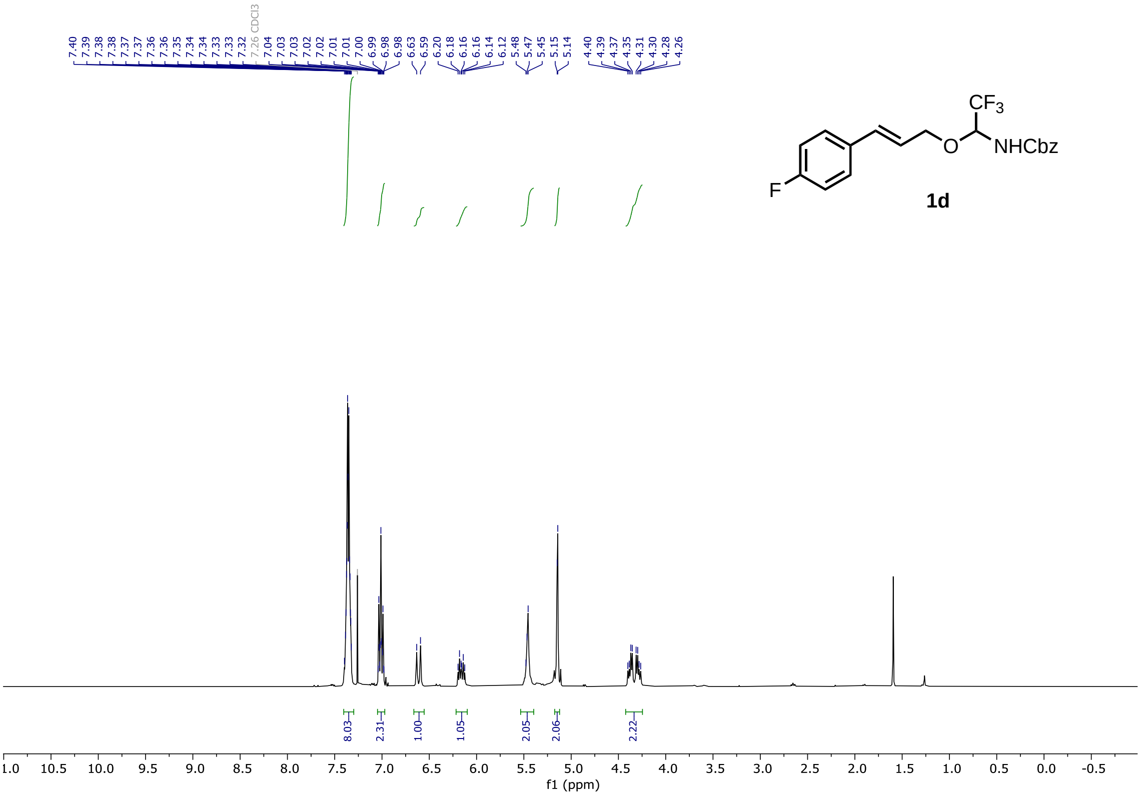

^{13}C NMR (101 MHz, $CDCl_3$)

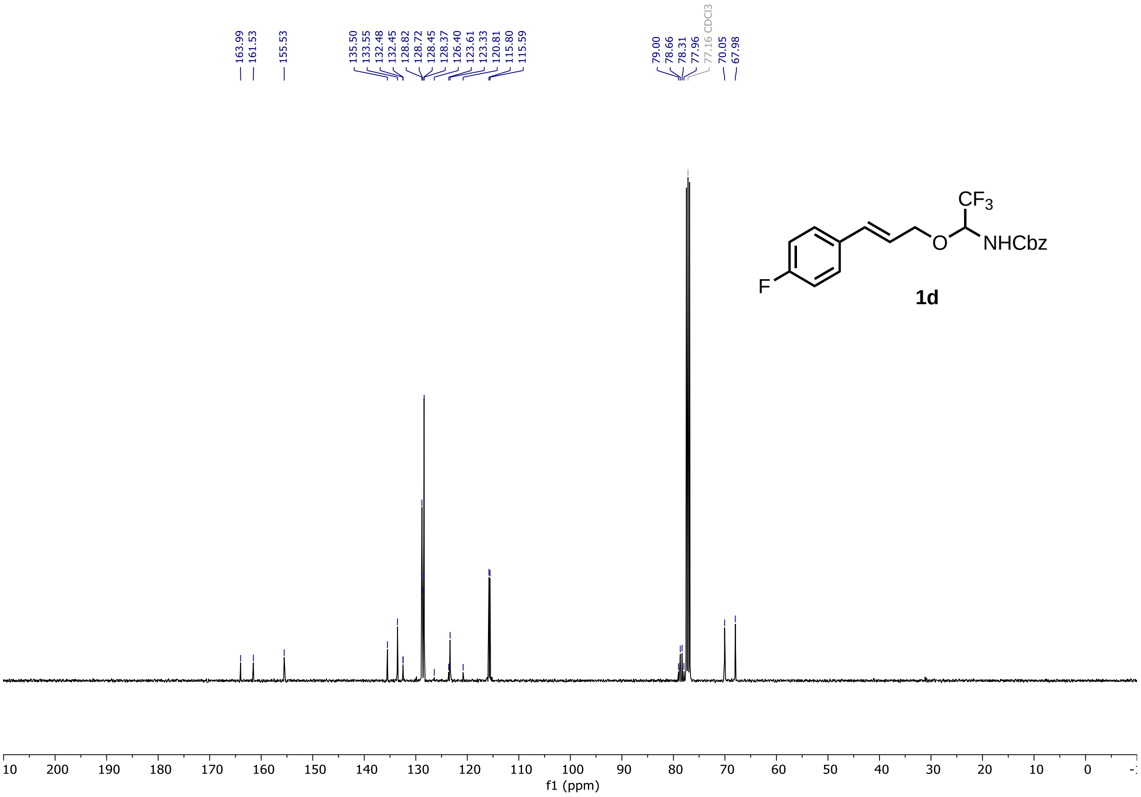

^{19}F NMR (376 MHz, $CDCl_3$)

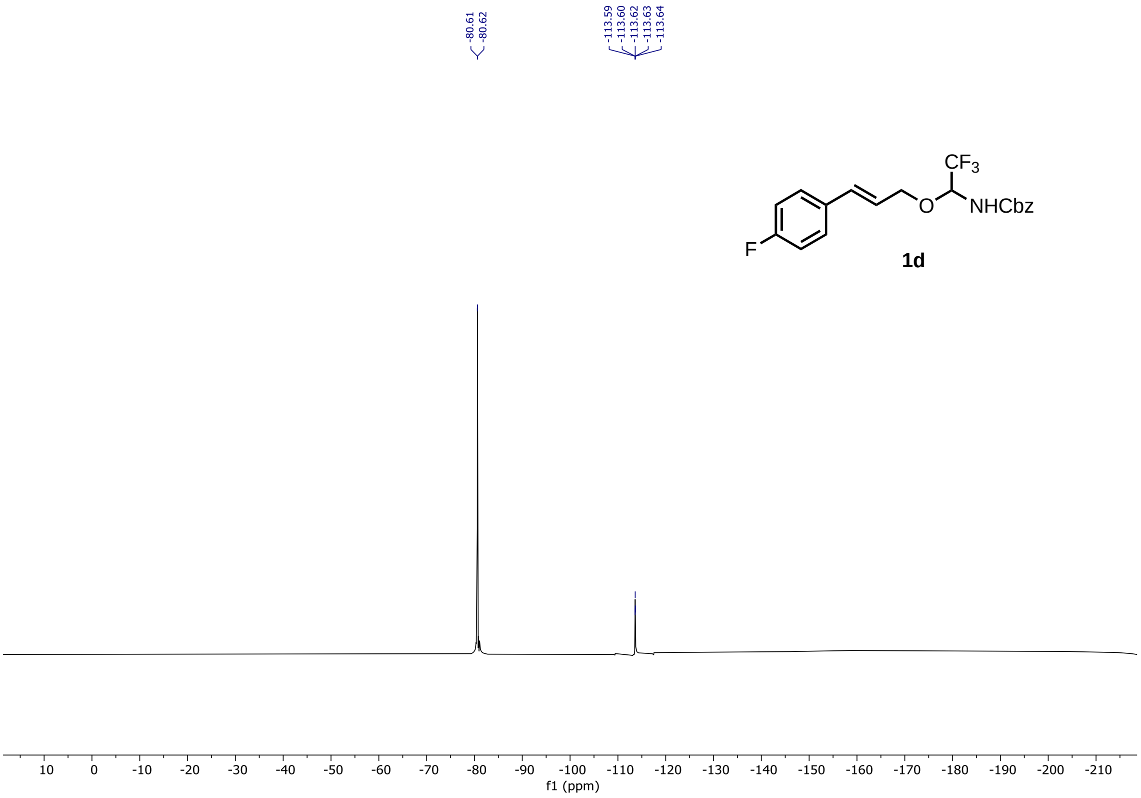

^{1}H NMR (400 MHz, $CDCl_3$)

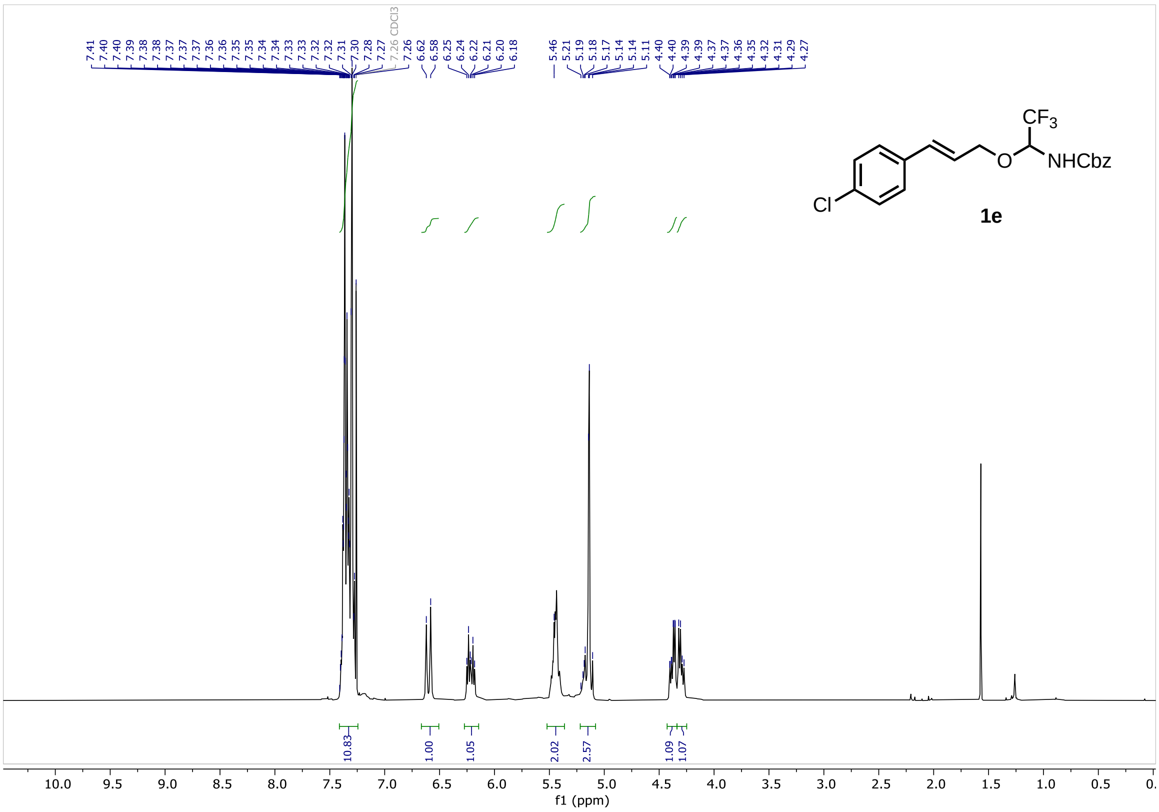

^{13}C NMR (101 MHz, $CDCl_3$)

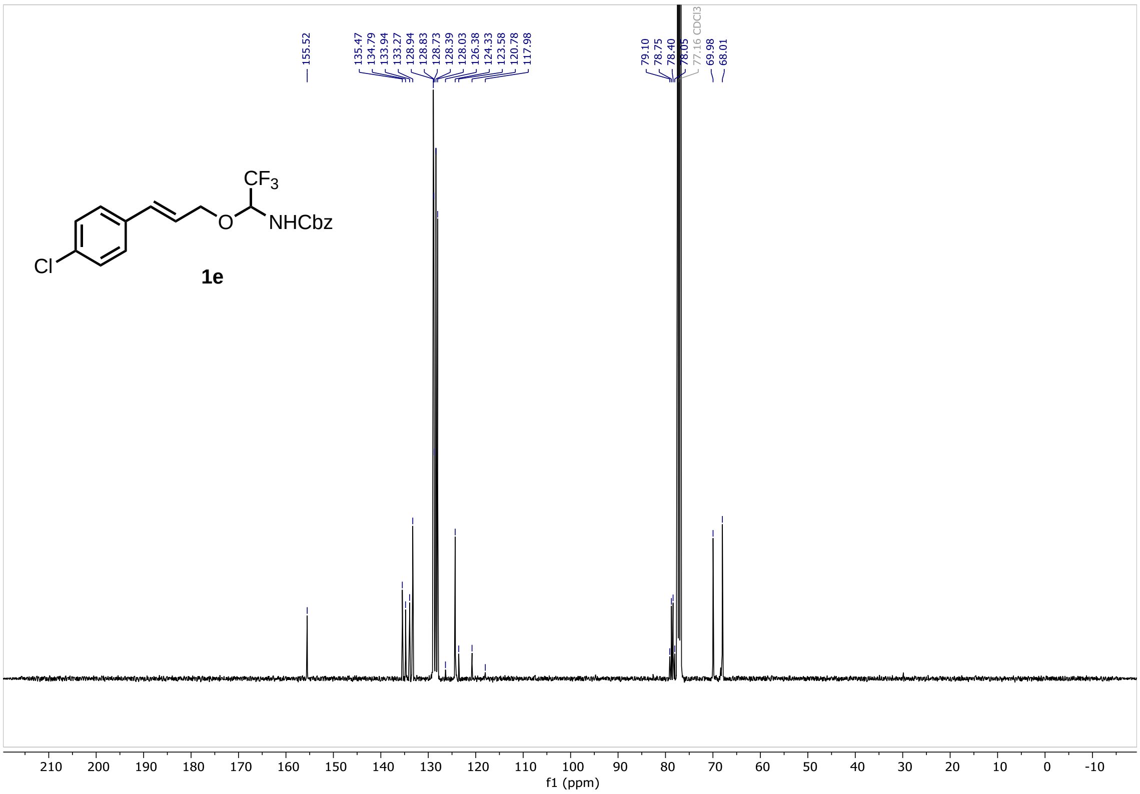

^{19}F NMR (376 MHz, $CDCl_3$)

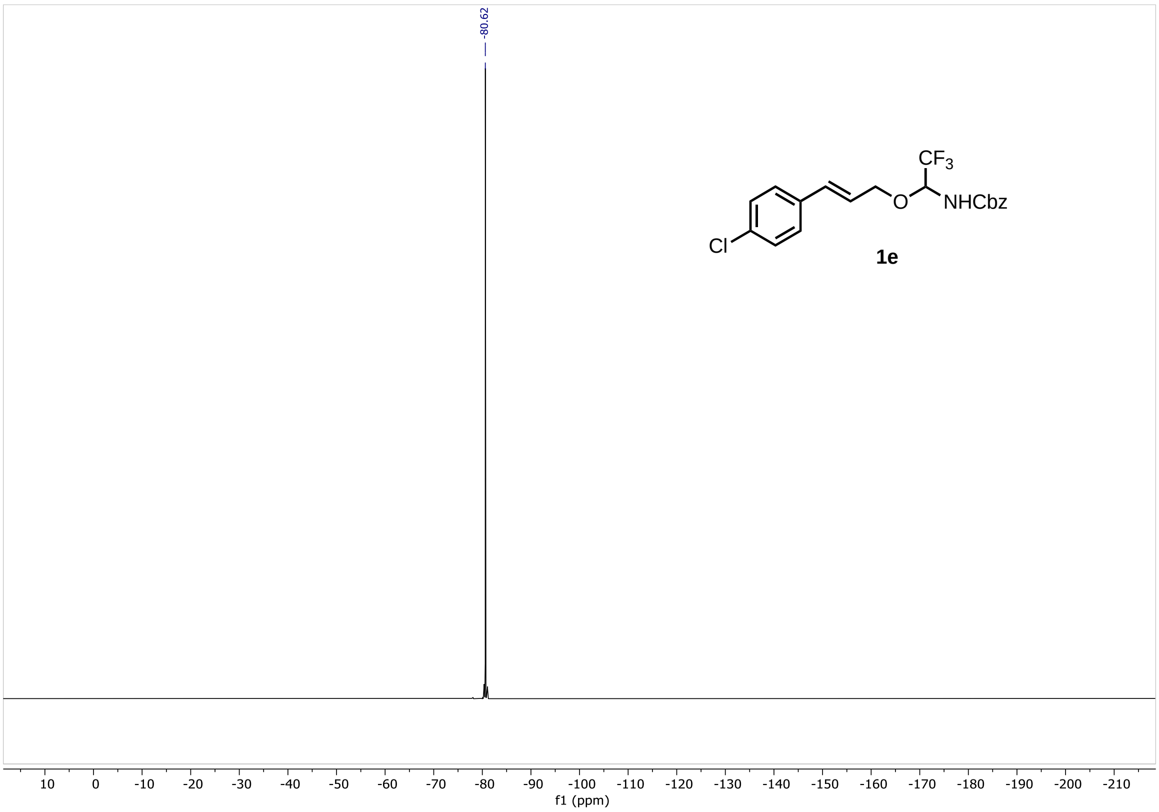

^{1}H NMR (400 MHz, $CDCl_3$)

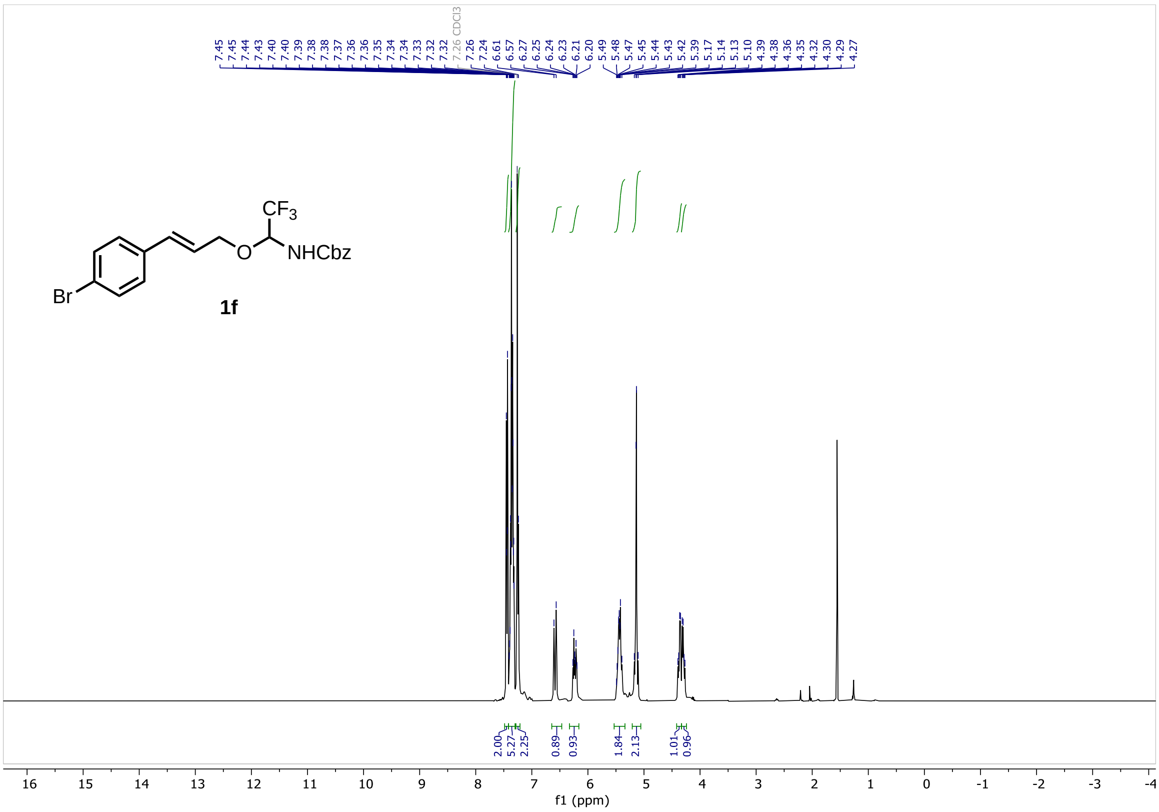

^{13}C NMR (101 MHz, $CDCl_3$)

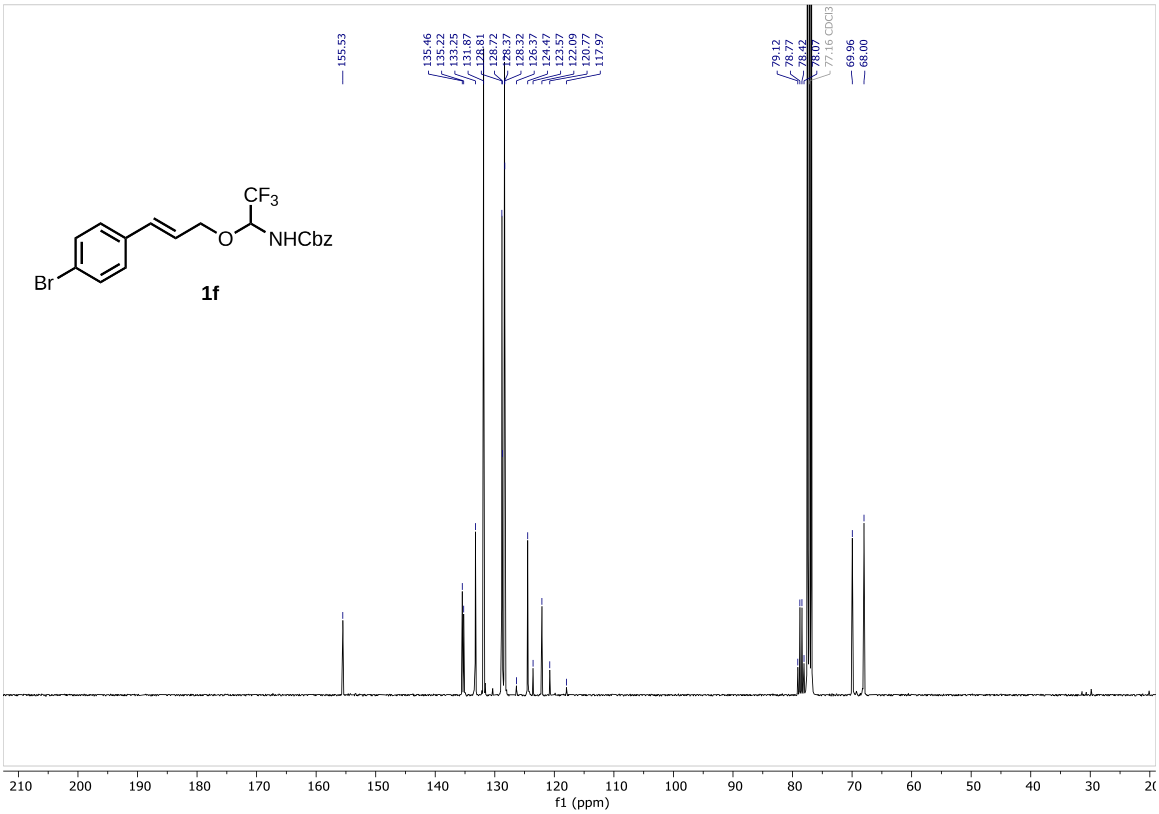

^{19}F NMR (376 MHz, $CDCl_3$)

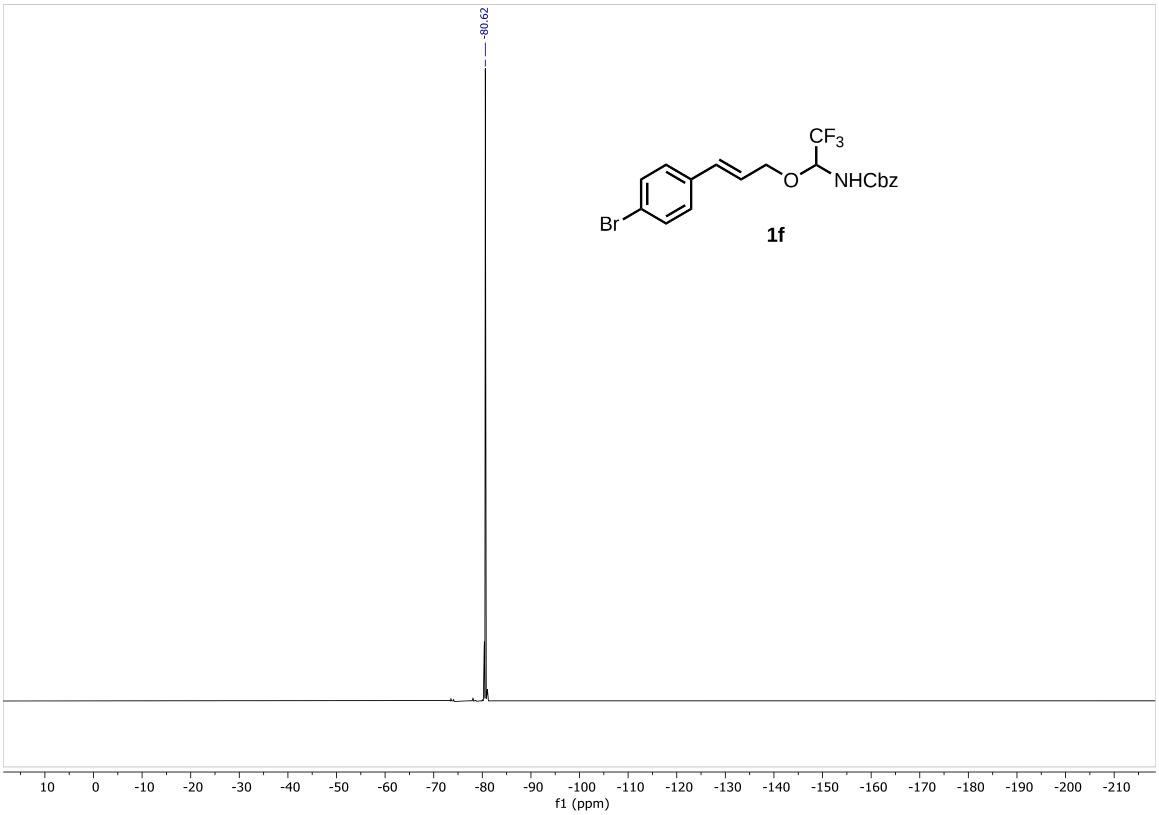

^{1}H NMR (400 MHz, $CDCl_3$)

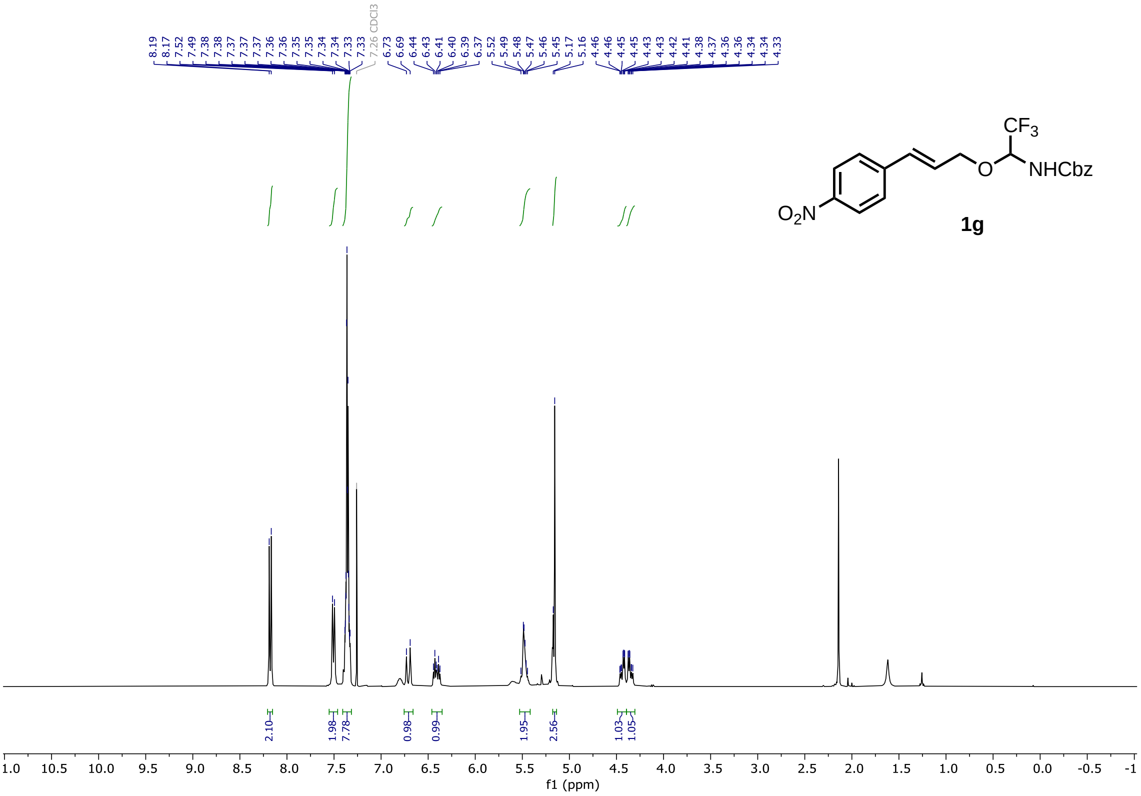

*remnants of coupling partner visible by NMR, yield has been adjusted accordingly.

^{13}C NMR (101 MHz, $CDCl_3$)

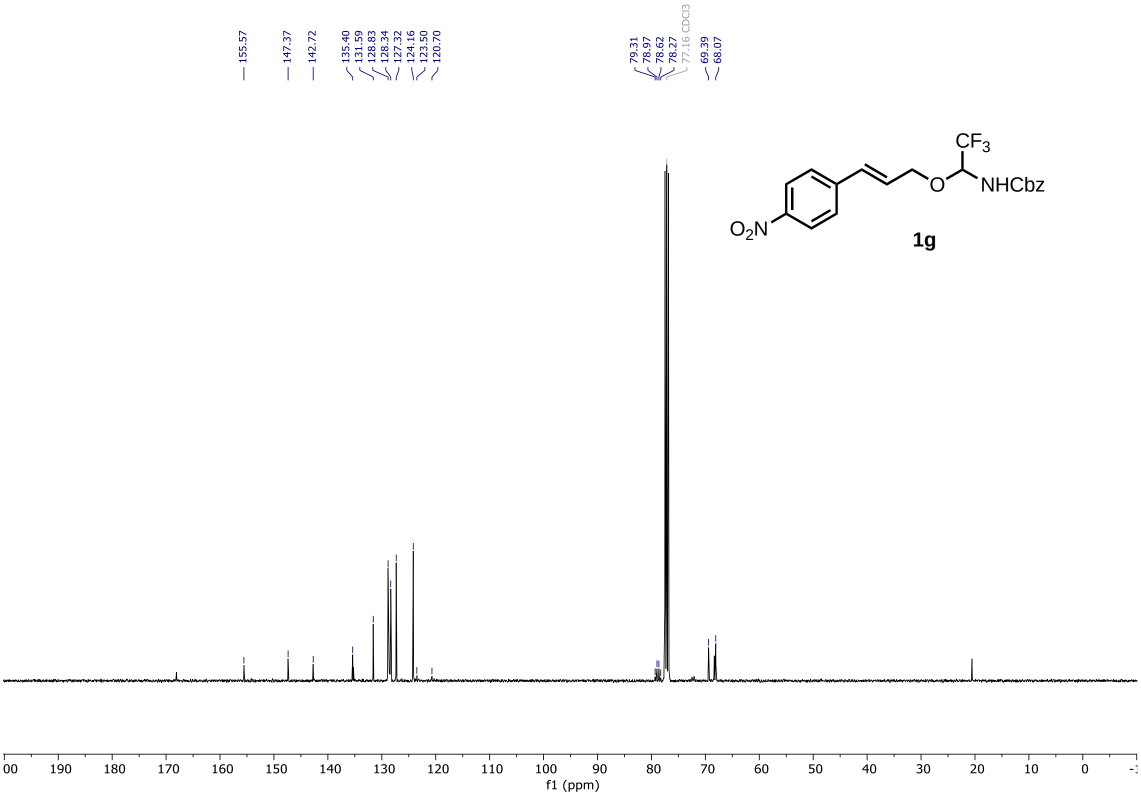

^{19}F NMR (376 MHz, $CDCl_3$)

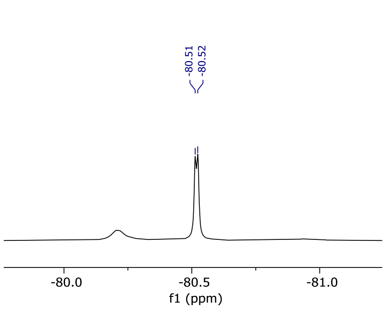

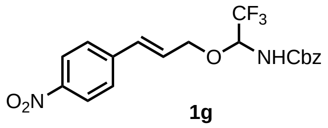

1g

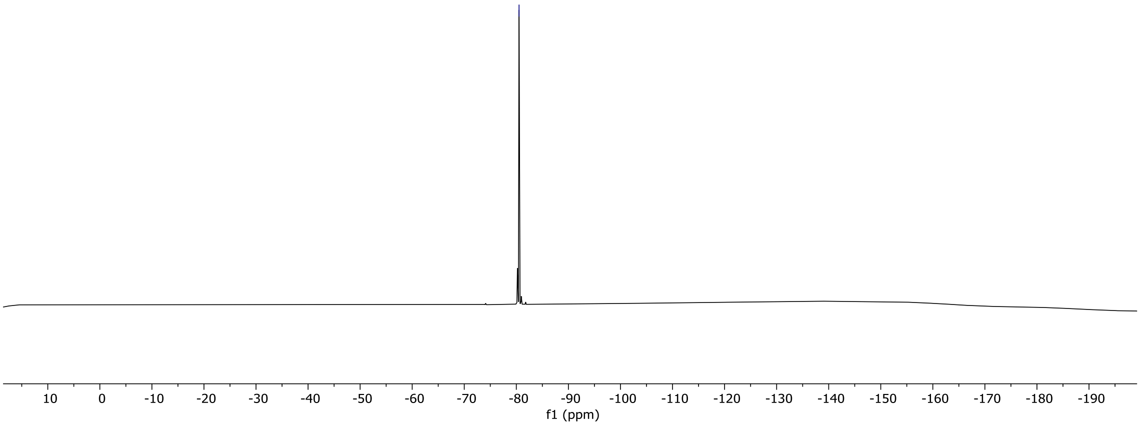

^{1}H NMR (400 MHz, $CDCl_3$)

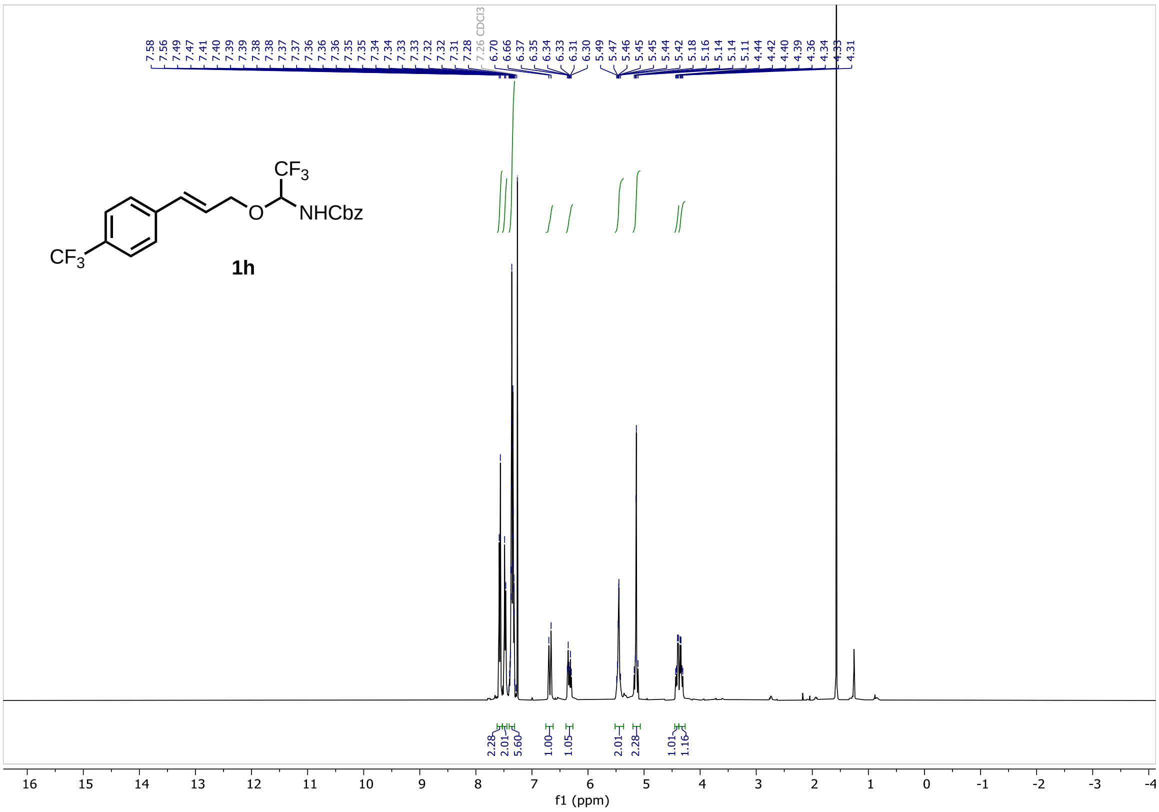

^{13}C NMR (101 MHz, $CDCl_3$)

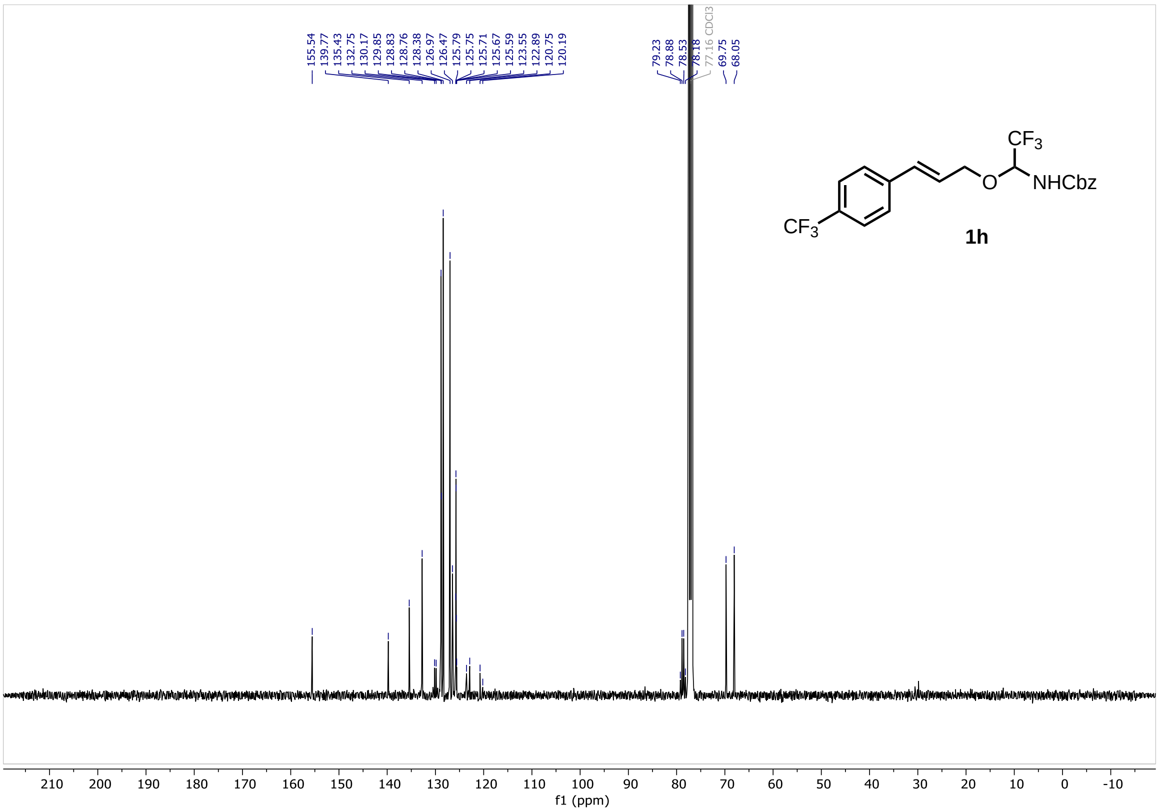

^{19}F NMR (376 MHz, $CDCl_3$)

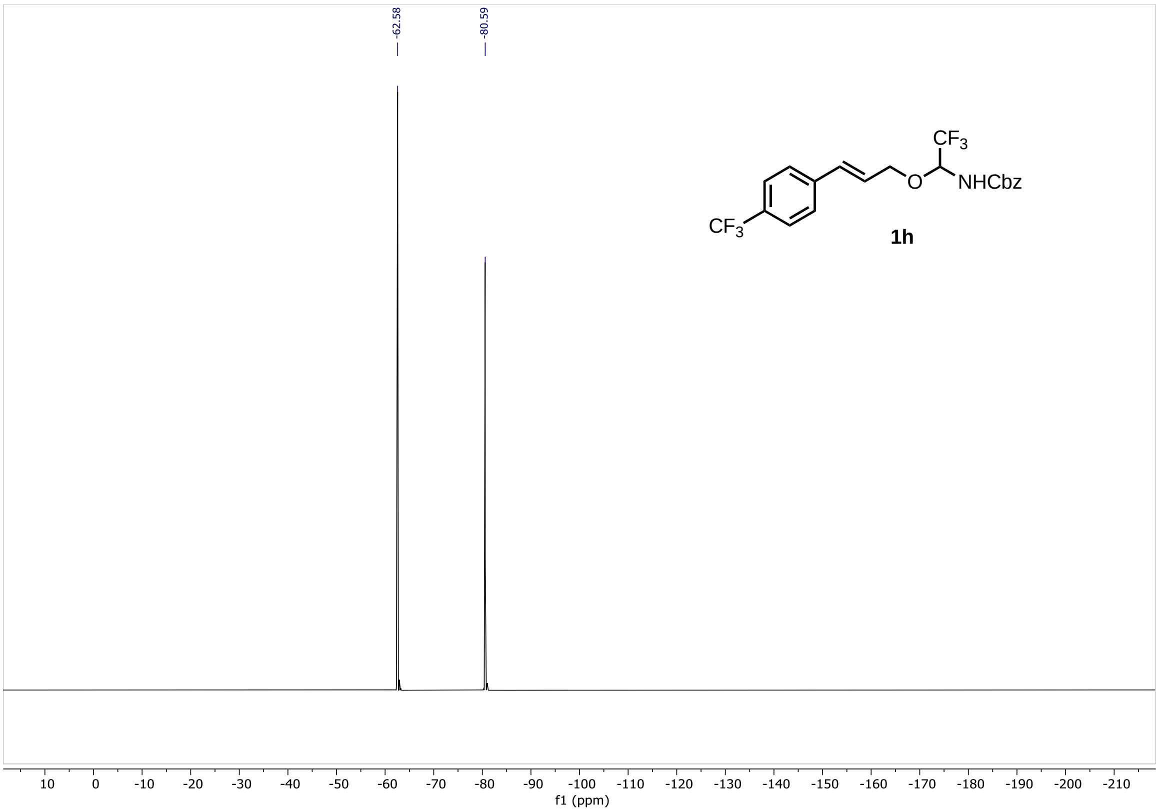

^{1}H NMR (400 MHz, $CDCl_3$)

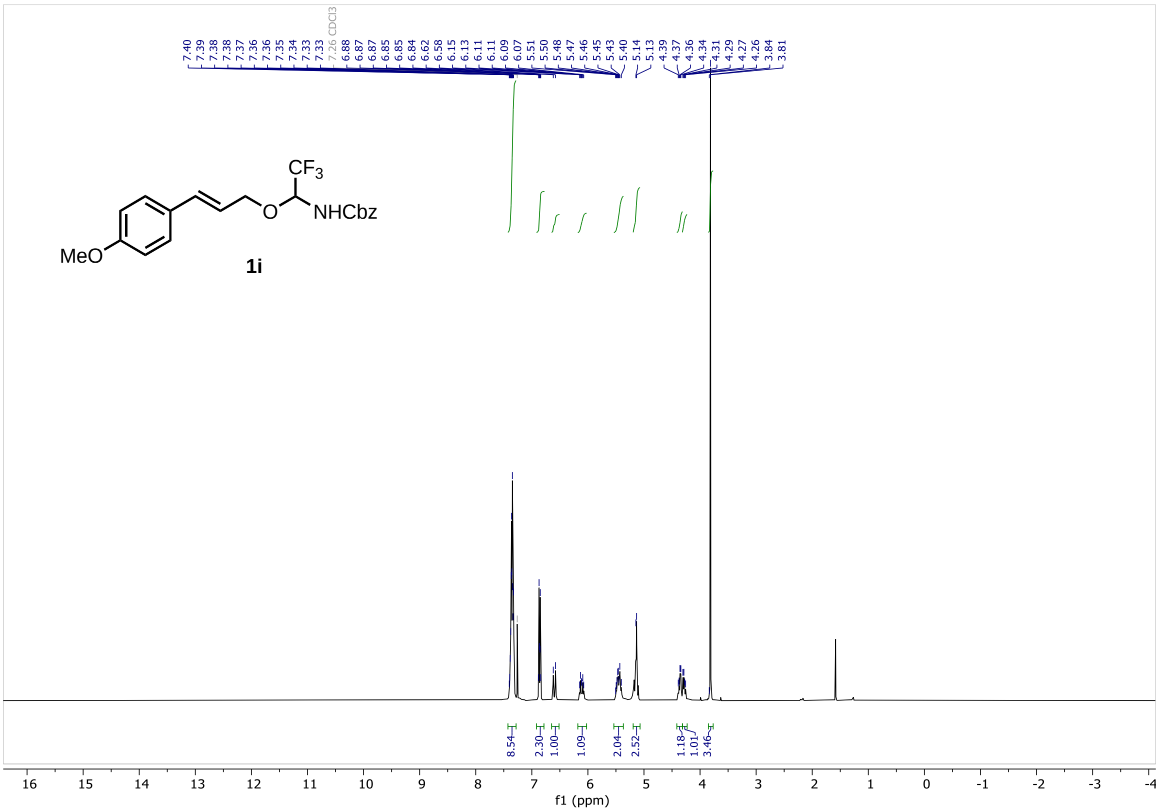

^{13}C NMR (101 MHz, $CDCl_3$)

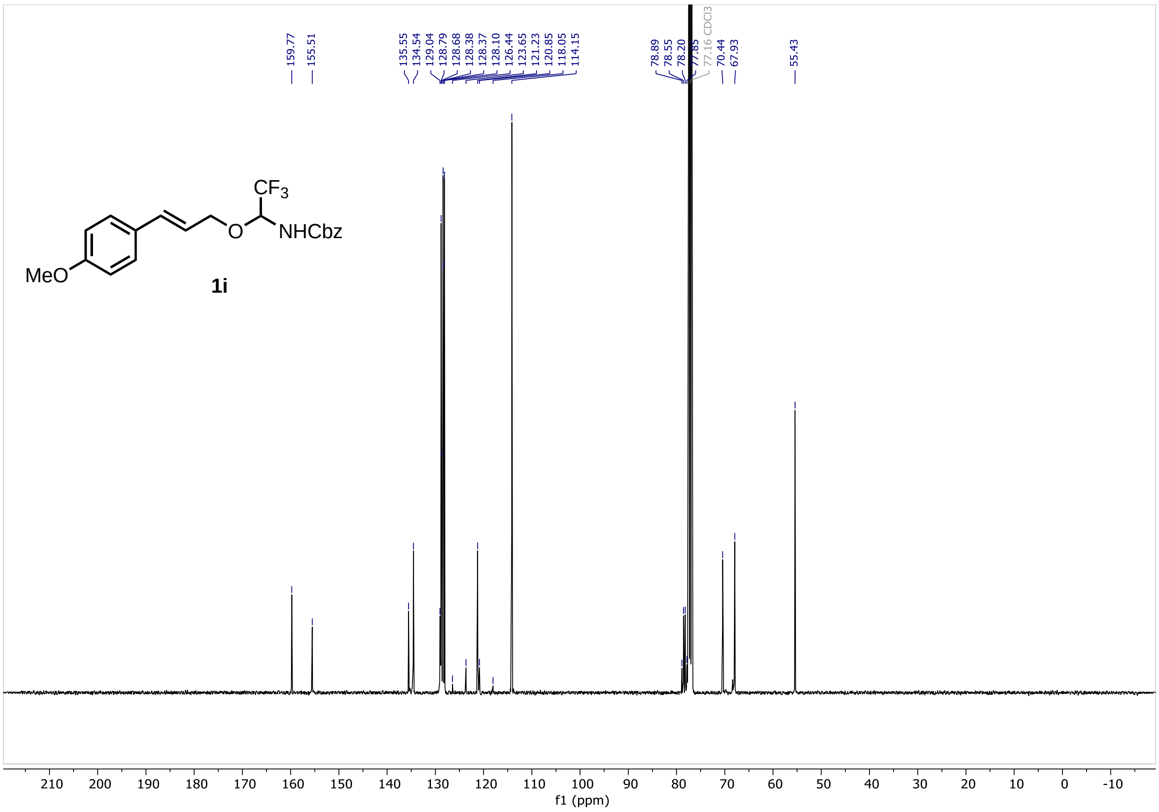

^{19}F NMR (376 MHz, $CDCl_3$)

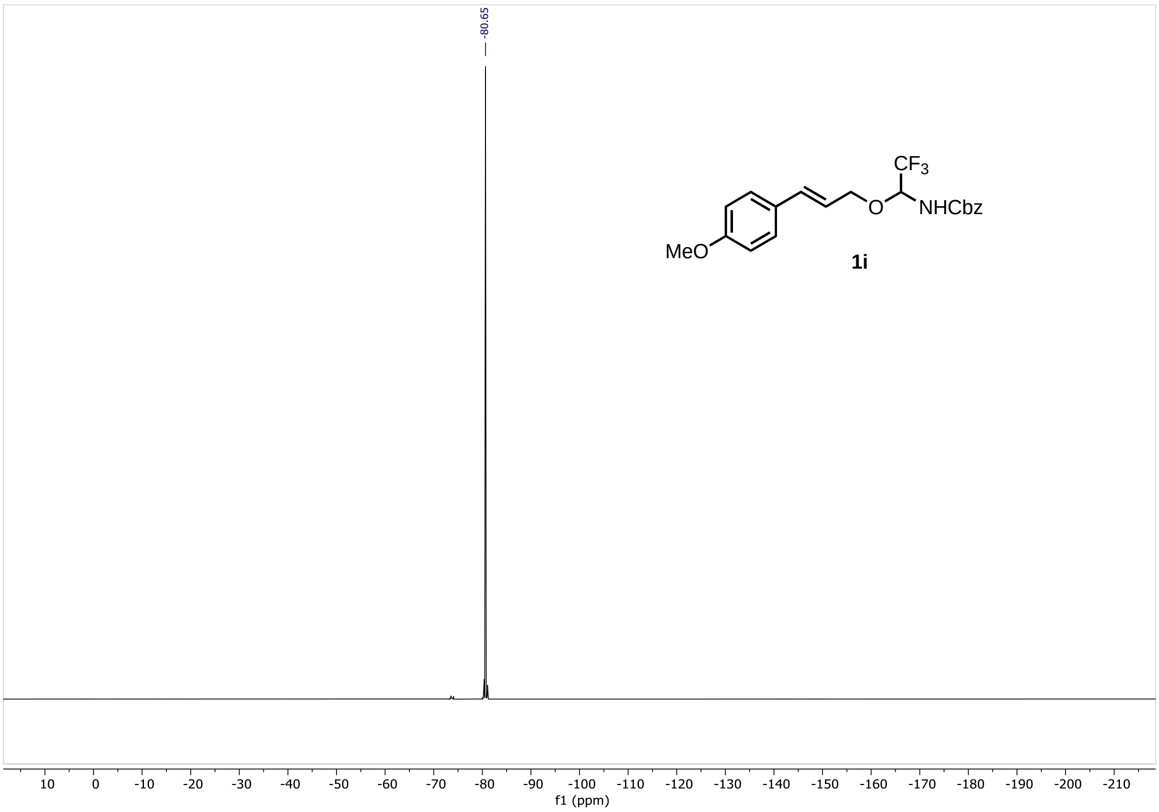

^{1}H NMR (400 MHz, $CDCl_3$)

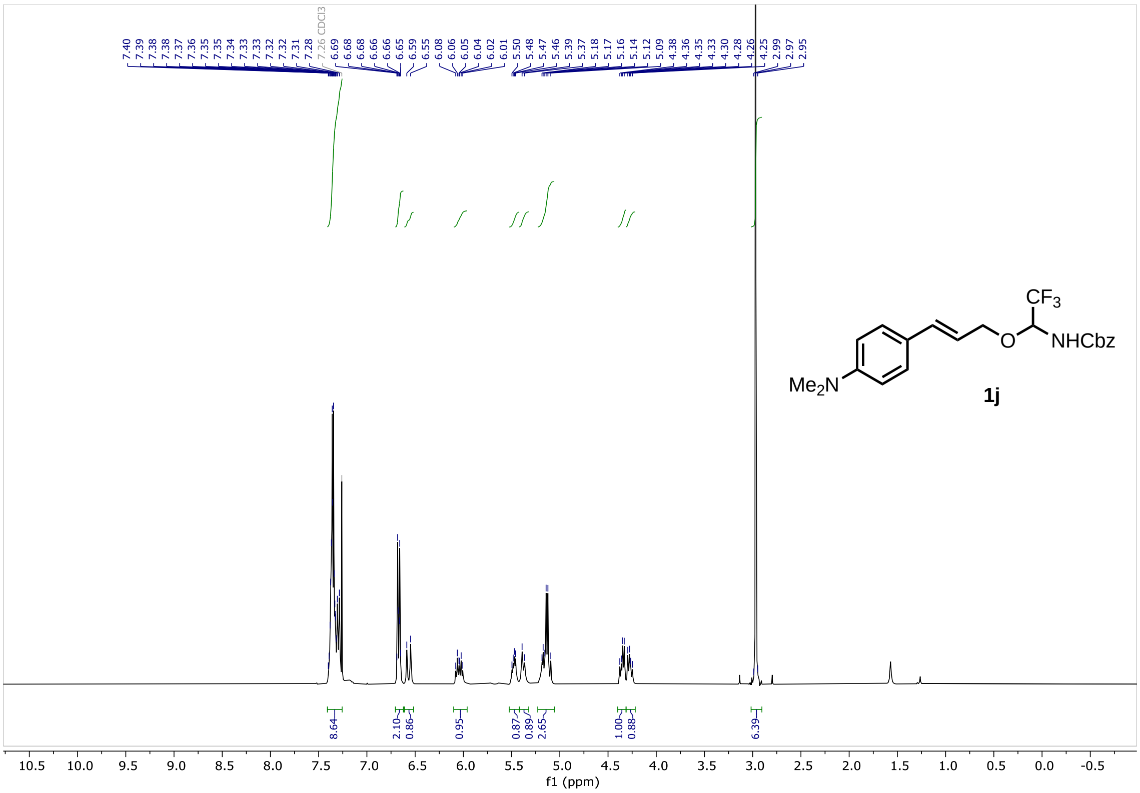

^{13}C NMR (101 MHz, $CDCl_3$)

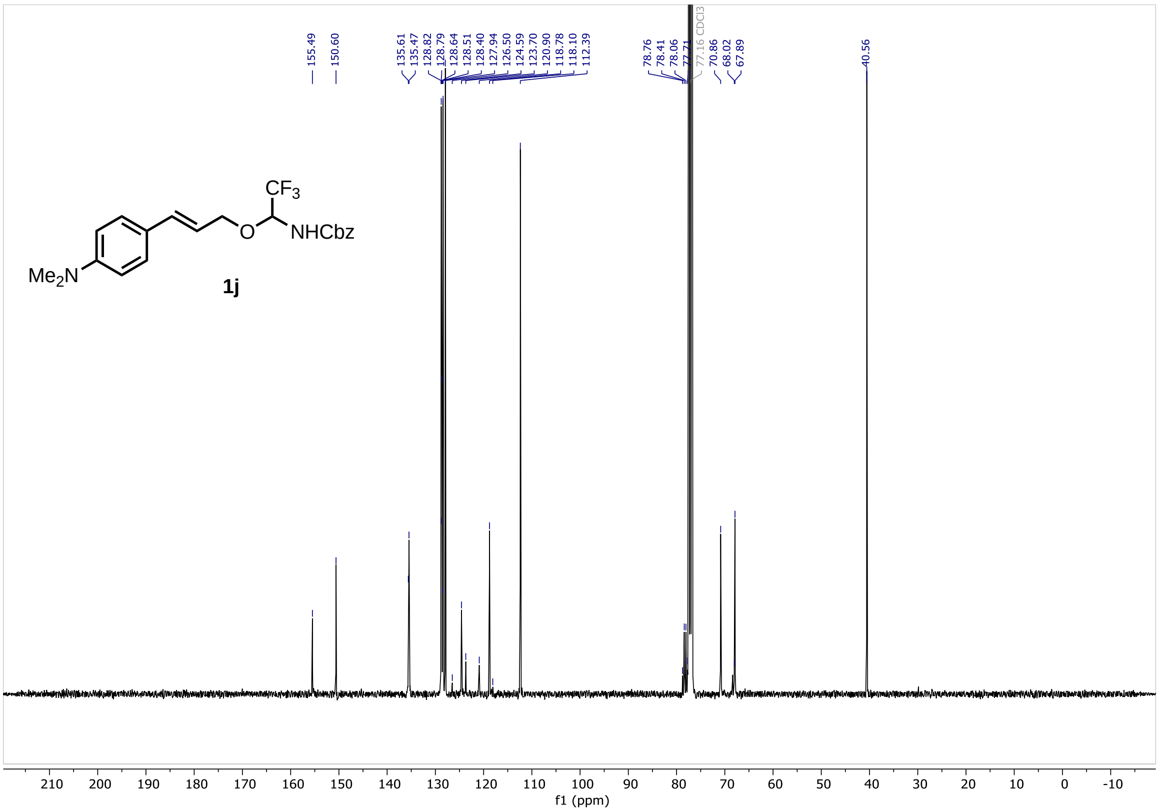

^{19}F NMR (376 MHz, $CDCl_3$)

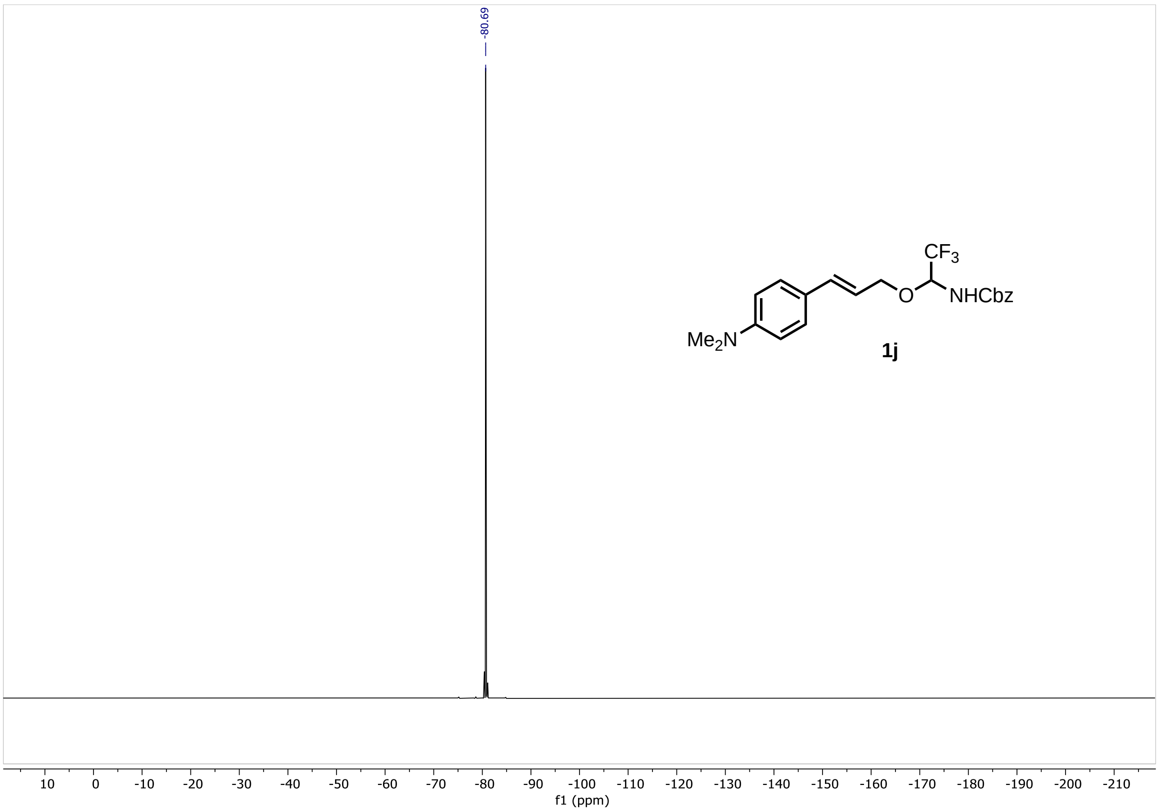

^{1}H NMR (400 MHz, $CDCl_3$)

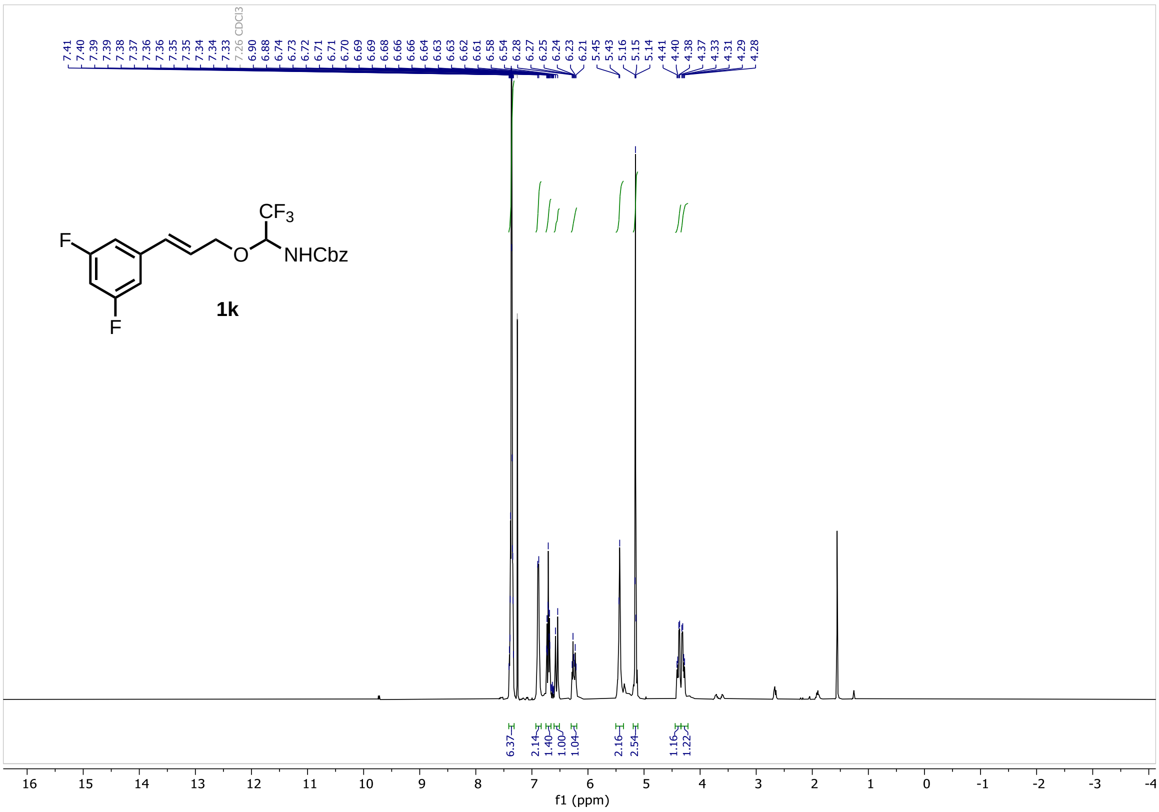

^{13}C NMR (101 MHz, $CDCl_3$)

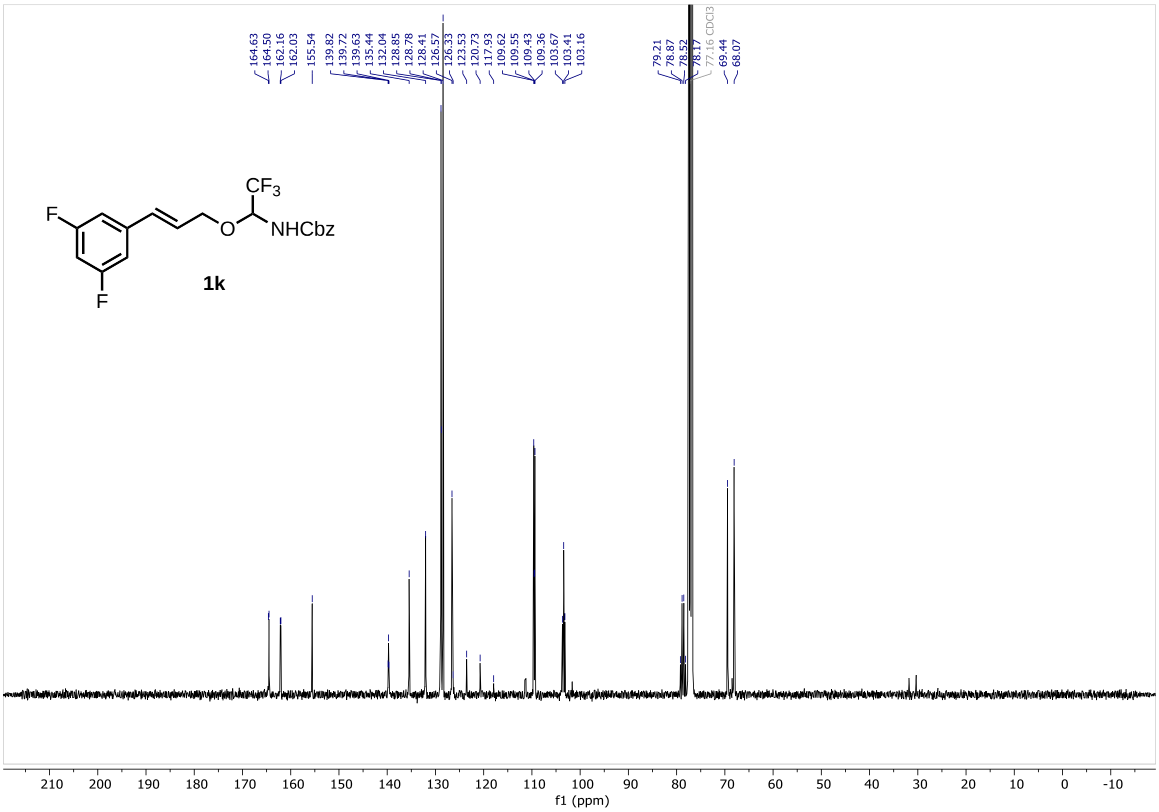

^{19}F NMR (376 MHz, $CDCl_3$)

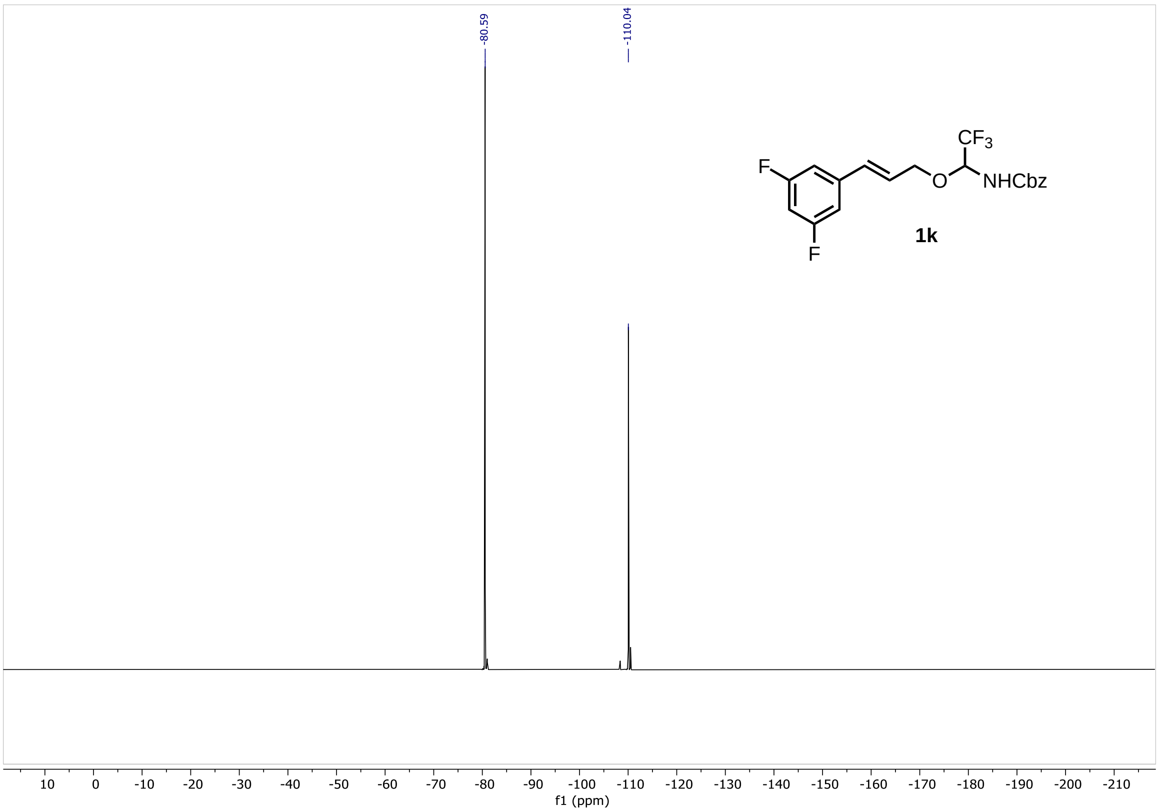

^{1}H NMR (400 MHz, $CDCl_3$)

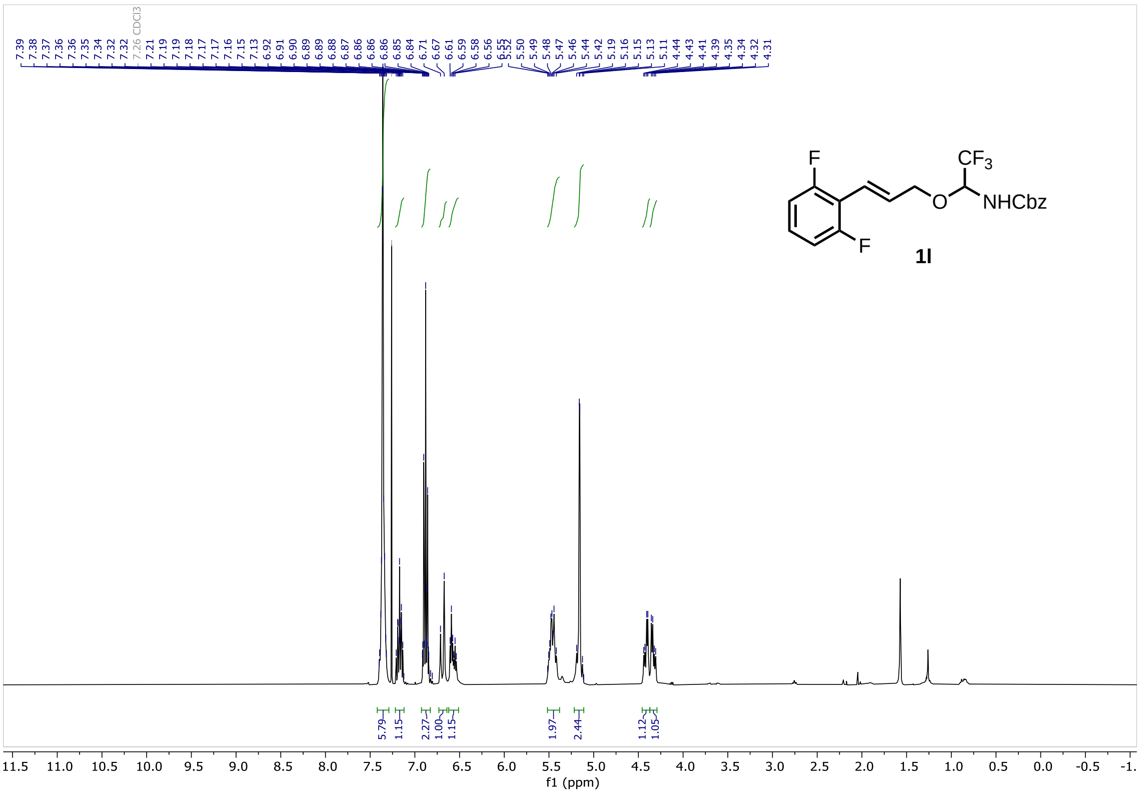

^{13}C NMR (101 MHz, $CDCl_3$)

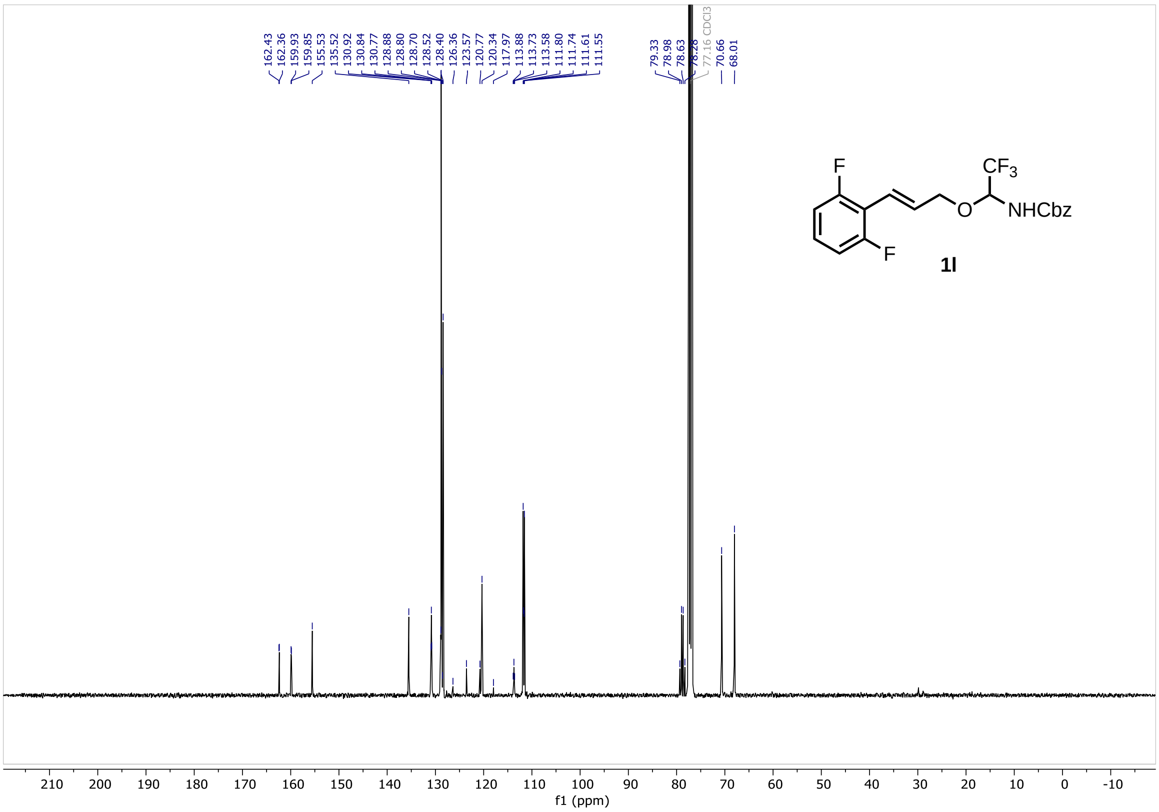

^{19}F NMR (376 MHz, $CDCl_3$)

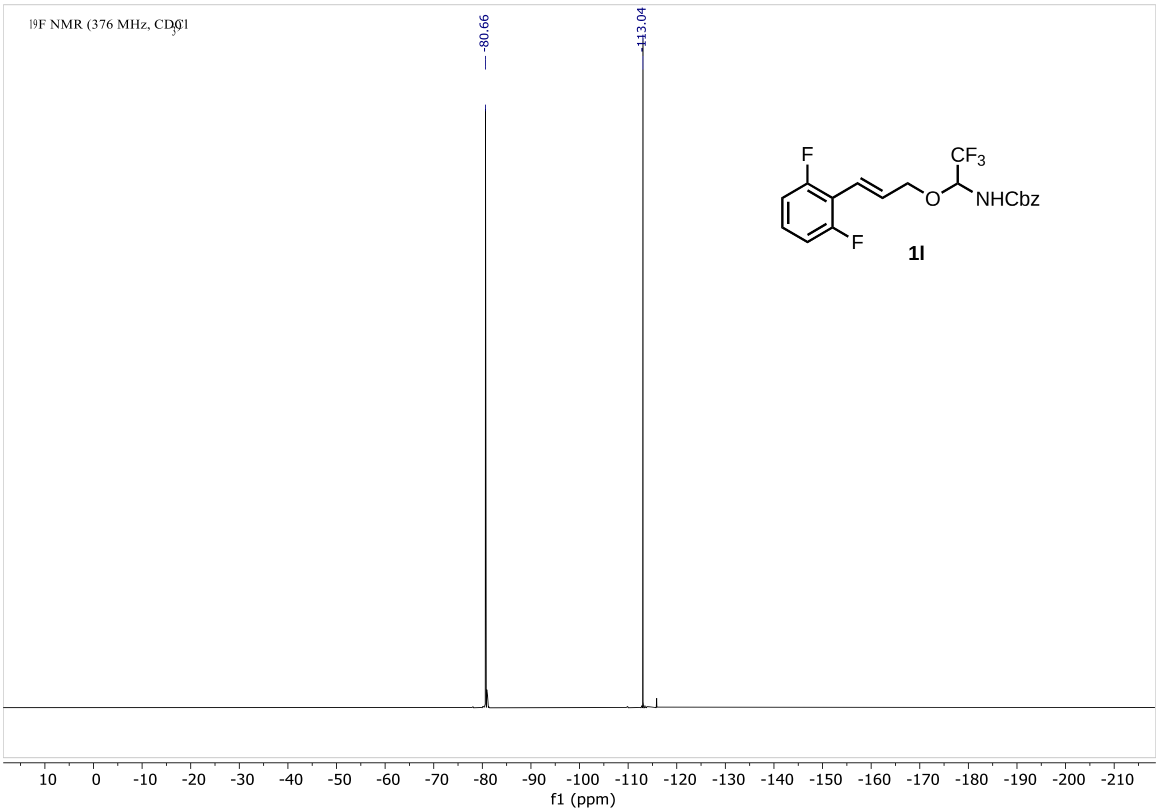

^{1}H NMR (400 MHz, $CDCl_3$)

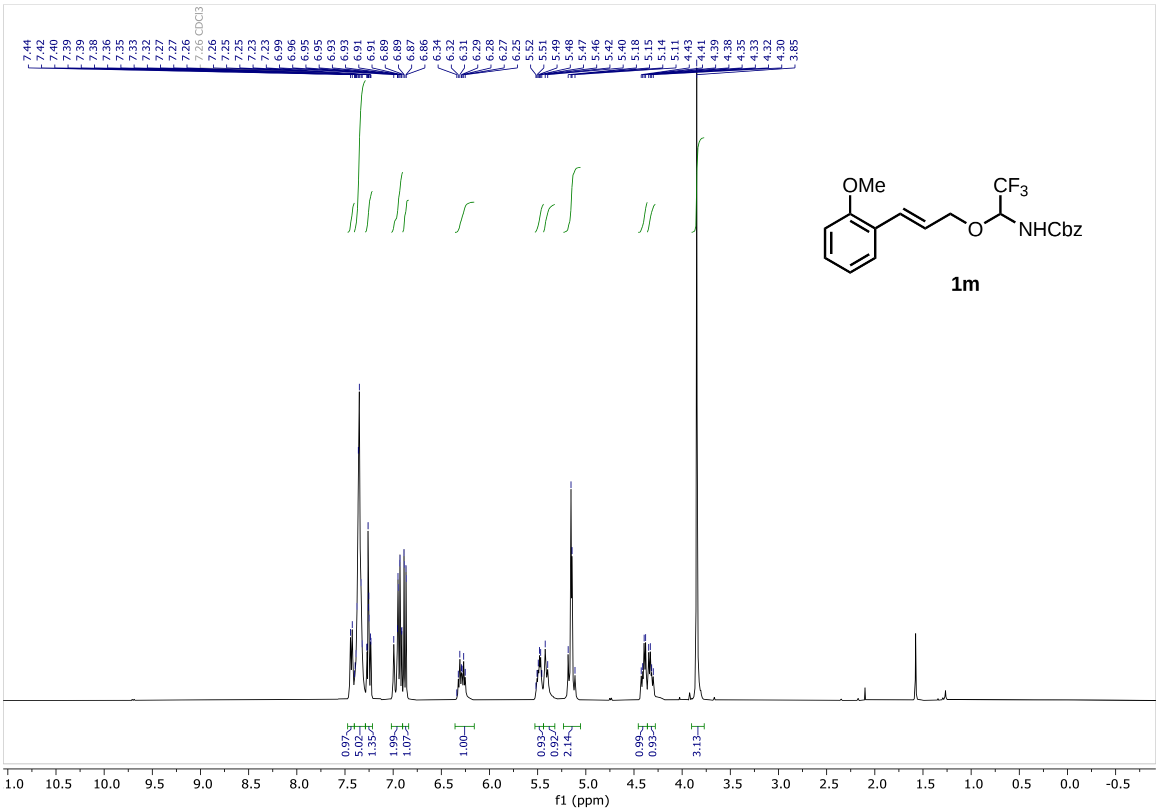

^{13}C NMR (101 MHz, $CDCl_3$)

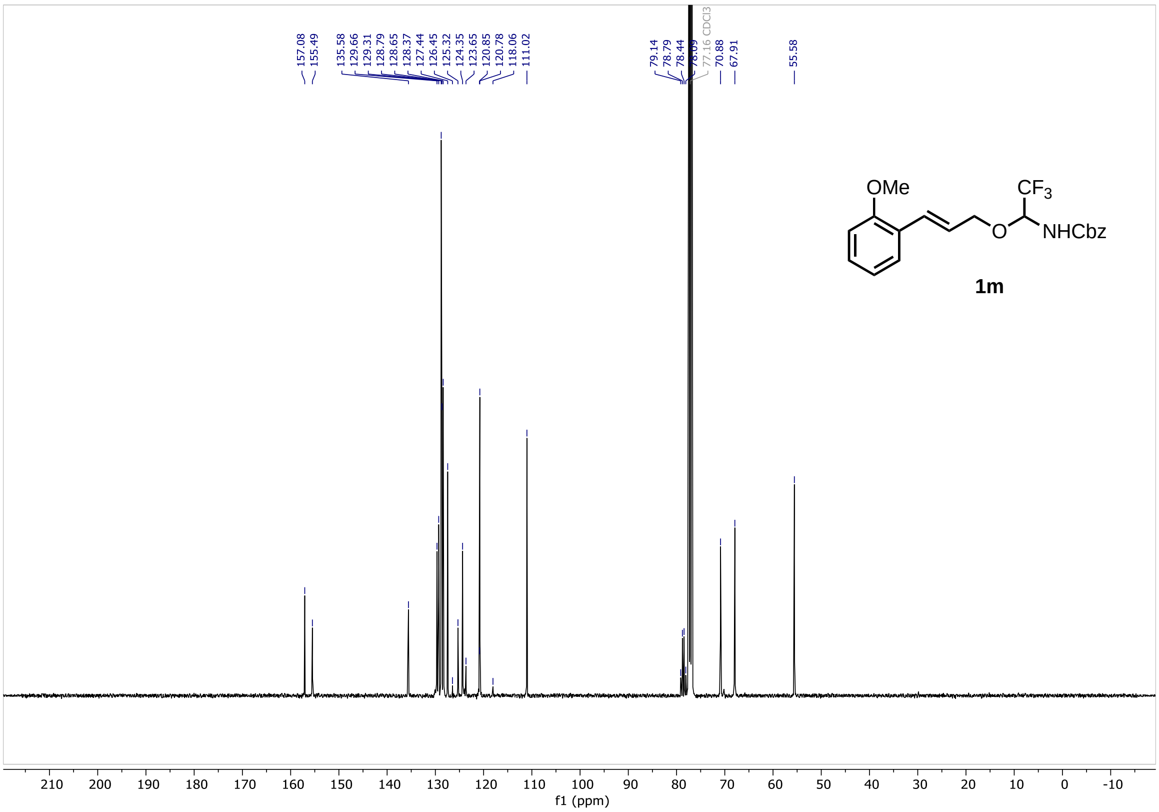

^{19}F NMR (376 MHz, $CDCl_3$)

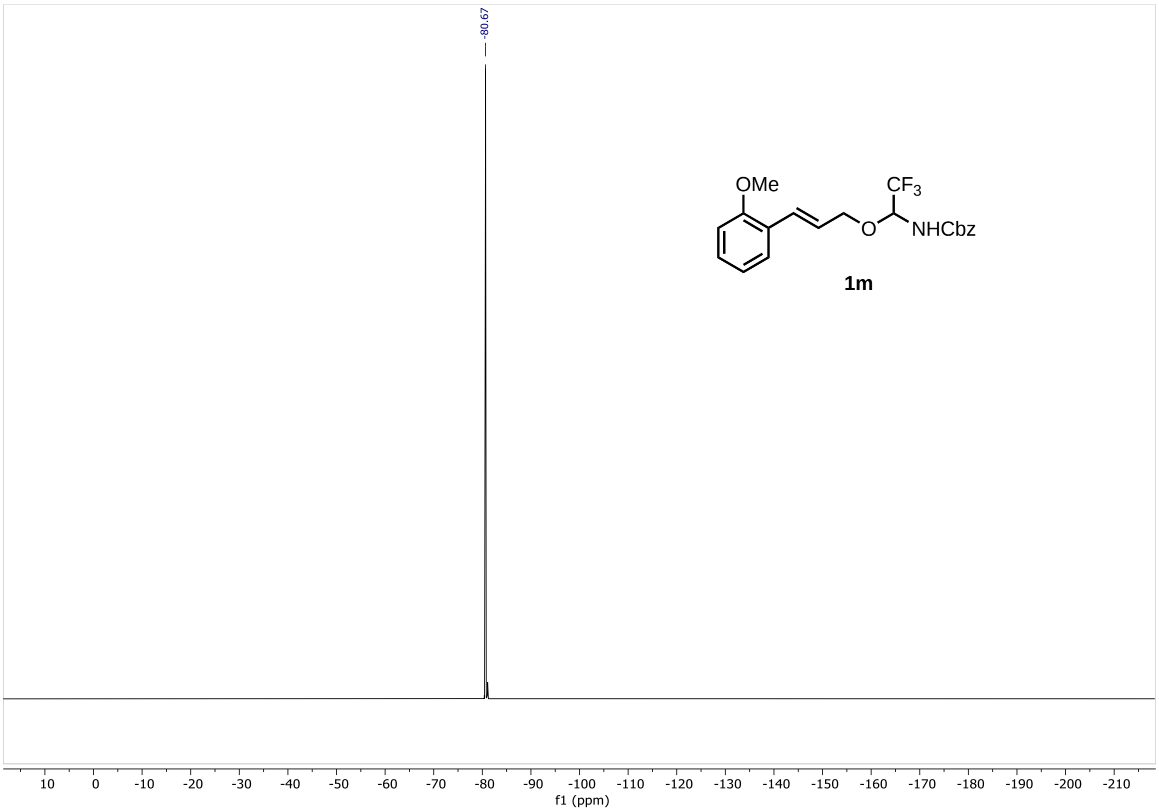

^{1}H NMR (400 MHz, $CDCl_3$)

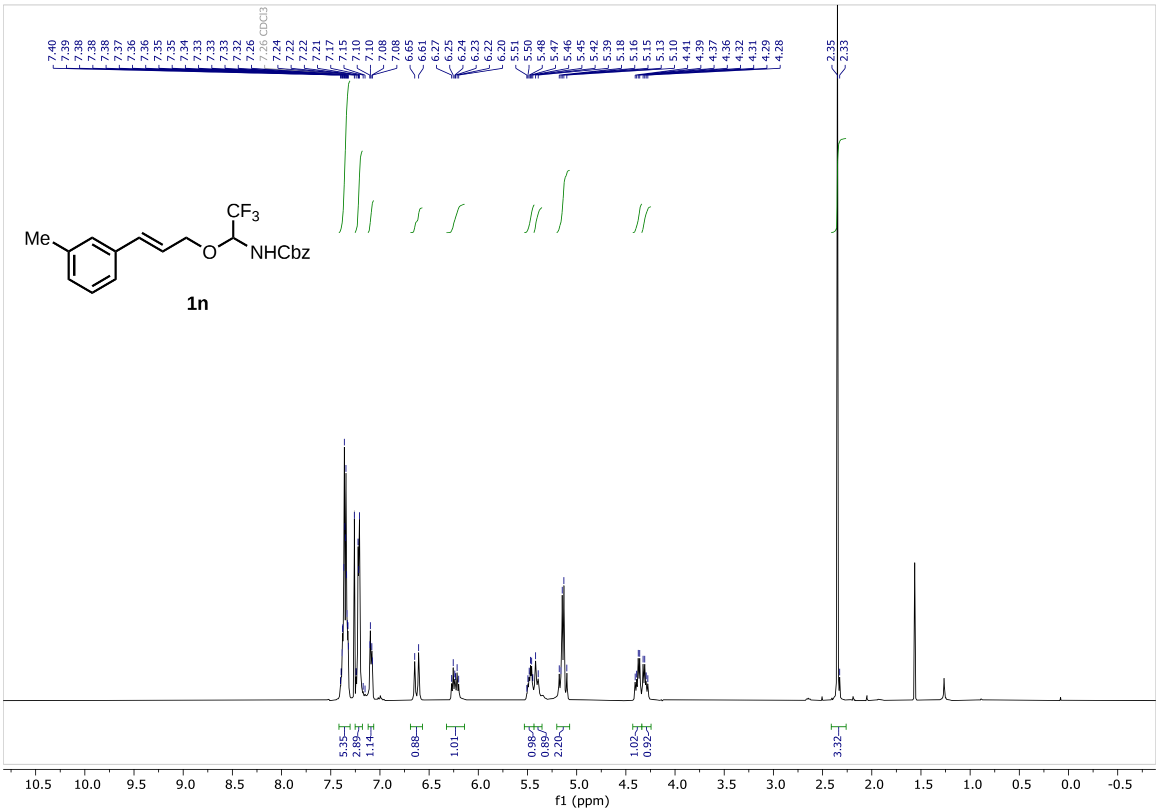

^{13}C NMR (101 MHz, $CDCl_3$)

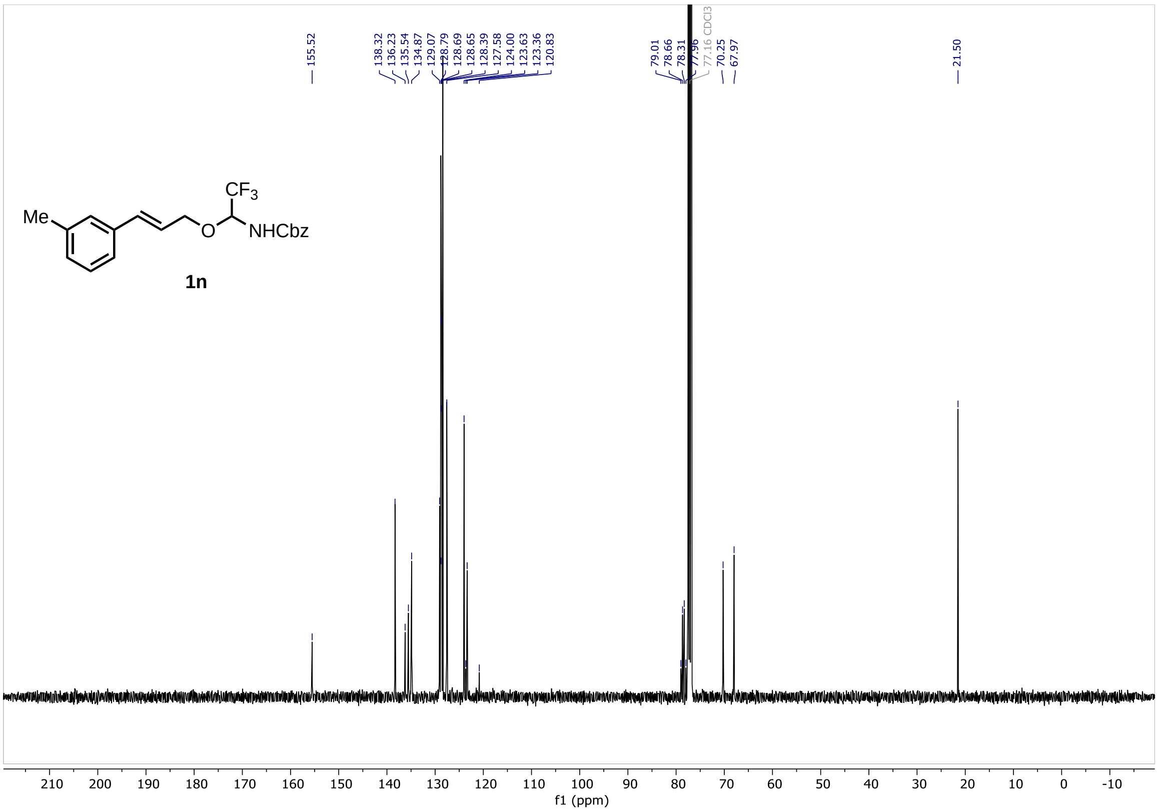

^{19}F NMR (376 MHz, $CDCl_3$)

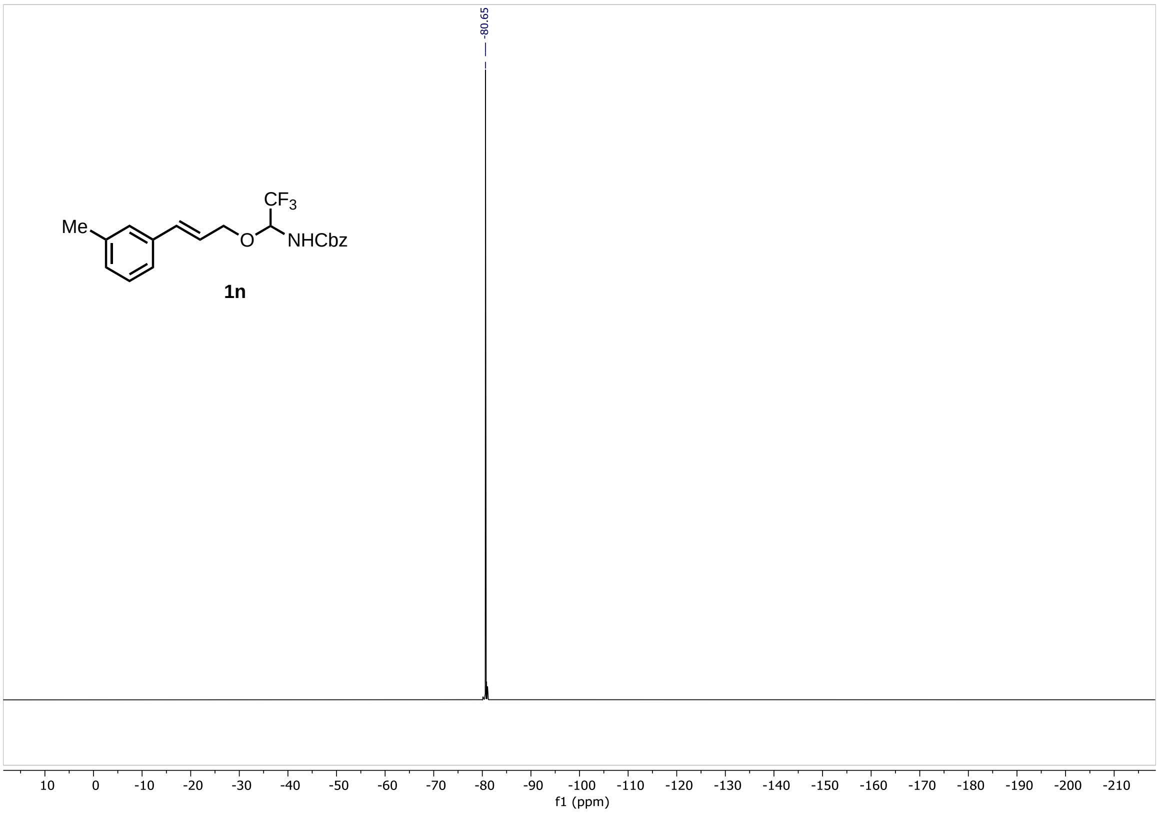

^{1}H NMR (400 MHz, $CDCl_3$)

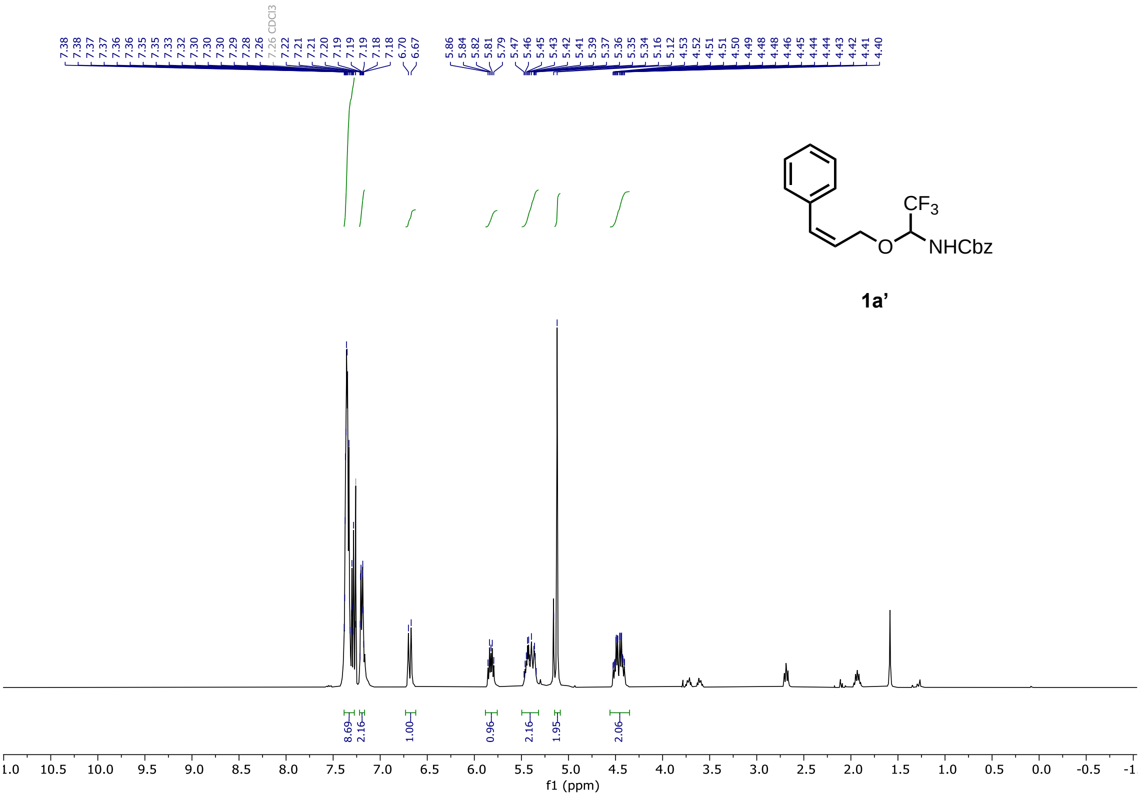

*Remnants of corresponding fully saturated product visible by NMR, yield has been adjusted accordingly.

^{13}C NMR (101 MHz, $CDCl_3$)

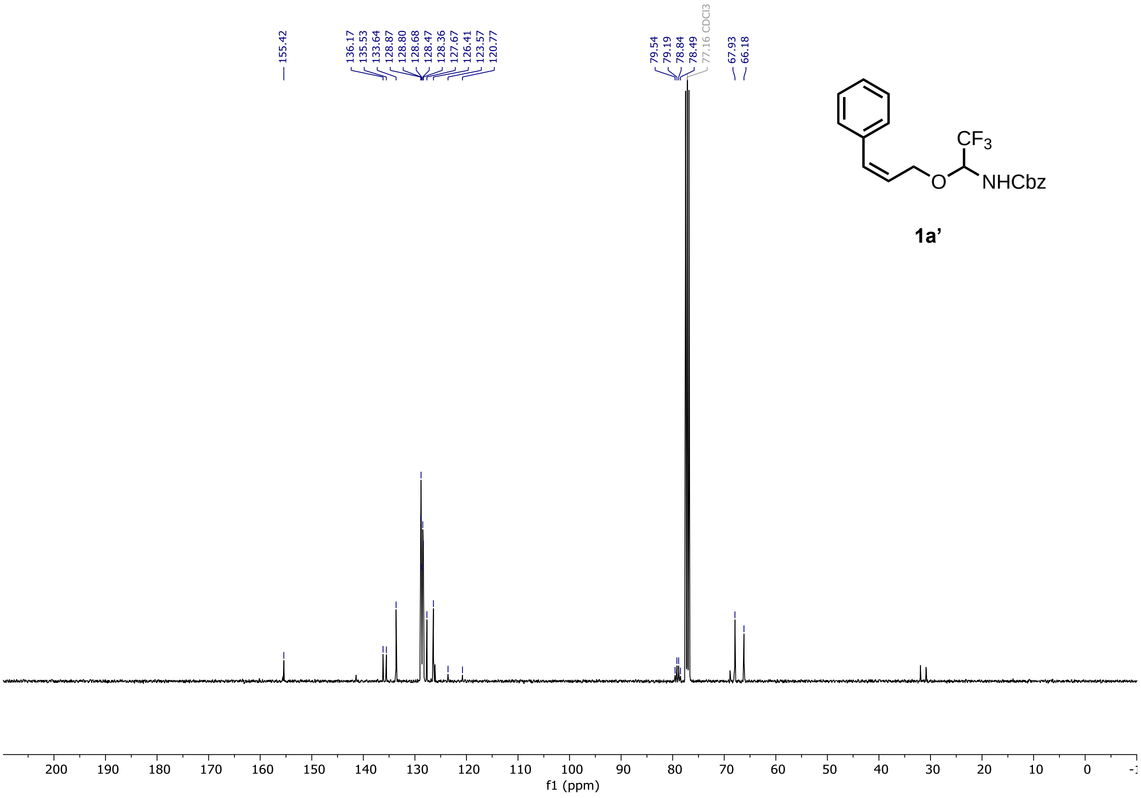

^{19}F NMR (376 MHz, $CDCl_3$)

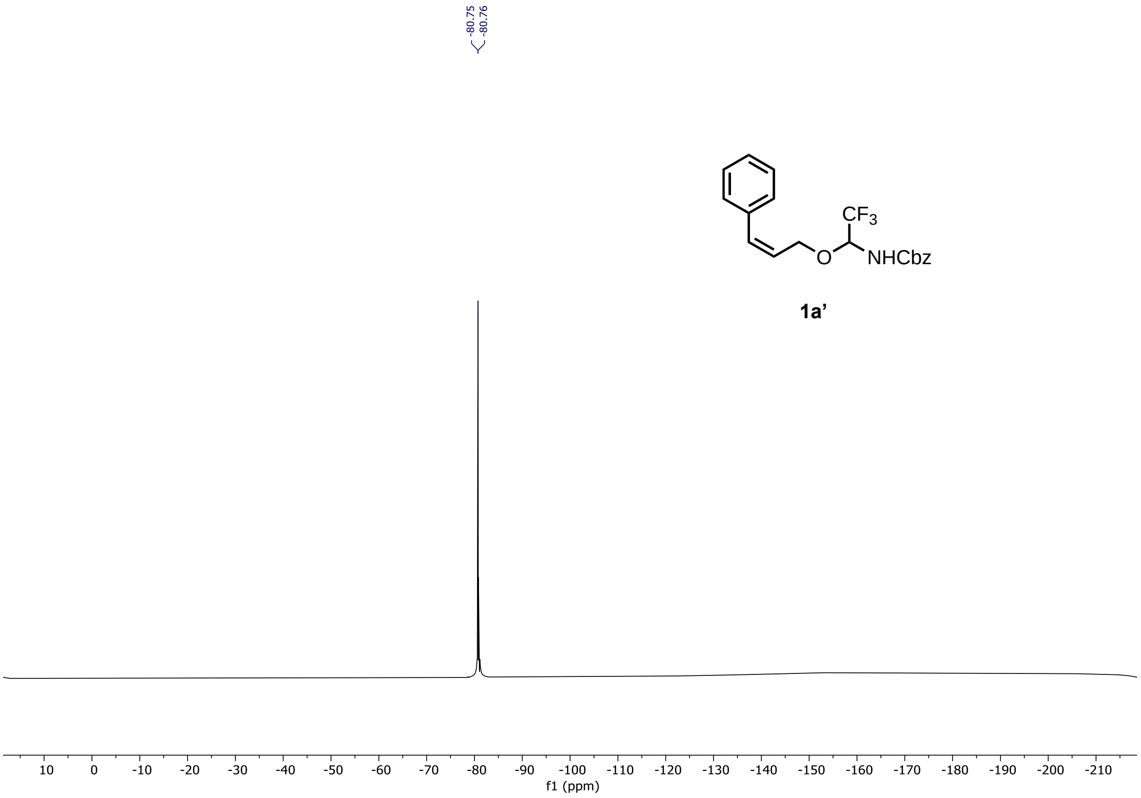

^{1}H NMR (400 MHz, $CDCl_3$)

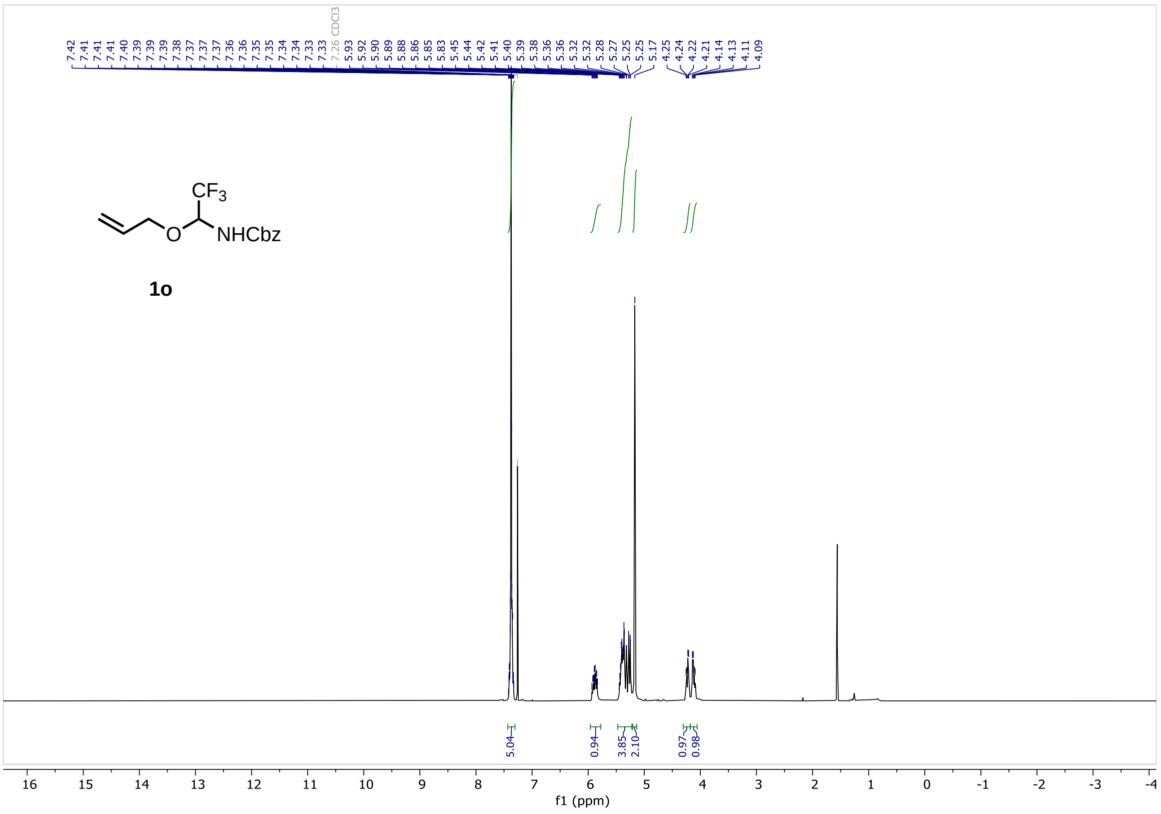

^{13}C NMR (101 MHz, $CDCl_3$)

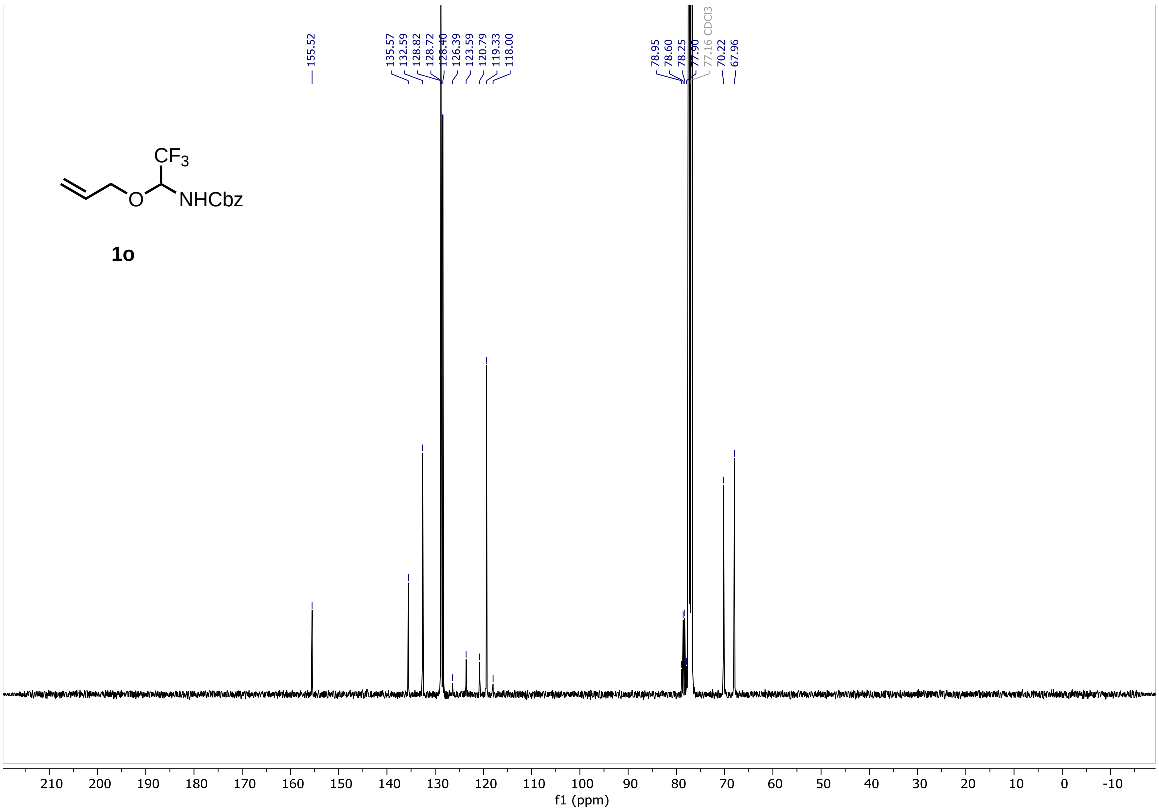

^{19}F NMR (376 MHz, $CDCl_3$)

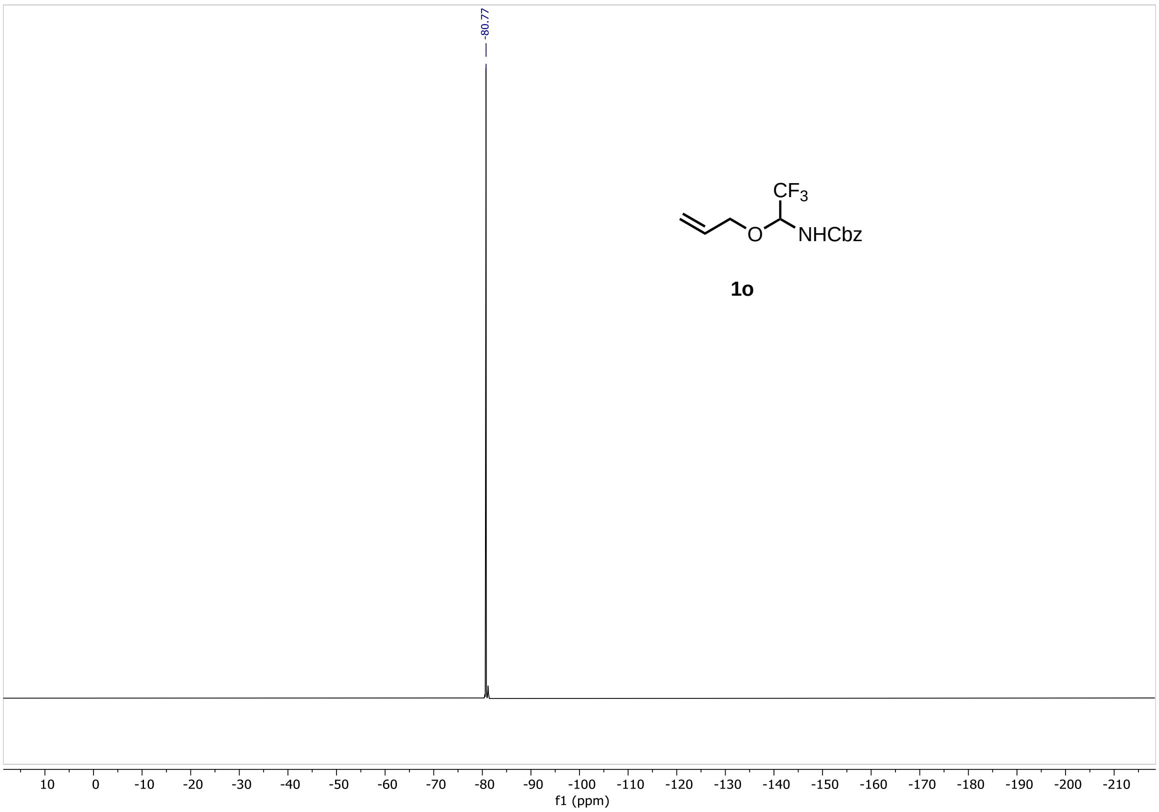

^{1}H NMR (400 MHz, $CDCl_3$)

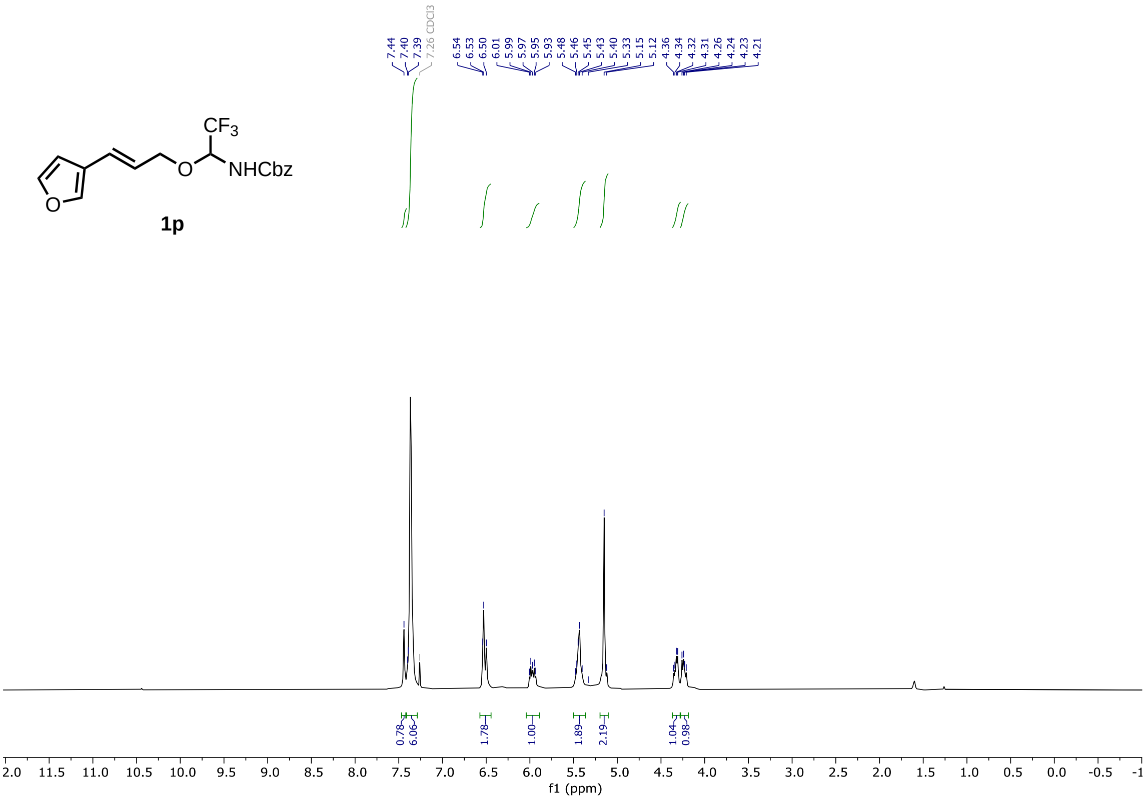

^{13}C NMR (101 MHz, $CDCl_3$)

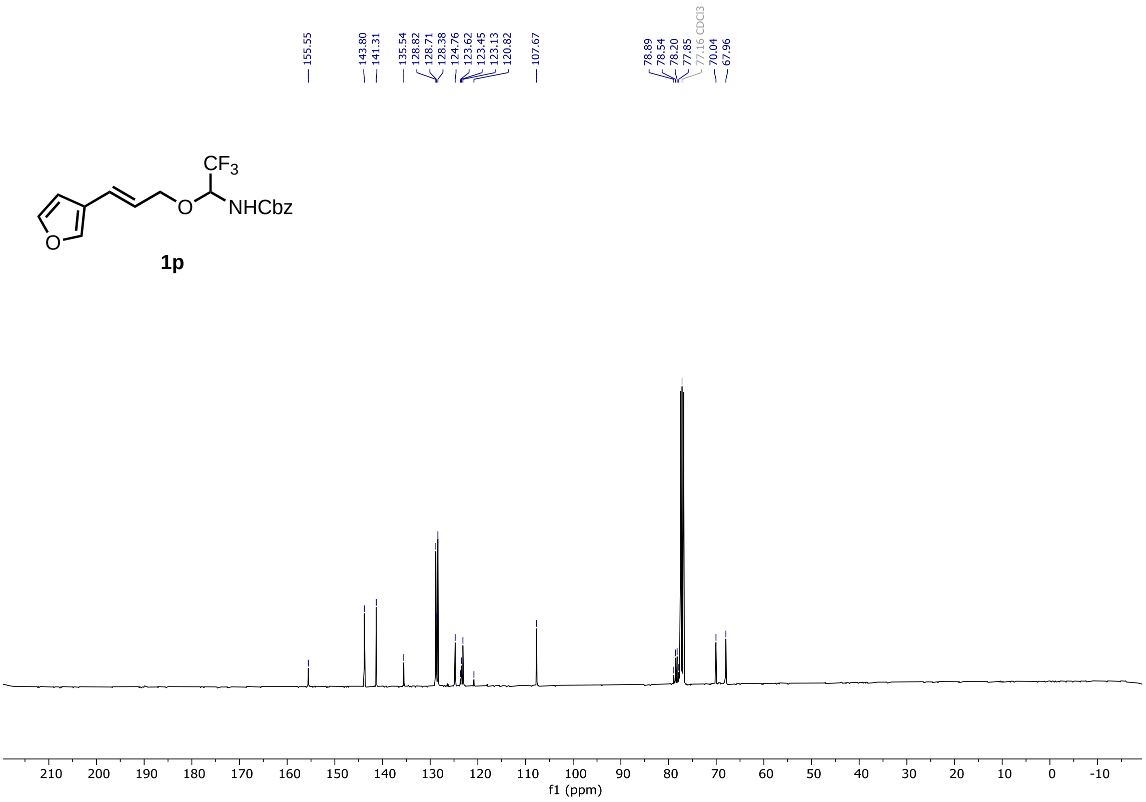

^{19}F NMR (376 MHz, $CDCl_3$)

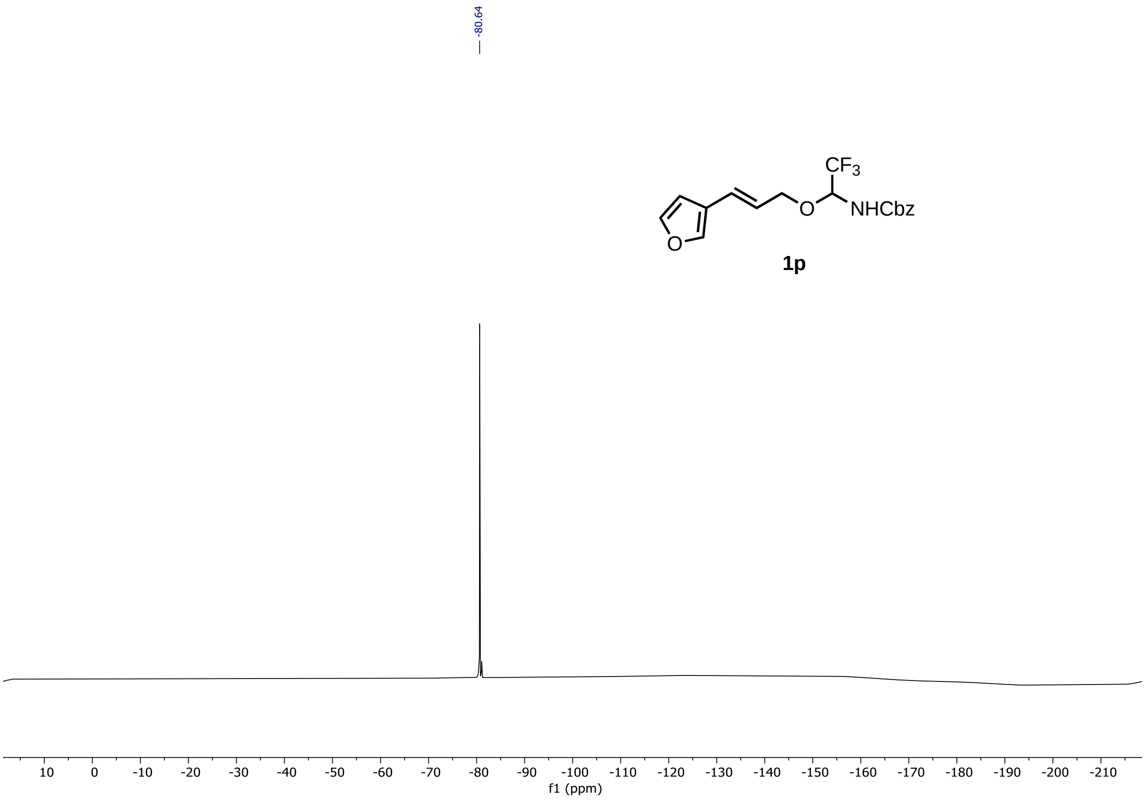

^{1}H NMR (400 MHz, $CDCl_3$)

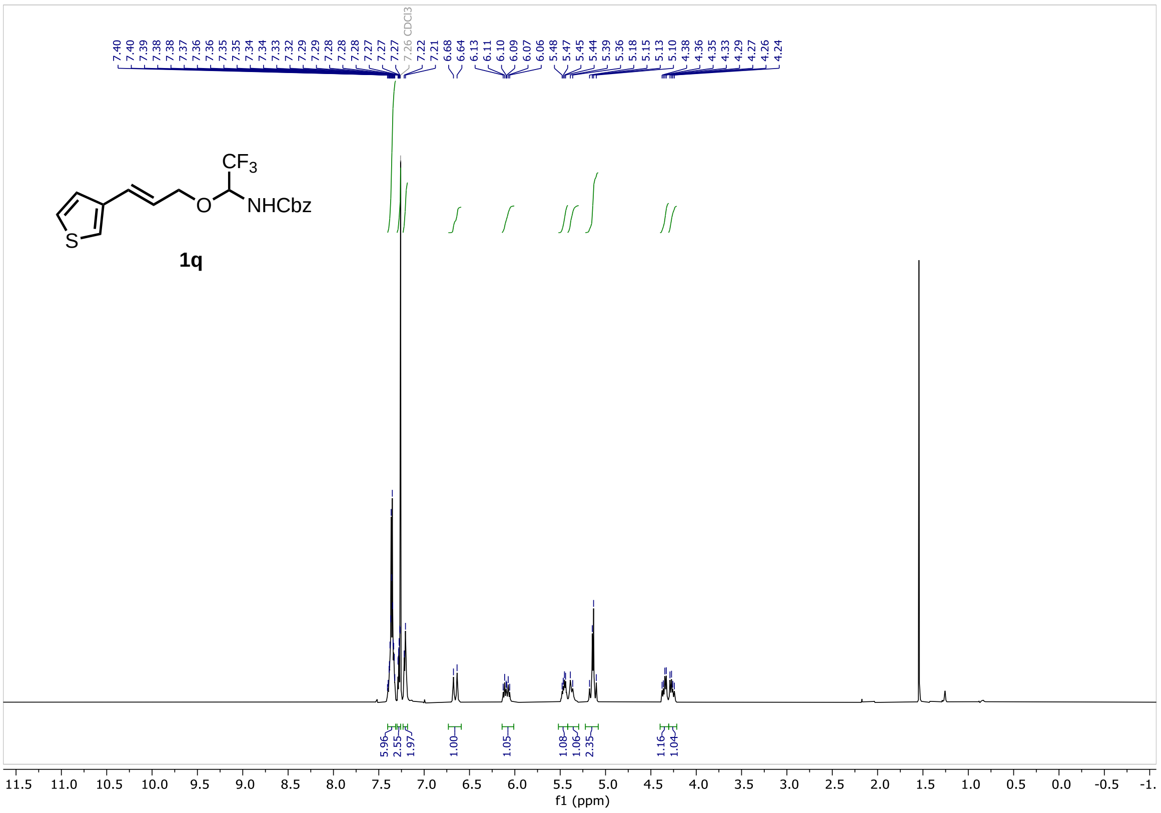

^{13}C NMR (101 MHz, $CDCl_3$)

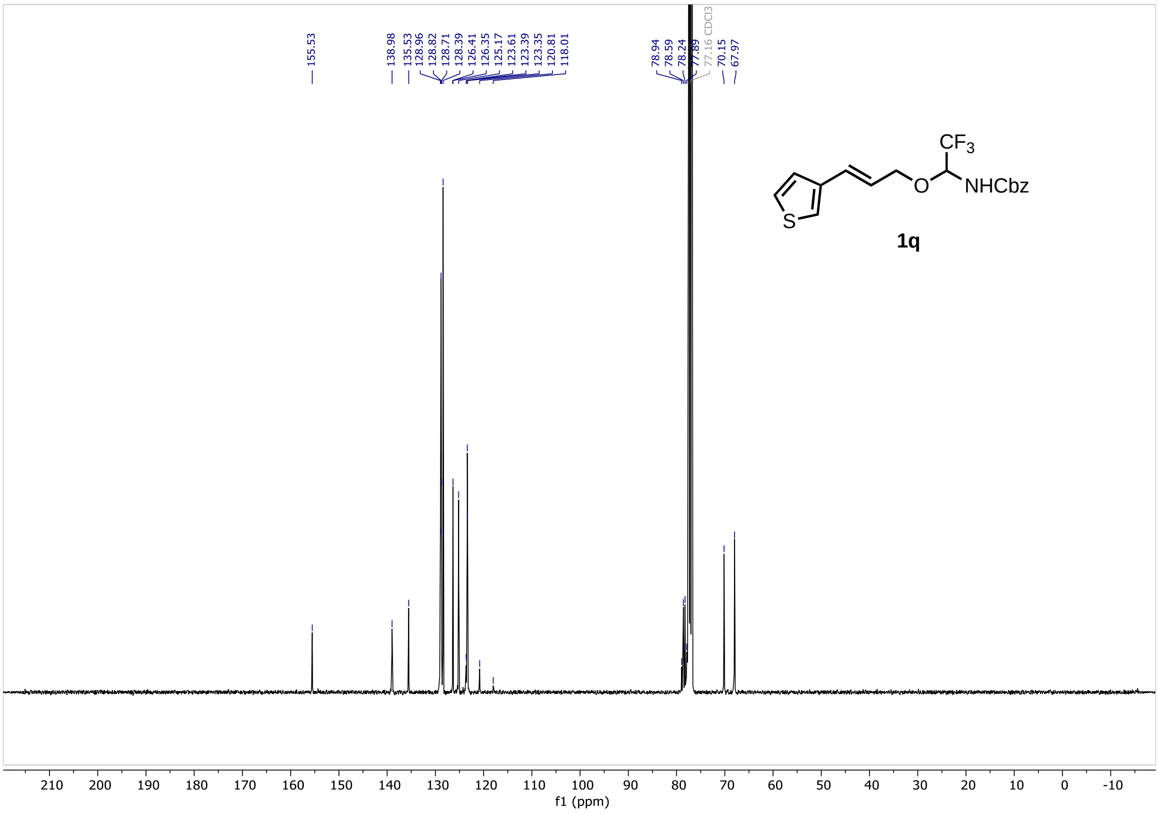

^{19}F NMR (376 MHz, $CDCl_3$)

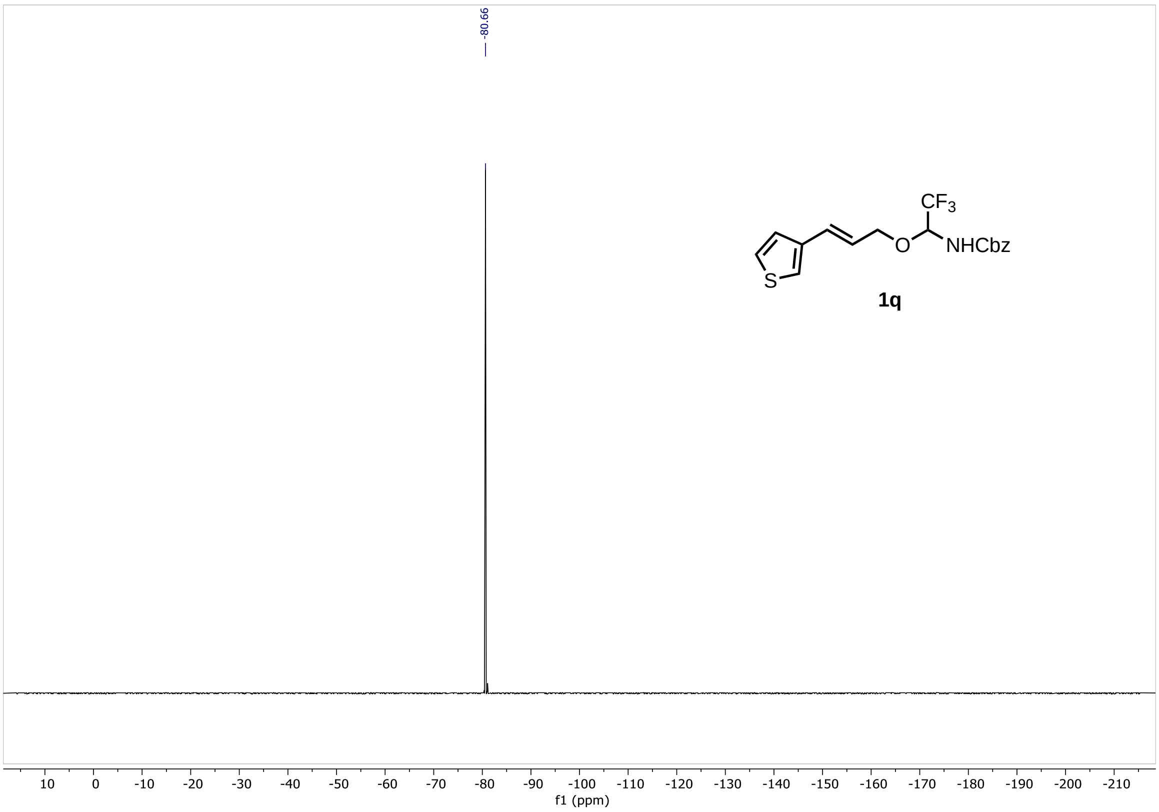

^{1}H NMR (400 MHz, $CDCl_3$)

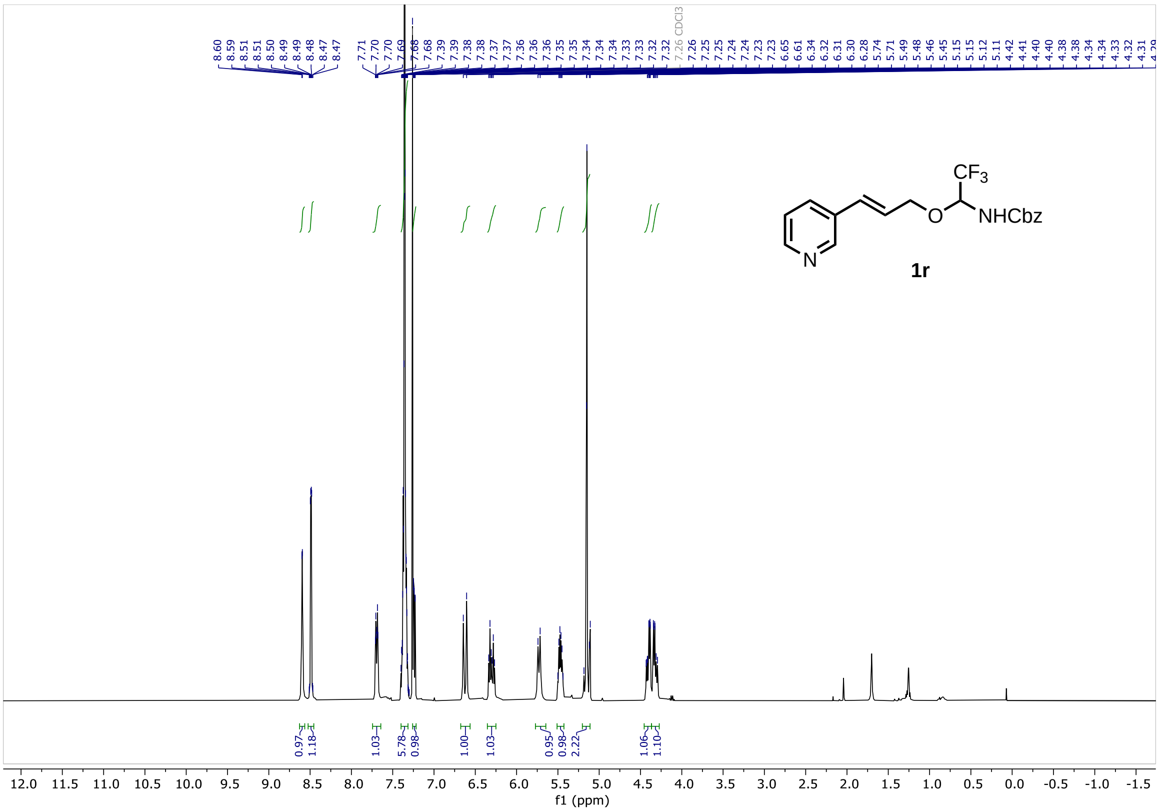

^{13}C NMR (101 MHz, $CDCl_3$)

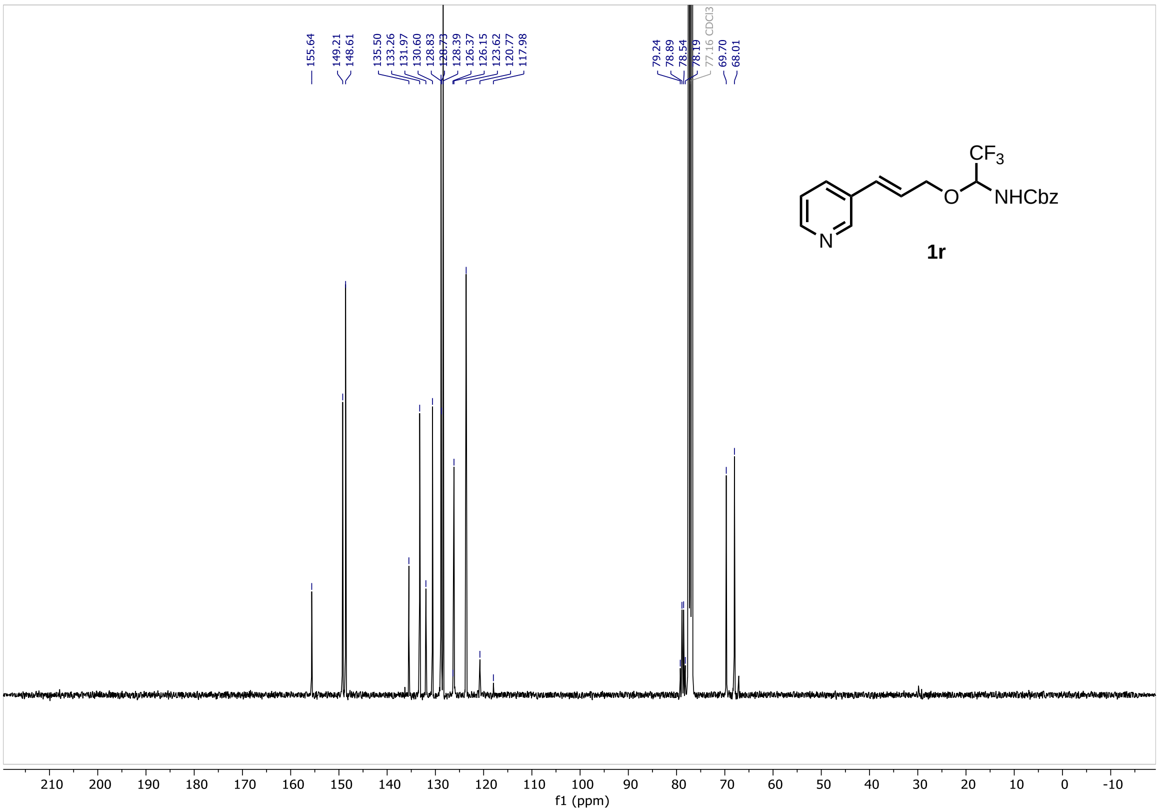

^{19}F NMR (376 MHz, $CDCl_3$)

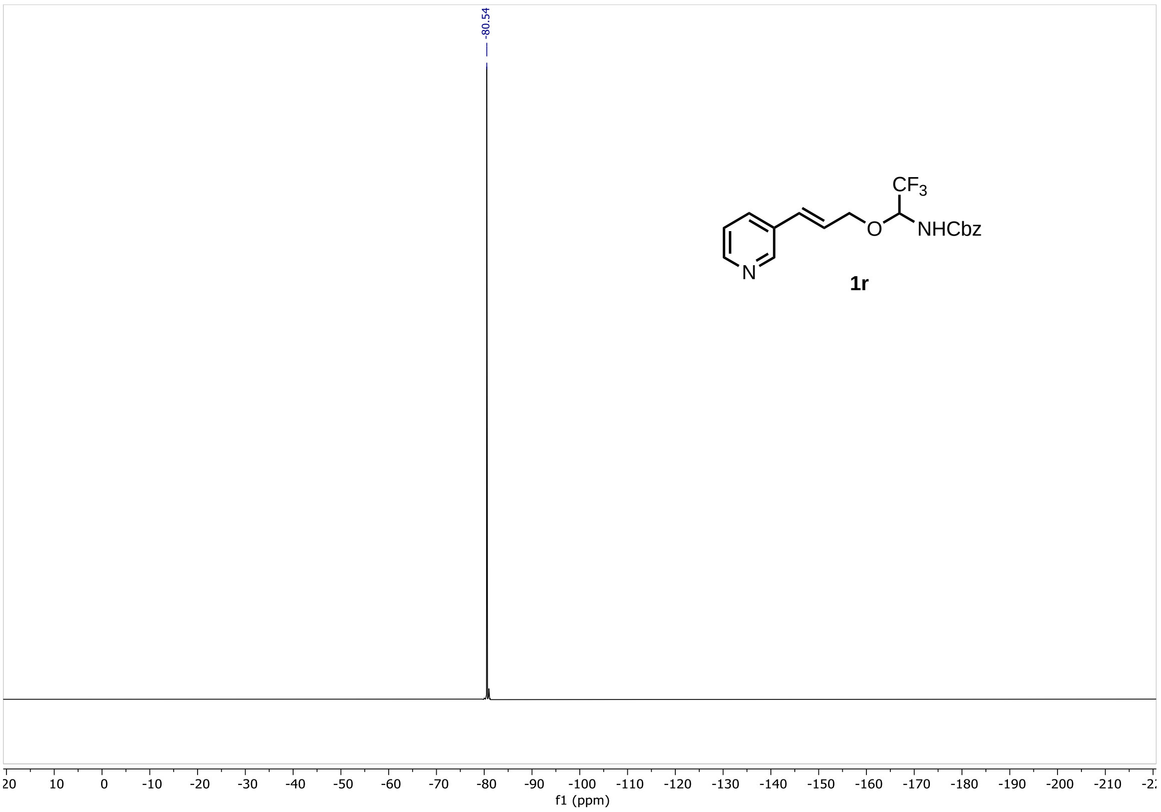

^{1}H NMR (400 MHz, $CDCl_3$)

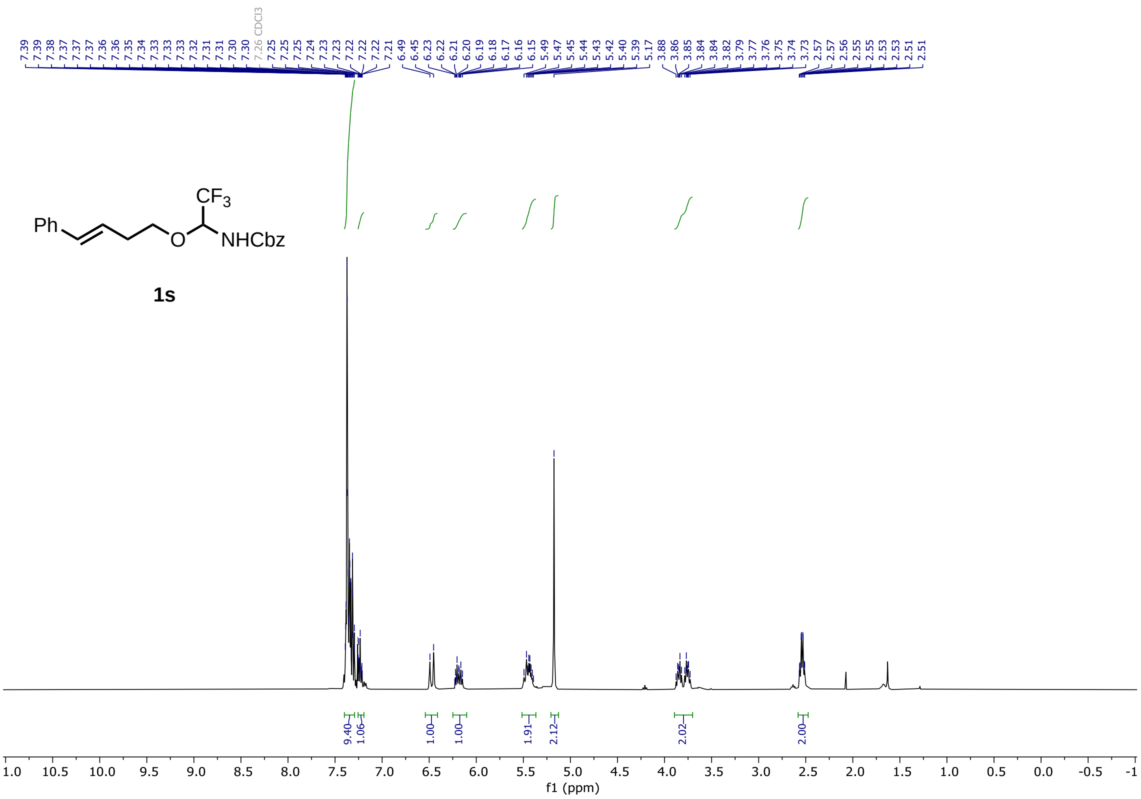

^{13}C NMR (101 MHz, $CDCl_3$)

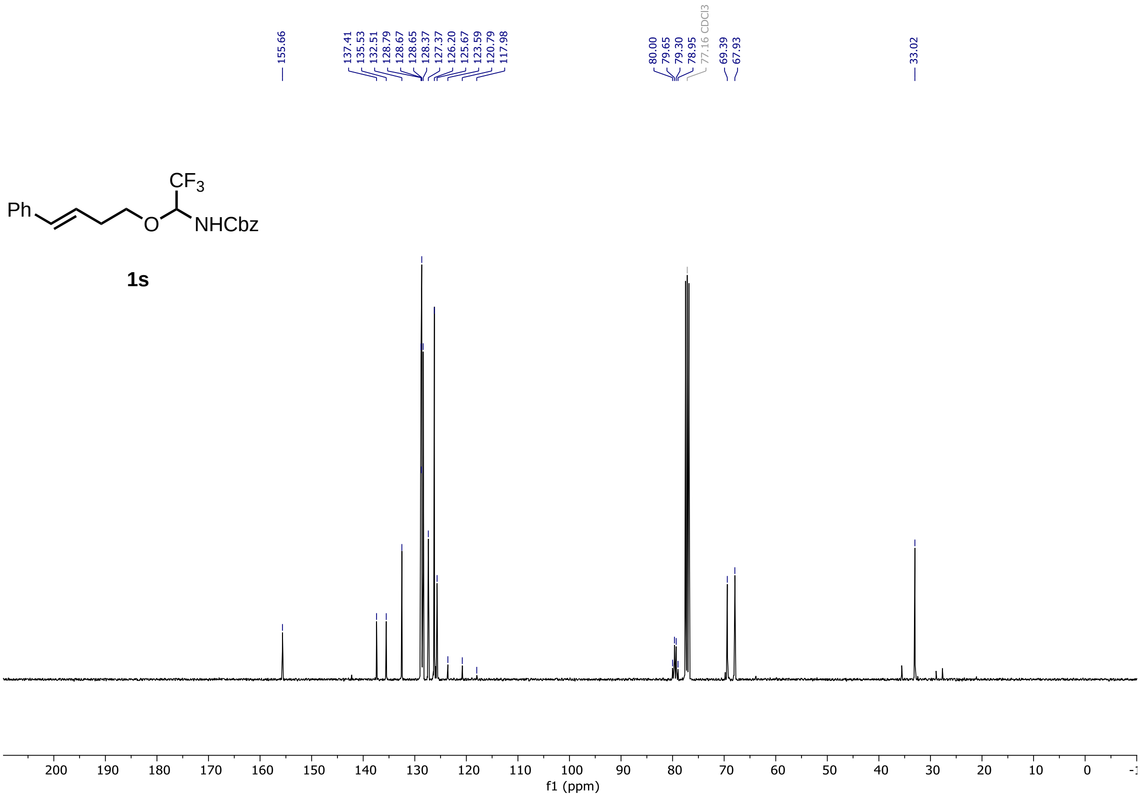

^{19}F NMR (376 MHz, $CDCl_3$)

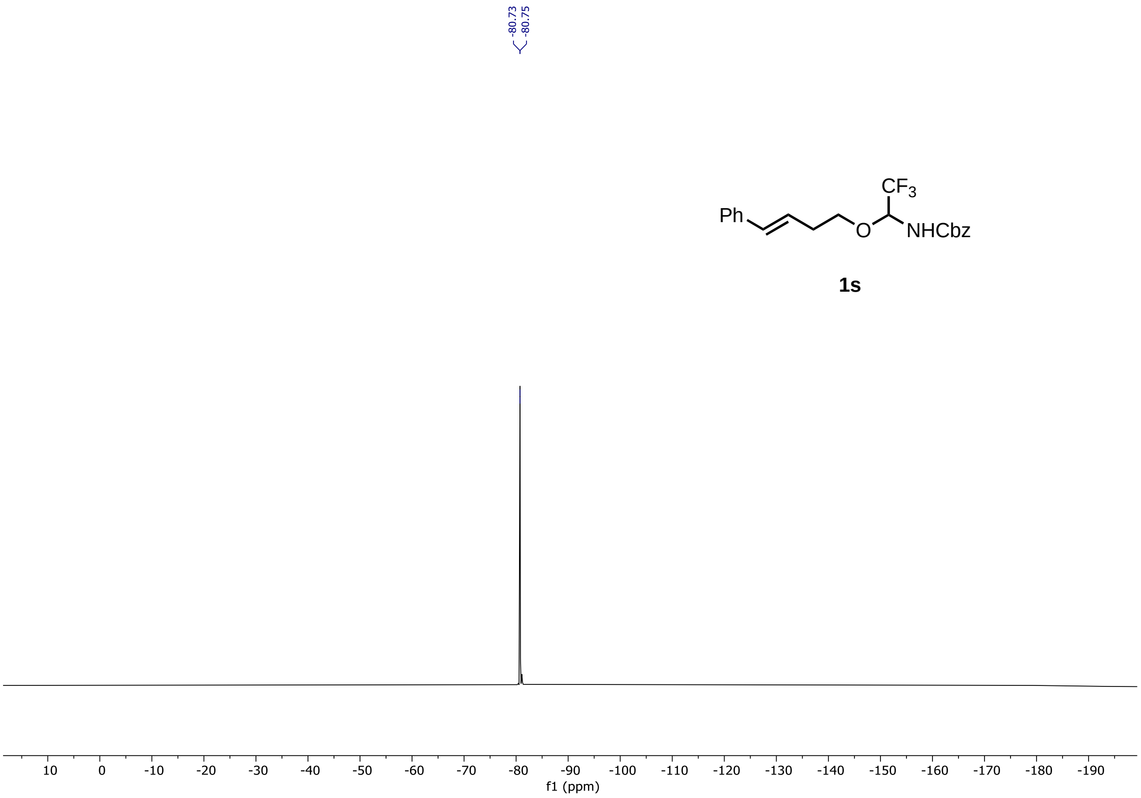

^{1}H NMR (400 MHz, $CDCl_3$)

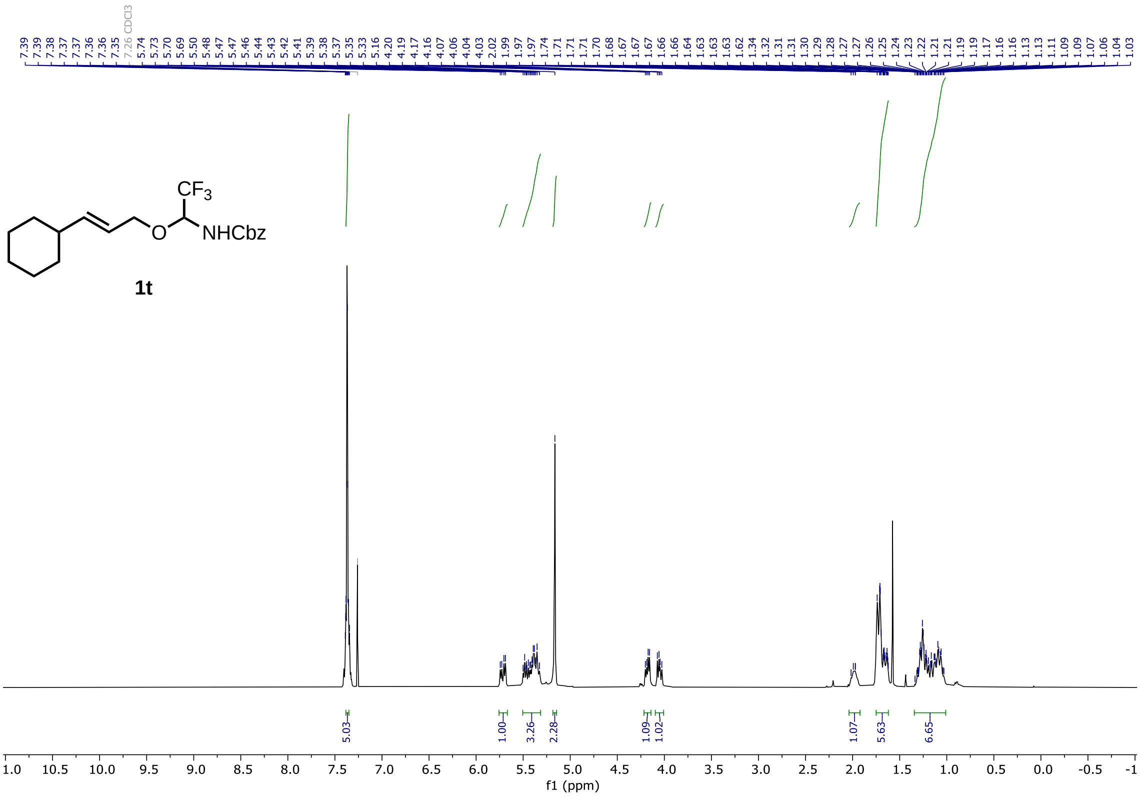

^{13}C NMR (101 MHz, $CDCl_3$)

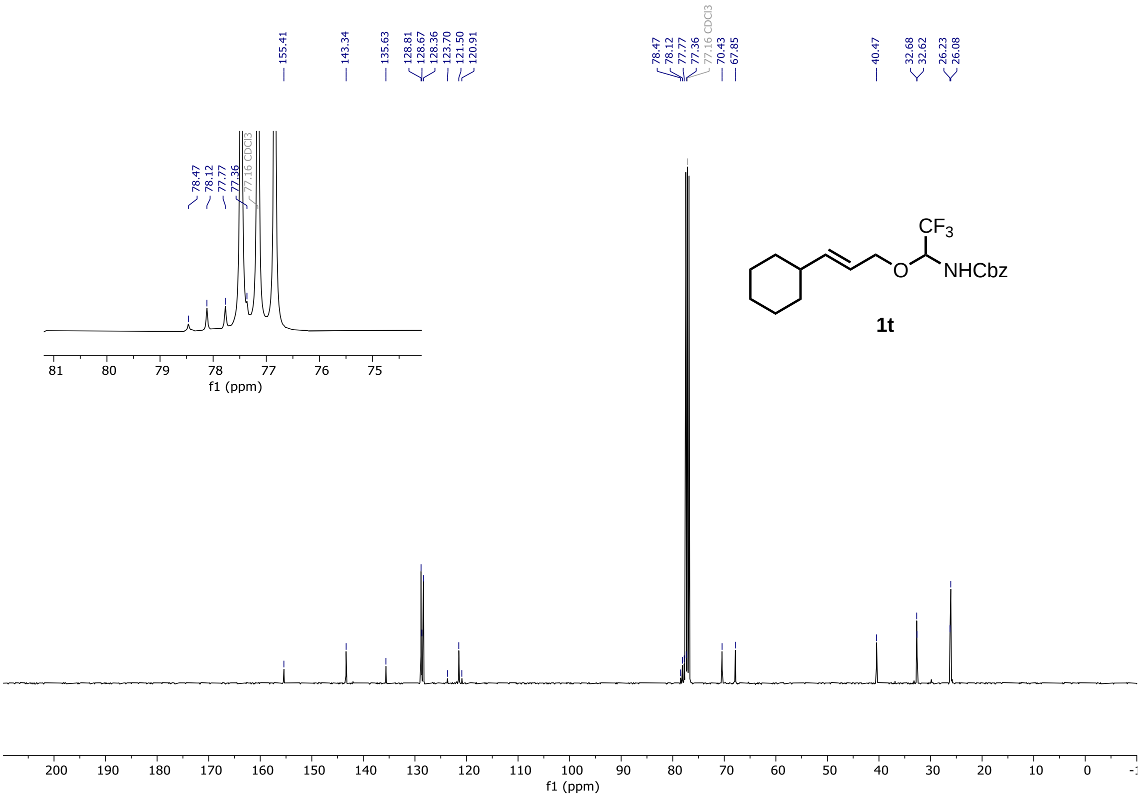

^{19}F NMR (376 MHz, $CDCl_3$)

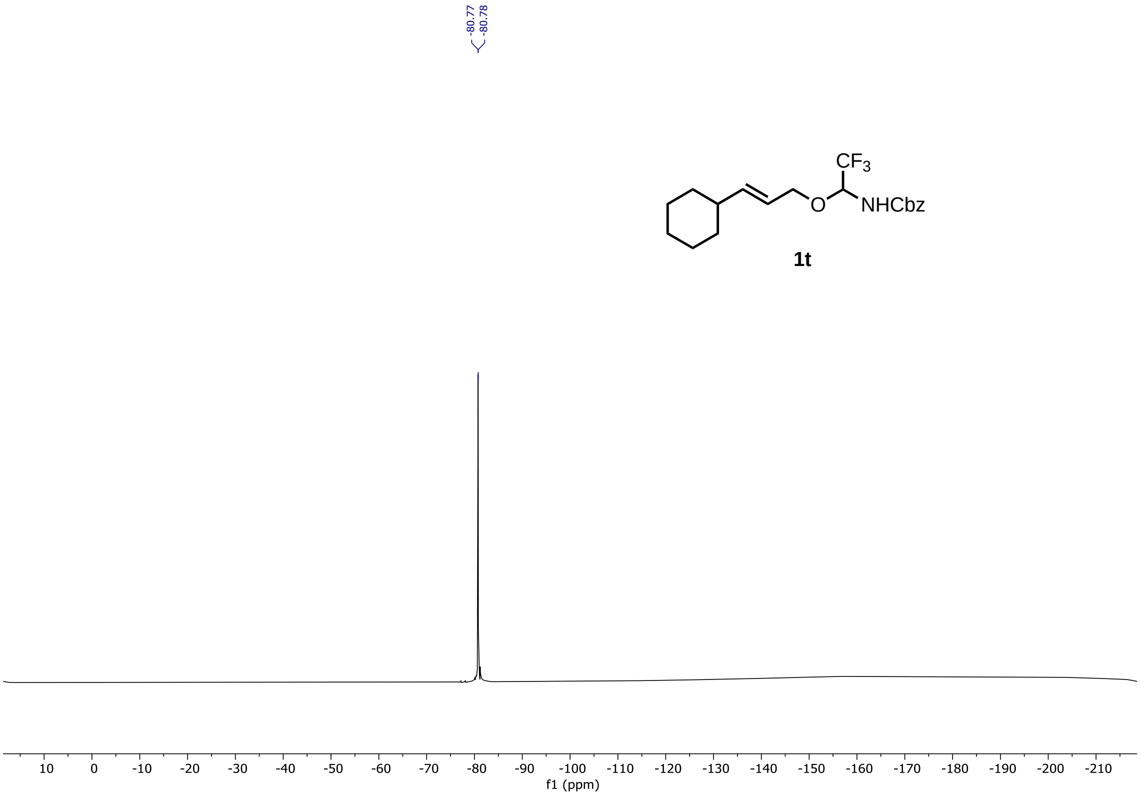

1t

^{1}H NMR (400 MHz, $CDCl_3$)

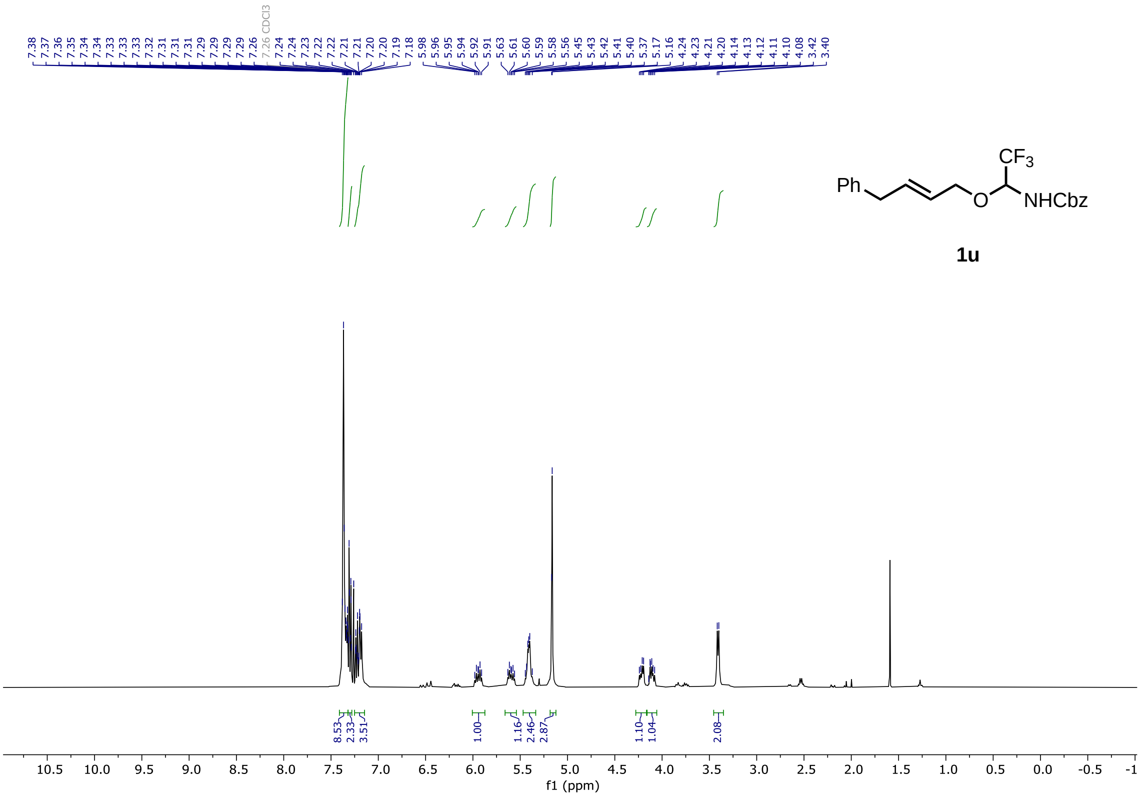

1u

*remnants of corresponding tether product with alkene in benzylic position visible by NMR, yield has been adjusted accordingly. This by-product was generated in the previous step and could not be removed. Under these conditions, 6-exo cyclisation of this by-product is not viable.

^{13}C NMR (101 MHz, $CDCl_3$)

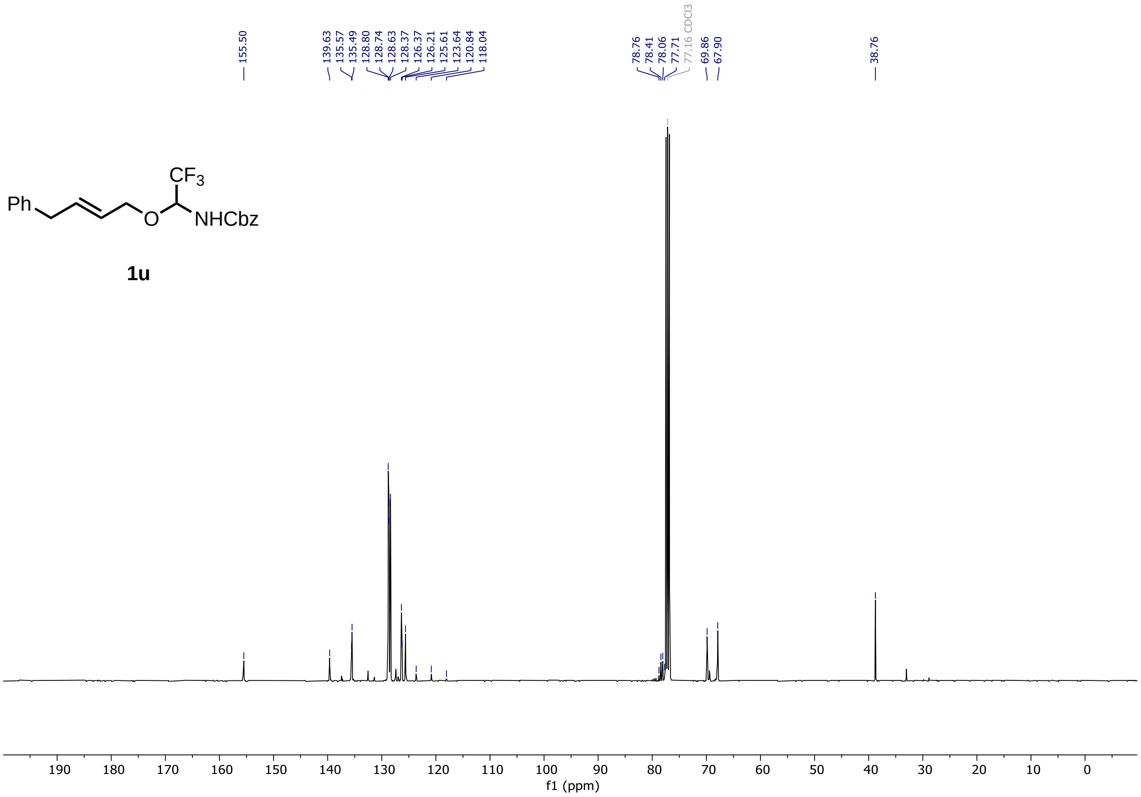

^{19}F NMR (376 MHz, $CDCl_3$)

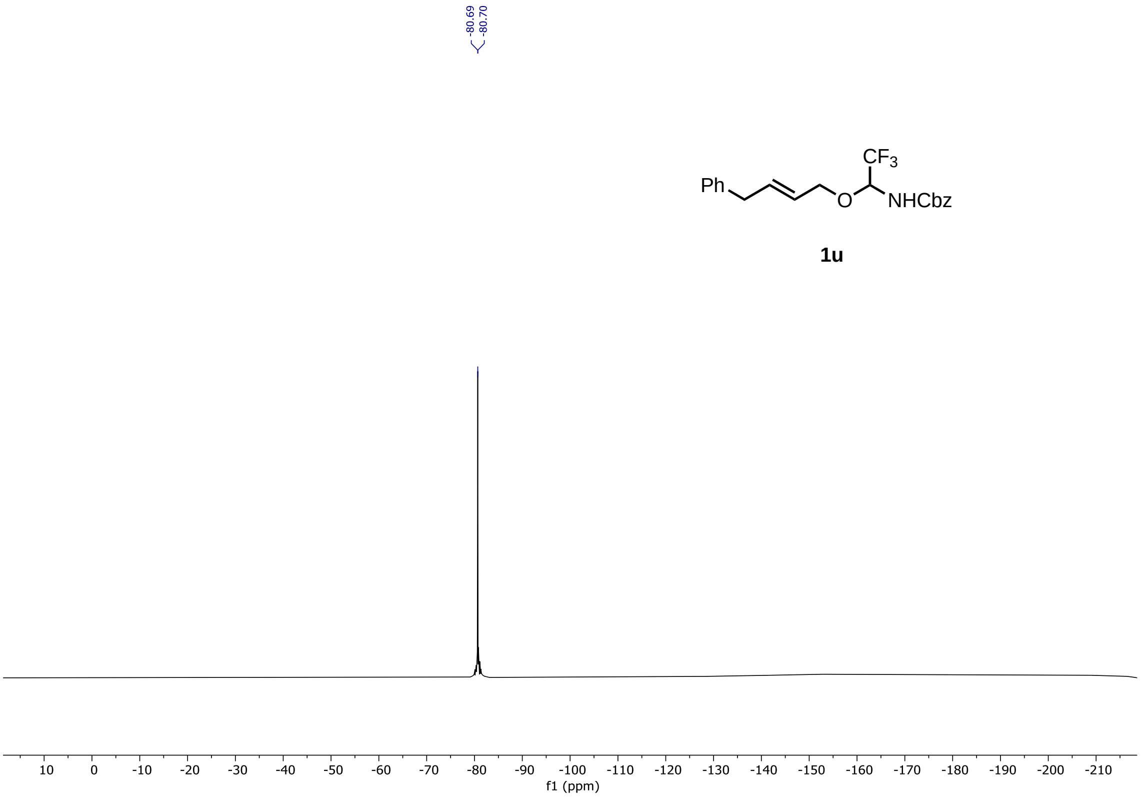

^{1}H NMR (400 MHz, $CDCl_3$)

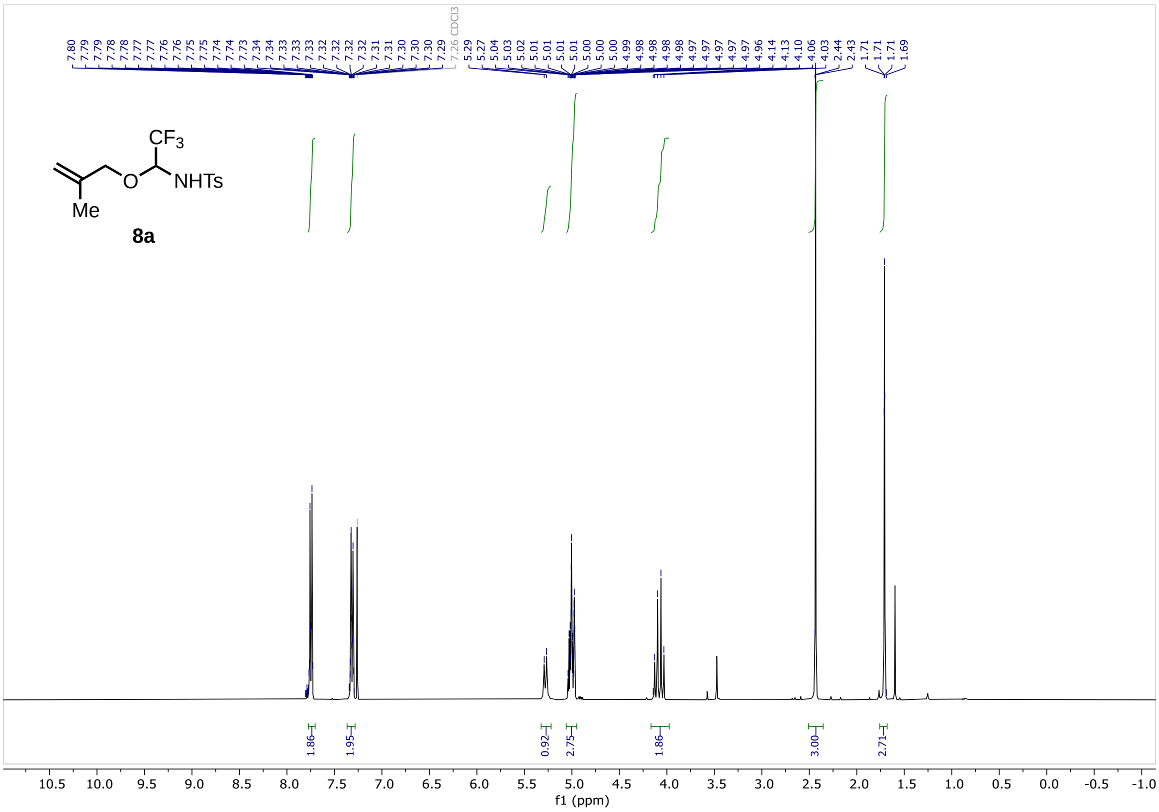

^{13}C NMR (101 MHz, $CDCl_3$)

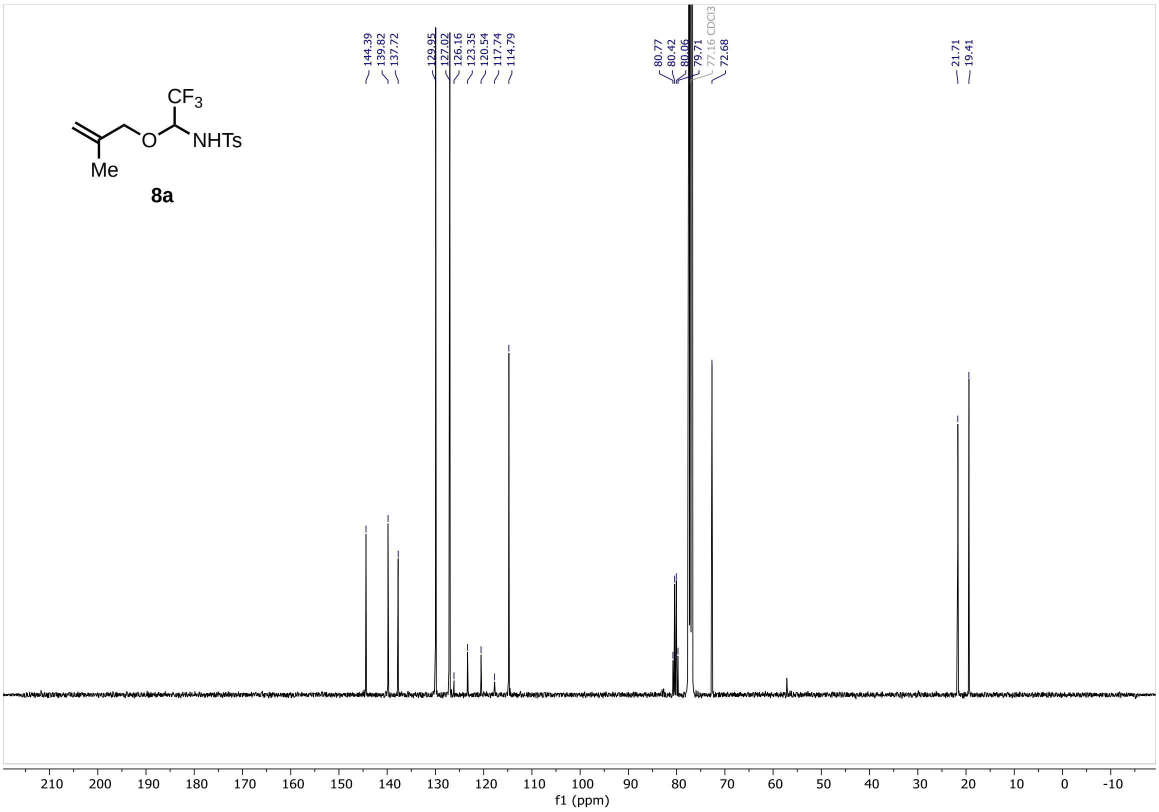

^{19}F NMR (376 MHz, $CDCl_3$)

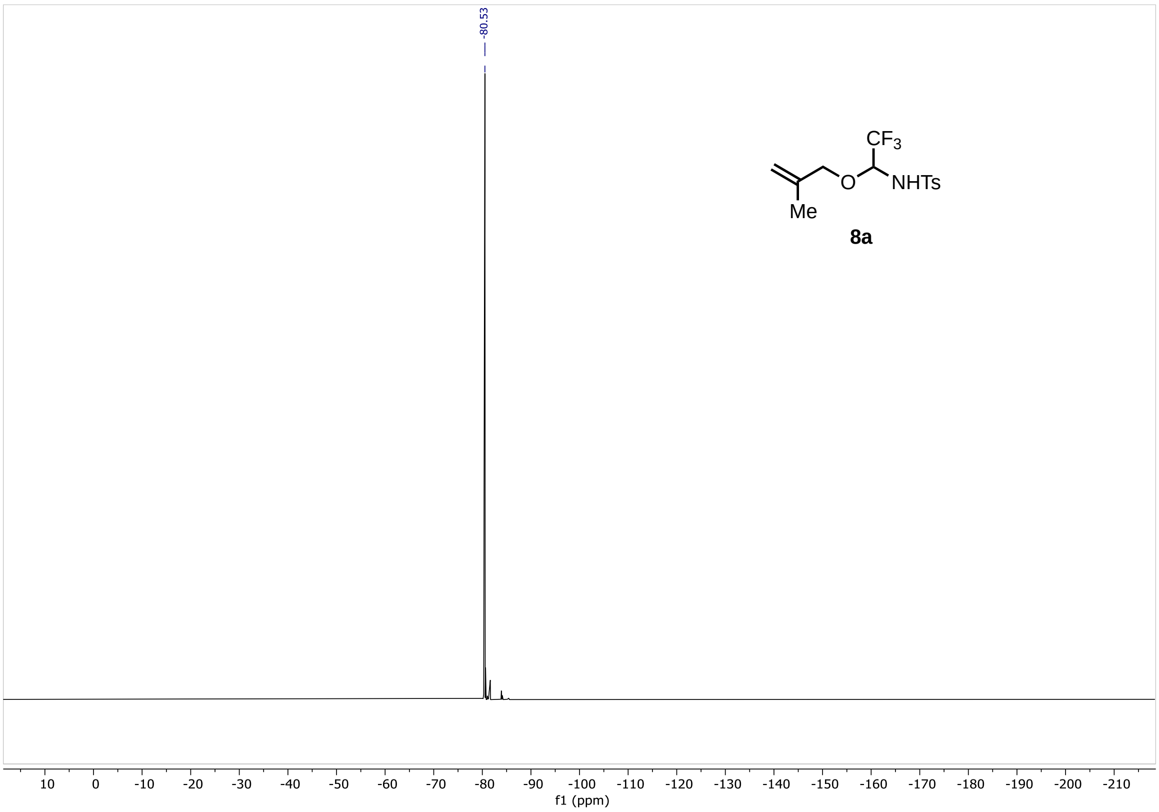

^{1}H NMR (400 MHz, $CDCl_3$)

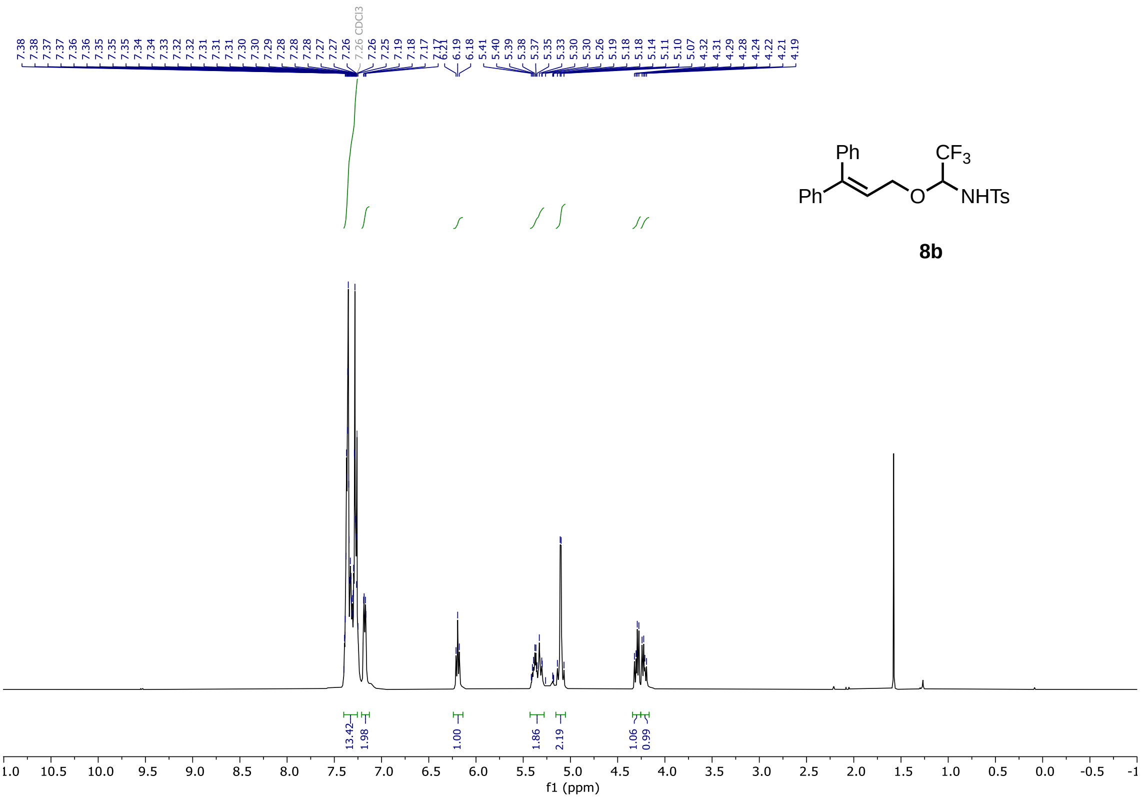

^{13}C NMR (101 MHz, $CDCl_3$)

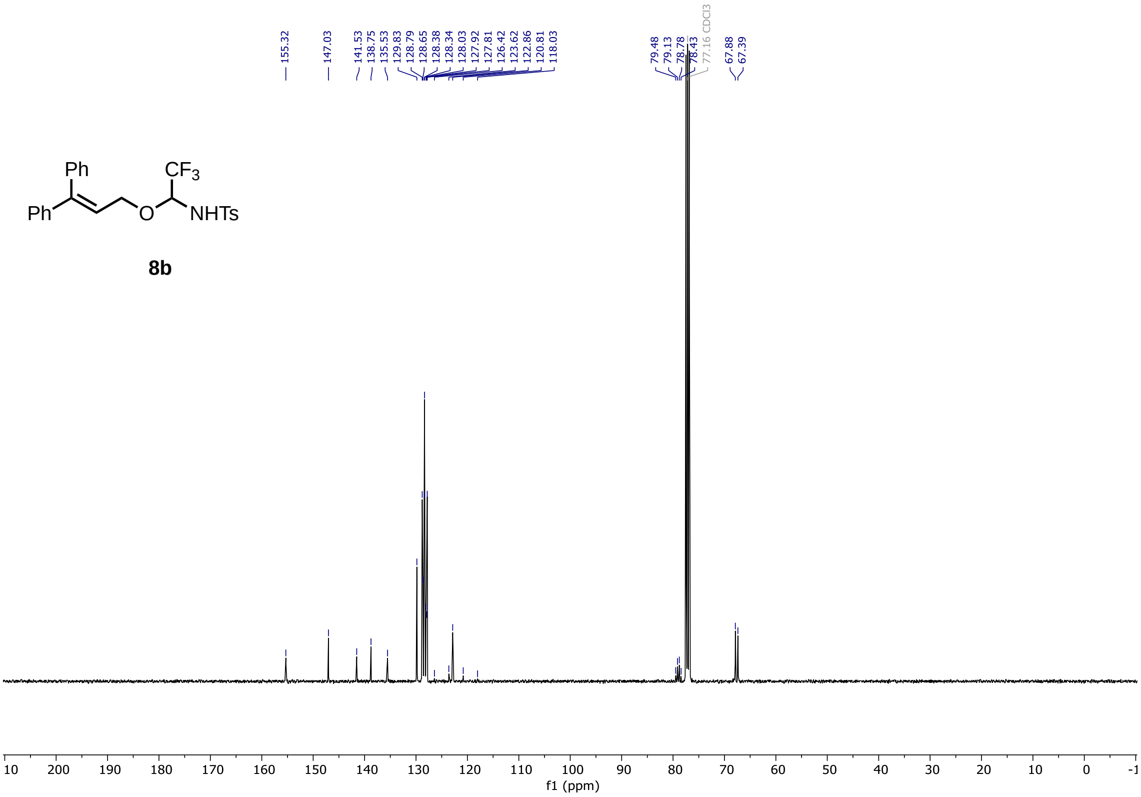

^{19}F NMR (376 MHz, $CDCl_3$)

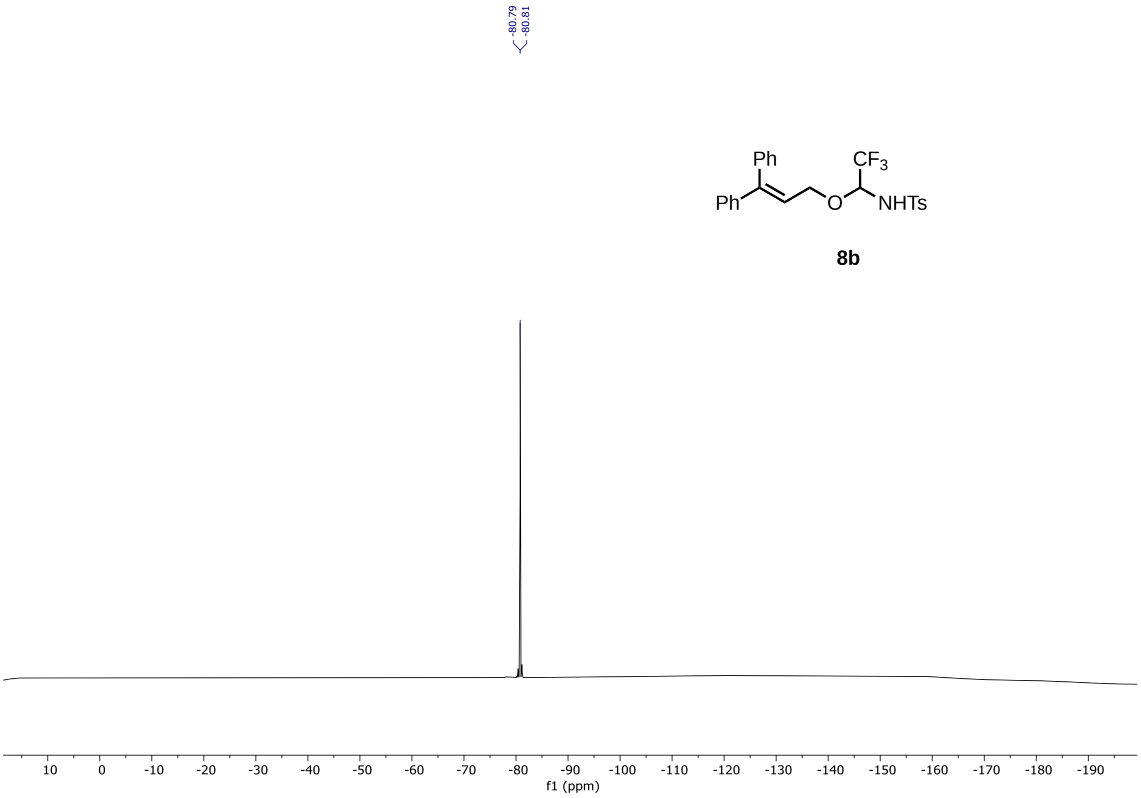

^{1}H NMR (400 MHz, $CDCl_3$)

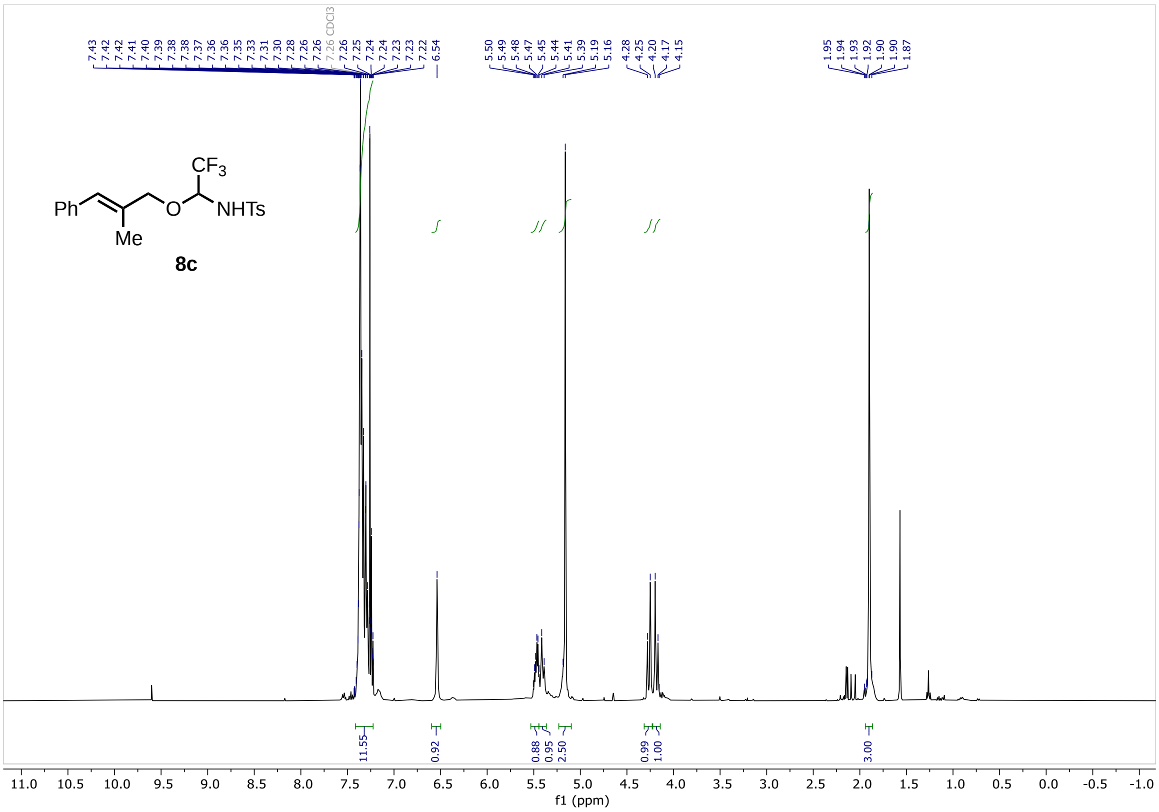

^{13}C NMR (101 MHz, $CDCl_3$)

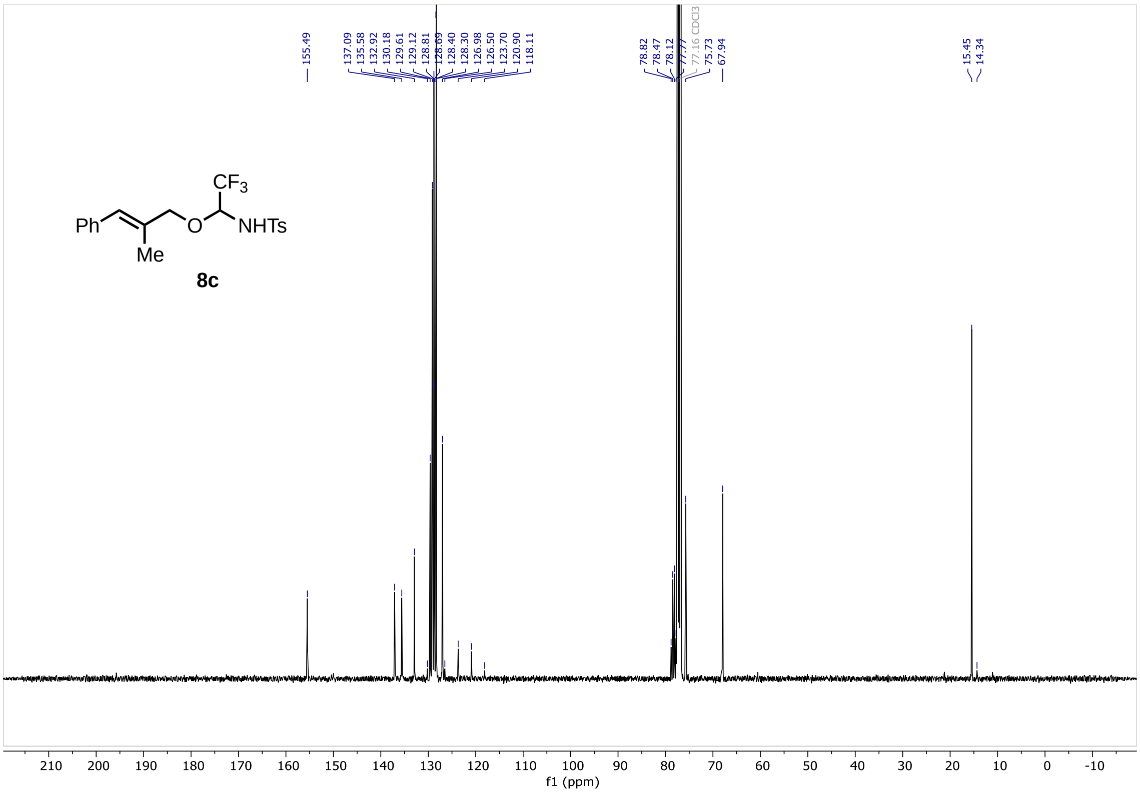

^{19}F NMR (376 MHz, CDCl$_3$)

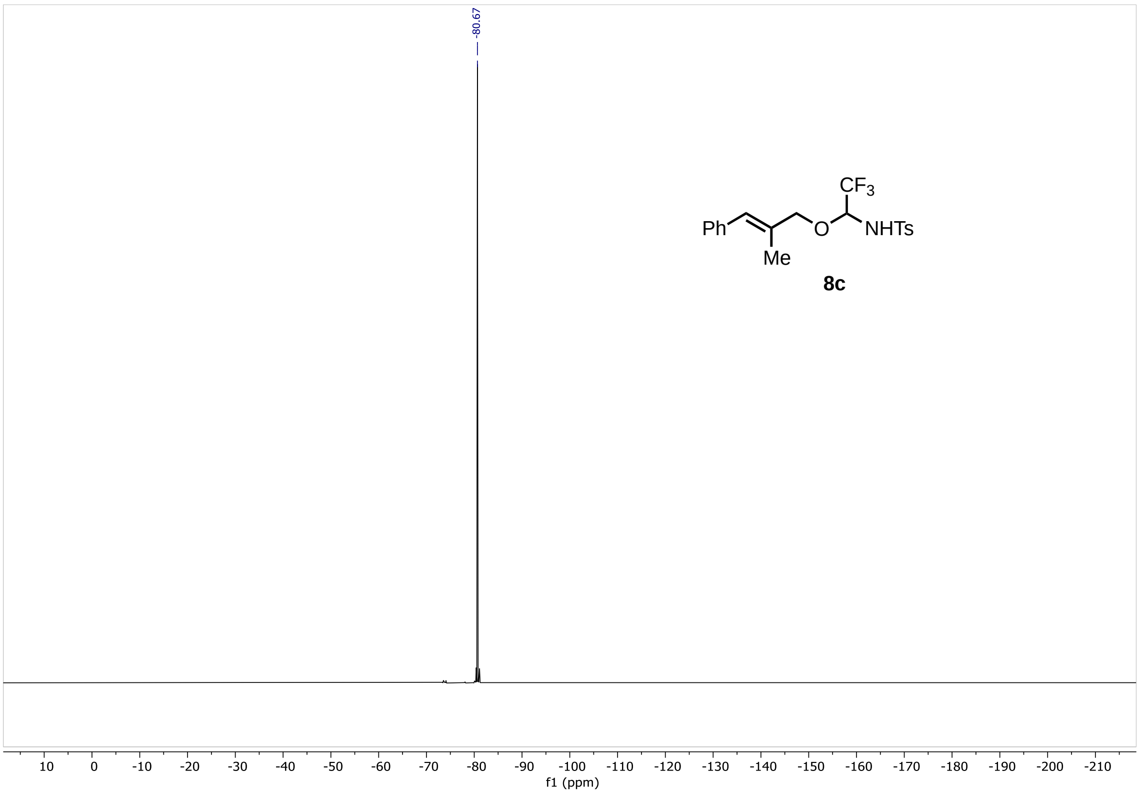

^{1}H NMR (400 MHz, CDCl$_3$)

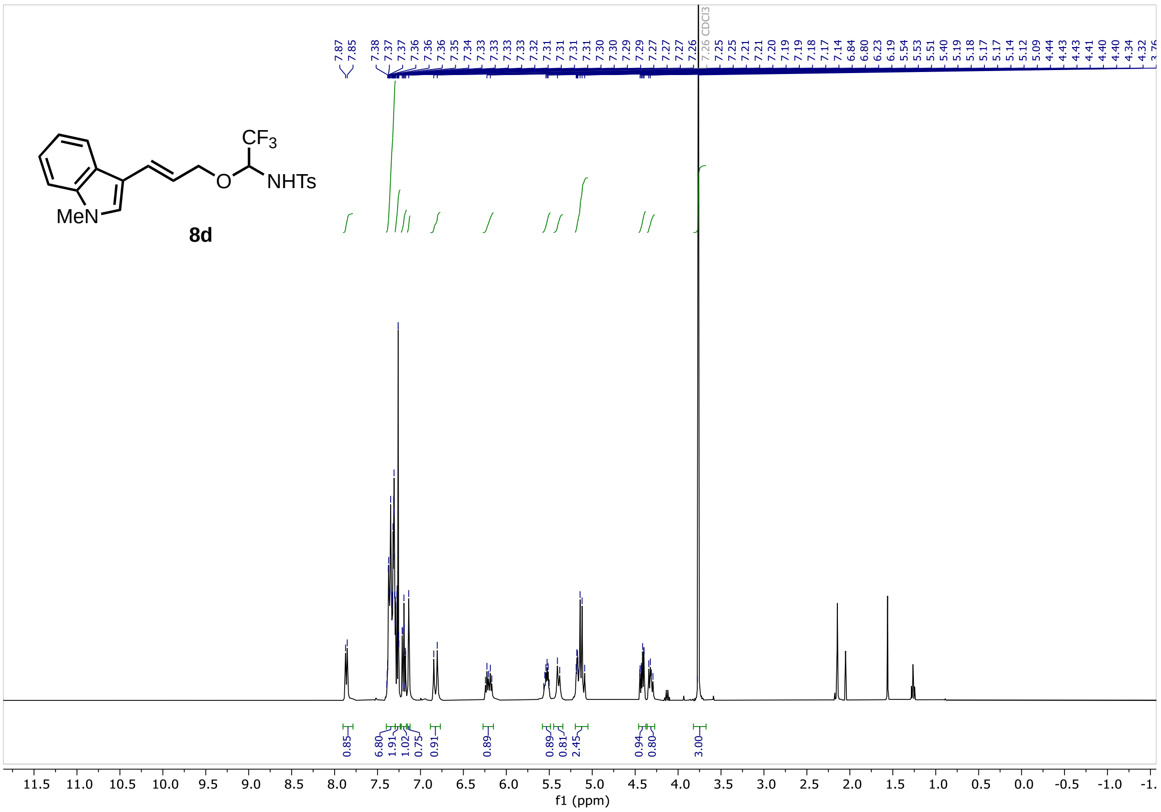

^{13}C NMR (101 MHz, $CDCl_3$)

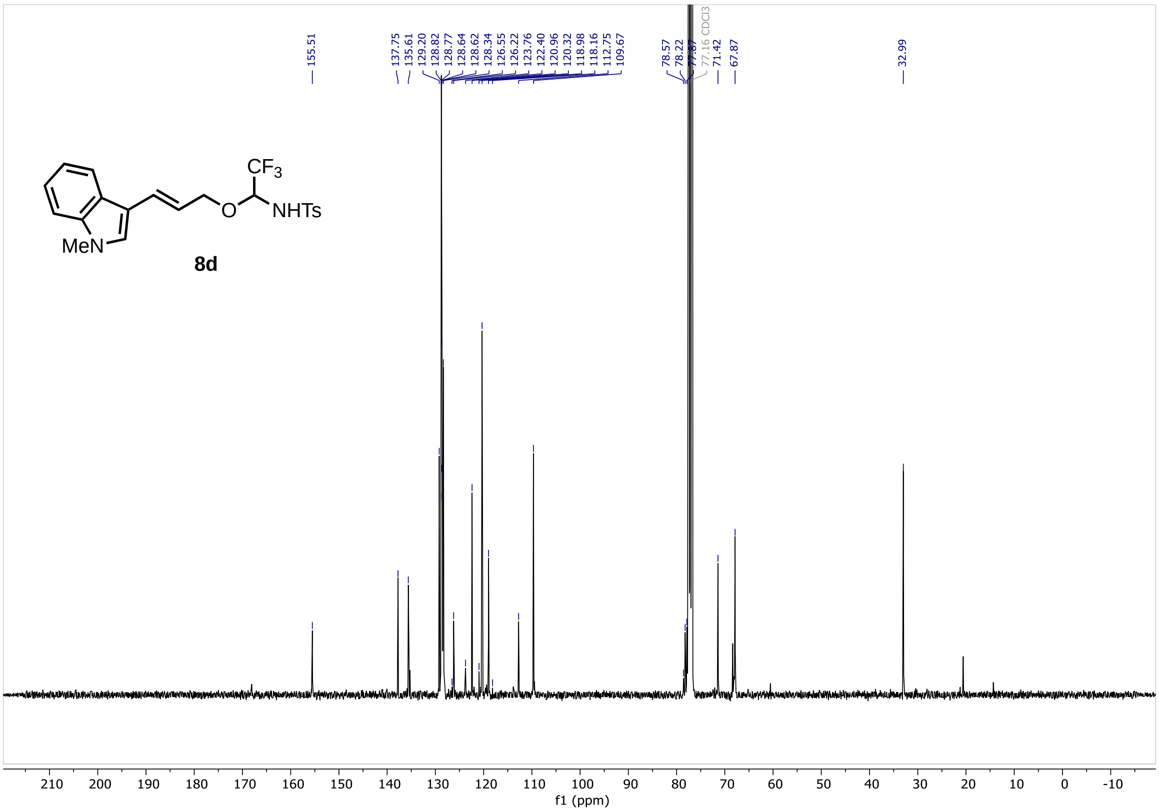

^{19}F NMR (376 MHz, $CDCl_3$)

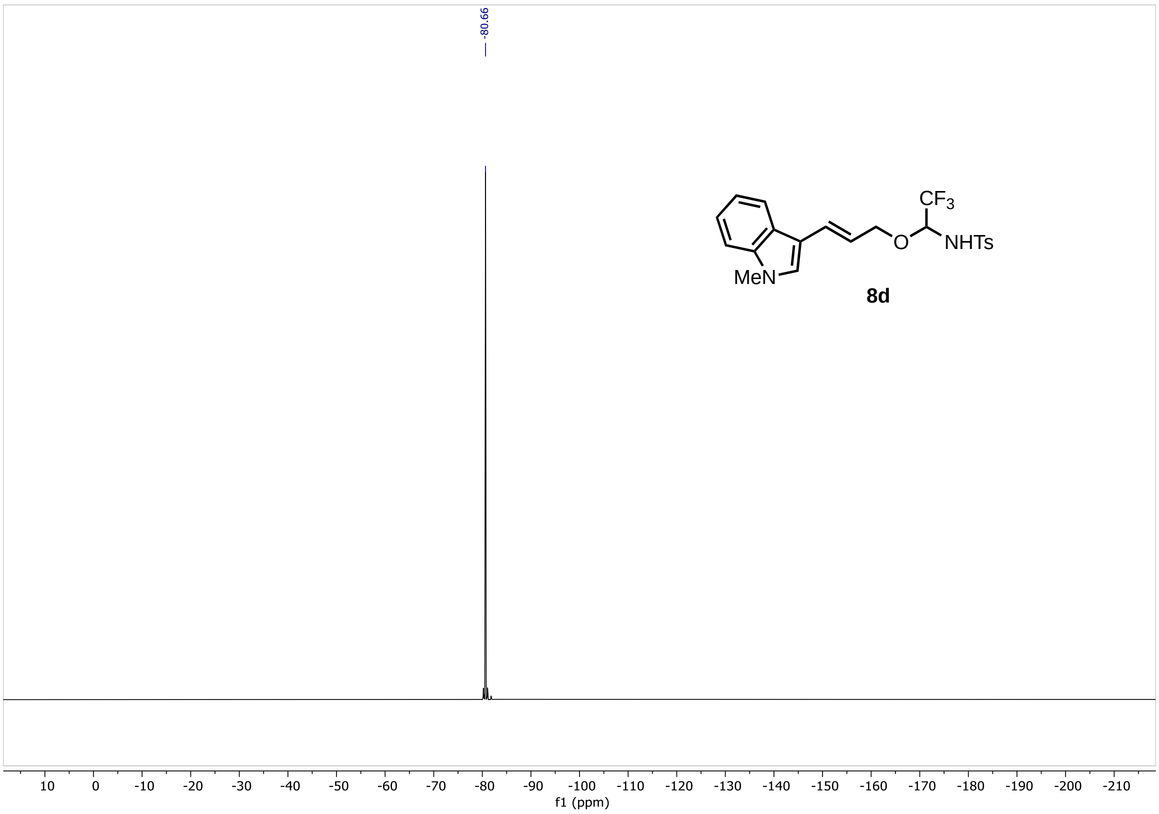

^{1}H NMR (400 MHz, $CDCl_3$)

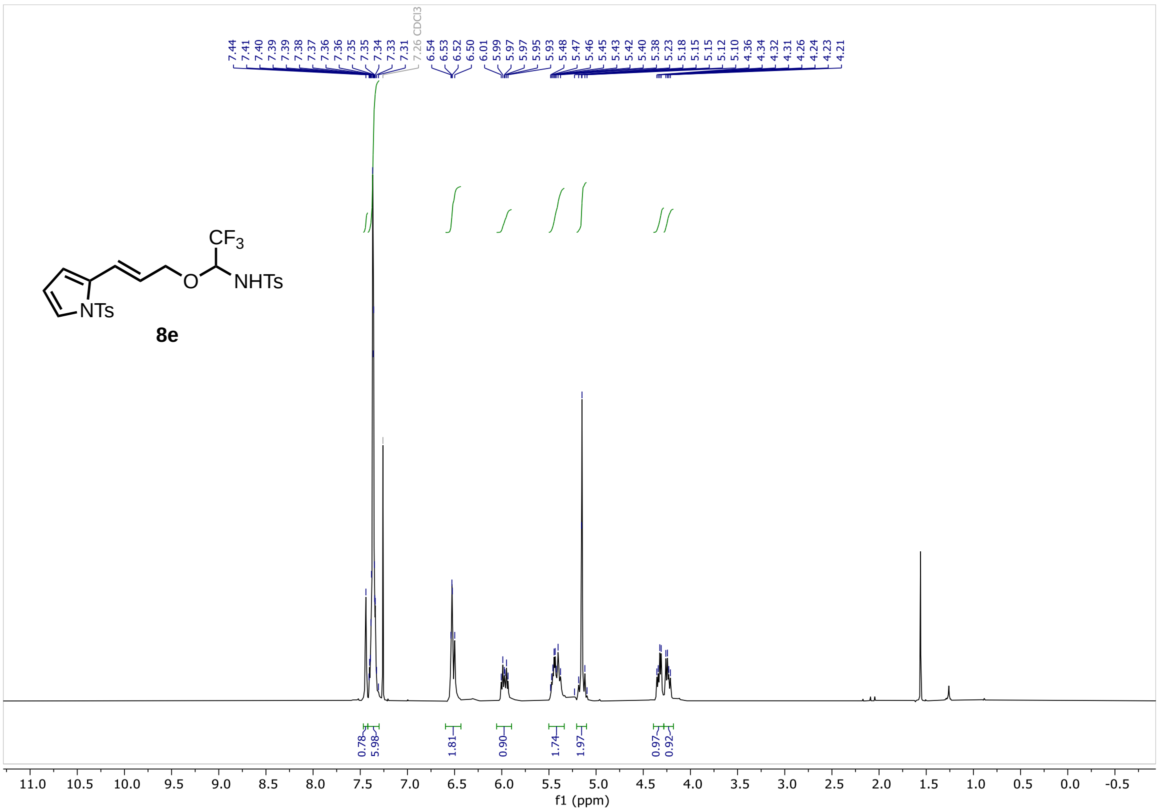

^{13}C NMR (101 MHz, $CDCl_3$)

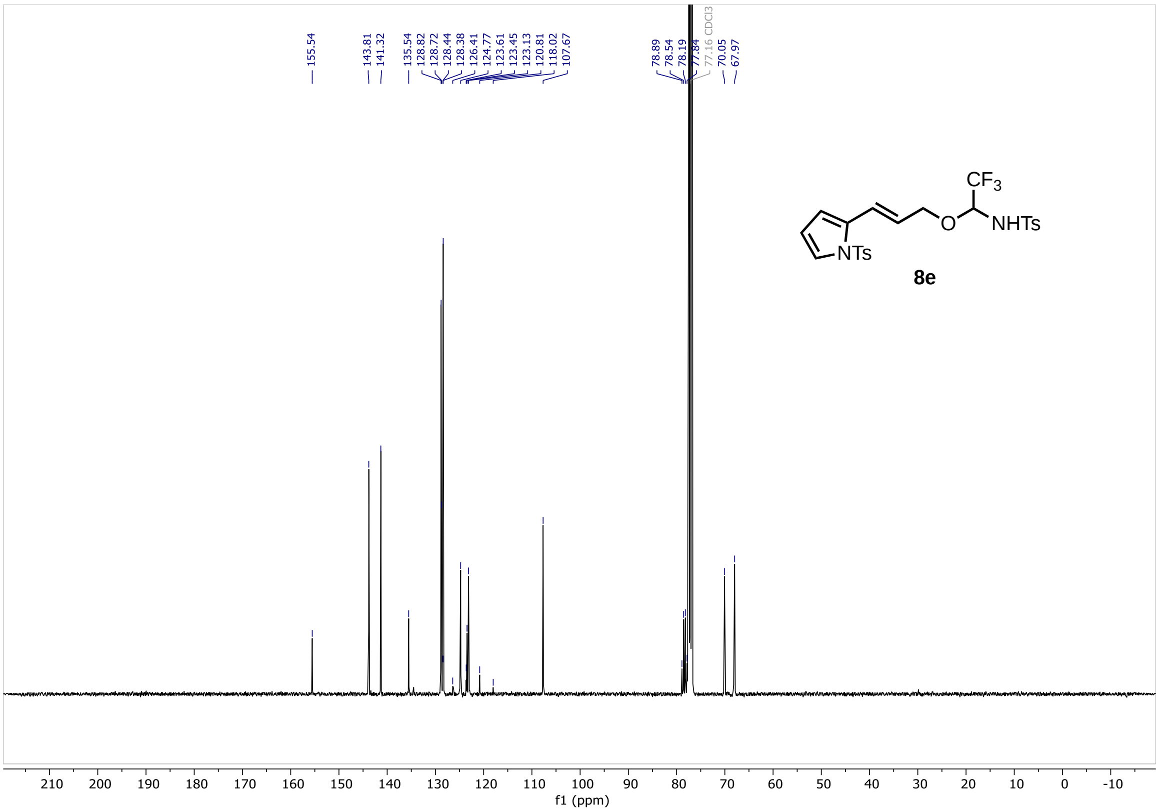

^{19}F NMR (376 MHz, $CDCl_3$)

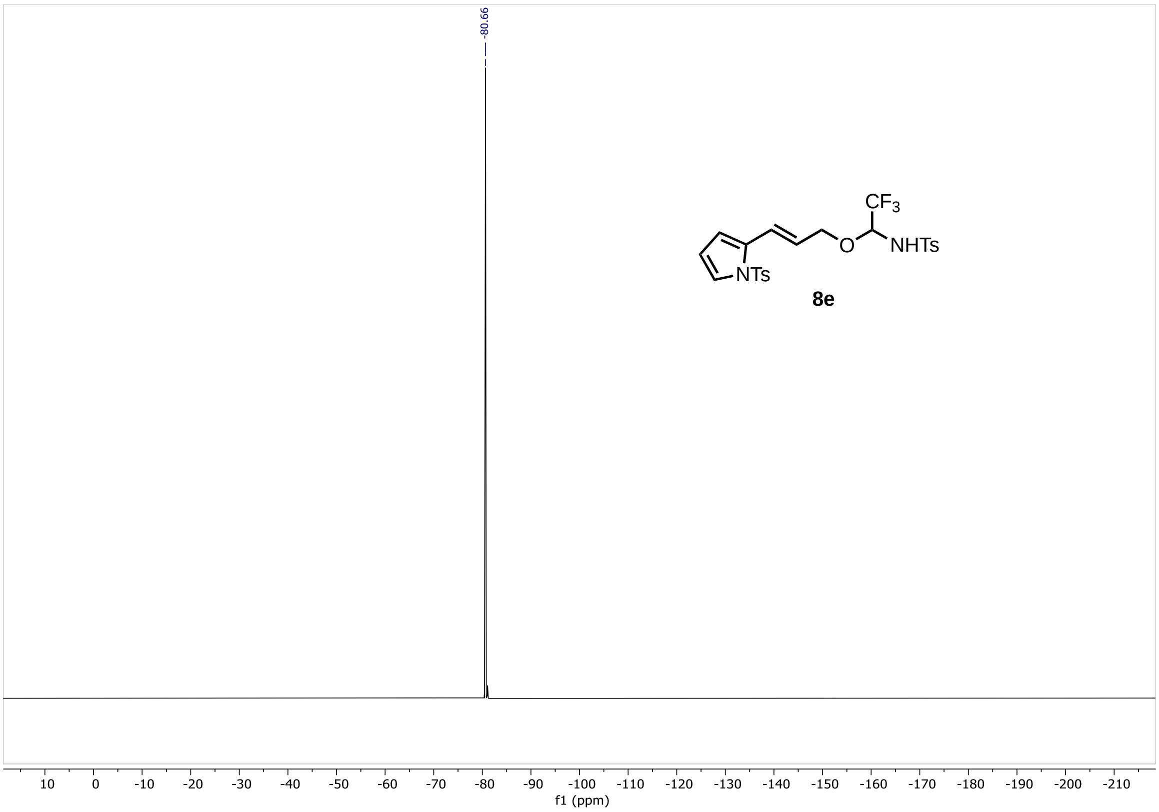

^{1}H NMR (400 MHz, CD_3CN)

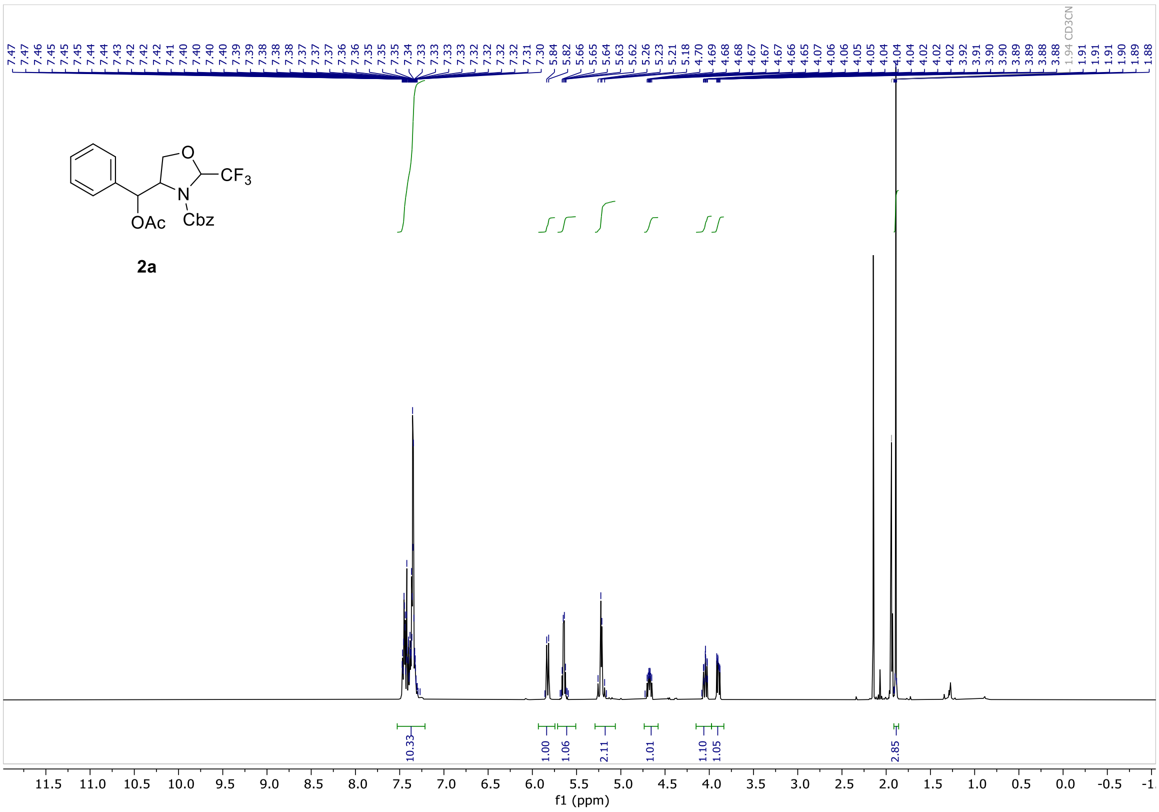

^{13}C NMR (101 MHz, CD_3CN)

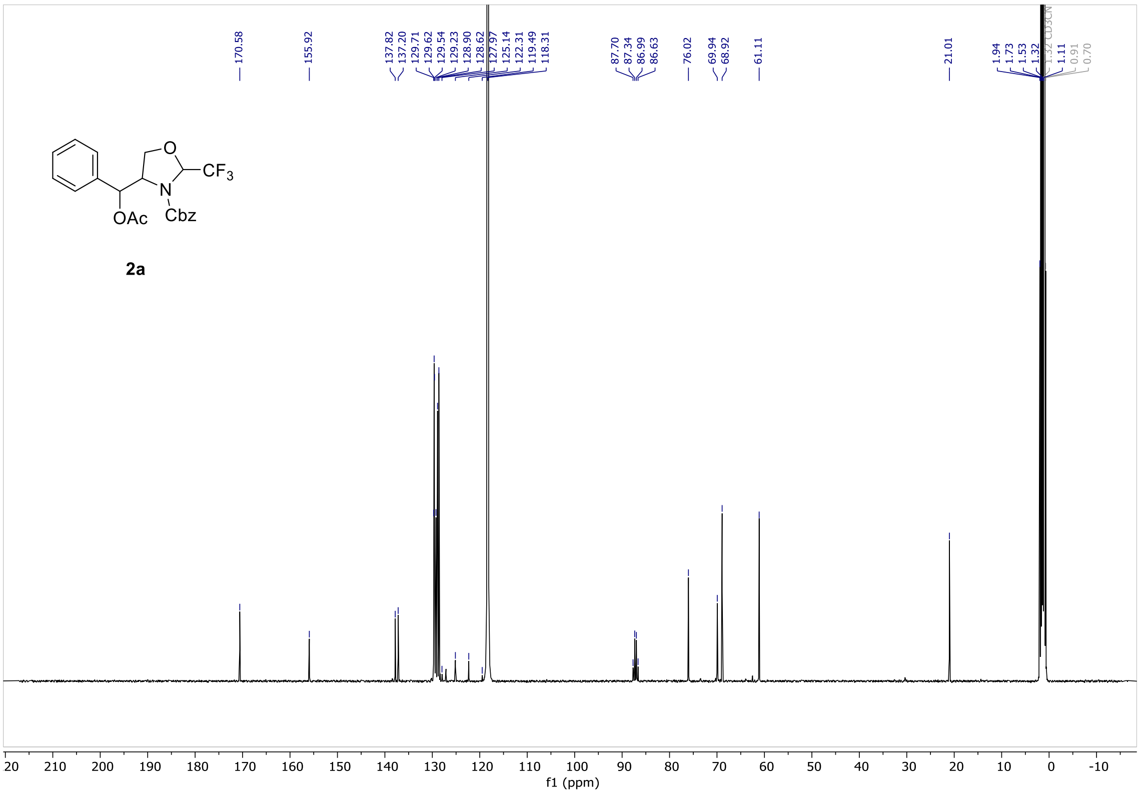

^{19}F NMR (376 MHz, CD_3CN)

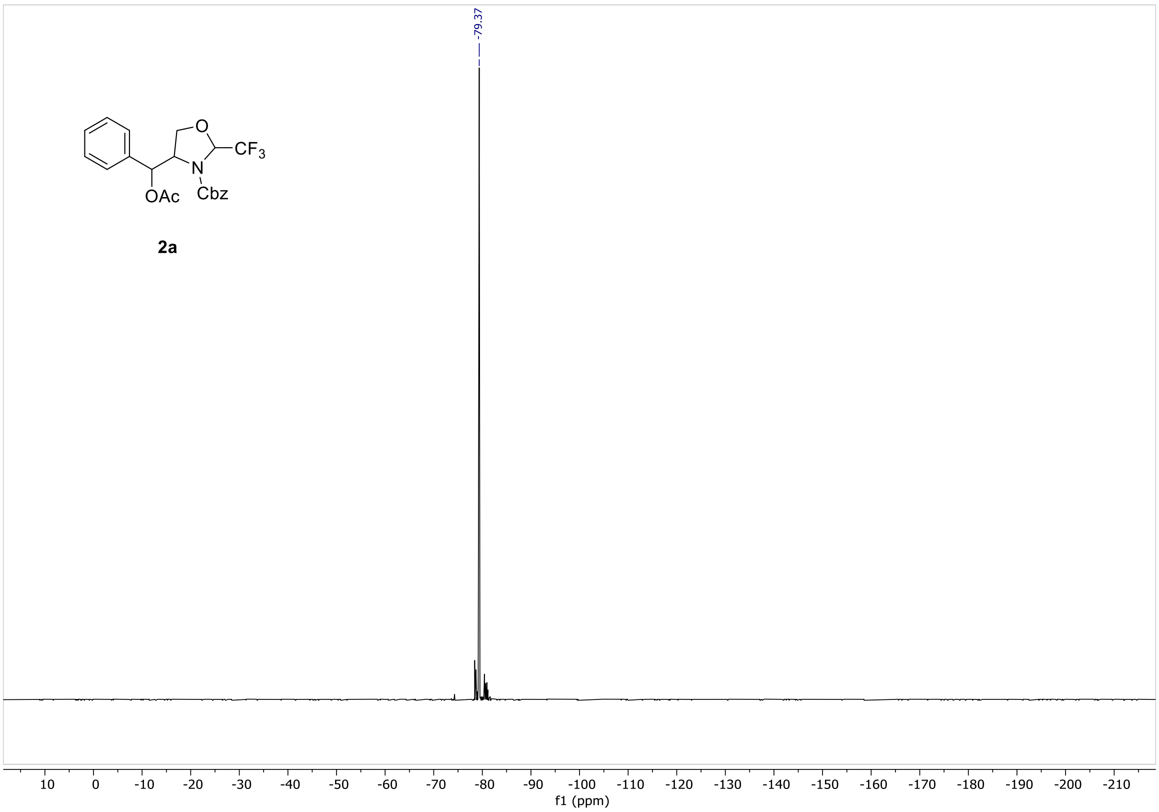

^{1}H NMR (400 MHz, $CDCl_3$)

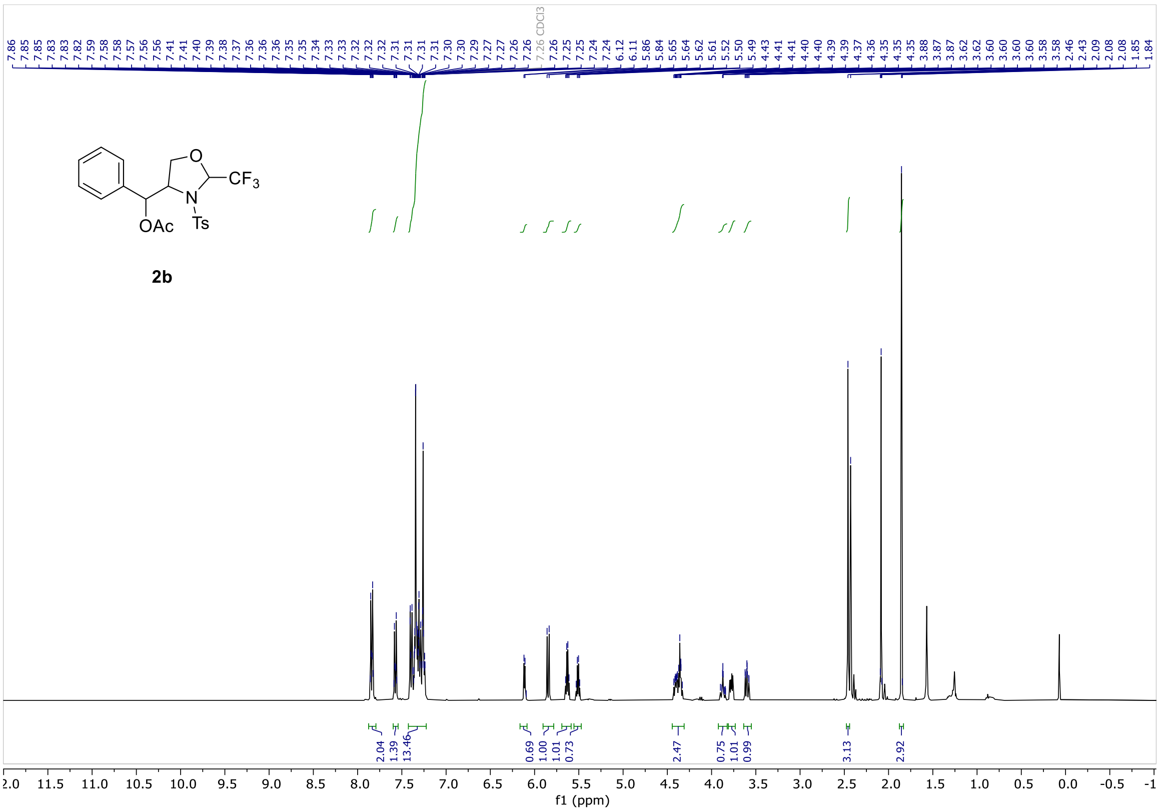

^{13}C NMR (101 MHz, $CDCl_3$)

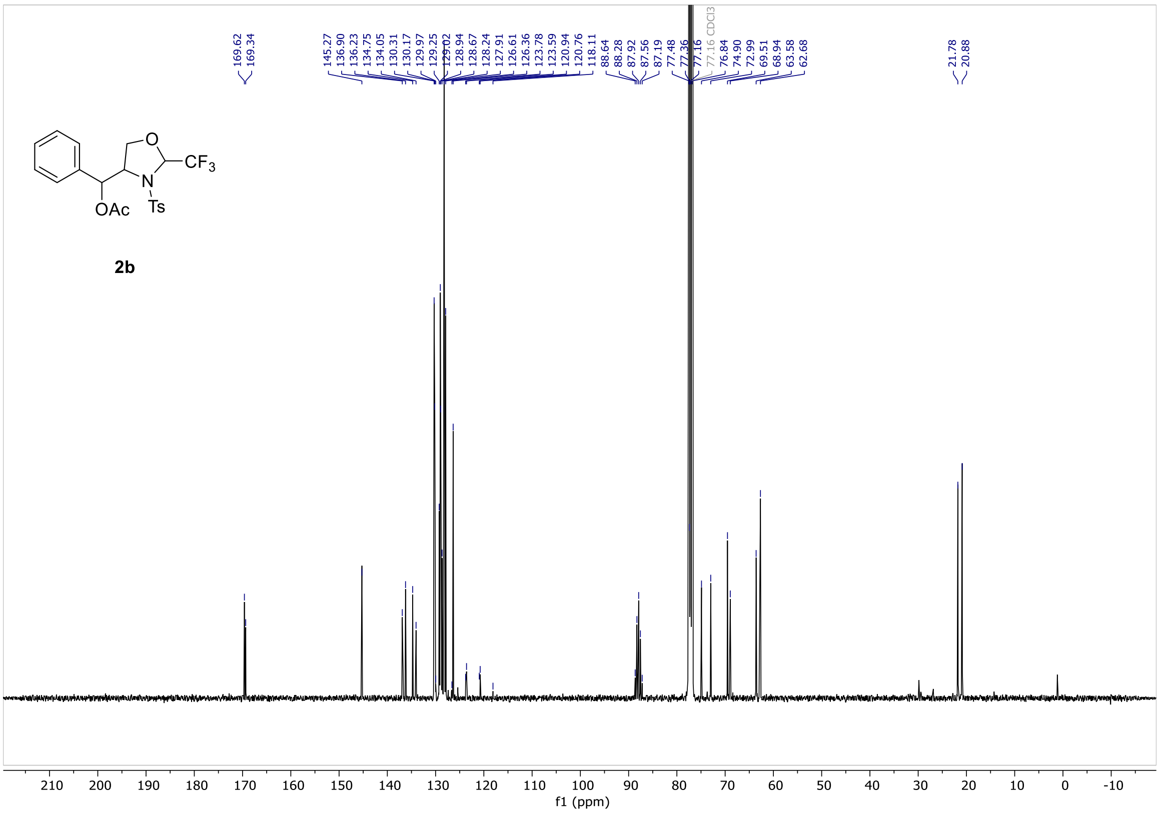

^{19}F NMR (376 MHz, $CDCl_3$)

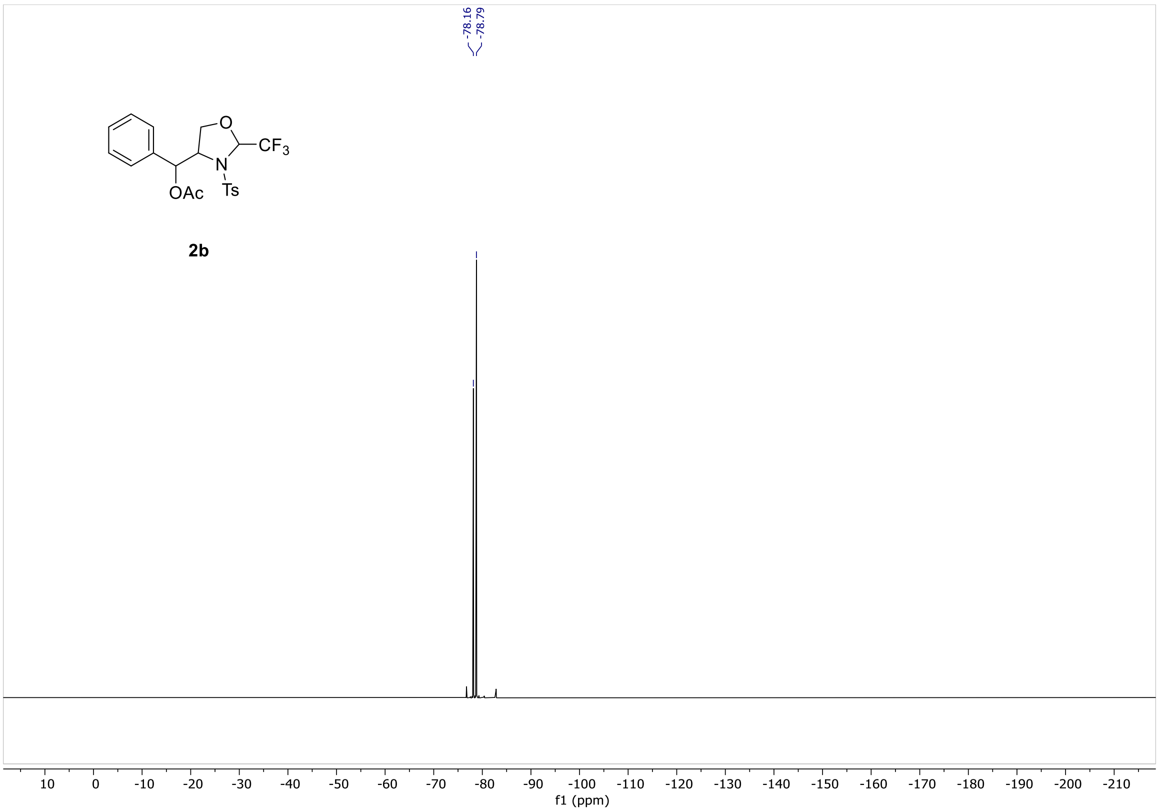

^{1}H NMR (400 MHz, $CDCl_3$)

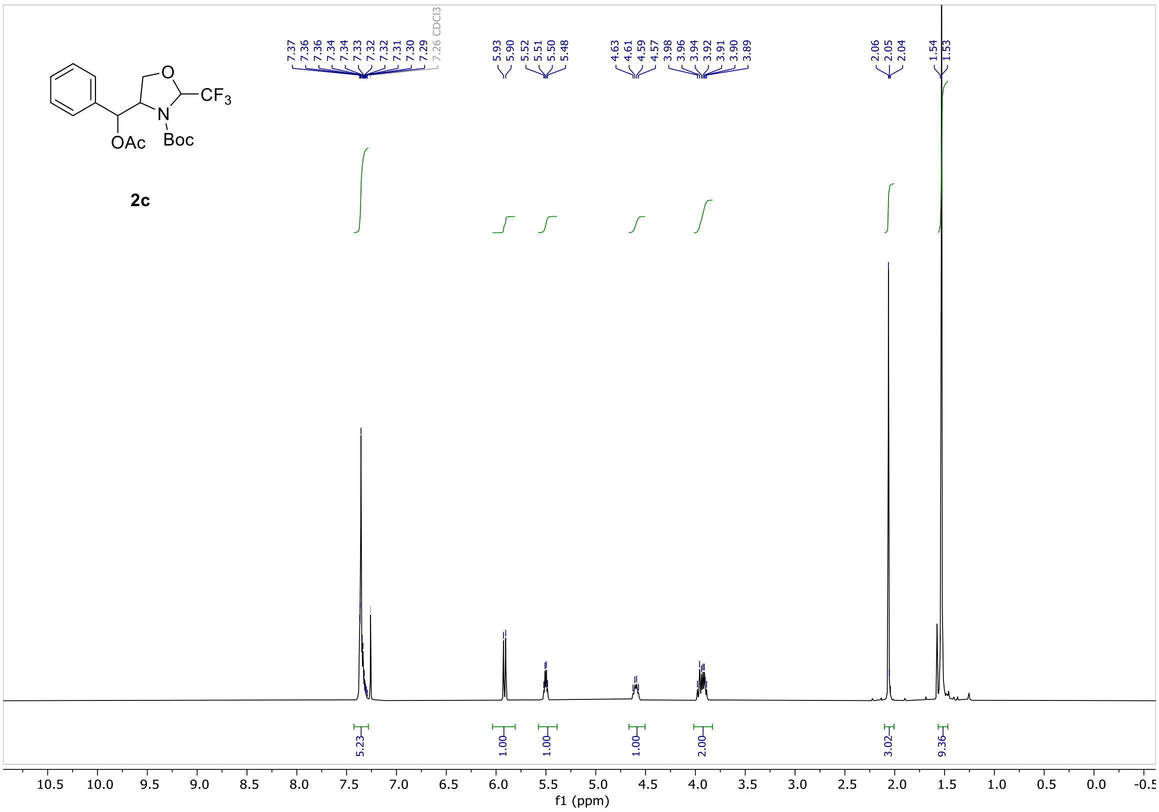

^{13}C NMR (101 MHz, $CDCl_3$)

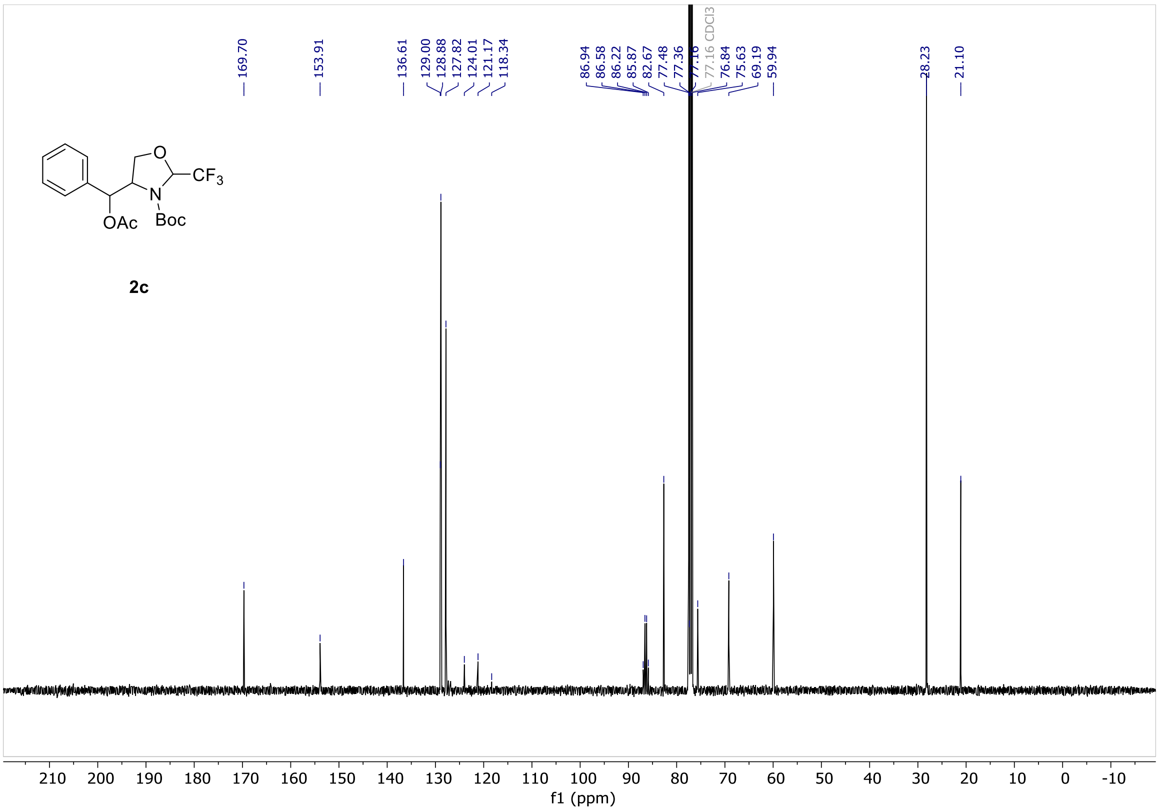

^{19}F NMR (376 MHz, $CDCl_3$)

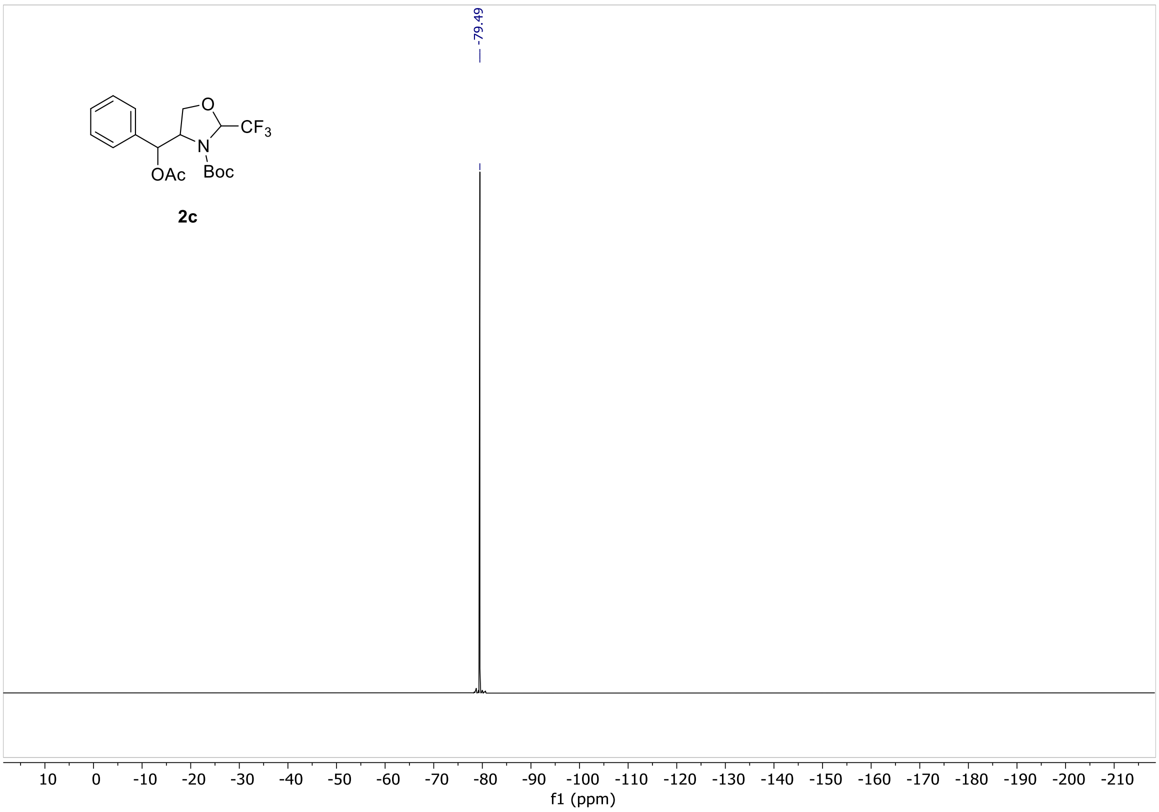

^{1}H NMR (400 MHz, $CDCl_3$)

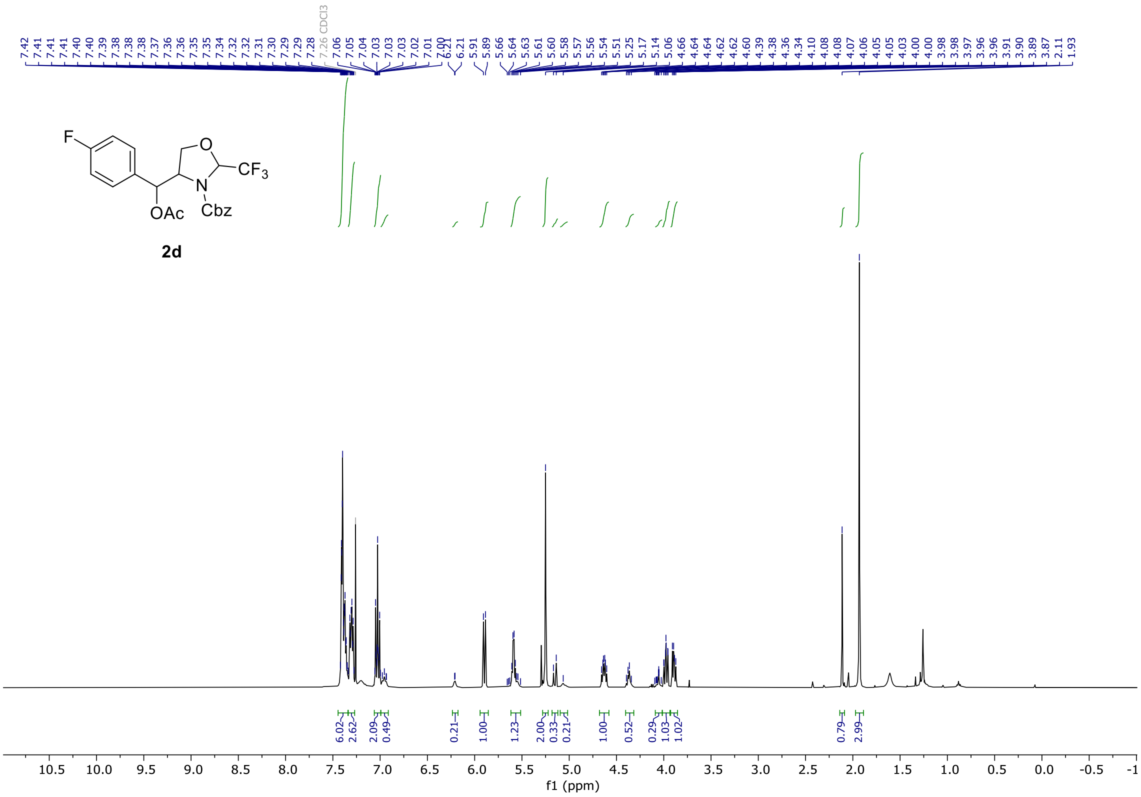

^{13}C NMR (101 MHz, $CDCl_3$)

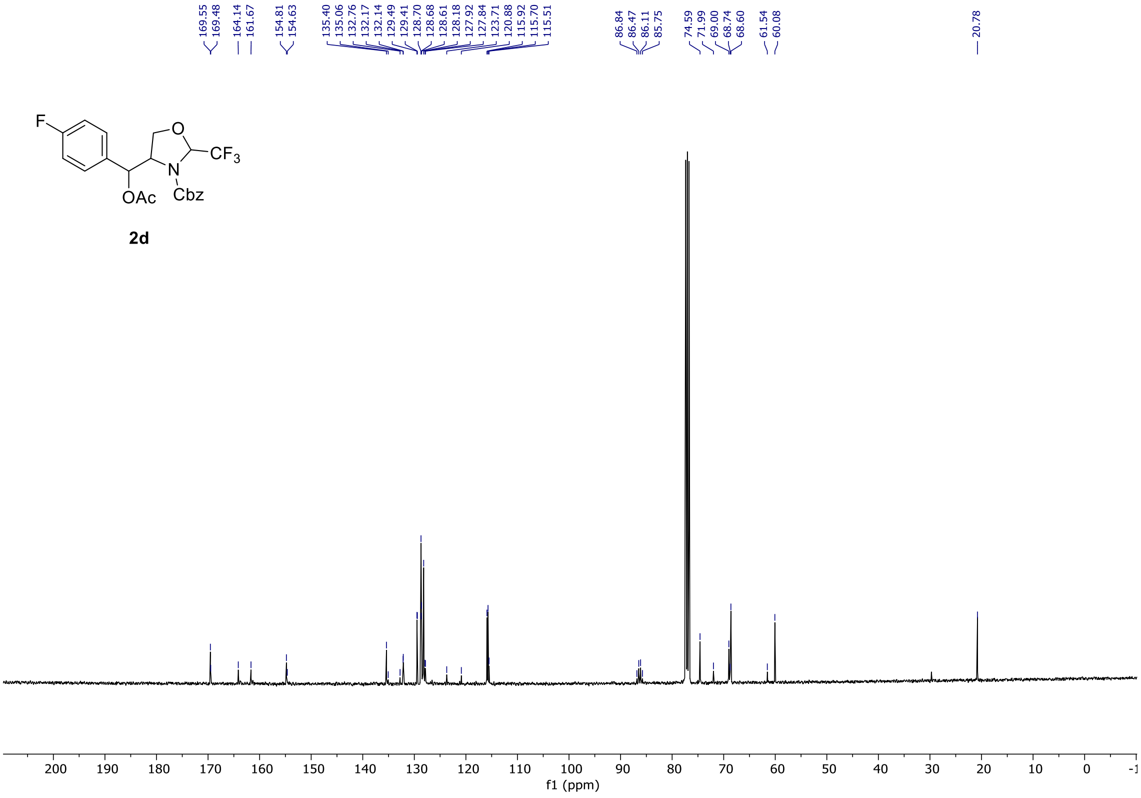

^{19}F NMR (376 MHz, $CDCl_3$)

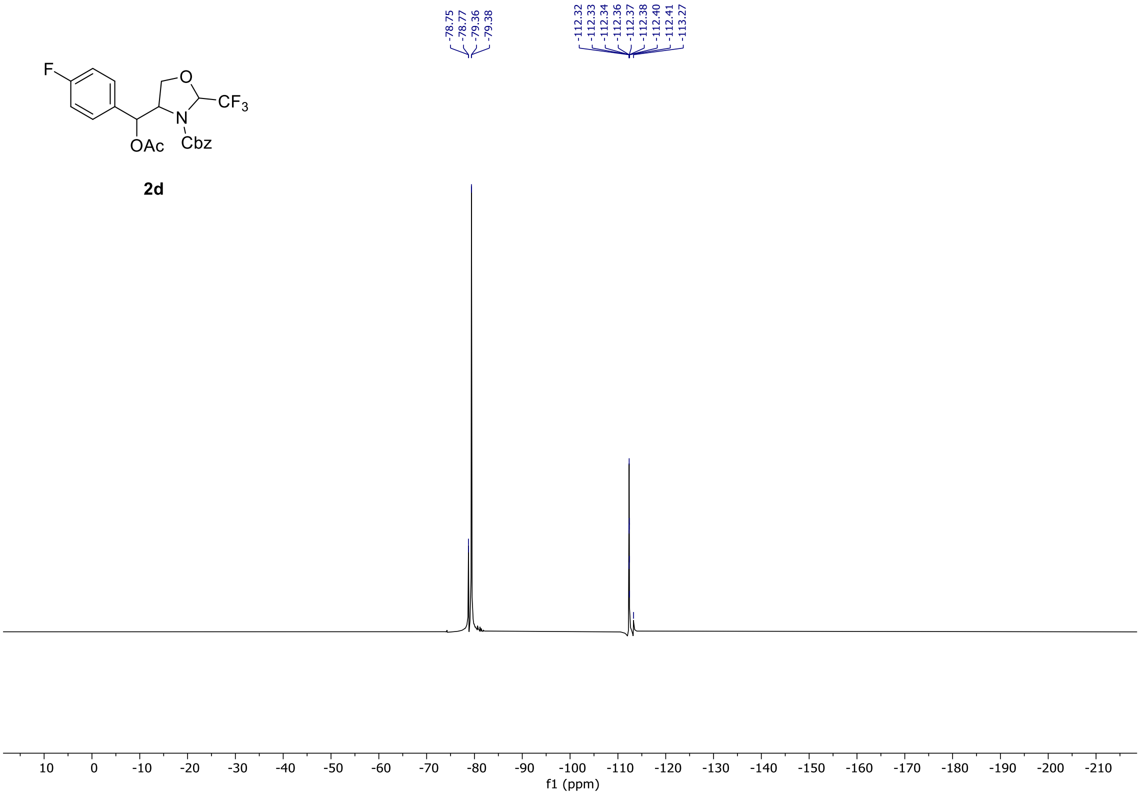

^{1}H NMR (400 MHz, $CDCl_3$)

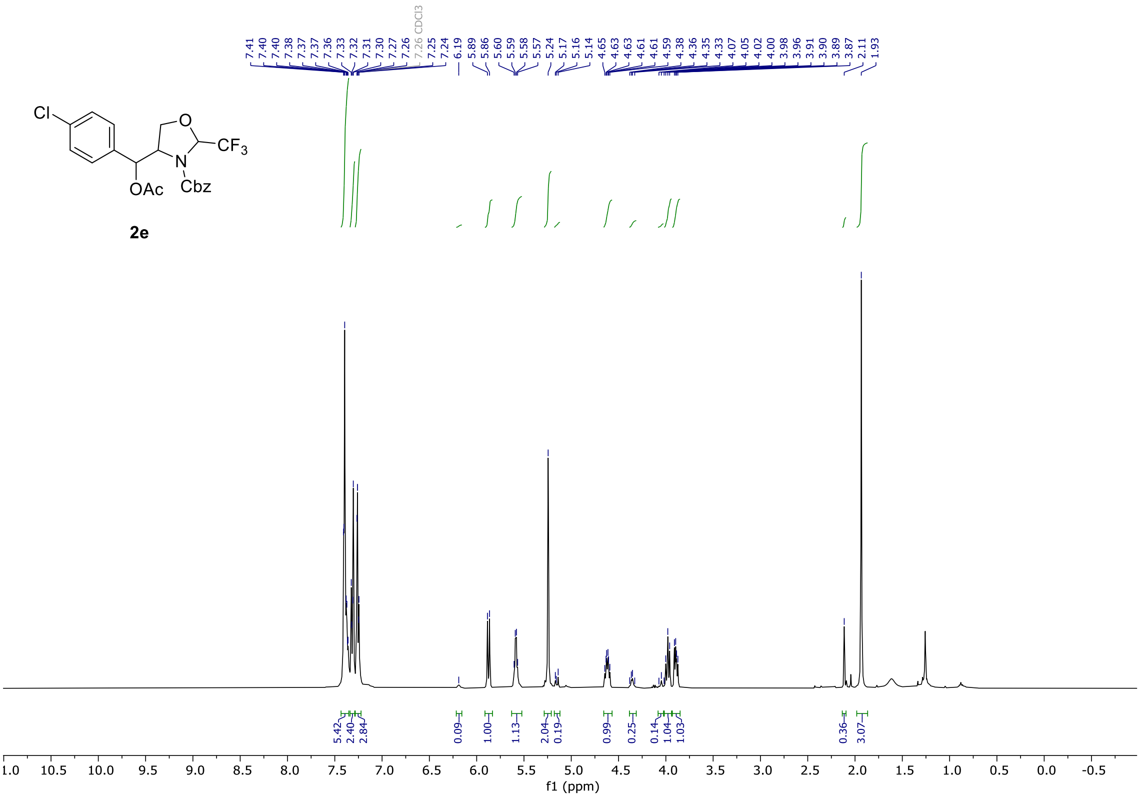

^{13}C NMR (101 MHz, $CDCl_3$)

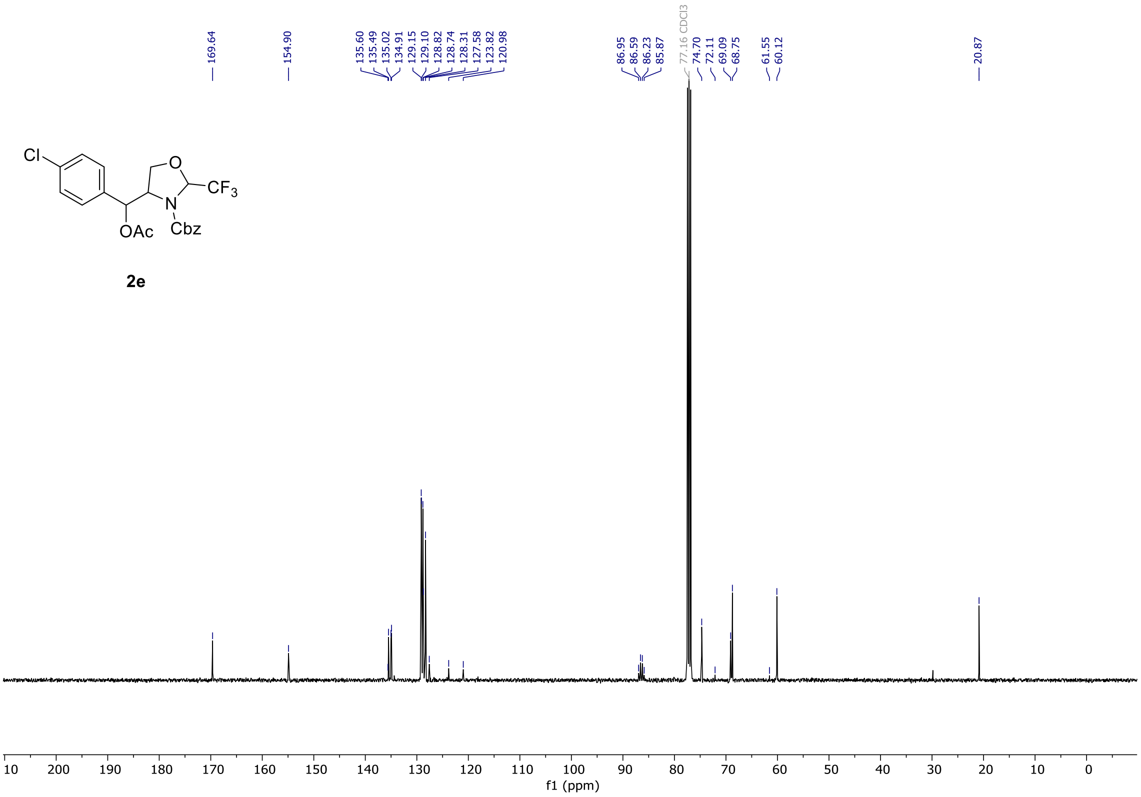

^{19}F NMR (376 MHz, $CDCl_3$)

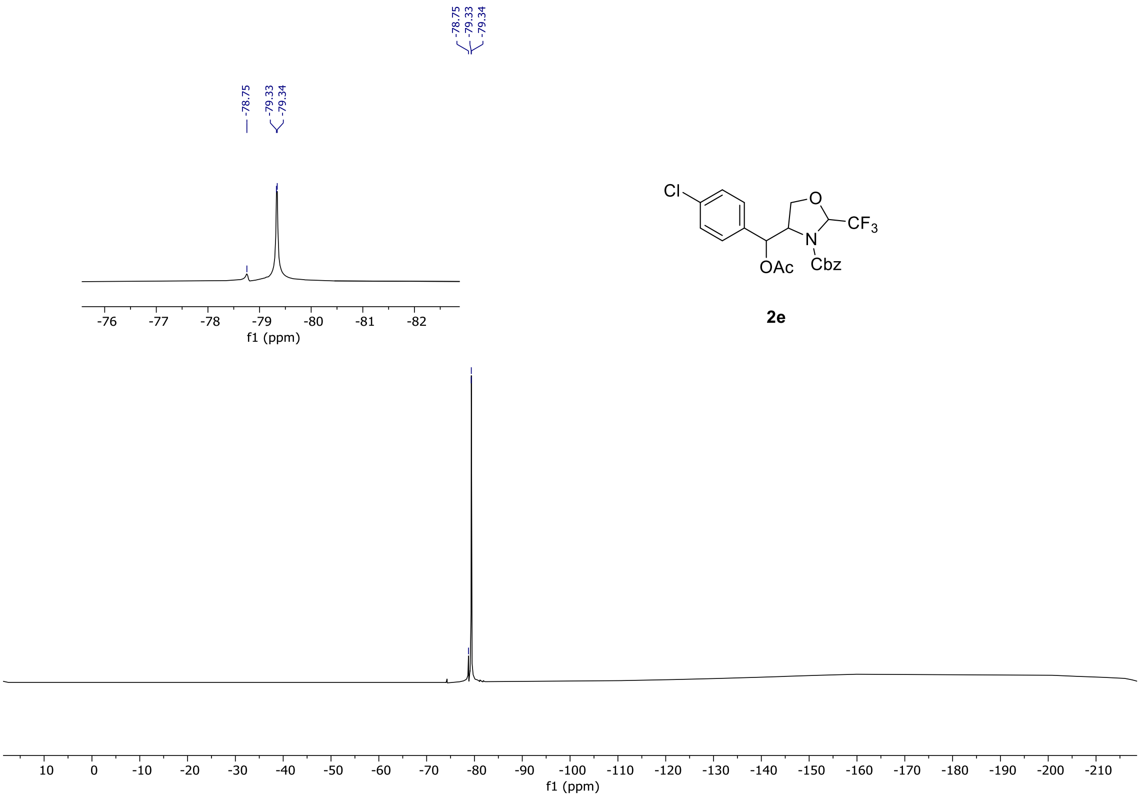

^{1}H NMR (400 MHz, $CDCl_3$)

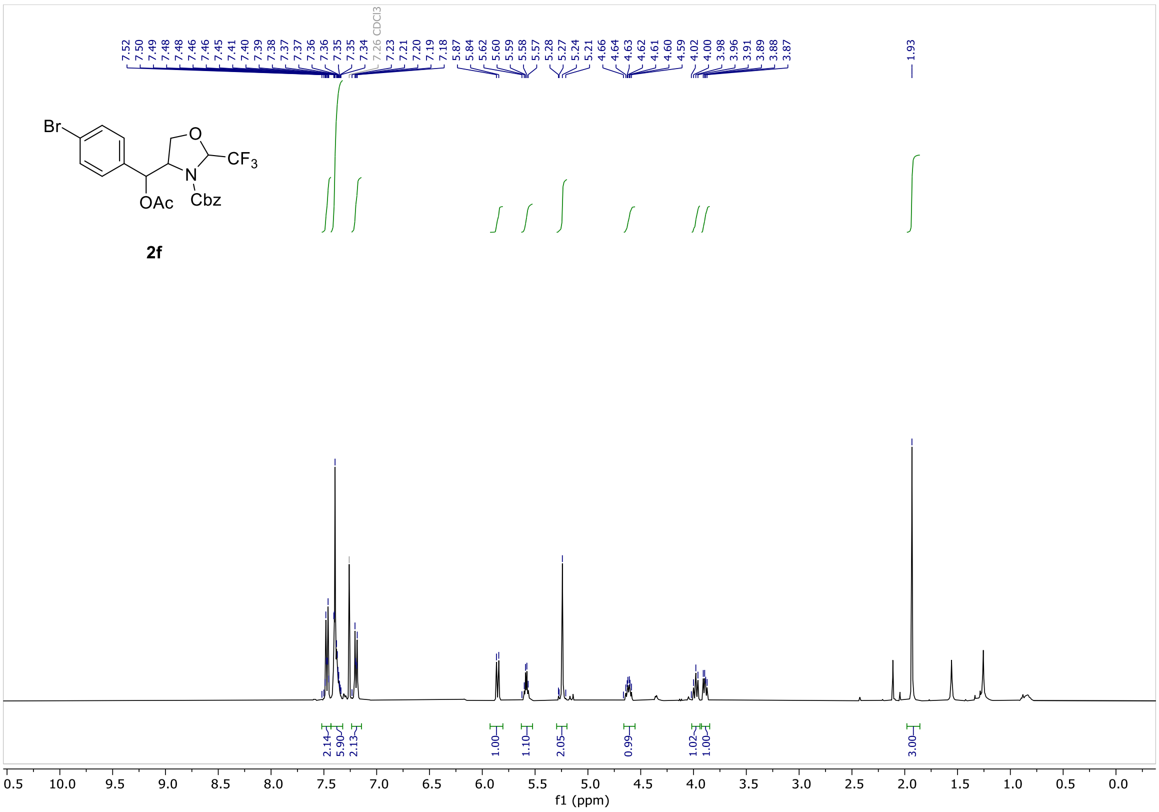

^{13}C NMR (101 MHz, $CDCl_3$)

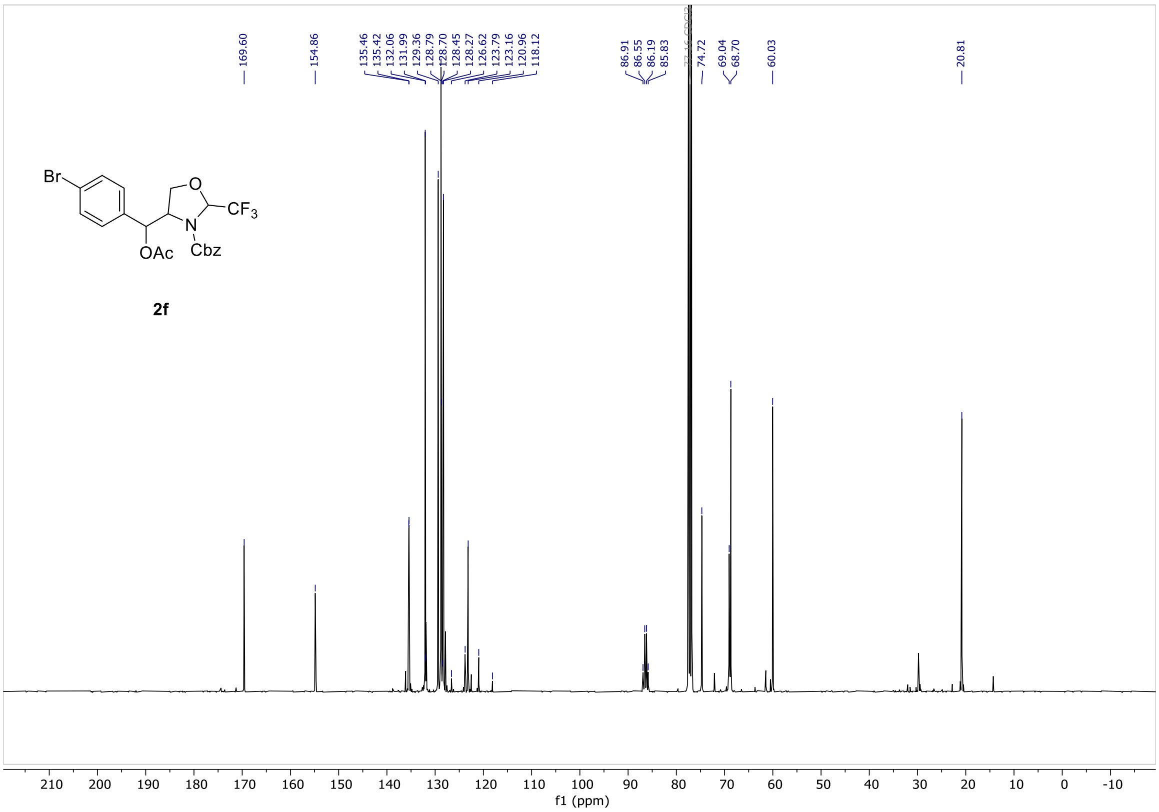

^{19}F NMR (376 MHz, $CDCl_3$)

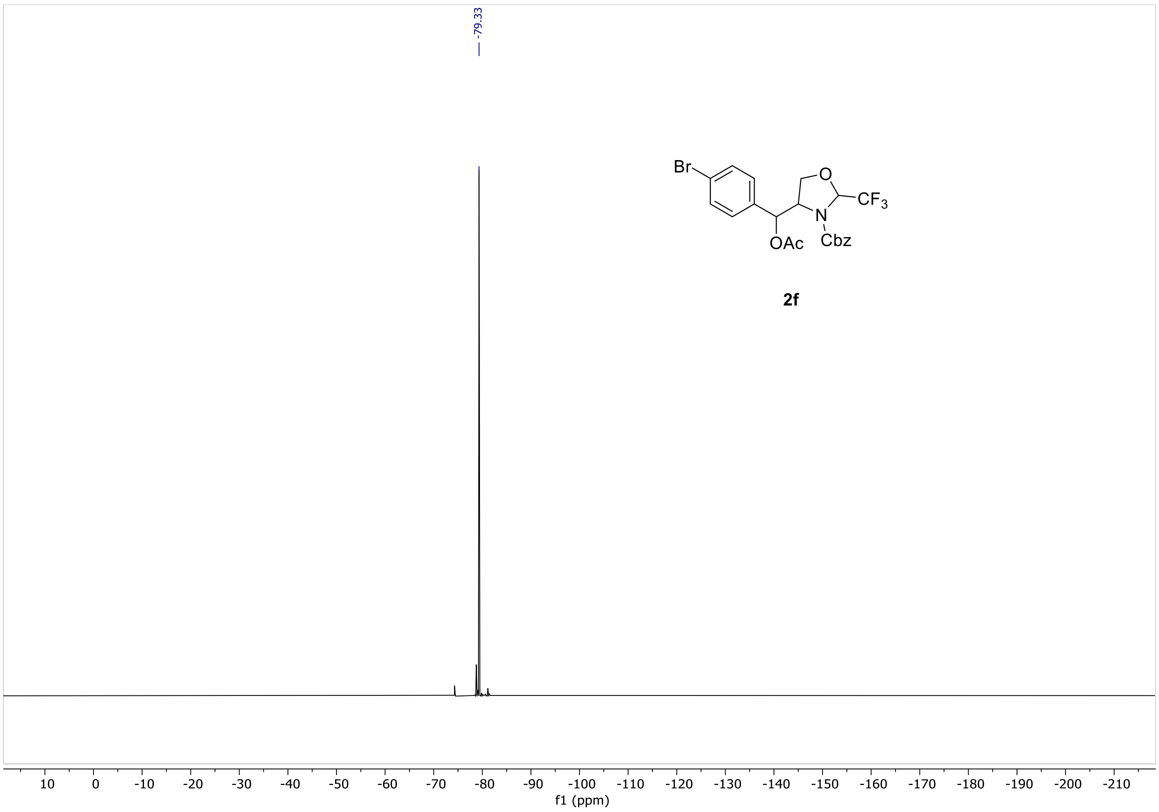

^{1}H NMR (400 MHz, $CDCl_3$)

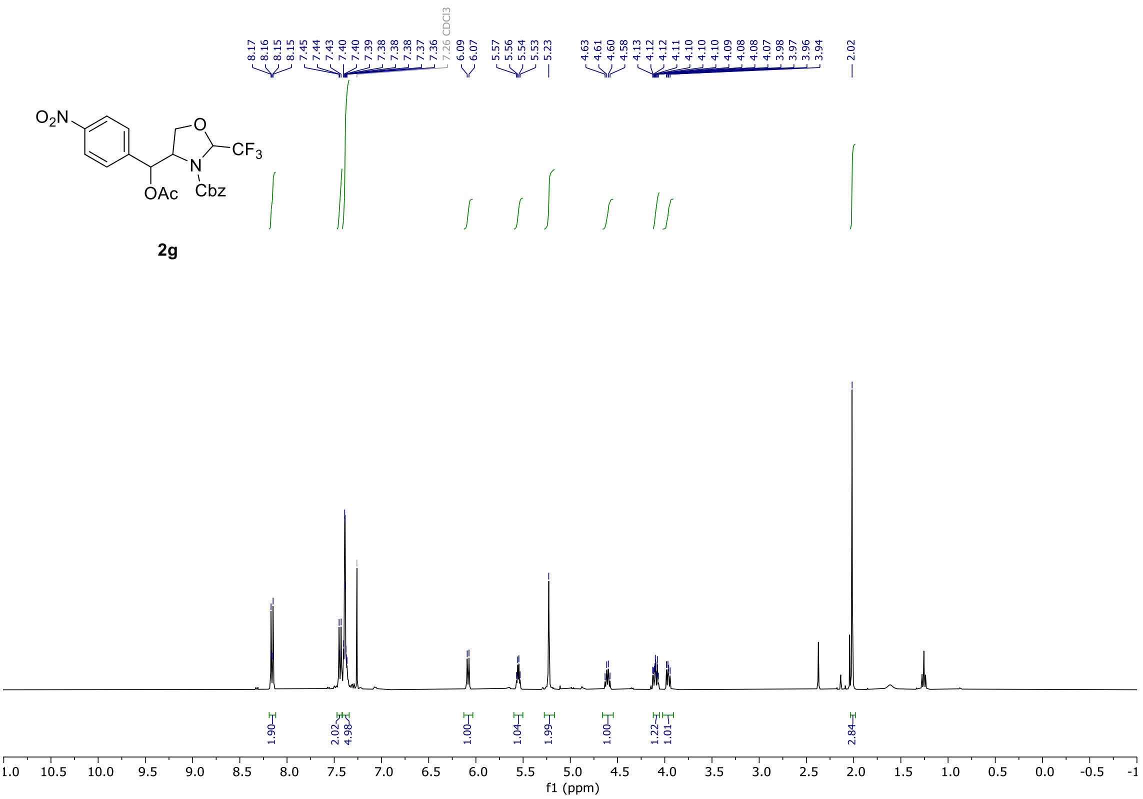

^{13}C NMR (101 MHz, $CDCl_3$)

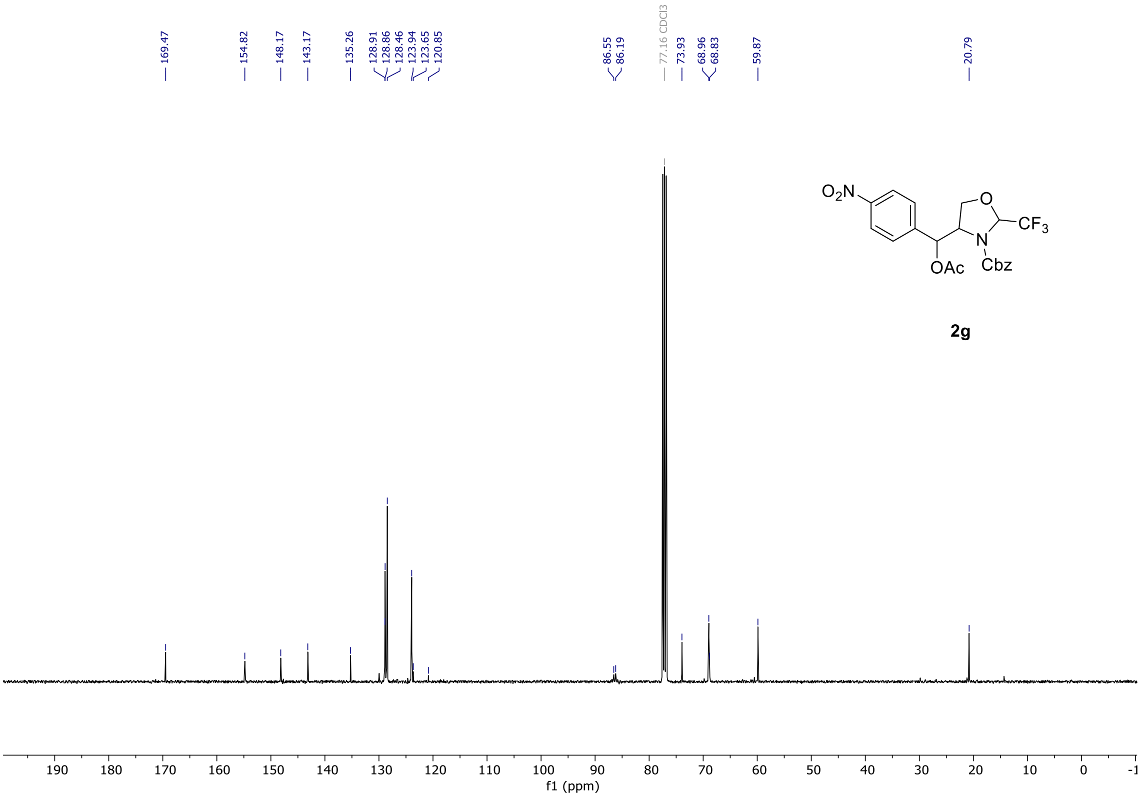

^{19}F NMR (376 MHz, $CDCl_3$)

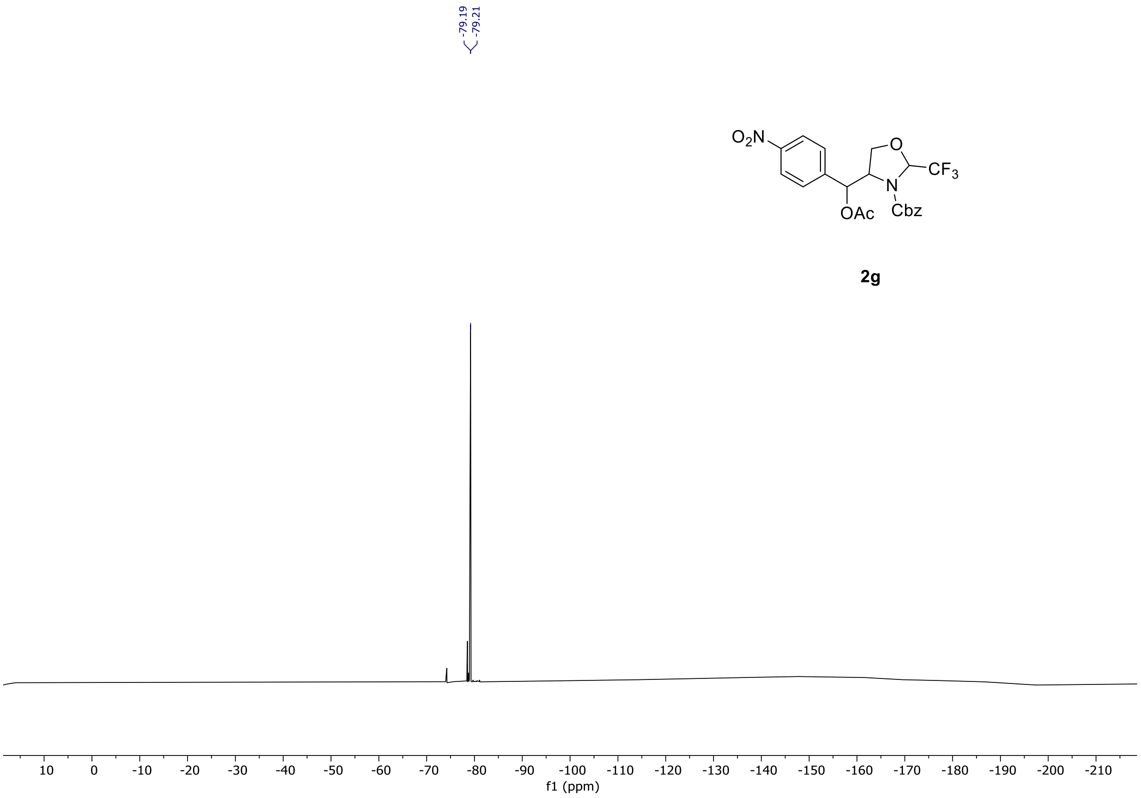

^{1}H NMR (400 MHz, $CDCl_3$)

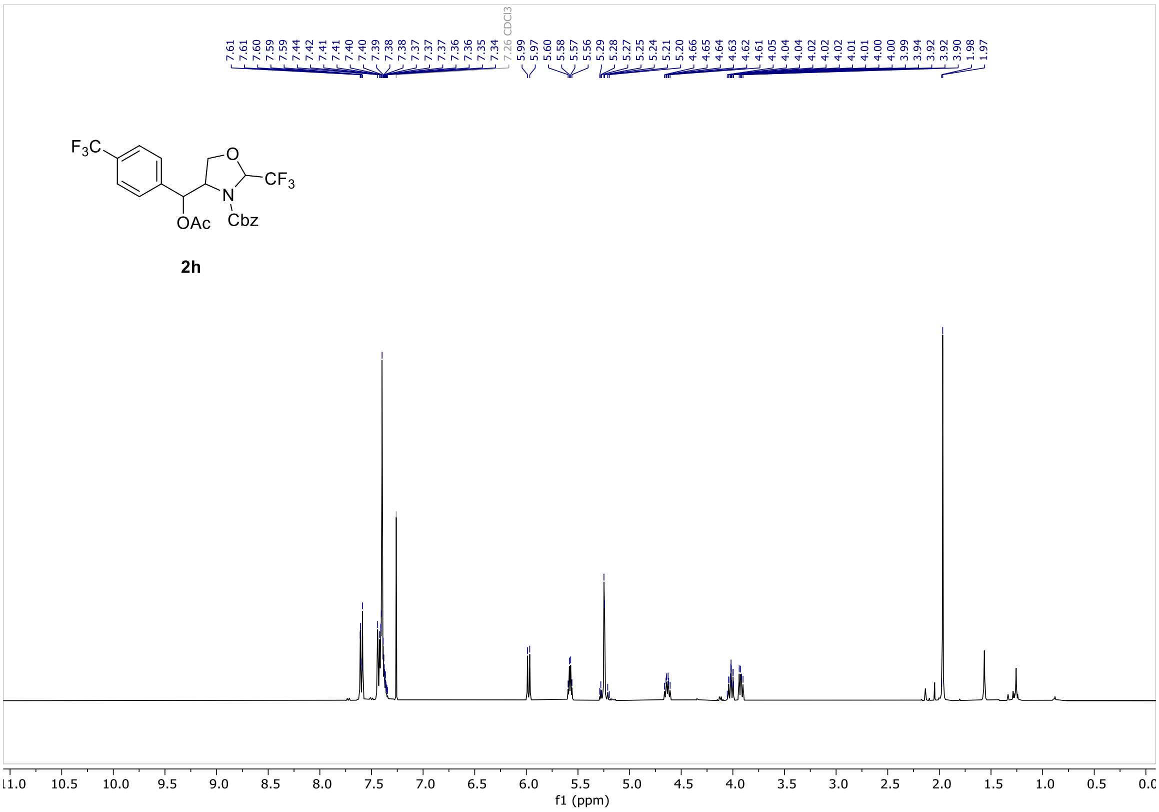

^{13}C NMR (101 MHz, $CDCl_3$)

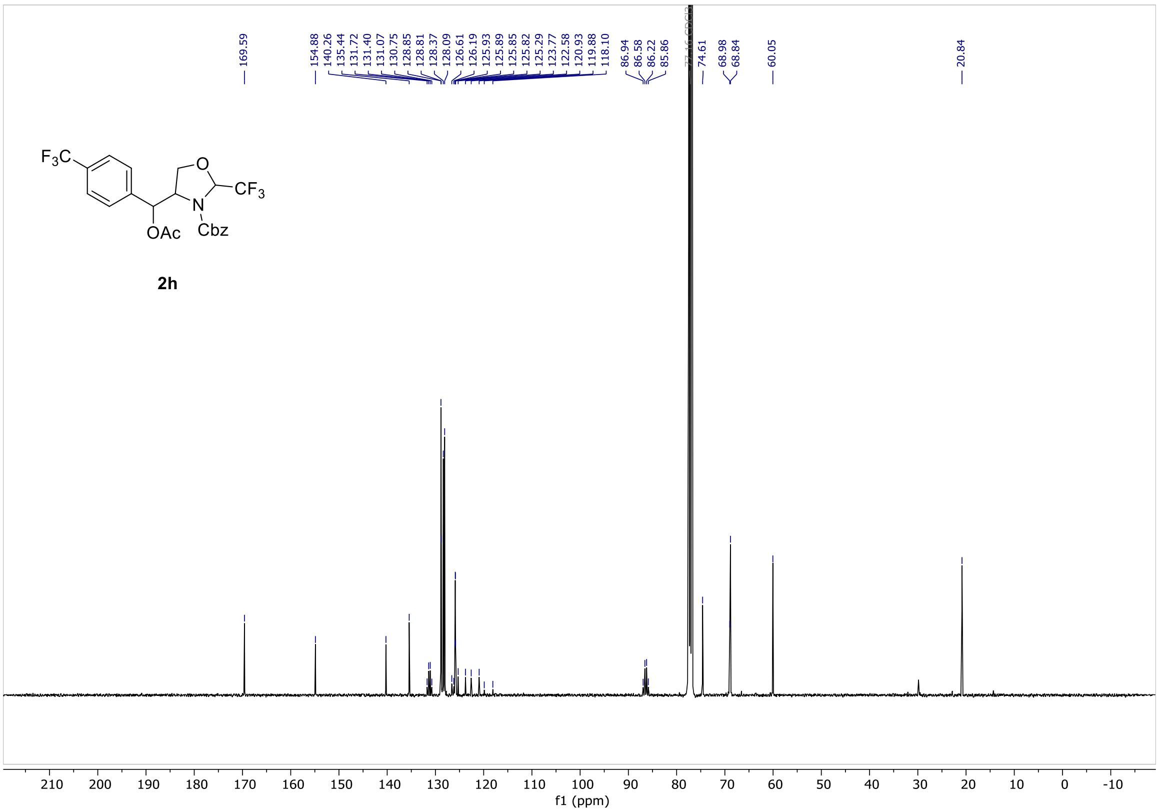

^{19}F NMR (376 MHz, $CDCl_3$)

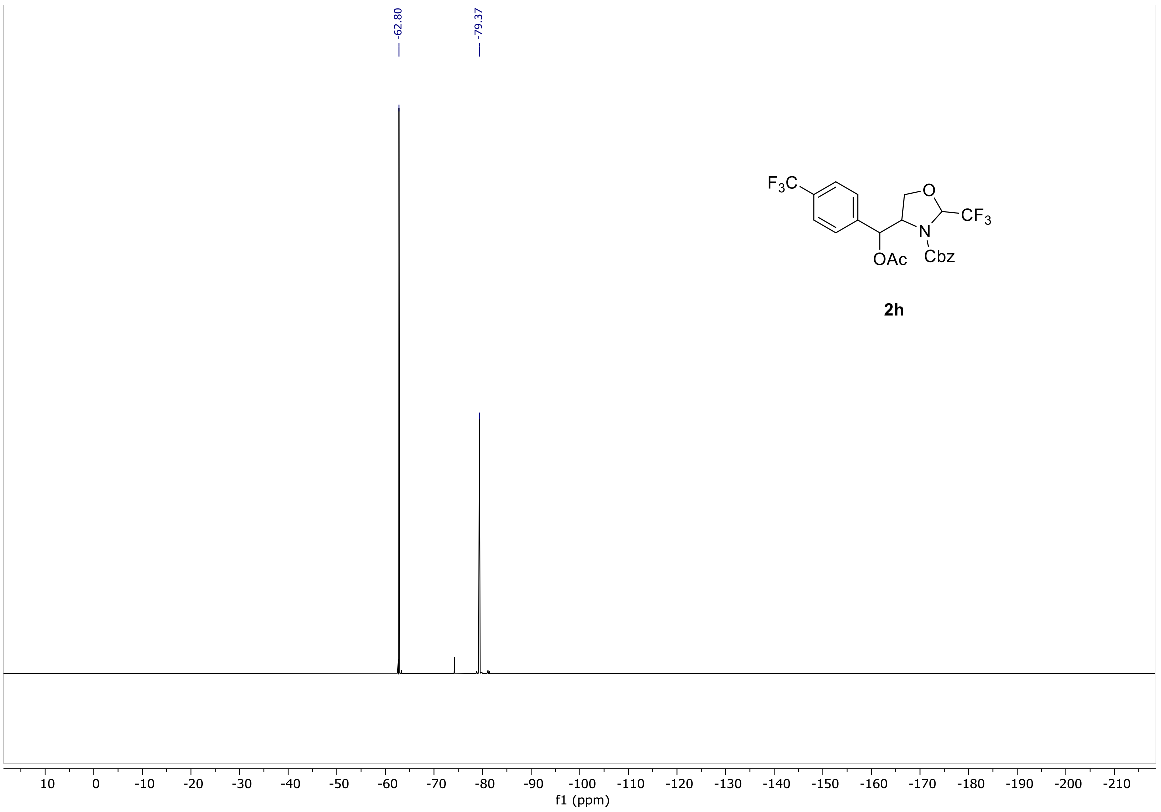

^{1}H NMR (400 MHz, $CDCl_3$)

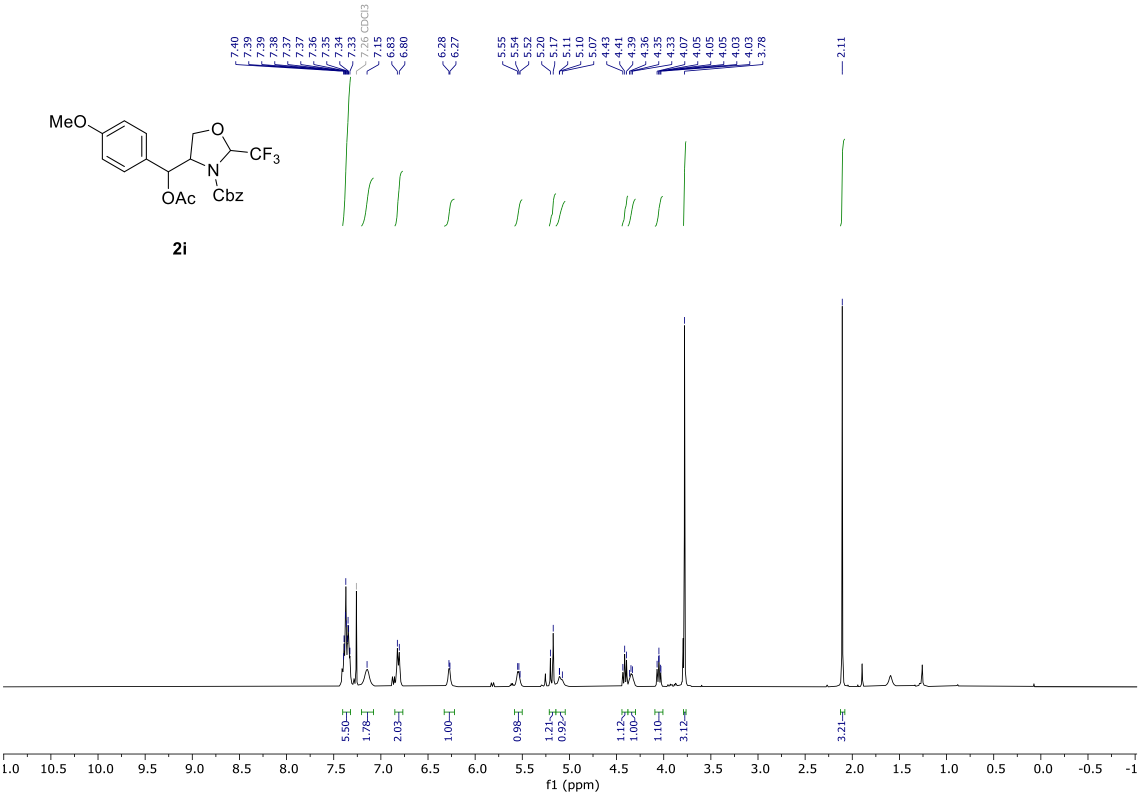

^{13}C NMR (101 MHz, $CDCl_3$)

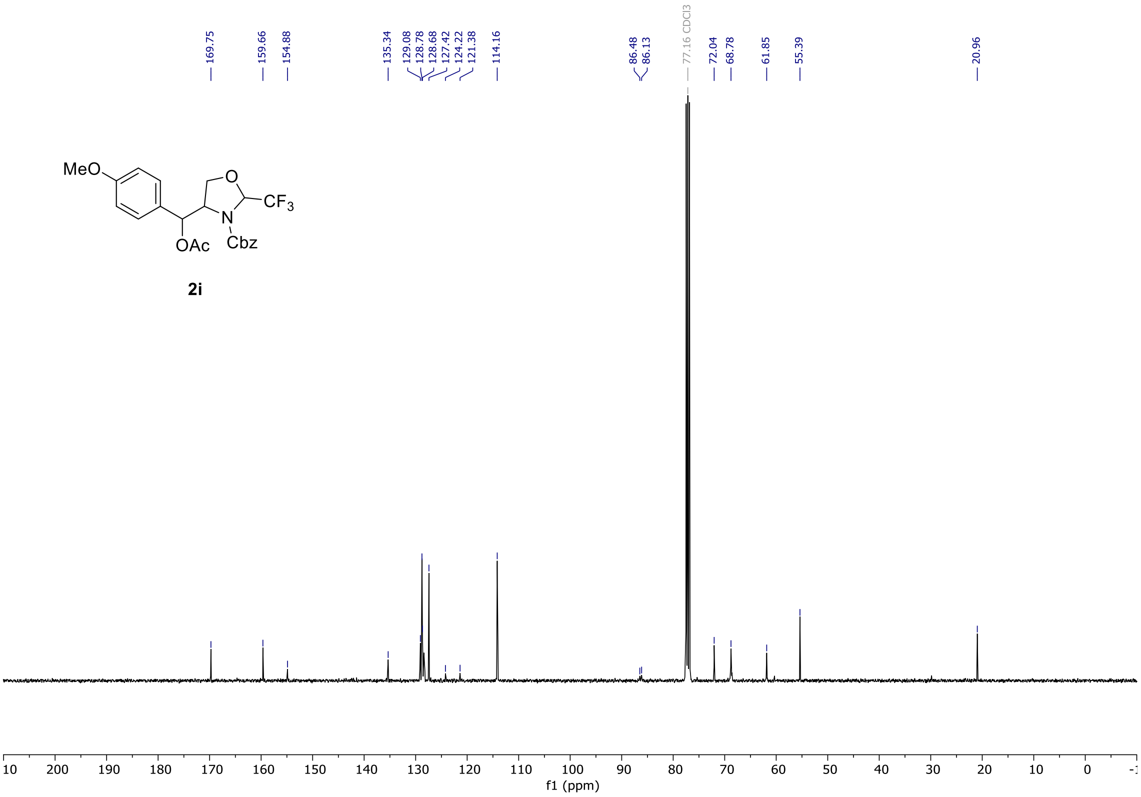

^{19}F NMR (376 MHz, $CDCl_3$)

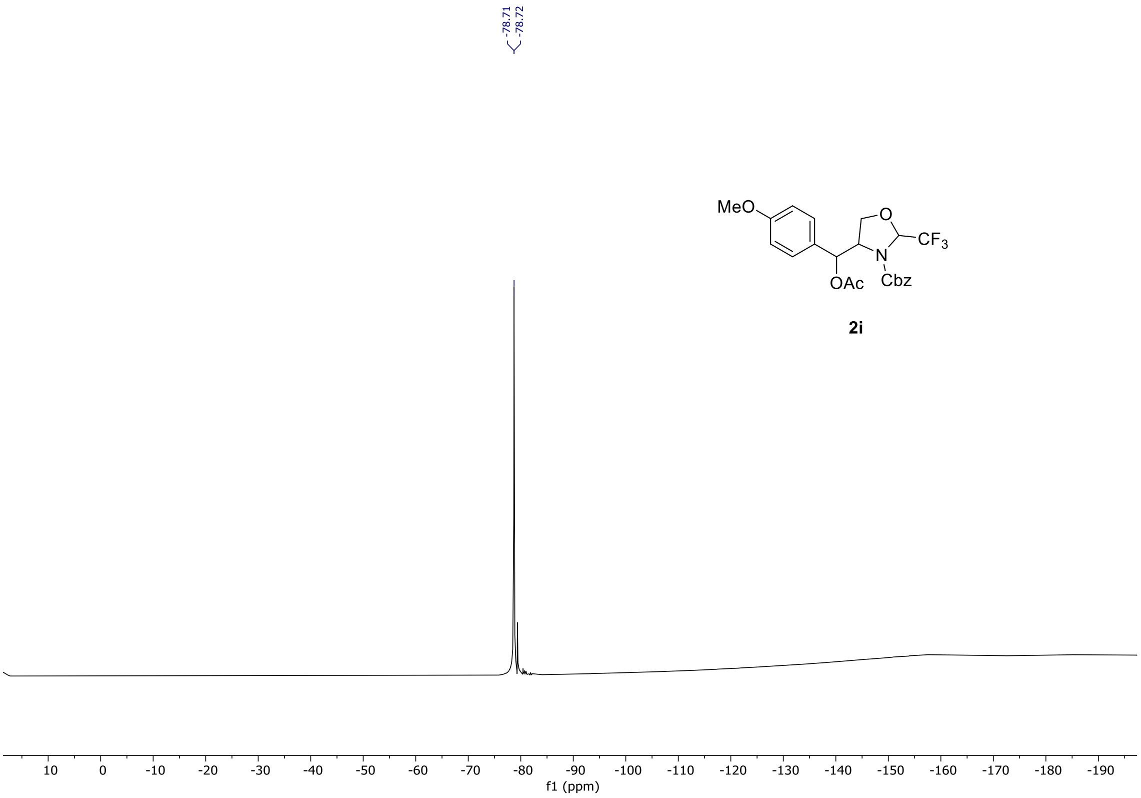

^{1}H NMR (400 MHz, $CDCl_3$)

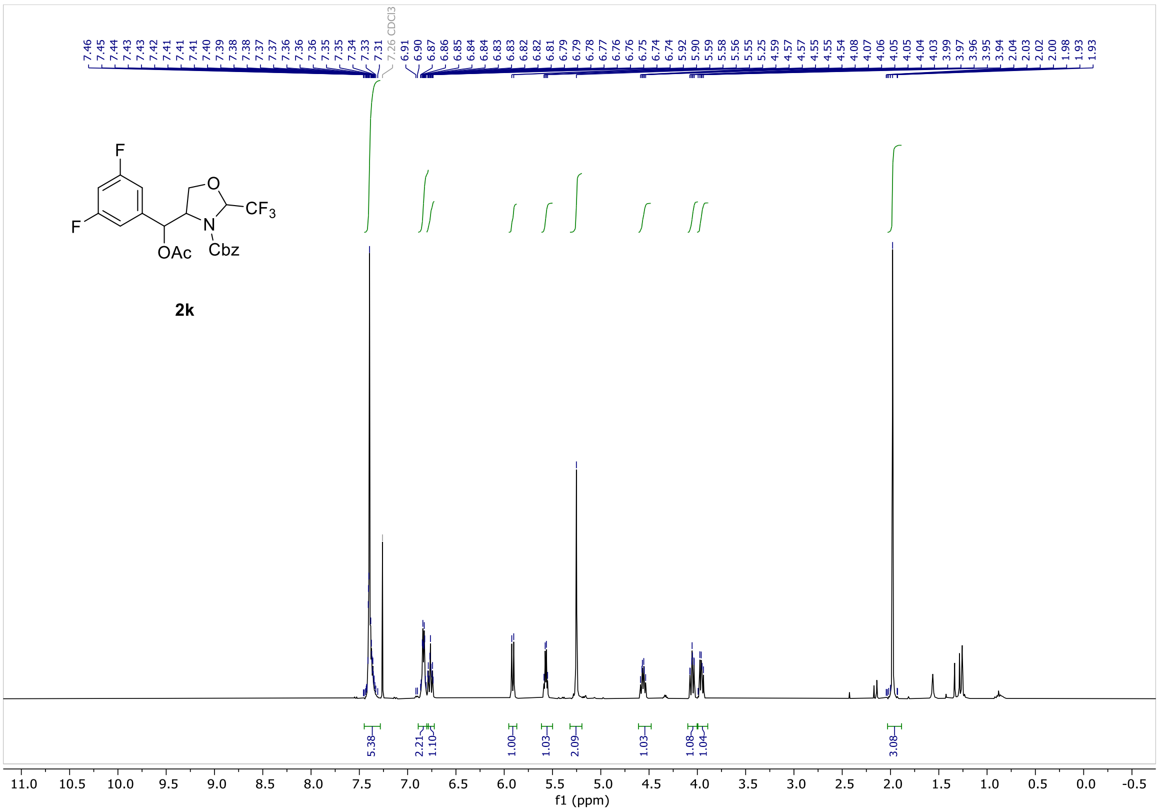

^{13}C NMR (101 MHz, $CDCl_3$)

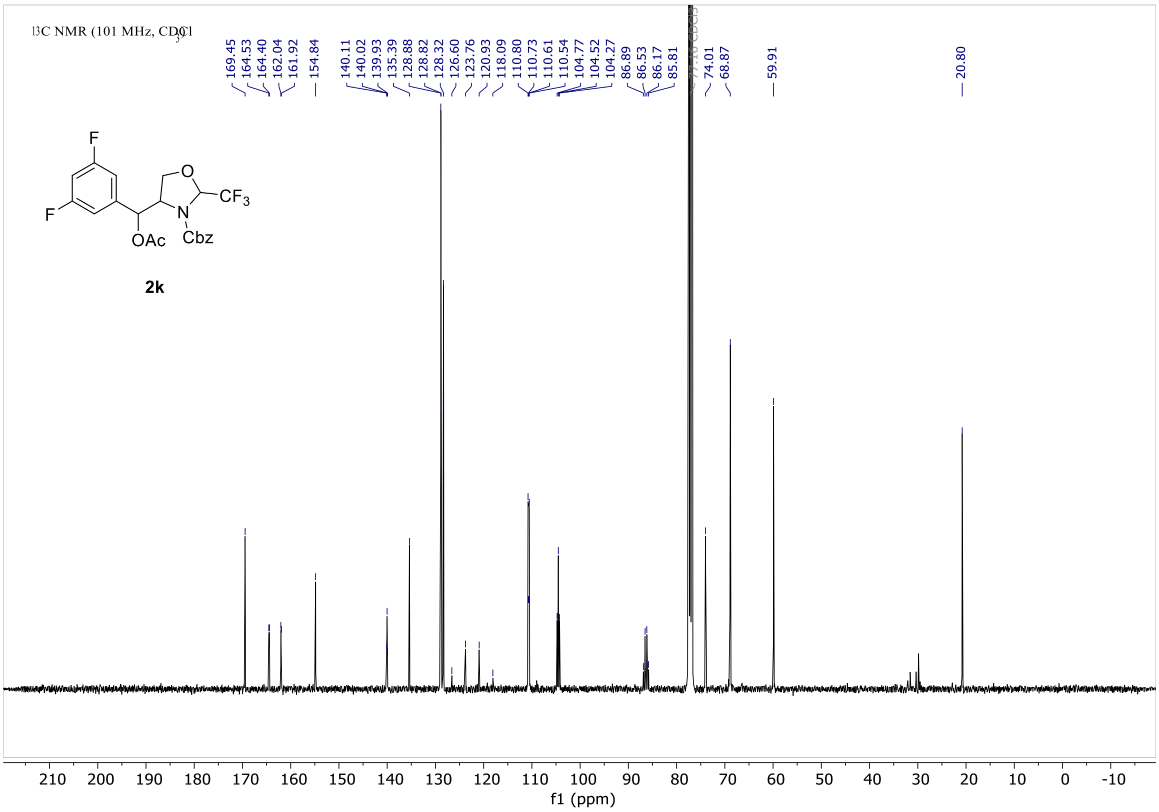

^{19}F NMR (376 MHz, $CDCl_3$)

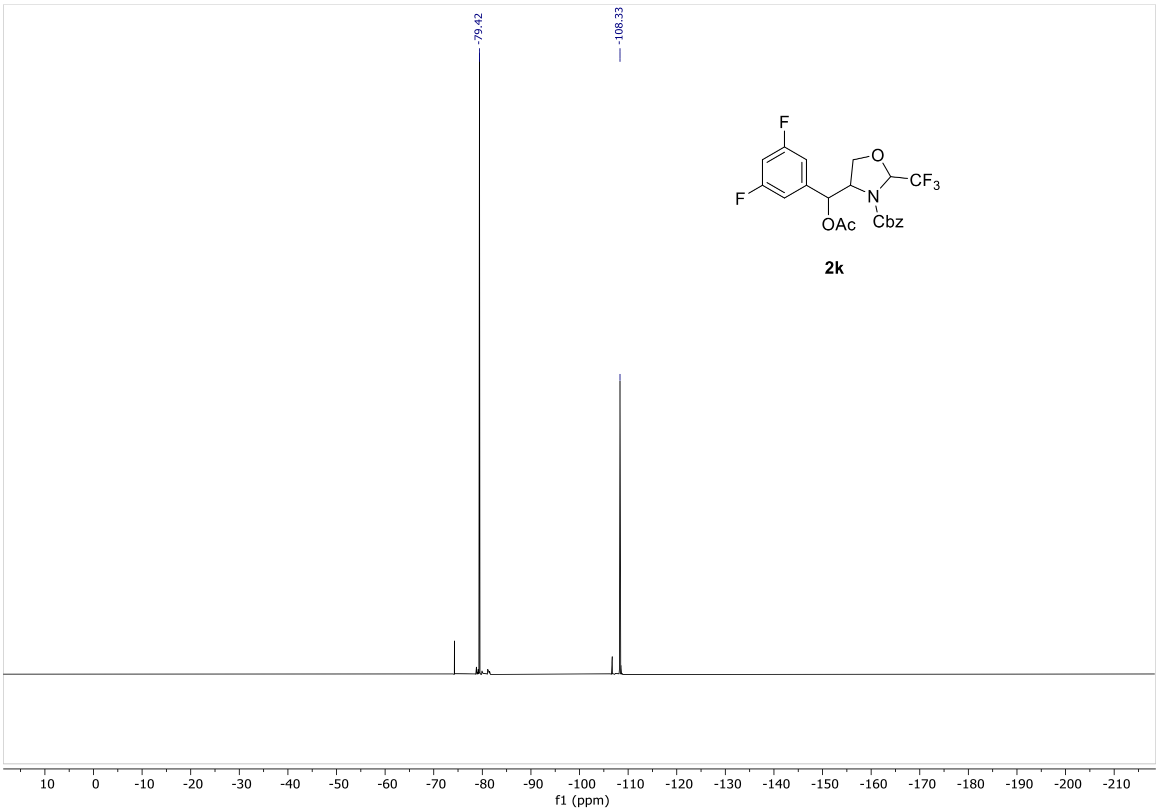

2k

^{1}H NMR (400 MHz, $CDCl_3$)

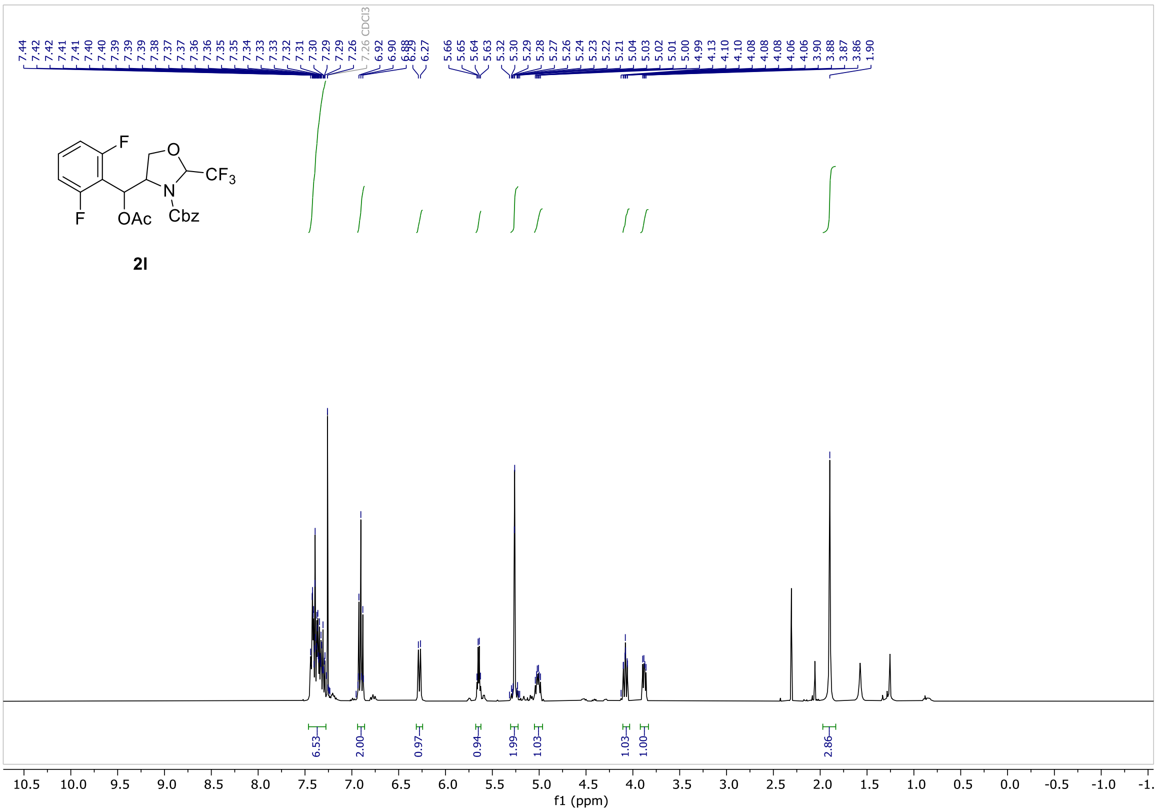

^{13}C NMR (101 MHz, $CDCl_3$)

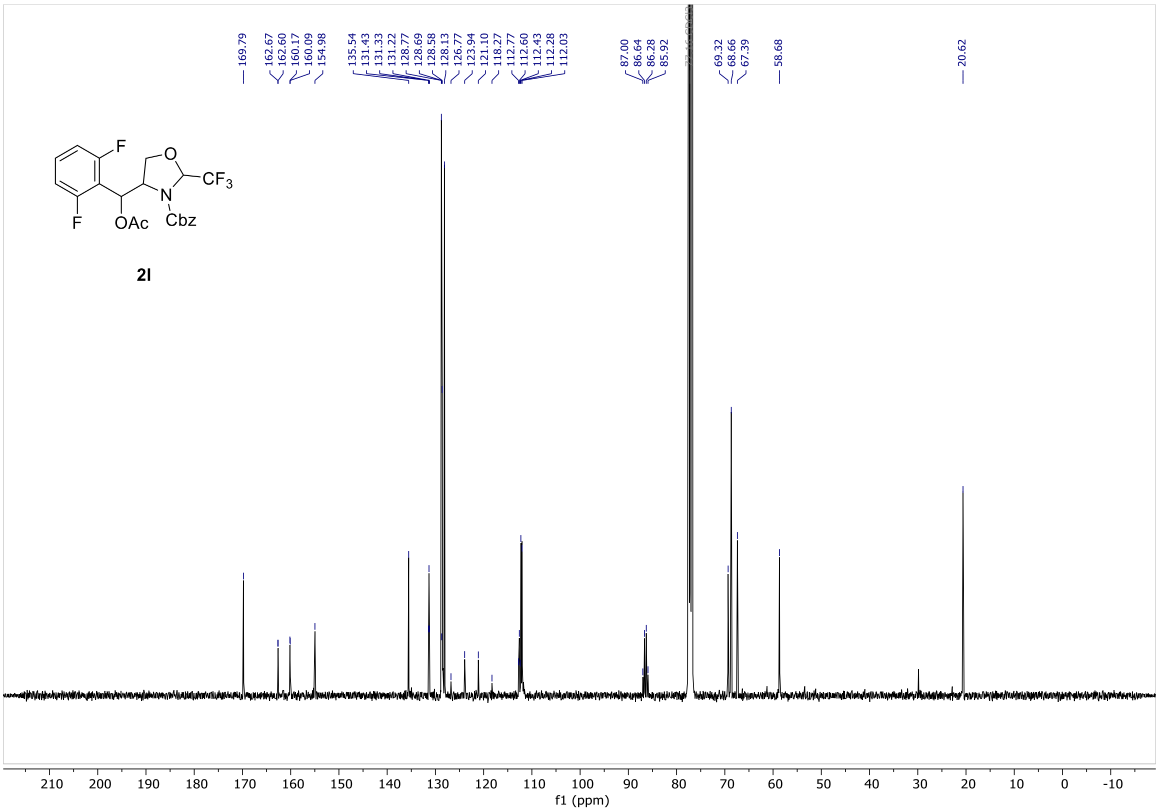

^{19}F NMR (376 MHz, $CDCl_3$)

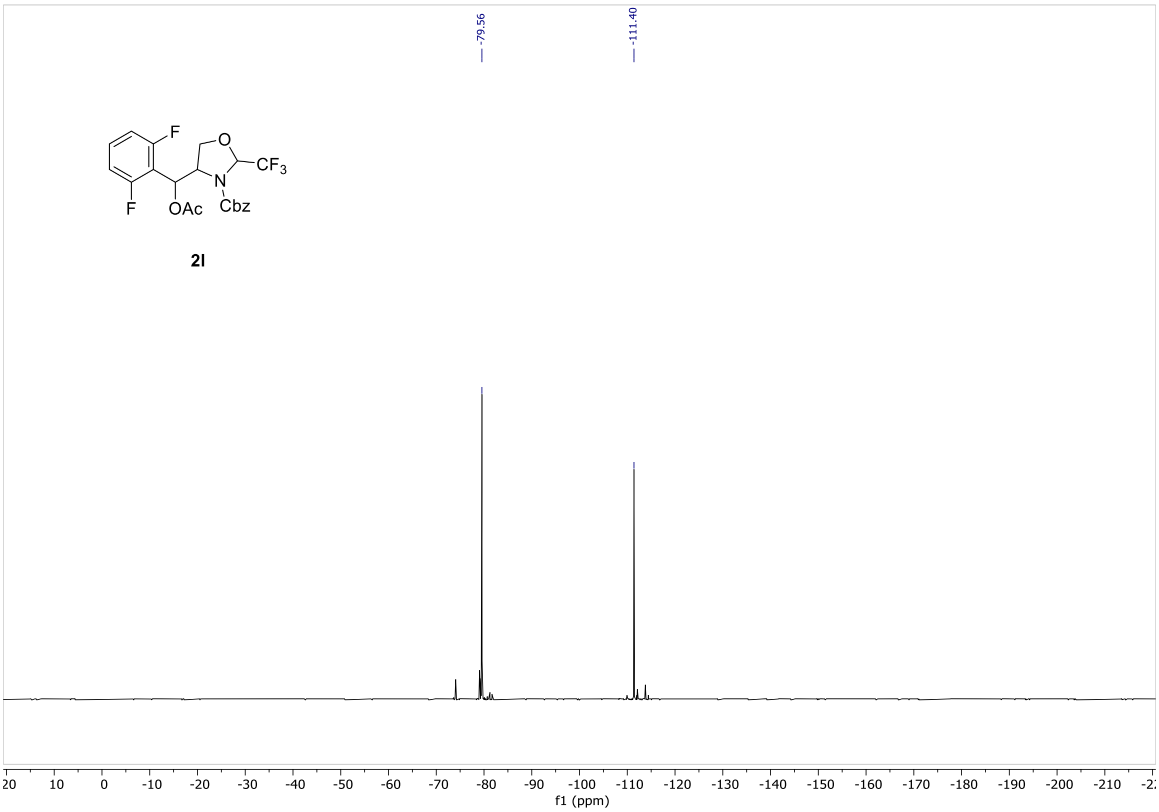

^{1}H NMR (400 MHz, $CDCl_3$)

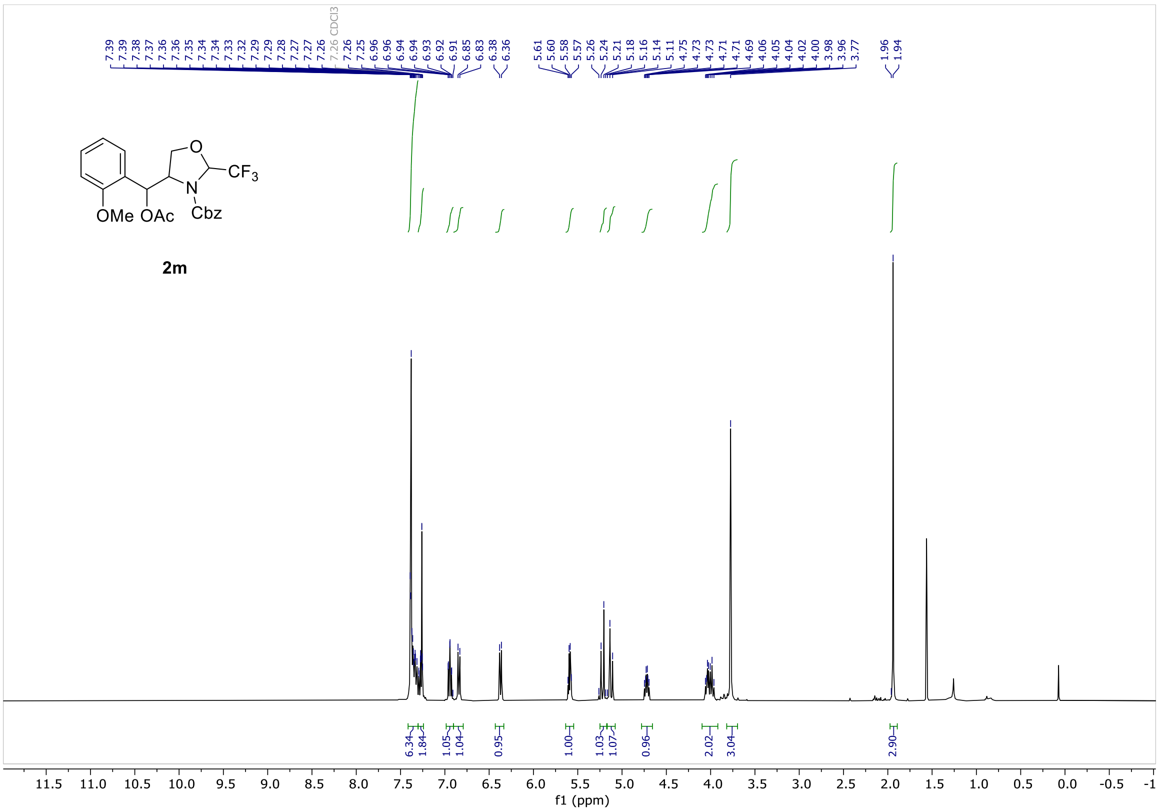

^{13}C NMR (101 MHz, $CDCl_3$)

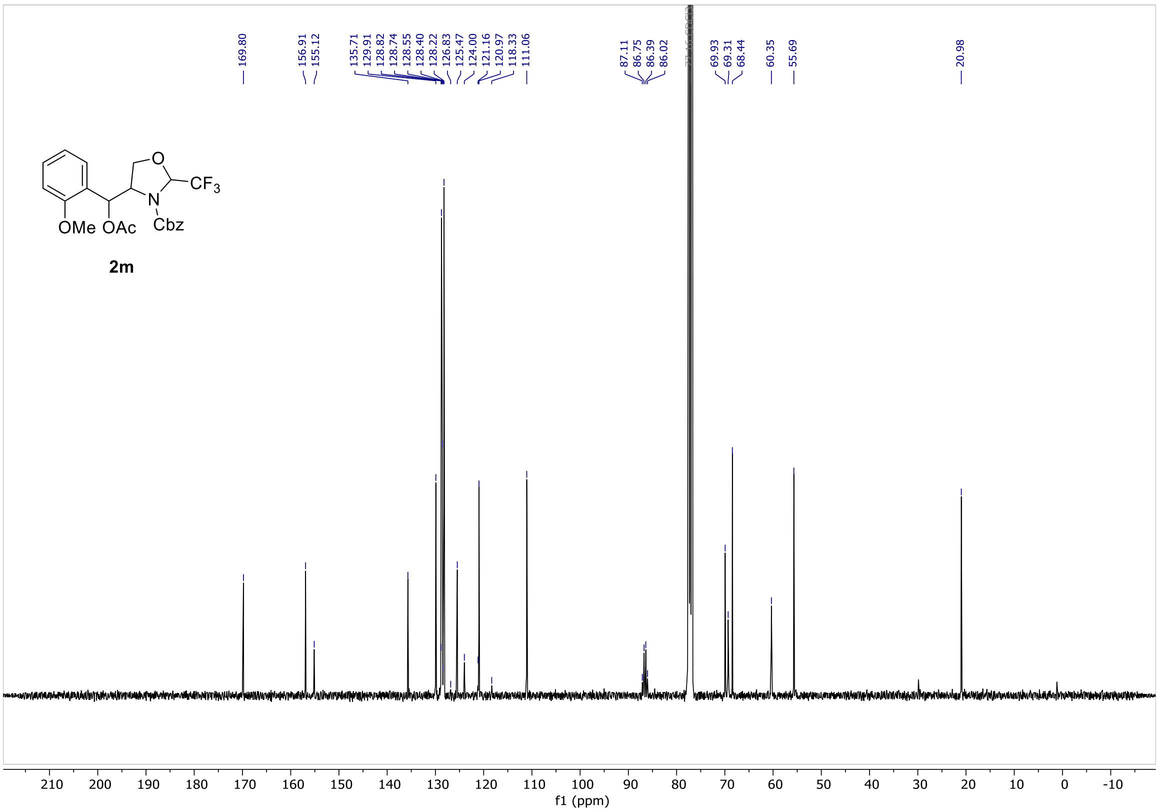

^{19}F NMR (376 MHz, $CDCl_3$)

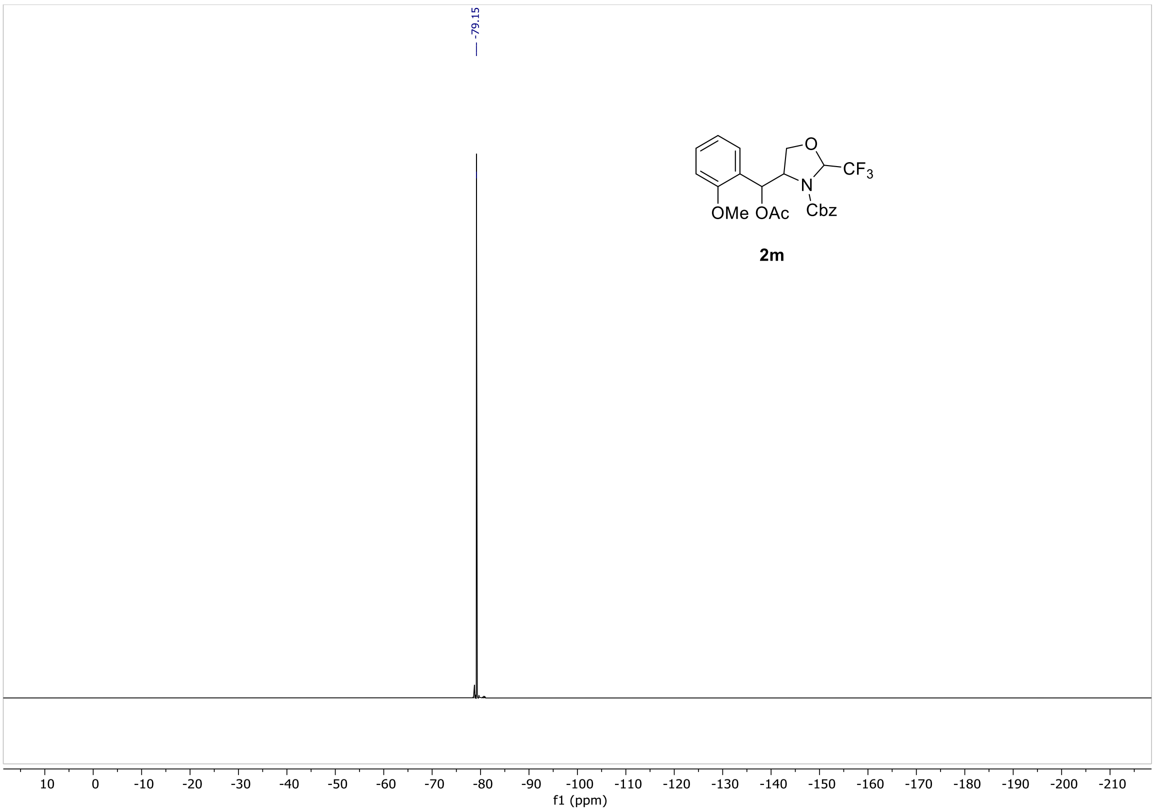

^{1}H NMR (400 MHz, $CDCl_3$)

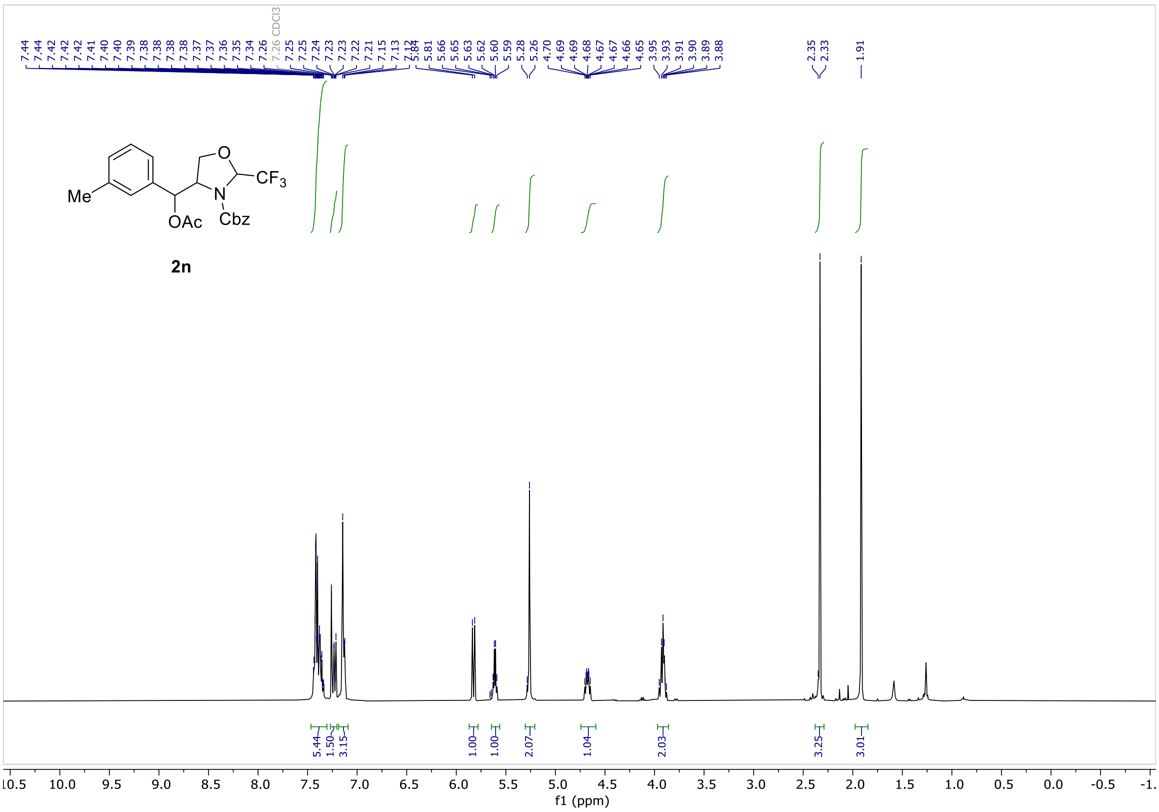

^{13}C NMR (101 MHz, $CDCl_3$)

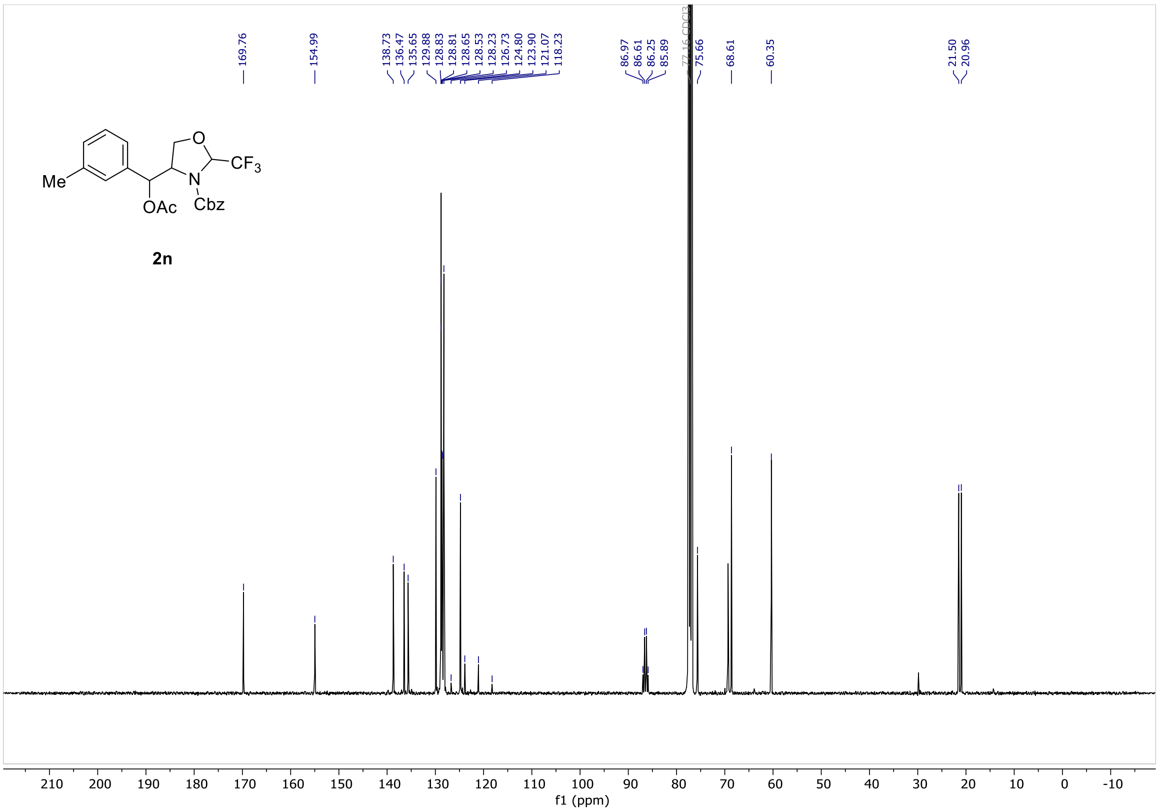

^{19}F NMR (376 MHz, $CDCl_3$)

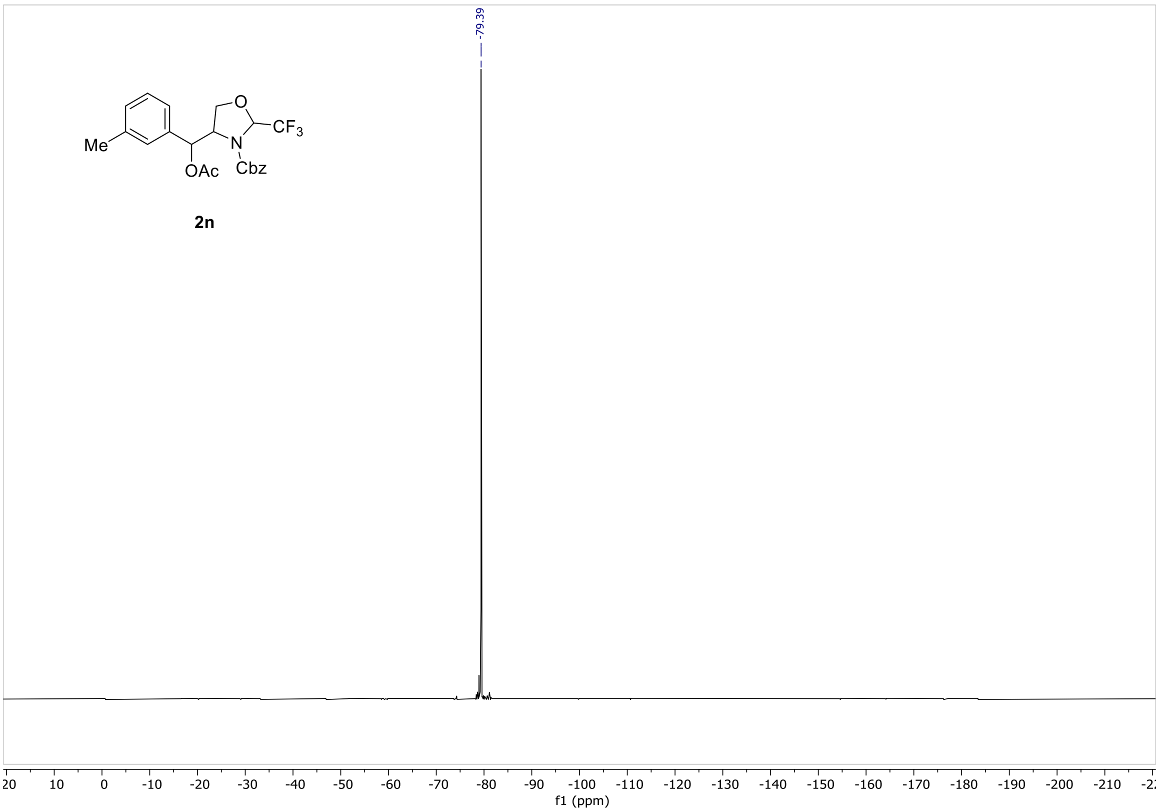

^{1}H NMR (400 MHz, CD_3CN)

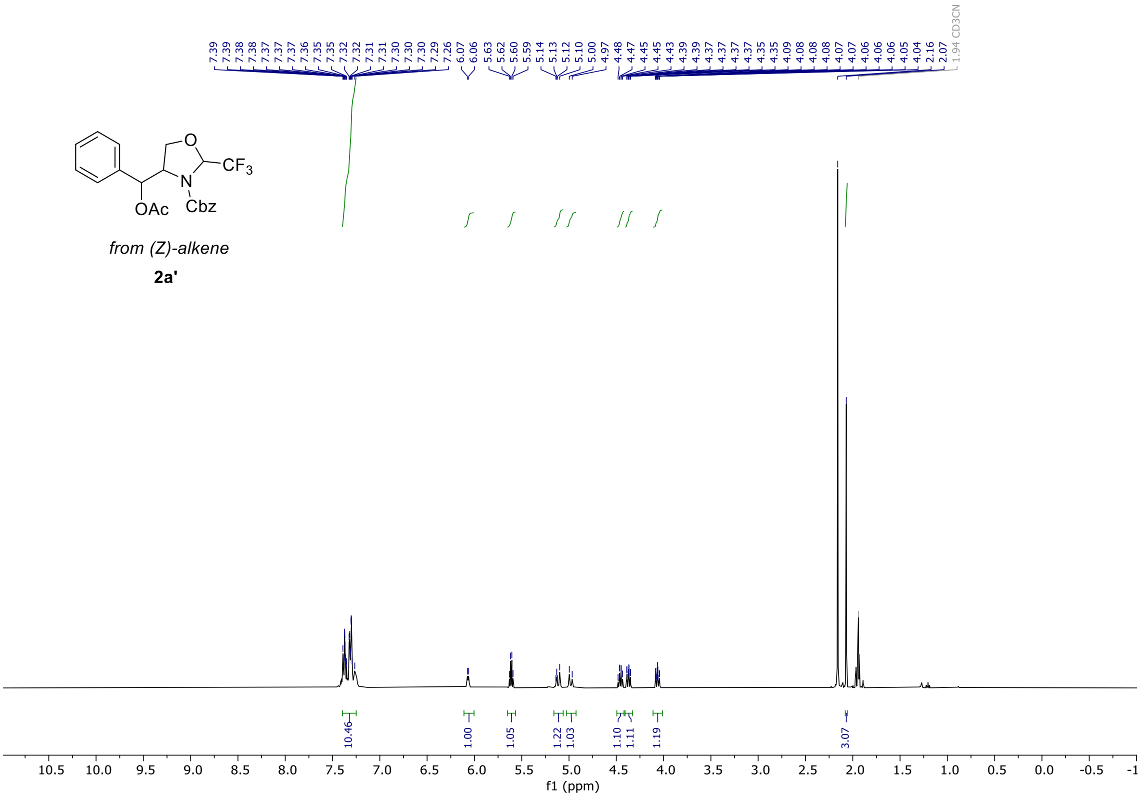

^{13}C NMR (101 MHz, CD_3CN)

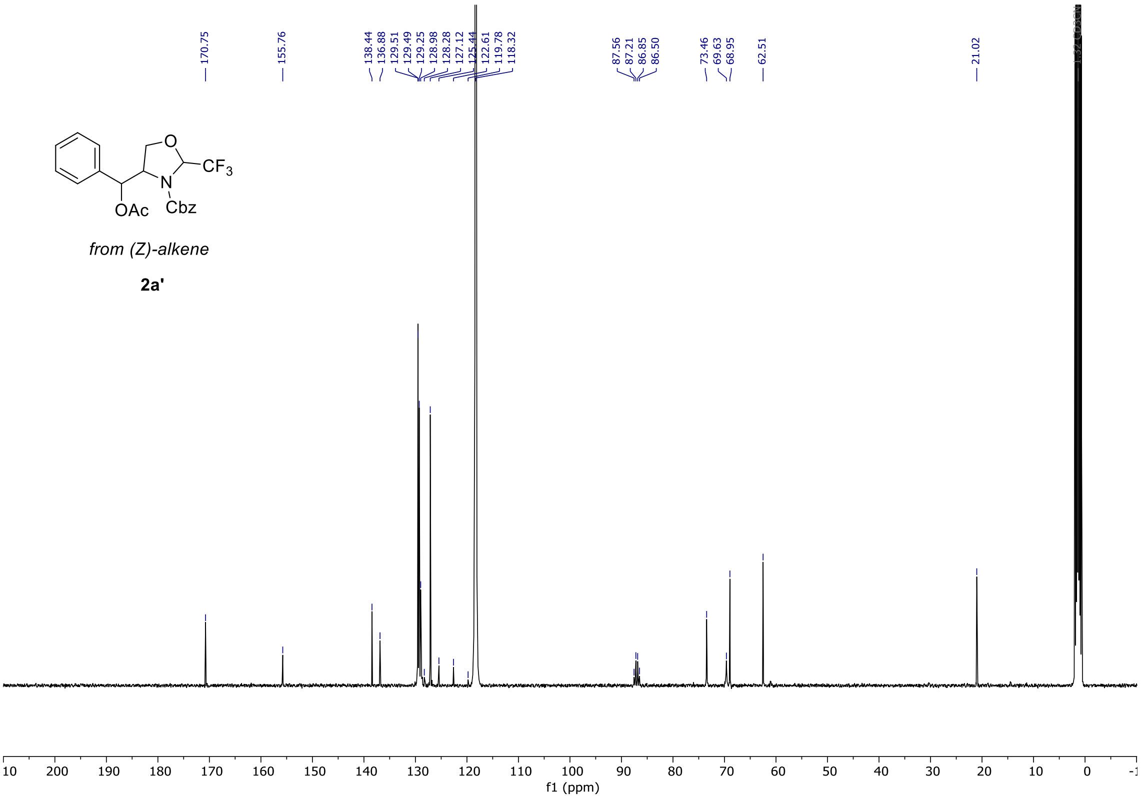

^{19}F NMR (376 MHz, CD_3CN)

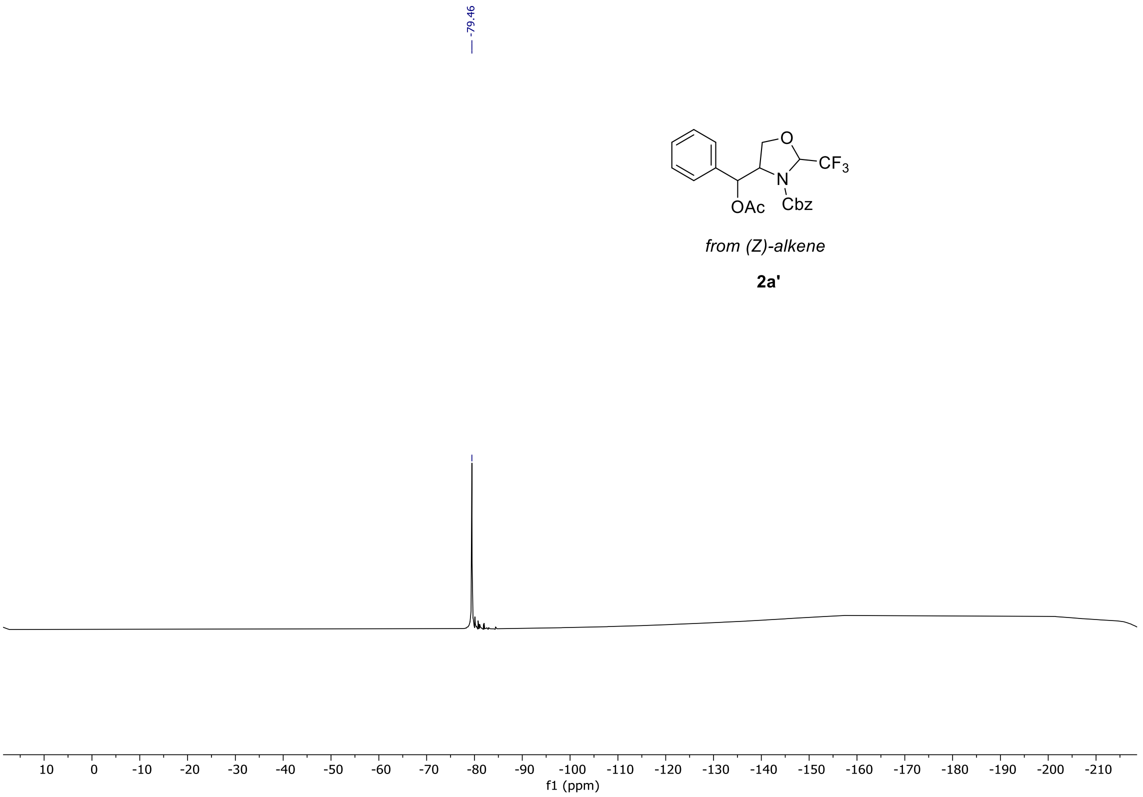

from (Z)-alkene

2a'

^{1}H NMR (400 MHz, CD_3CN)

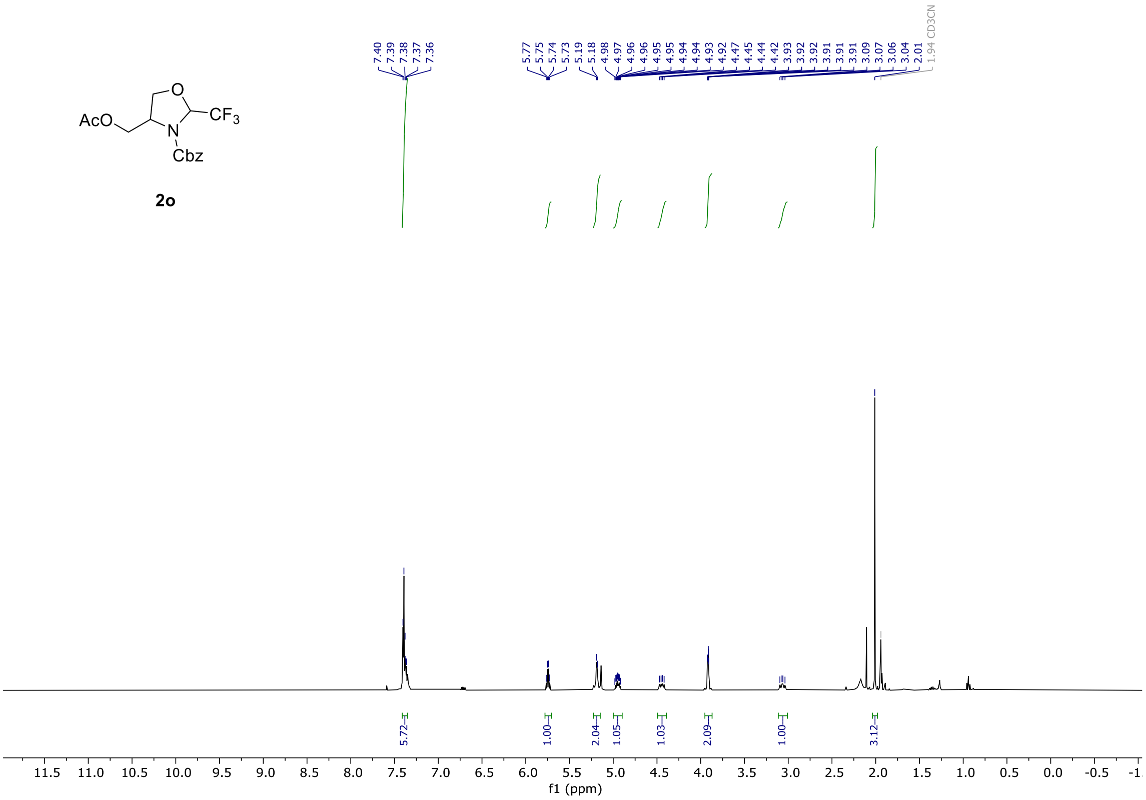

2o

^{13}C NMR (101 MHz, CD_3CN)

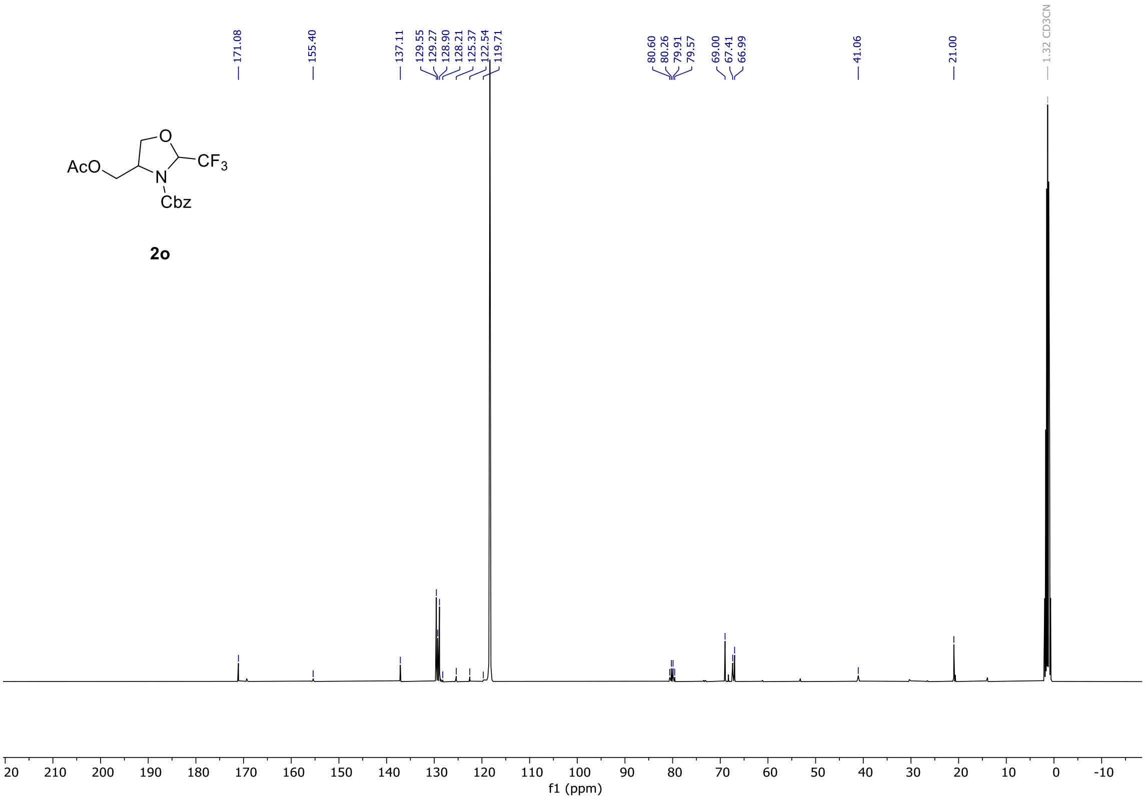

^{19}F NMR (376 MHz, CD_3CN)

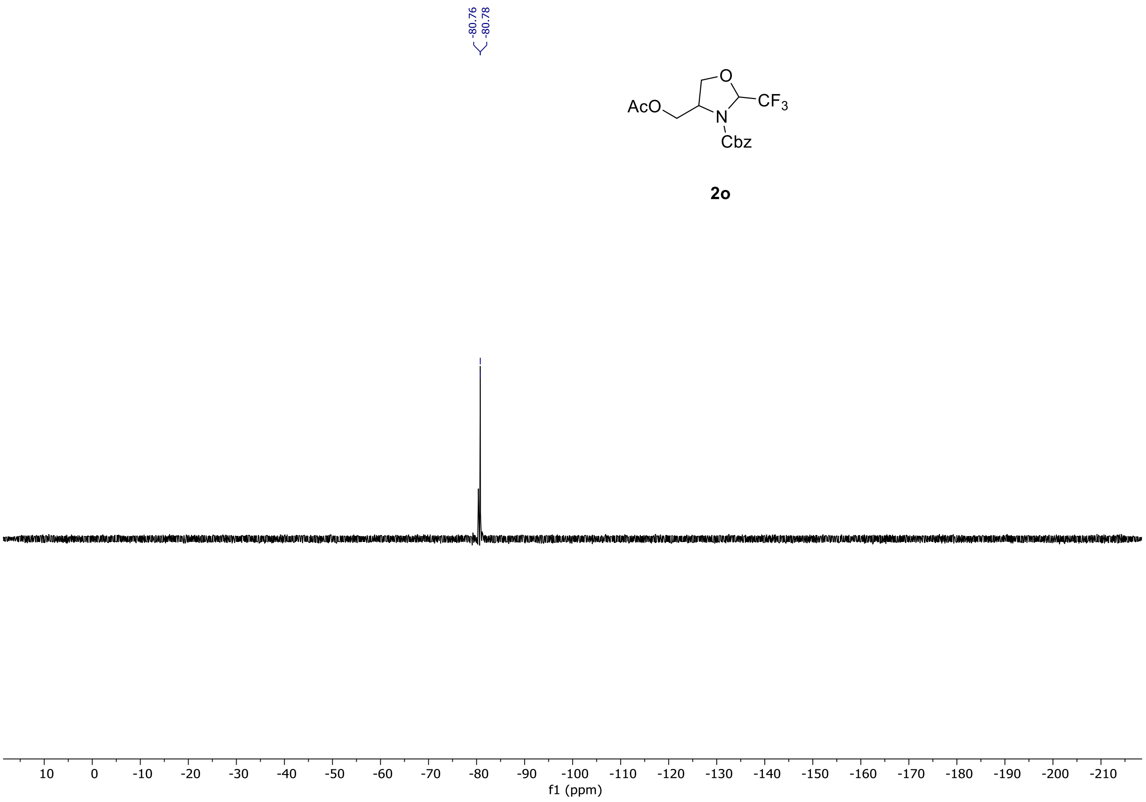

^1H NMR (400 MHz, CD_3CN)

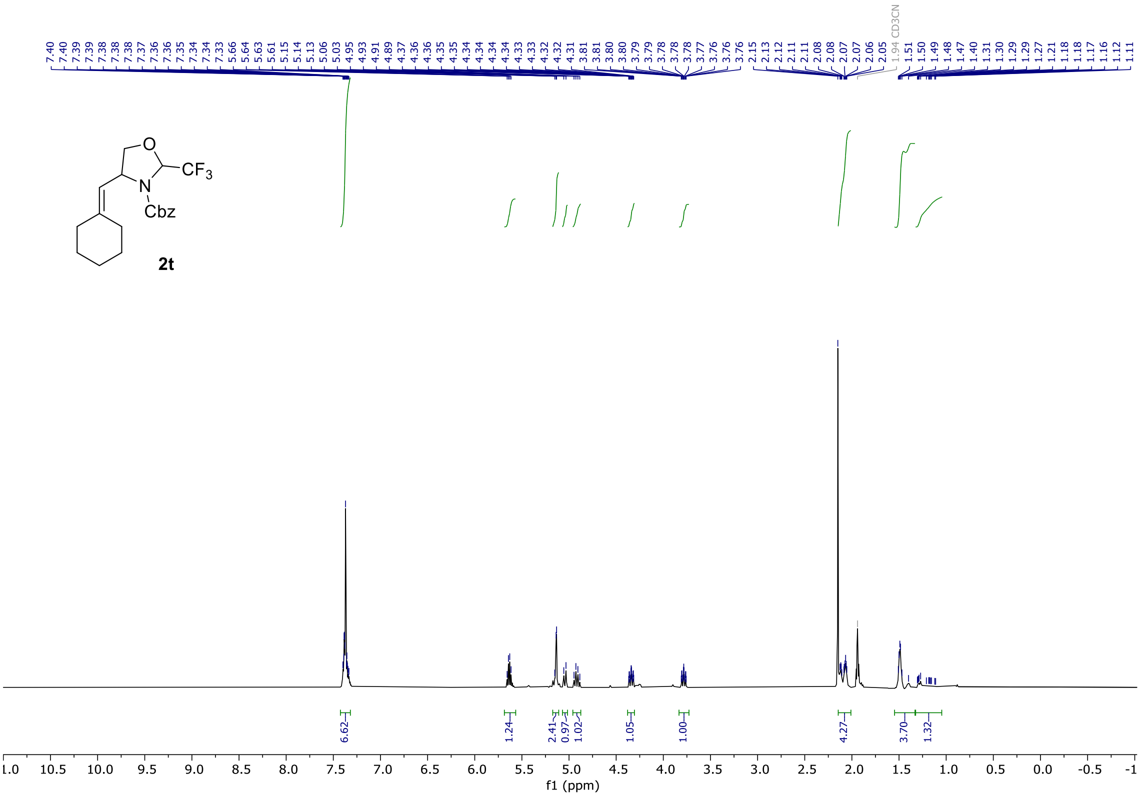

*traces of not completely resolved minor diastereoisomer visible, NMR data given for major isomer.

^{13}C NMR (101 MHz, CD_3CN)

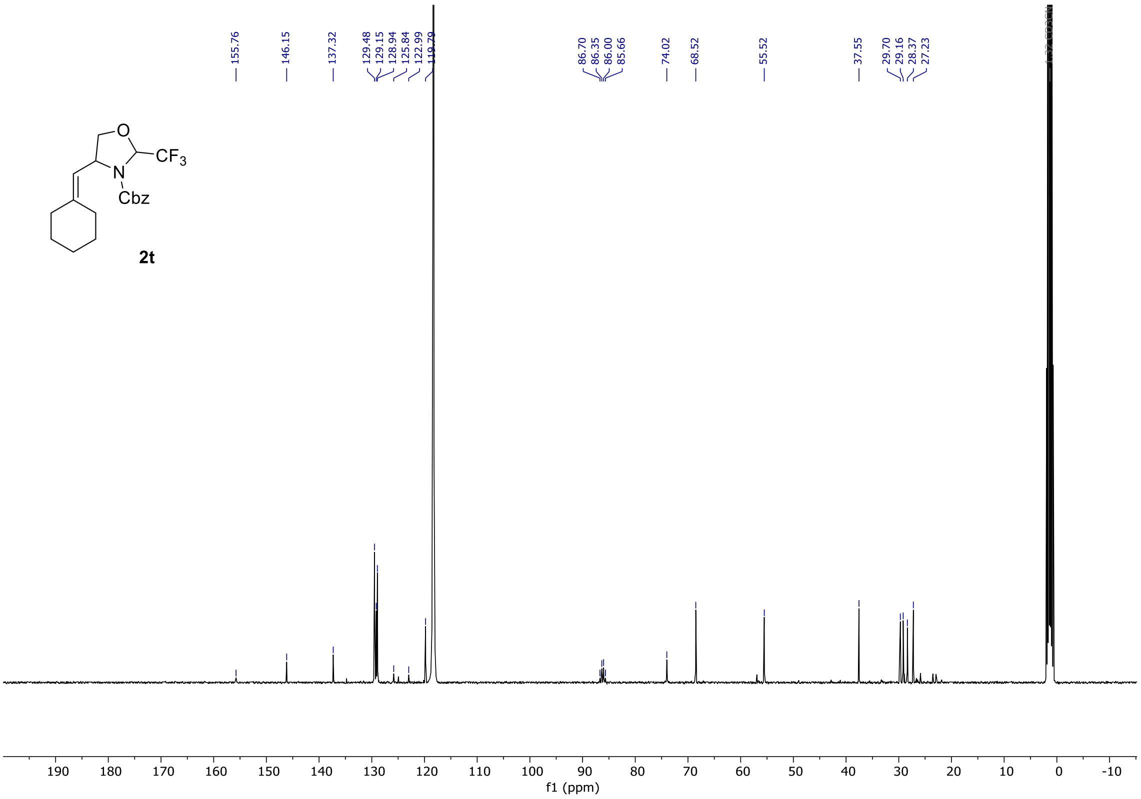

^{19}F NMR (376 MHz, CD_3CN)

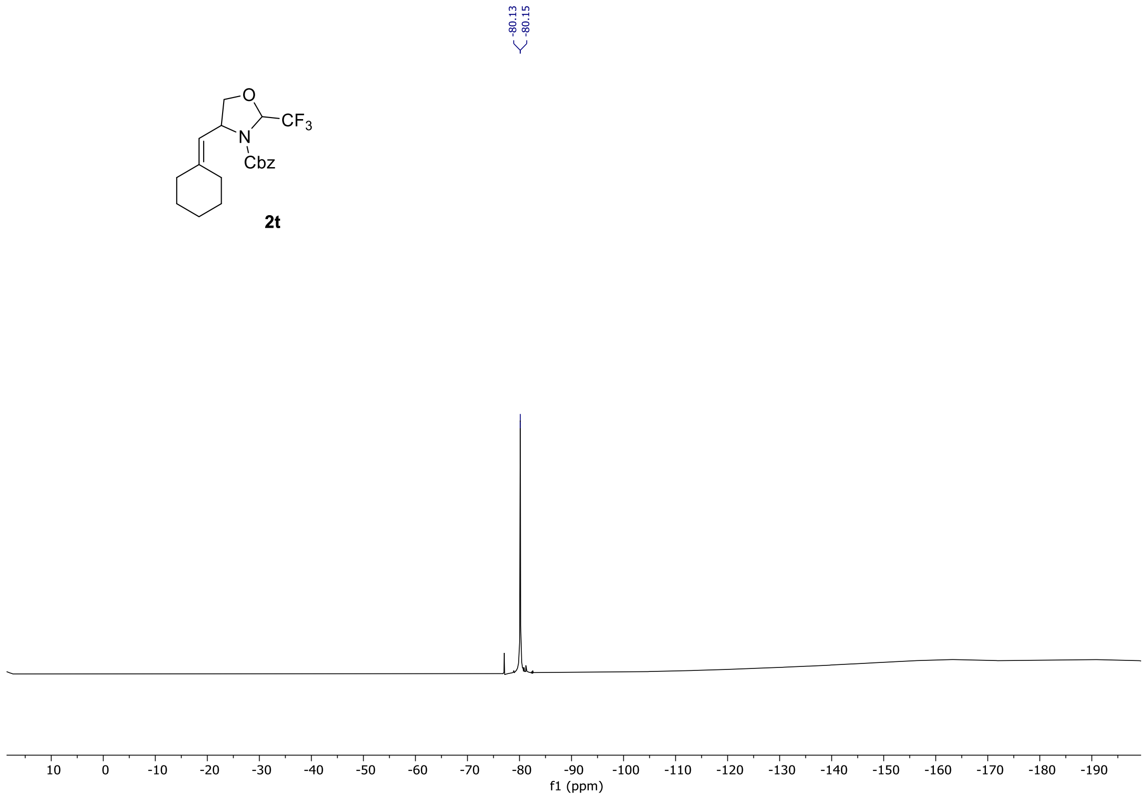

^{1}H NMR (400 MHz, $CDCl_3$)

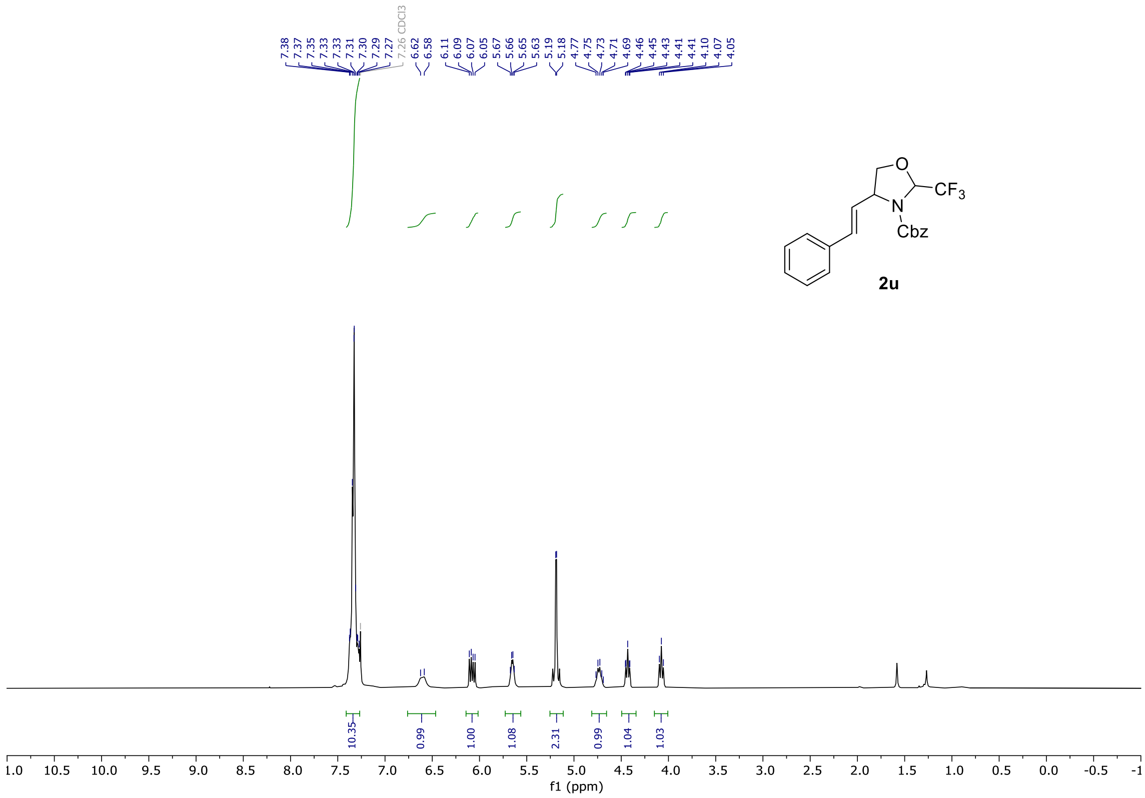

^{13}C NMR (101 MHz, $CDCl_3$)

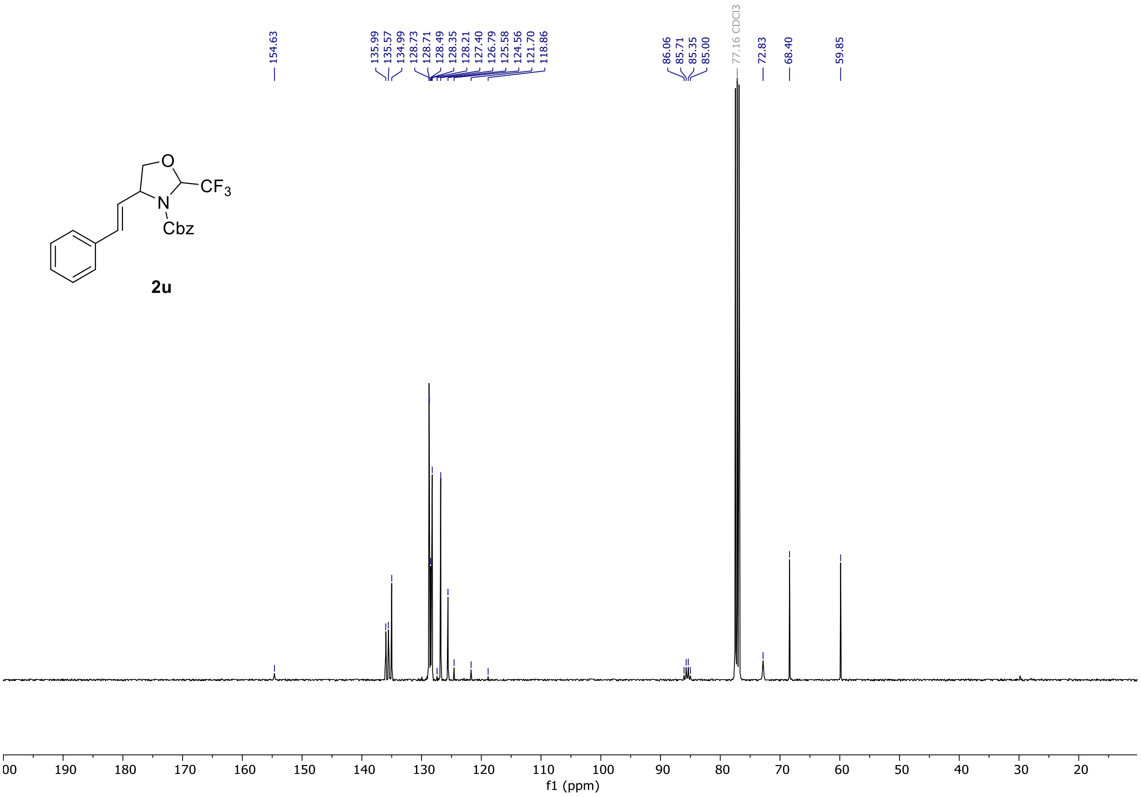

^{19}F NMR (376 MHz, $CDCl_3$)

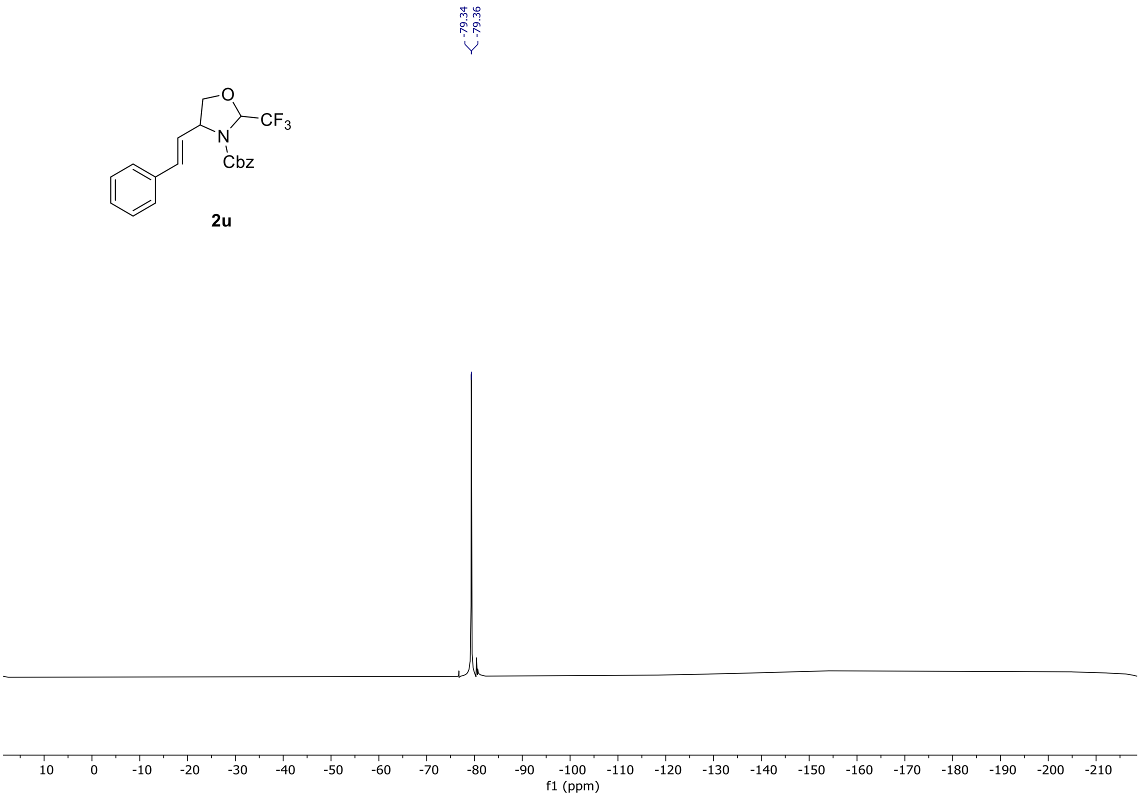

^{1}H NMR (400 MHz, CD_3CN)

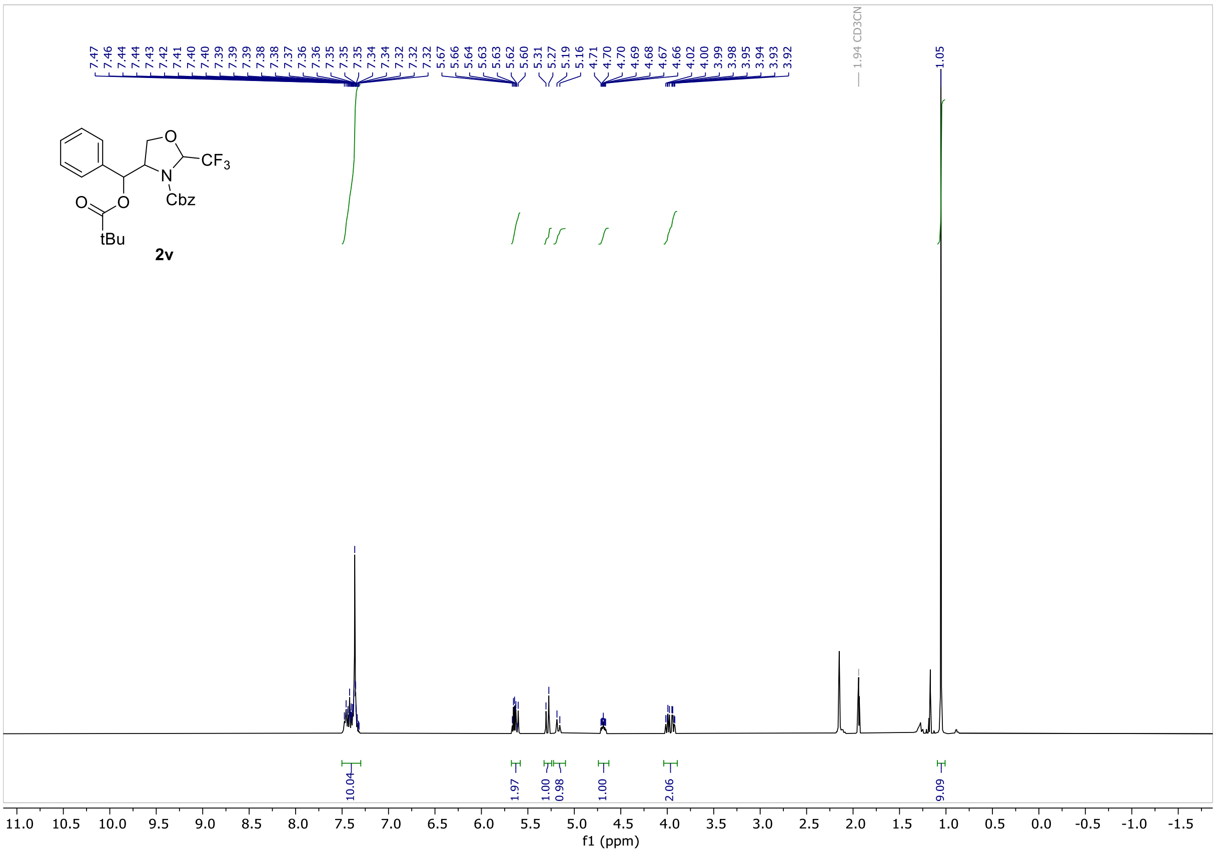

^{13}C NMR (101 MHz, CD_3CN)

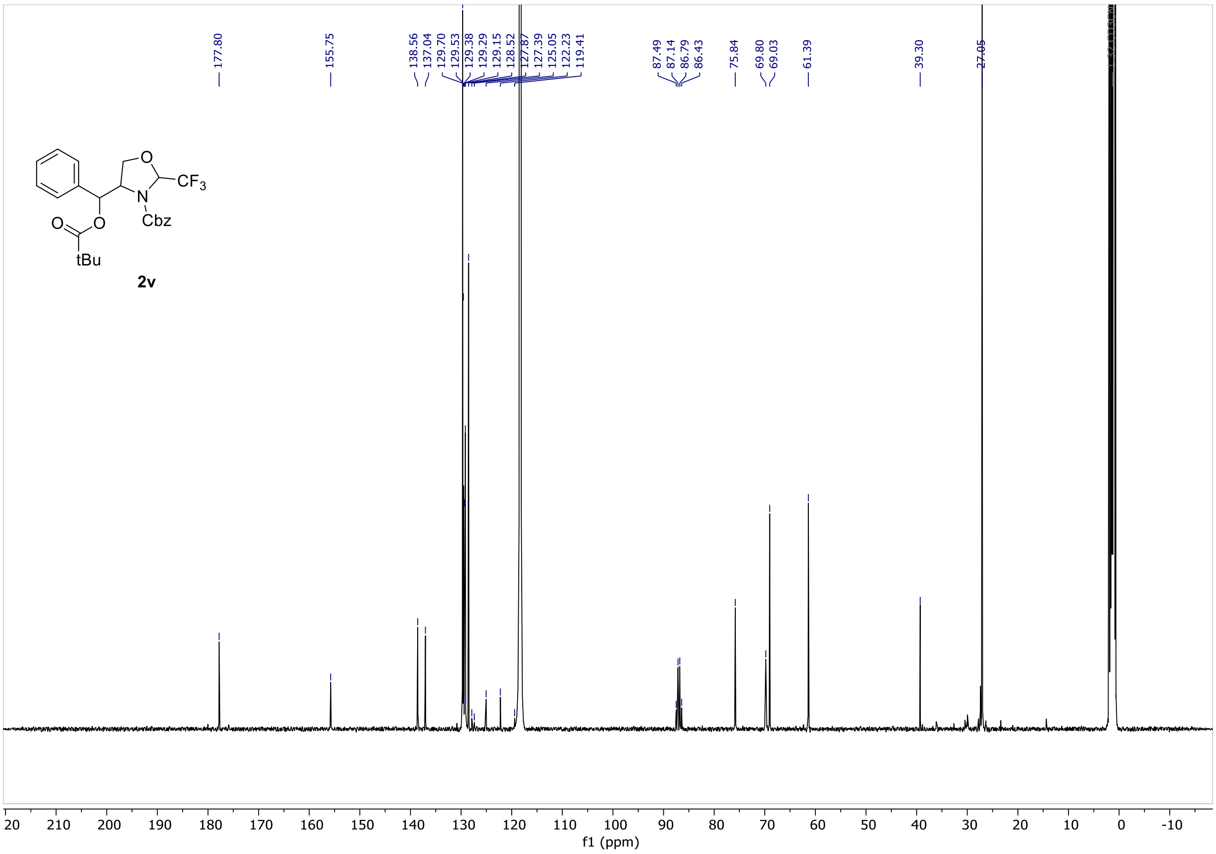

^{19}F NMR (376 MHz, CD_3CN)

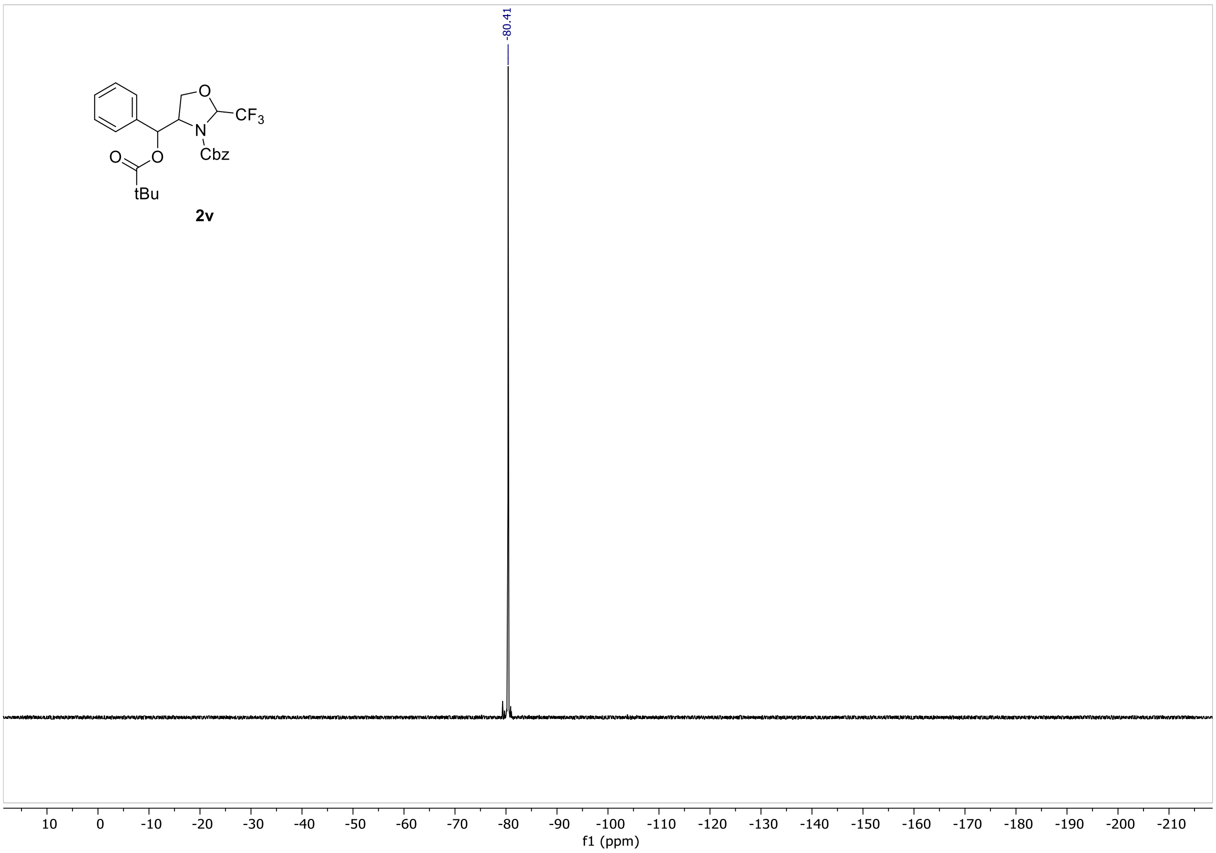

^{1}H NMR (400 MHz, CD_3CN)

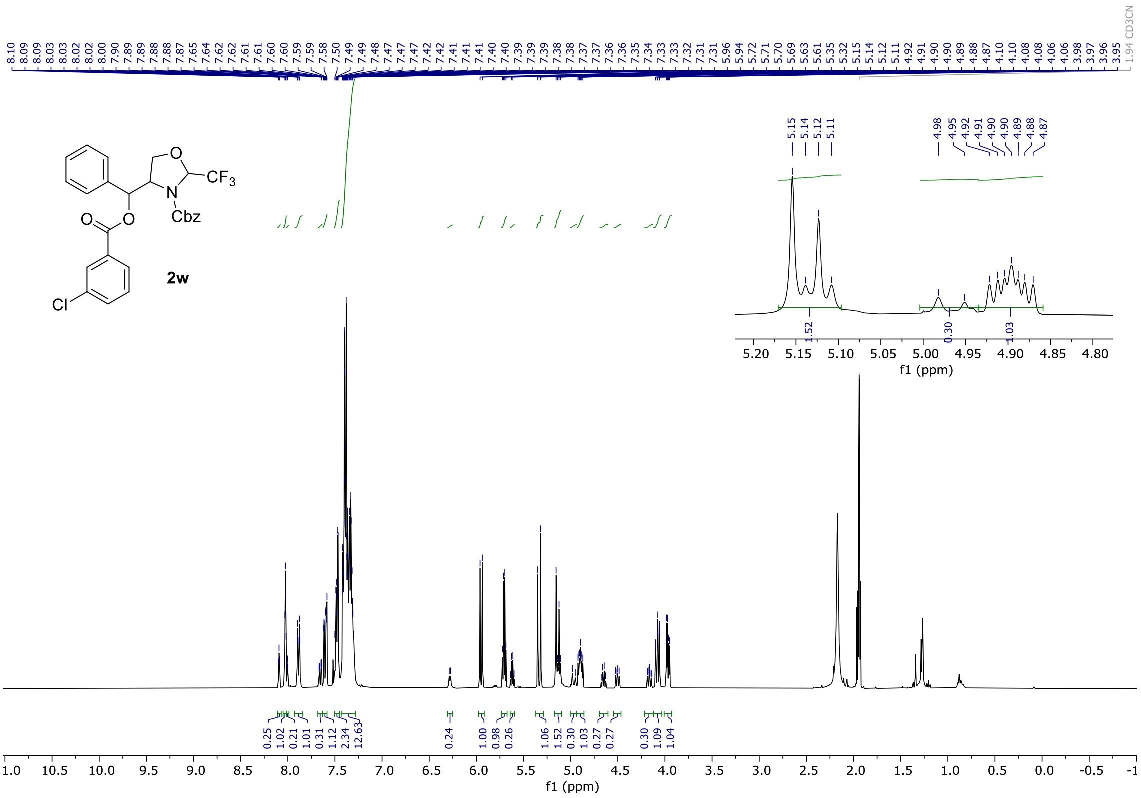

^{13}C NMR (101 MHz, CD_3CN)

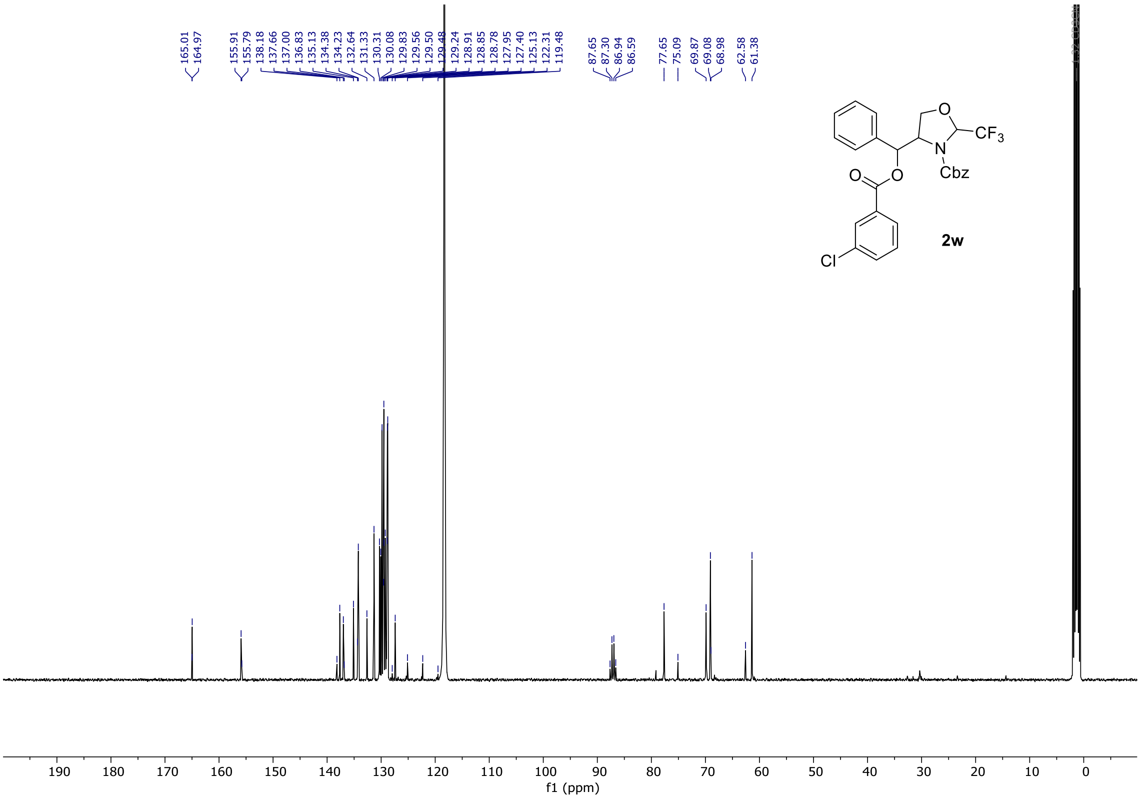

^{19}F NMR (376 MHz, CD_3CN)

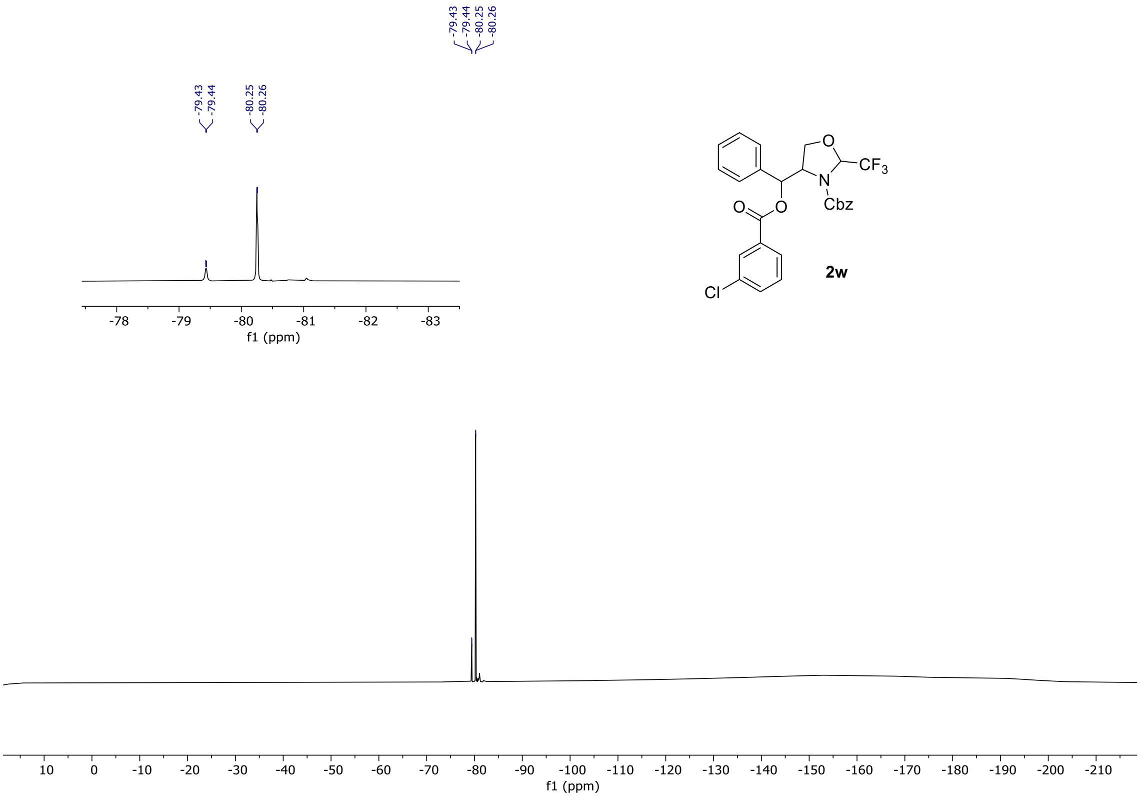

^{1}H NMR (400 MHz, $CDCl_3$)

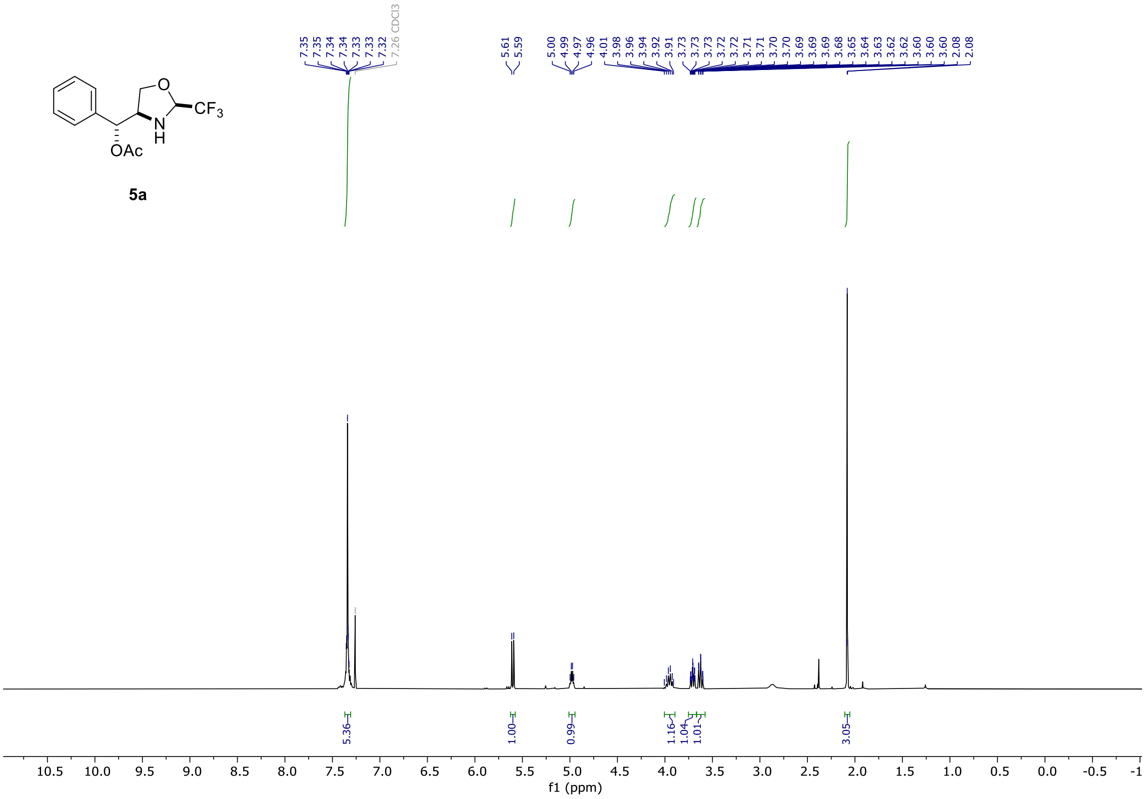

^{13}C NMR (101 MHz, $CDCl_3$)

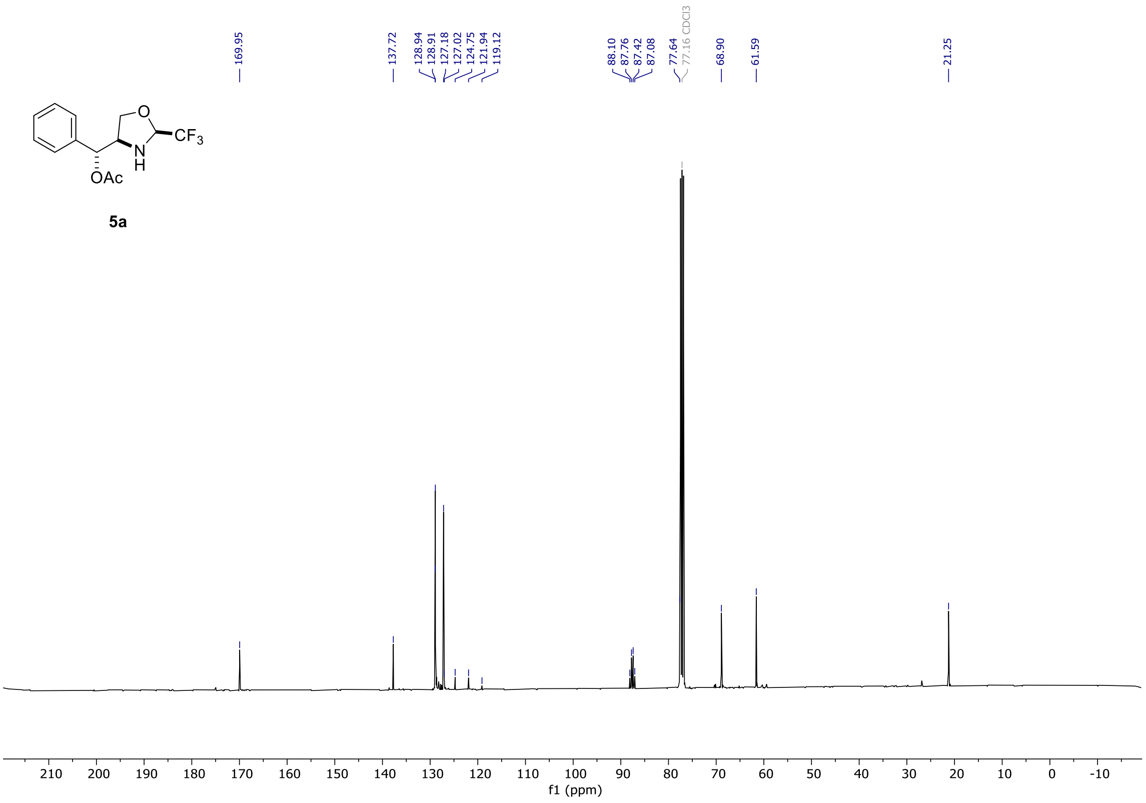

^{19}F NMR (376 MHz, $CDCl_3$)

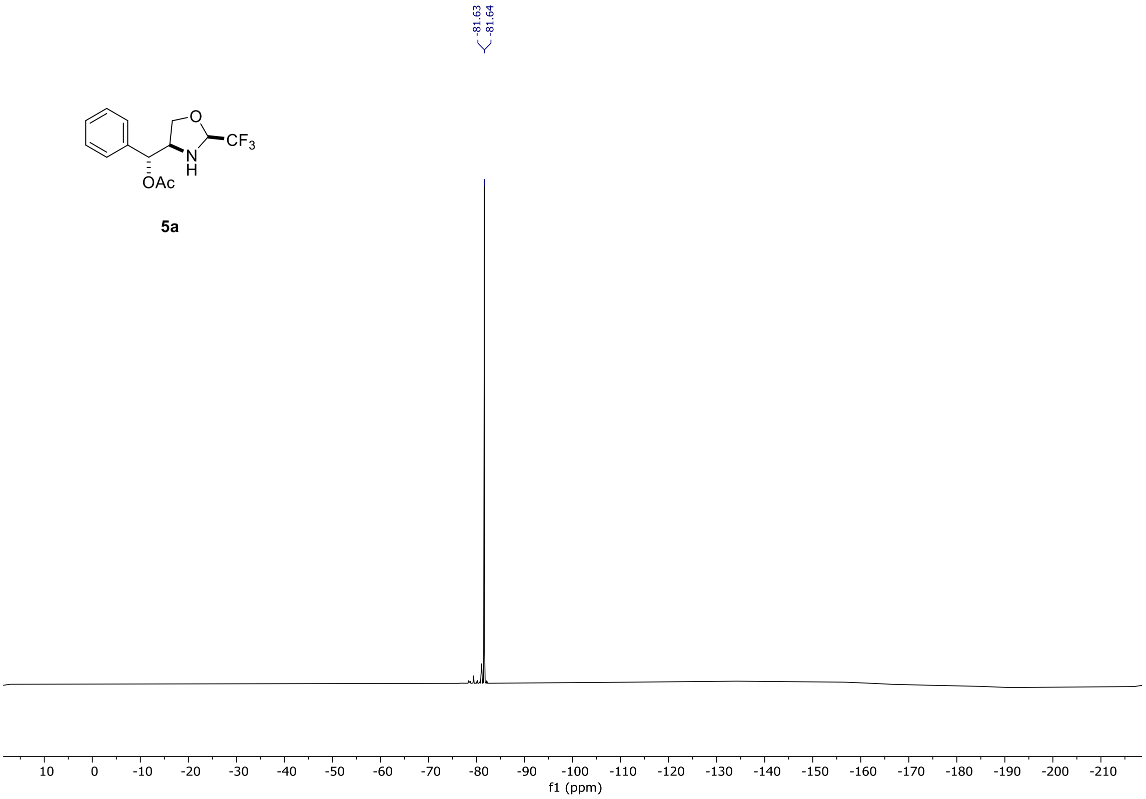

^{1}H NMR (400 MHz, MeOD)

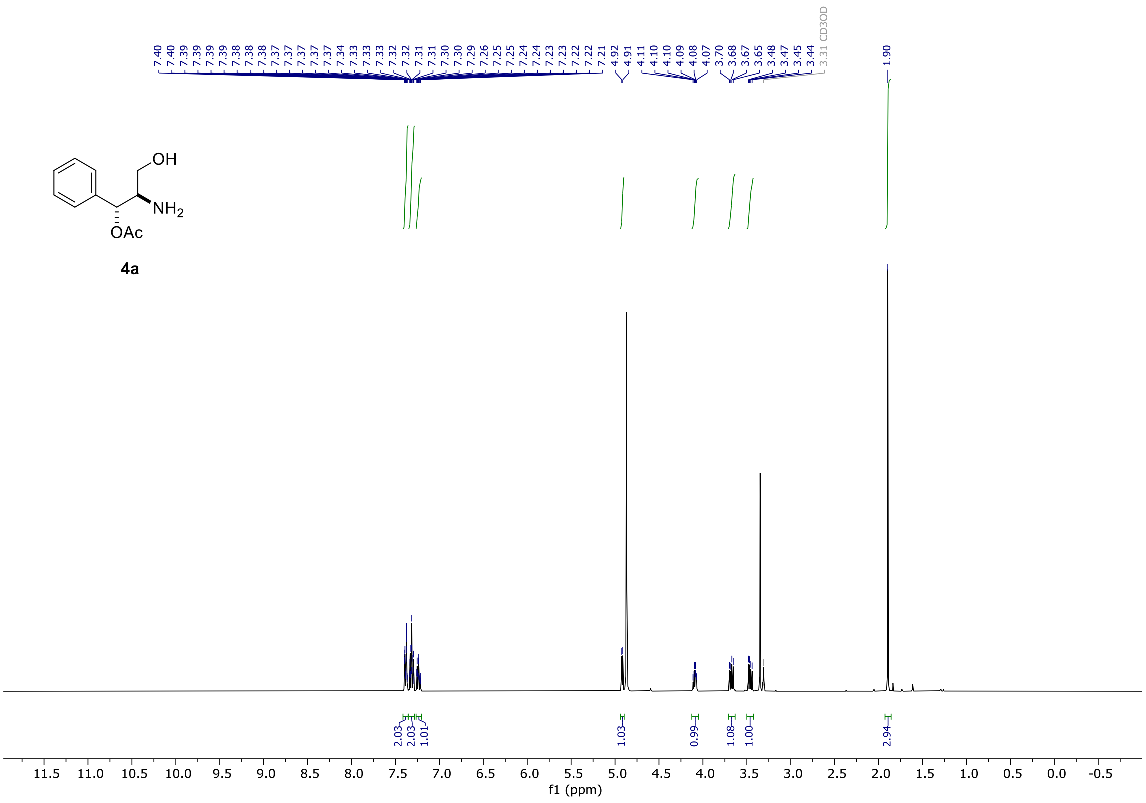

^{13}C NMR (101 MHz, MeOD)

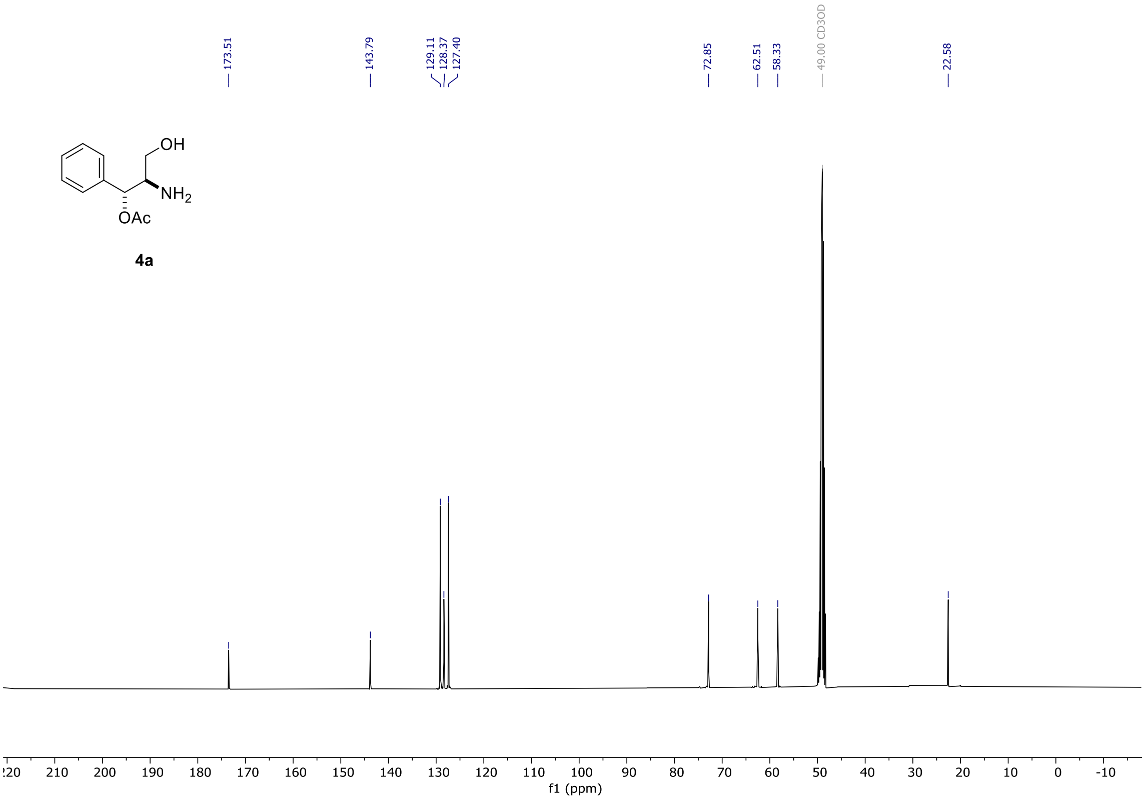

^{1}H NMR (400 MHz, MeOD)

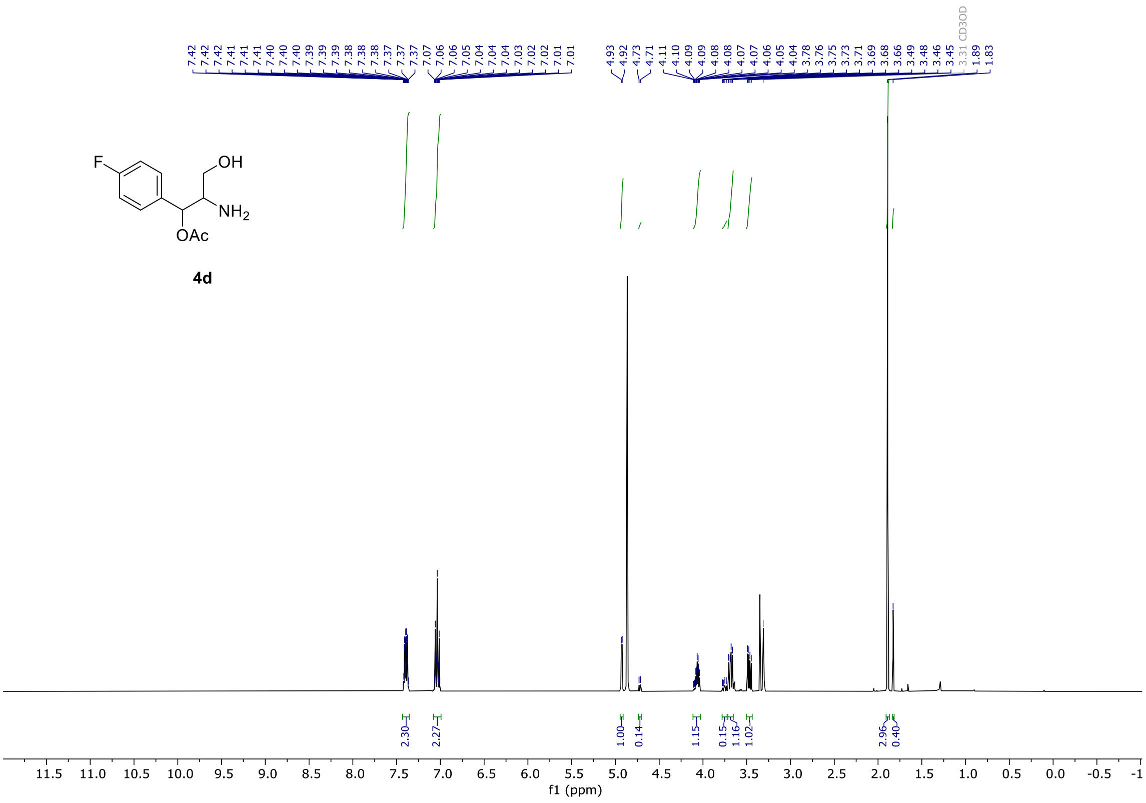

^{13}C NMR (101 MHz, MeOD)

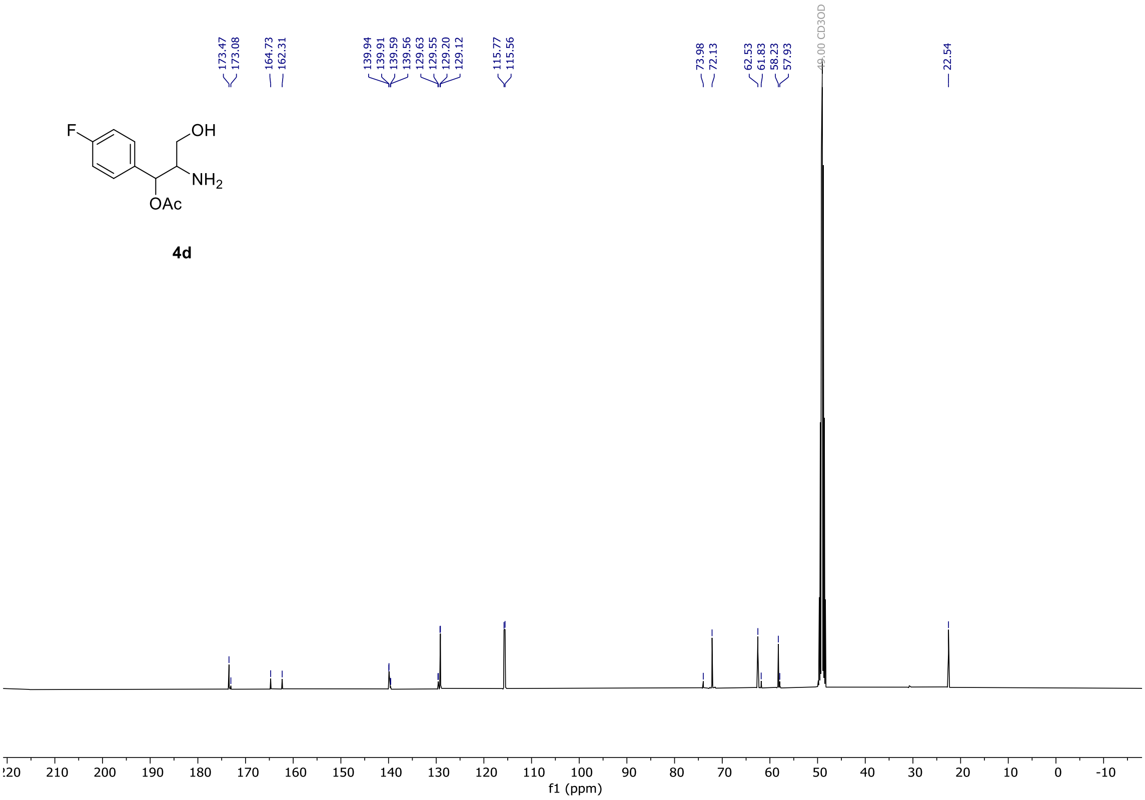

^{19}F NMR (376 MHz, MeOD)

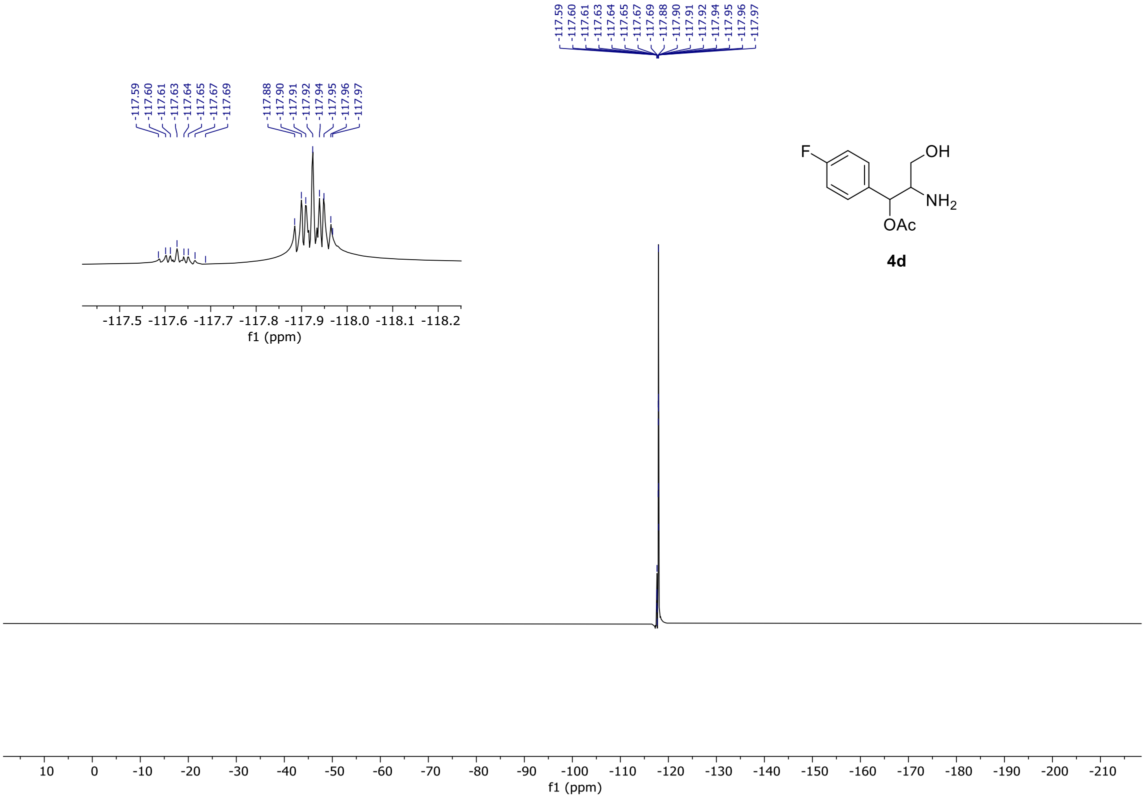

Supplement: Supplementary file 1 — ol2c01838_si_001.pdf [file ol2c01838_si_001.pdf]
